# Zona incerta somatostatin^+^ neurons modulate seizure progression through hypothalamic circuits

**DOI:** 10.1038/s41467-026-78174-9

**Published:** 2026-09-29

**Authors:** Mengdi Zhang, Lin Yang, Yuan Zhou, Yeping Ruan, Dongxiao Jiang, Lilong Yu, Jiajia Ying, Wenjing Wang, Tianyu Tang, Menghan Li, Zhong Chen, Fan Fei, Cenglin Xu, Yi Wang

**Affiliations:** 1 Zhejiang Collaborative Innovation Center for the Brain Diseases with Integrative Medicine, Zhejiang Key Laboratory of Neuropsychopharmacology, School of Pharmaceutical Sciences, The First Affiliated Hospital of Zhejiang Chinese Medical University, Hangzhou, China; 2 Medical Research Center, Shandong Medical and Pharmaceutical University Hospital, Binzhou, China; 3 College of Pharmaceutical Sciences, Zhejiang Pharmaceutical University, Ningbo, China

**Keywords:** Epilepsy, Neural circuits

## Abstract

GABAergic neurons critically regulate epileptic seizures, yet circuit mechanisms mediated by subtype-specific GABAergic neurons in the zona incerta (ZI) remain unclear. Here we reveal the essential role of ZI somatostatin^+^ GABAergic neurons in seizure modulation in the mouse hippocampal kindling model. In vivo Ca^2+^ fiber photometry and optogenetics show that ZI GABAergic neurons are gradually activated during seizure progression. Opto-activation inhibits seizure progression, especially the spread of generalized seizures at the later phase, whereas opto-inhibition accelerates it. Although both somatostatin^+^ and parvalbumin^+^ neurons are dynamically recruited during seizure progression, only somatostatin^+^ neurons exert bidirectional seizure modulation. Circuitry mapping and functional modulation demonstrate that ZI somatostatin^+^ neurons projecting to the ventromedial hypothalamus (VMH) glutamatergic neurons suppress the spread of generalized seizures by inhibiting the excitability of VMH-related downstream seizure-spread regions. These findings uncover a cell-type-specific circuit mechanism for seizure regulation by ZI somatostatin^+^ neurons, highlighting these neurons as a potential therapeutic target for epilepsy.

## Abstract (web summary)

The role of zona incerta (ZI) GABAergic subtypes in regulating epileptic seizures remains elusive. The authors find that ZI somatostatin^+^ GABAergic neurons bidirectionally control seizure spread via projections onto ventromedial hypothalamus, serving as a potential therapeutic target for epilepsy.

## Introduction

Epilepsy is one of the most common neurological disorders, affecting nearly 1% of the global population^1^. It is considered a circuitry abnormity characterized by recurrent seizure activity, caused by the heightened neuronal excitability usually associated with the imbalance betweent excitatory glutamatergic transmission and inhibitory GABAergic transmission^2,3^. Current therapeutic approaches, including anti-seizure drugs, surgical resection, and brain stimulation, show a lack of consistency and fail to control seizures adequately^4–6^. Among various epilepsy subtypes, temporal lobe epilepsy (TLE) is one of the most common drug-resistant forms, with seizures originating in limbic regions, particularly the hippocampus^6,7^-and often spreading to generalized seizures (GSs) due to the complex epileptogenic networks. Patients with seizure propagation or secondary GSs tend to have a poorer prognosis and an increased risk of sudden death. Therefore, identifying the key brain regions and neuronal circuits underlying seizure propagation in TLE is critical for developing more precise and effective interventions for seizure control.

The zona incerta (ZI), located in the subthalamic nucleus, is a brain region that mainly contains GABAergic neurons^8^, including distinct GABAergic subtypes, such as parvalbumin^+^ (PV^+^)- and somatostatin^+^ (SST^+^)-expressing neurons^9^, implicated in diverse functions, such as processing sensory information, controlling motor behavior, conveying motivational states, and participating in neural plasticity^10–15^. The ZI sends strong inhibitory projections to the thalamus, a pathway that modulates diverse neural information processing^16–18^. More importantly, our previous study has described widespread c-Fos activation across multiple brain regions, including the hippocampus, thalamus, and ZI^19^. Early work has demonstrated that the ZI is considered to be a hypersensitive region for epilepsy, as pharmacological interference^20–22^ can modulate the seizure severity. Our prior work further demonstrated that deep brain stimulation targeting the ZI greatly attenuates the seizure severity, driving its enriched inhibitory GABAergic neurons^23^. However, inconsistent findings have been reported^20–22^, suggesting that the functional contributions of ZI GABAergic neurons may depend on distinct neuronal subtypes and specific neural circuits. The detailed mechanisms underlying such cell-type-specific functions remain largely unresolved.

In this study, we employed in vivo Ca^2+^ fiber photometry, optogenetics, chemogenetics, and viral tracing to dissect the cell-specific role of ZI GABAergic neurons in the kindling-induced hippocampal seizures. We found that ZI SST^+^, but not PV^+^ GABAergic neurons, were both sufficient and essential for seizure control, especially the spread of GS. At the circuit level, the SST^+^ projection from the ZI to the ventromedial hypothalamic nucleus (VMH) constitutes a key circuit that contributes to bidirectionally modulating seizure progression in TLE, via directly inhibiting VMH glutamatergic neurons. These findings provide mechanistic insights into the cell-specific role of ZI GABAergic circuits in seizure progression and highlight a potential avenue for precise intervention targeting SST^+^ neurons.

## Results

### ZI GABAergic neurons are gradually activated during seizure progression

We first focused on the functional response of ZI neurons during kindling-induced hippocampal seizures. The experimental model involves the long-term implantation of stimulating electrodes in the ventral hippocampal CA3 region of mice, which are connected to an amplifier to deliver current from a constant-current stimulator. A current of 20 Hz for 2 s (1 ms pulses, 0.4 mA), triggers discharges that induce hippocampal seizures^24–26^. We performed immunohistochemical staining for c-Fos, encoding an immediate early response gene (neural activity marker), and found that c-Fos expression in the ZI was significantly increased after seizures, compared with the sham group (Supplementary Fig. 1a, c). As a positive control, the hippocampus (including the DG, CA1, and CA3 regions) also exhibited a significant increase in c-Fos expression following seizures (Supplementary Fig. 1b, d). Collectively, these data indicate that ZI neurons are activated during hippocampal seizures.

**Fig. 1:**
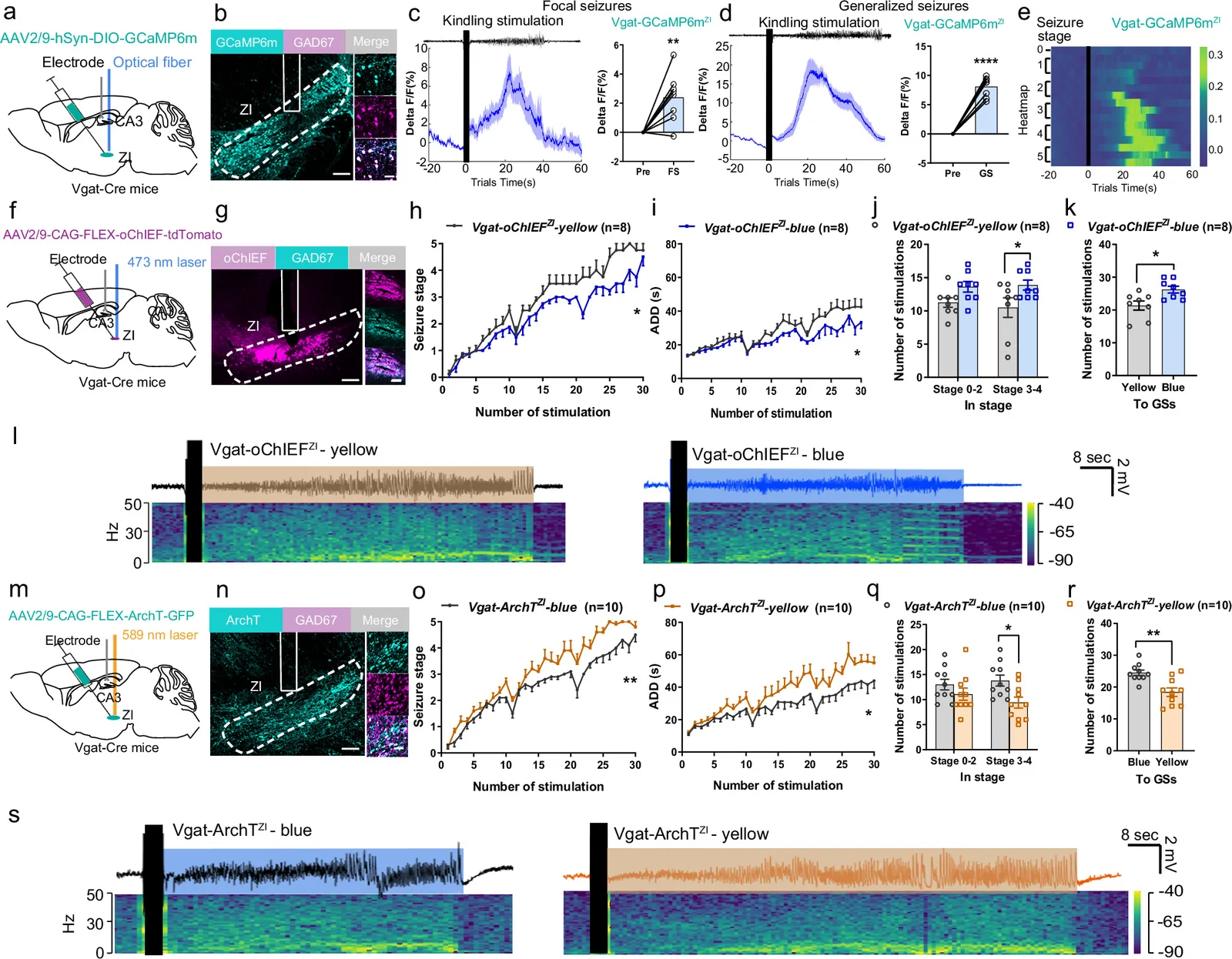
ZI GABAergic neurons bidirectionally modulate seizure progression. **a** Schematic for virus injection and Ca^2+^ fiber photometry of ZI GABAergic neuron during hippocampal seizures in *Vgat-Cre* mice. **b** Representative ZI GCaMP6m expression/GAD67 co-labelling. Scale bars: 200 μm (Left), 20 μm (Right). **c, d** Average calcium signals (∆*F/F*) and group quantification for ZI GABAergic neuron during FSs and GSs (*n* = 8 mice). **e** Representative heatmap of calcium signals across seizure stages from one *Vgat-GCaMP6m*^*ZI*^ mouse. **f** Schematic for virus injection and opto-activation in *Vgat-oChIEF*^*ZI*^ mice. **g** Representative oChIEF expression/GAD67 co-staining. Scale bar: 200 μm (Left), 50 μm (Right). Effects of opto-activation of ZI GABAergic neurons on the development of seizure stage (**h**), ADD (**i**), stage-specific stimulation numbers (**j**) and the number of stimulations of GSs onset (**k**) (*n* = 8 mice per group). **l** Representative EEGs and power spectrogram for oChIEF groups. **m** Schematic for viral injection and optogenetic inhibition in *Vgat-ArchT*^*ZI*^ mice. **n** ArchT expression in ZI with GAD67 co-staning. Scale bar: 200 μm (Left), 50 μm (Right). Effects of opto-inhibition of ZI GABAergic neurons on the development of seizure stage (**o**), ADD (**p**), stage-specific stimulation numbers (**q**) and the number of stimulations of GS onset (**r**) (*n* = 10 mice per group). **s** Representative EEGs and power spectrogram for ArchT groups. Statistical analyses: Two-tailed paired Student’s *t-*test for (**c**, **d**), Two-way ANOVA for repeated measures for (**h**, **i**, **o**, **p**). Two-way ANOVA with post-hoc Bonferroni tests for (**j**, **q**). Two-tailed unpaired Student’s *t*-test for (**k**, **r**). Significance levels were presented as ^*^P < 0.05, ^**^P < 0.01, or ^****^P < 0.0001. Data are presented as means ± SEM. Exact *P* values are provided in Supplementary Data 1. Source data are provided as a Source Data file. (ZI zona incerta, CA3 hippocampal CA3, FS focal seizure, GS generalized seizure, ADD after-discharge duration).

Given that GABAergic neurons constitute the predominant neuronal type in the ZI^8^, we further paid attention to the functional dynamics of ZI GABAergic neurons during hippocampal seizures. For this purpose, we stereotaxically injected the *Cre*-dependent adeno-associated virus AAV2/9-hSyn-DIO-GCaMP6m, a genetically encoded calcium indicator, into the ZI of *Vgat-Cre* mice (hereafter referred to as *Vgat-GCaMP6m*^*ZI*^ mice), and implanted an optical cannula above the ZI for recording GCaMP fluorescence signals during kindling-induced hippocampal seizures (Fig. 1a, b). We observed significantly elevated GCaMP fluorescence signals during both focal seizure (FS) and GS in the seizure progression (Fig. 1c, d). Notably, calcium signals in ZI GABAergic neurons gradually increased along with the development of seizure severity during kindling acquisition (Fig. 1e). The peak calcium signals during GS were higher than those during FS (Supplementary Fig. 1e), and the GCaMP6 signal intensity was significantly correlated with the after-discharge duration (ADD) of seizures (Supplementary Fig. 1f), indicating that the activation level of ZI GABAergic neurons intensifies with seizure progression. Considering that the ZI is closely related to motor control^27^ to rule out the possibility that the increased GCaMP fluorescence signals were caused by the convulsive seizure (movement artifact), we recorded ZI GCaMP fluorescence signals during hippocampal kindling-induced seizures in *Vgat-GCaMP6m*^*ZI*^ mice under systematic anesthesia induced by isoflurane. Consistently, elevated GCaMP fluorescence in ZI GABAergic neurons was recapitulated in isoflurane-anesthesia mice during seizures (Supplementary Fig. 1g, h), and the calcium signal intensity remained positively correlated with ADD during seizures (Supplementary Fig. 1i). Consistently, these results imply that ZI GABAergic neurons are dynamically activated during seizure progression.

### ZI GABAergic neurons bidirectionally modulate seizure progression

To investigate the causal role of ZI GABAergic neurons in hippocampal seizures, we stereotaxically injected the AAV2/9-CAG-FLEX-oChIEF-tdTomato virus into the ZI of *Vgat-Cre* mice to selectively optogenetically activate ZI GABAergic neurons (Fig. 1f, g). In the hippocampal kindling model, photo-stimulation (473 nm, 20 Hz, 5 mW, 10 ms) was delivered to the ipsilateral ZI immediately following each hippocampal kindling stimulation, which is similar to a closed-loop stimulation pattern^28^. We found that optogenetic activation of ZI GABAergic neurons retarded the progression of seizure stage, shortened ADD (Fig. 1h, i), and significantly increased the number of stimulations in 3−4 seizure stage and reaching GS compared with yellow-light control (Fig. 1j−l, and Supplementary Fig. 2d). Interestingly, optical fiber implantation targeting the anterior ZI (covering the majority of the ZI region) showed a significant impact on the seizure activity, whereas the optical fiber placement at ZI edge showed a relatively weaker effect on the seizure behavior (Supplementary Fig. 2a−c). These results indicated that distinct ZI subregions may differentially affect seizure severity, which may be related to the different location of subtype GABAergic neurons in the ZI^9^.

To further test whether ZI GABAergic neurons are required for seizure progression, we further used optogenetics to selectively inhibit ZI GABAergic neurons by injecting the AAV2/9-CAG-FLEX-ArchT-GFP virus into the ZI of *Vgat-Cre* mice (Fig. 1m, n). We found that yellow photo-inhibition of ZI GABAergic neurons accelerated the progression of seizure stage, prolonged ADD, and significantly decreased the number of stimulations in 3−4 seizure stage and reaching GS compared with the blue light control group (Fig. 1o−s, and Supplementary Fig. 2e). All of the above data showed that ZI GABAergic neurons bidirectionally regulated seizure progression, especially the spread of GS during the later phase. Specifically, activation of ZI GABAergic neurons alleviated seizure progression, whereas their inhibition accelerates seizure progression.

### Frequency-dependent anti-seizure effects of ZI PV^+^ and SST^+^ GABAergic neurons

As PV+ and SST+ neurons are the main subtypes of GABAergic neurons distributed in the ZI^9^, we further clarify the cell-type specificity of ZI GABAergic neurons mediating seizure progression. We first performed double immunofluorescence staining for PV or SST in neurons activated after seizures in the ZI. The quantitative analysis revealed that 23.6% of the seizure-activated neurons were identified as PV^+^, while 48.9% were SST^+^ (Supplementary Fig. 3a−f), indicating that SST^+^ GABAergic neurons are preferentially recruited during seizures. Interestingly, c-Fos^+^ cells that co-localized with the PV^+^ or SST^+^ neuronal subsets exhibited spatial variation across ZI subregions: the c-Fos^+^ PV neurons were predominantly distributed in the ZIv (45.8%) rather than ZIr (11.6%); however, the c-Fos^+^ SST neurons were enriched in the ZIr (56.6%) relative to the ZIv (30.4%) (Supplementary Fig. 3a−f). Additionally, hippocampal seizures also activated 15.2% of nNOS neurons in the ZIr, while exerting no obvious effects on VGLUT2 neurons (Supplementary Fig. 3g−l).

**Fig. 2:**
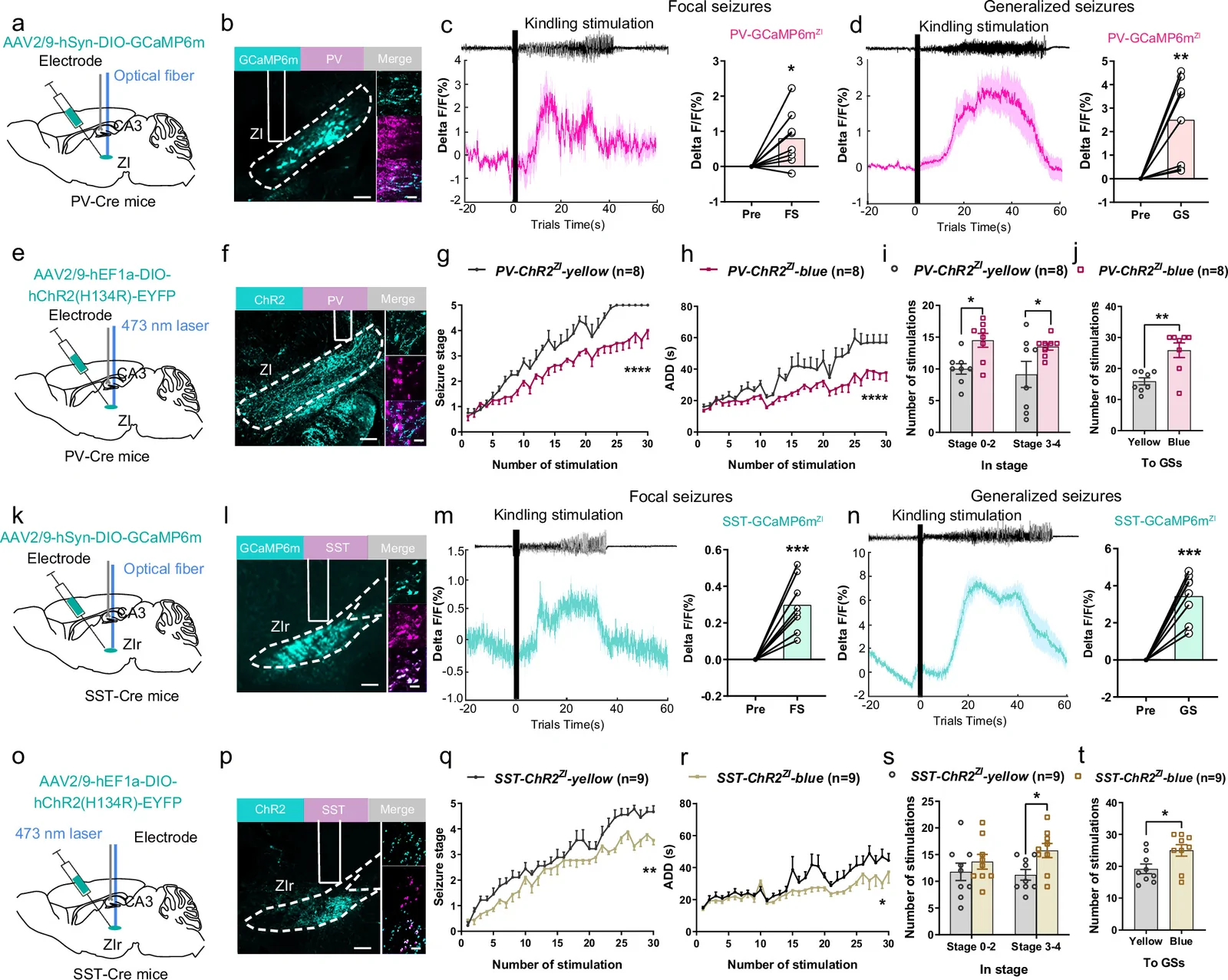
Optogenetic activation of ZI PV^+^ or SST^+^ GABAergic neurons attenuates seizure progression. **a** Scheme for virus injection and Ca^2+^ fiber photometry of ZI PV^+^ neuron during hippocampal seizures in *PV-Cre* mice. **b** Representative ZI GCaMP6m expression/PV co-labelling. Scale bars: 200 μm (Left), 20 μm (Right). **c**, **d** Average calcium signals (∆*F/F*) and group quantification for PV^+^ neuron during FSs and GSs (*n* = 8 mice). **e** Experimental schematic for optogenetic activation in *PV-ChR2*^*ZI*^ mice. **f** Representative ZI ChR2/PV co-expression images. Scale bar: 200 μm (Left), 50 μm (Right). Seizure stage (**g**), ADD (**h**), stage-specific stimulation numbers (**i**) and the number of stimulations of GS onset (**j**) (*n* = 8 mice per group). **k** Scheme for virus injection and Ca^2+^ fiber photometry of ZI SST^+^ neuron during seizures in *SST-Cre* mice. **l** Representative ZI GCaMP6m expression/SST co-labelled. Scale bars: 150 μm (Left), 50 μm (Right). **m**, **n** ∆*F/F* and group quantification of ZI SST^+^ neuron during FSs and GSs (*n* = 8 mice). **o** Schematic for virus injection and opto-activation in *SST-ChR2*^*ZI*^ mice. **p** Representative ZI ChR2/SST co-staining. Scale bar: 150 μm (Left), 50 μm (Right). Seizure stage (**q**) ADD (**r**), stage-specific stimulation numbers (**s**) and the number of stimulations of GS onset (**t**) (*n* = 9 mice per group). Statistical analyses: Two-tailed paired Student’s *t*-test for (**c**, **d**, **m**, **n**). Two-way ANOVA for repeated measures for (**g**, **h**, **q**, **r**); Two-way ANOVA with post-hoc Bonferroni tests for (**i**, **s**); Two-tailed Mann–Whitney U test for (**j**, **t**). Significance: ^*^P < 0.05, ^**^P < 0.01, or ^****^P < 0.0001. Data are presented as means ± SEM. Exact *P* values are provided in Supplementary Data 1. Source data are provided as a Source Data file. (ZI zona incerta, ZIr rostral zona incerta, CA3 hippocampal CA3, FS focal seizure, GS generalized seizure, ADD after-discharge duration).

Then, we targeted ZIv PV^+^ or ZIr SST^+^ GABAergic neurons in *PV-Cre* or *SST-Cre* mice by expressing GCaMP6s, followed by optical fibers implantation to monitor calcium dynamics during hippocampal seizures (Fig. 2a, b, k, l). We detected a slight and significant increase in GCaMP fluorescence in both PV^+^ and SST^+^ neurons during FS and GS stages, respectively (Fig. 2c, d, m, n), indicating that ZI PV^+^ or SST^+^ GABAergic neurons are gradually activated during seizure progression. Interestingly, we observed distinct intrinsic dynamic patterns in calcium activity between PV^+^ and SST^+^ neurons. Further analysis of calcium signal duration of PV^+^ and SST^+^ neurons during hippocampal seizures revealed that SST^+^ neurons tended to exhibit a faster increase in calcium signals with a slightly longer duration, as indicated by the steeper slope in SST during GS compared to those of PV^+^ neurons (Supplementary Fig. 4a, b). The changed calcium signals of SST^+^ neurons have a significant correlation with both seizure stage and ADD of hippocampal seizures (Supplementary Fig. 4c, d). Meanwhile, we investigated whether alterations in stimulation intensities modulated ZI calcium signals, and found that ZI calcium signals increased progressively with increasing stimulation intensity (Supplementary Fig. 4e−h). These results suggested that ZI PV^+^ and SST^+^ GABAergic neurons may be innervated with a different circuit organization.

To investigate the causal roles of ZI PV^+^ and SST^+^ GABAergic neurons in seizure progression, we used an optogenetic approach to selectively stimulate Channelrhodopsin 2 (ChR2)-expressing PV^+^ or SST^+^ GABAergic neurons in the ZI (Fig. 2e, f, o, p). Blue photo-activation of either ZI PV^+^ or SST^+^ GABAergic neurons strongly retarded the progression of seizure stage, shortening ADD, and increasing the number of stimulations in 3−4 seizure stage and reaching GS of kindling-induced seizures compared with yellow light controls (Fig. 2g−j, q−t, and Supplementary Fig. 5a, c). This cell-type-specific optogenetic activation broadly attenuated EEG power spectra (Supplementary Fig. 6a, b), with pronounced suppression of delta band power during GS (Supplementary Fig. 6i, j). To investigate whether the distinct spontaneous firing frequencies of PV^+^ and SST^+^ neurons contribute to hippocampal seizures, we set three gradient photo-stimulation paradigms (10 Hz, 20 Hz, and 40 Hz) for targeted neuronal activation. Photo-stimulation of ZI PV^+^ neurons at 10 Hz, 20 Hz, and 40 Hz all significantly attenuated seizure progression, with the inhibitory effect on ADD progressively increasing as stimulation frequency increased (Supplementary Fig. 7a−c). In contrast, ZI SST^+^ neuronal activation exerted prominent anti-seizure effects under 10 Hz and 20 Hz photo-stimulation, whereas no obvious seizure suppression was observed at 40 Hz photo-stimulation (Supplementary Fig. 7d−f).

To further confirm the regulatory role of ZI SST^+^ neuronal excitability in hippocampal seizures and eliminate potential artifacts caused by optogenetic manipulation, we performed chemogenetic activation experiments. Consistent with our optogenetic activation results, chemogenetic activation of ZI SST^+^ neurons significantly attenuated hippocampal seizure progression (Supplementary Fig. 8). These results further solidified the inhibitory regulatory role of ZI SST^+^ neurons in hippocampal seizures. Together, these data indicated that both ZI PV^+^ and SST^+^ GABAergic neurons exhibited significant activation during seizure progression, and activation of ZI PV^+^ and SST^+^ GABAergic neurons effectively retarded the seizure progression.

### Inhibition of ZI SST^+^ but not PV^+^ GABAergic neurons amplifies seizure progression

To further test whether ZI PV^+^ and SST^+^ GABAergic neurons exert an inhibitory constraint on seizure progression, the AAV2/9-CAG-DIO-taCaspase3-TEVp virus was stereotaxically injected into the ZI of *PV-Cre* mice or *SST-Cre* mice for selective ablation of PV^+^ or SST^+^ GABAergic neurons, while the AAV2/9-EF1ɑ-DIO-EYFP was injected as the control virus (Supplementary Fig. 9a−c, i−k). In the kindling-induced hippocampal seizures, we found that specifictive ablation of ZI PV^+^ GABAergic neurons had no significant effect on seizure progression (Supplementary Fig. 9d−h). In contrast, specifically ablation of ZI SST^+^ GABAergic neurons significantly accelerated the progression of seizure stage, prolonged ADD, and decreased the number of stimulations in 3−4 seizure stage and reaching GS (Supplementary Fig. 9l−p), suggesting that ZI SST^+^ GABAergic neurons (not PV^+^ GABAergic neurons) exert an inhibitory constraint on seizure progression, and loss of ZI SST^+^ neuronal function exacerbates seizure progression.

**Fig. 3:**
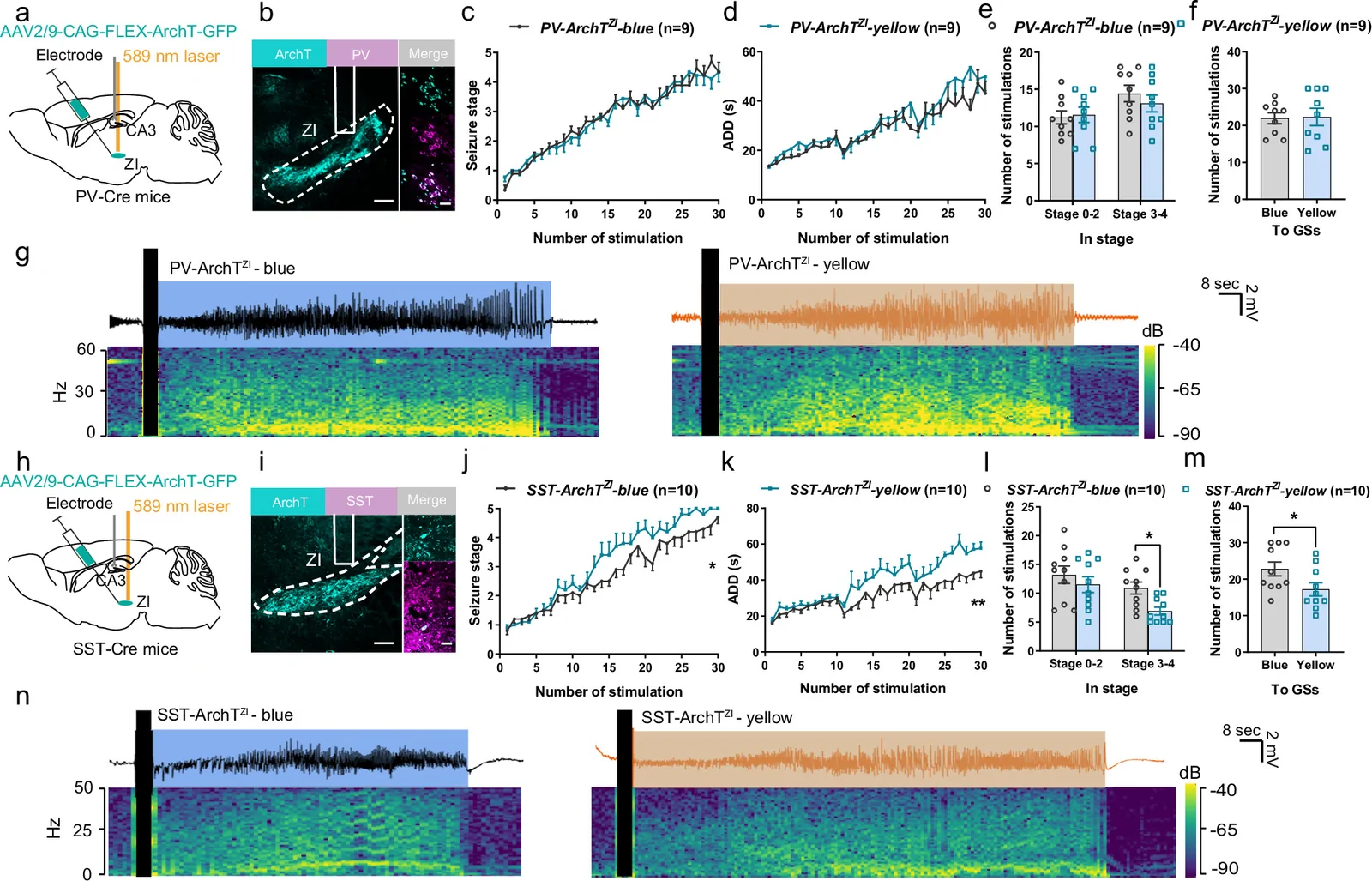
Optogenetic inhibition of ZI SST^+^ but not PV^+^ GABAergic neurons amplifies seizure progression. **a** Schematic for virus injection and optogenetic inhibition in *PV-ArchT*^*ZI*^ mice. **b** Representative ArchT expression in ZI with PV co-staining. Scale bar: 200 μm (Left), Scale bar: 50 μm (Right). Effects of optogenetic inhibition of ZI SST^+^ GABAergic neurons on the development of seizure stage (**c**), ADD (**d**), stage-specific stimulation numbers (**e**) and the number of stimulations of GS onset (**f**) (*n* = 9 mice per group). **g** Representative EEGs and power spectrogram during seizures. **h** Schematic for virus injection and optogenetic inhibition in *SST-ArchT*^*ZI*^ mice **i** Representative ArchT expression in ZI with SST co-staining. Scale bar: 150 μm (Left), Scale bar: 50 μm (Right). Seizure stage (**j**) ADD (**k**), stage-specific stimulation numbers (**l**) and the number of stimulations of GS onset (**m**) (*n* = 10 mice per group). Effects of optogenetic inhibition of ZI SST^+^ GABAergic neurons on the development of seizure stage (**j**), ADD (**k**), the number of stimulations at the seizure stage (**l**) and the number of stimulations of GSs (**m**). *SST-ArchT*^*ZI*^*-blue*, n = 10 mice; *SST-ArchT*^*ZI*^*-yellow*, *n* = 10 mice. **n** Representative EEGs and power spectrogram during seizures. Statistical analyses: Two-way ANOVA for repeated measures for (**c**, **d**, **j**, **k**); Two-way ANOVA with post-hoc Bonferroni test for (**e**); Two-tailed unpaired Student’s *t*-test for (**f**, **m**). Significance levels were presented as ^*^P < 0.05, or ^**^P < 0.01. Data are presented as means ± SEM. Exact *P* values are provided in Supplementary Data 1. Source data are provided as a Source Data file. (ZI zona incerta, CA3 hippocampal CA3, PV parvalbumin, SST somatostatin, GS generalized seizure, ADD after-discharge duration).

To further verify the above findings, we injected the AAV2/9-CAG-FLEX-ArchT-GFP virus into the ZI of *PV-Cre* mice or *SST-Cre* mice to selectively inhibit ZI PV^+^ and SST^+^ GABAergic neurons. Consistent with the results of the neuronal ablation experiment, optogenetic inhibition of PV^+^ GABAergic neurons had no significant effect on seizure progression (Fig. 3a−g, and Supplementary Fig. 6c, g, k). In contrast, optogenetic inhibition of ZI SST^+^ GABAergic neurons markedly accelerated the progression of seizure stage, prolonged ADD, decreased the number of stimulations in 3−4 seizure stage and reached GS (Fig. 3h−n, and Supplementary Fig. 5b, d). Correspondingly, inhibition of ZI SST⁺ neurons elevated global EEG power spectra (Supplementary Fig. 6d), alongside a pronounced rise in delta band power during both FS and GS (Supplementary Fig. 6h, l). A direct comparison of the effects of optogenetic activation or inhibition of ZI PV^+^ and SST^+^ neurons was performed, with emphasis on their differential efficacy in delaying seizure progression. Quantitative analysis showed the two neuronal types differed in their influence on seizure dynamics (Supplementary Fig. 10). Taken together, these findings demonstrated that ZI SST^+^ neurons were the critical GABAergic subtype responsible for the bidirectional regulation of seizure progression by ZI GABAergic neurons. Notably, inhibition of ZI GABAergic neurons, particularly the SST^+^ subtype, amplified seizure progression.

### The ZI^SST^-VMH projections mediate the role of ZI SST^+^ GABAergic neurons in seizure progression

**Fig. 4:**
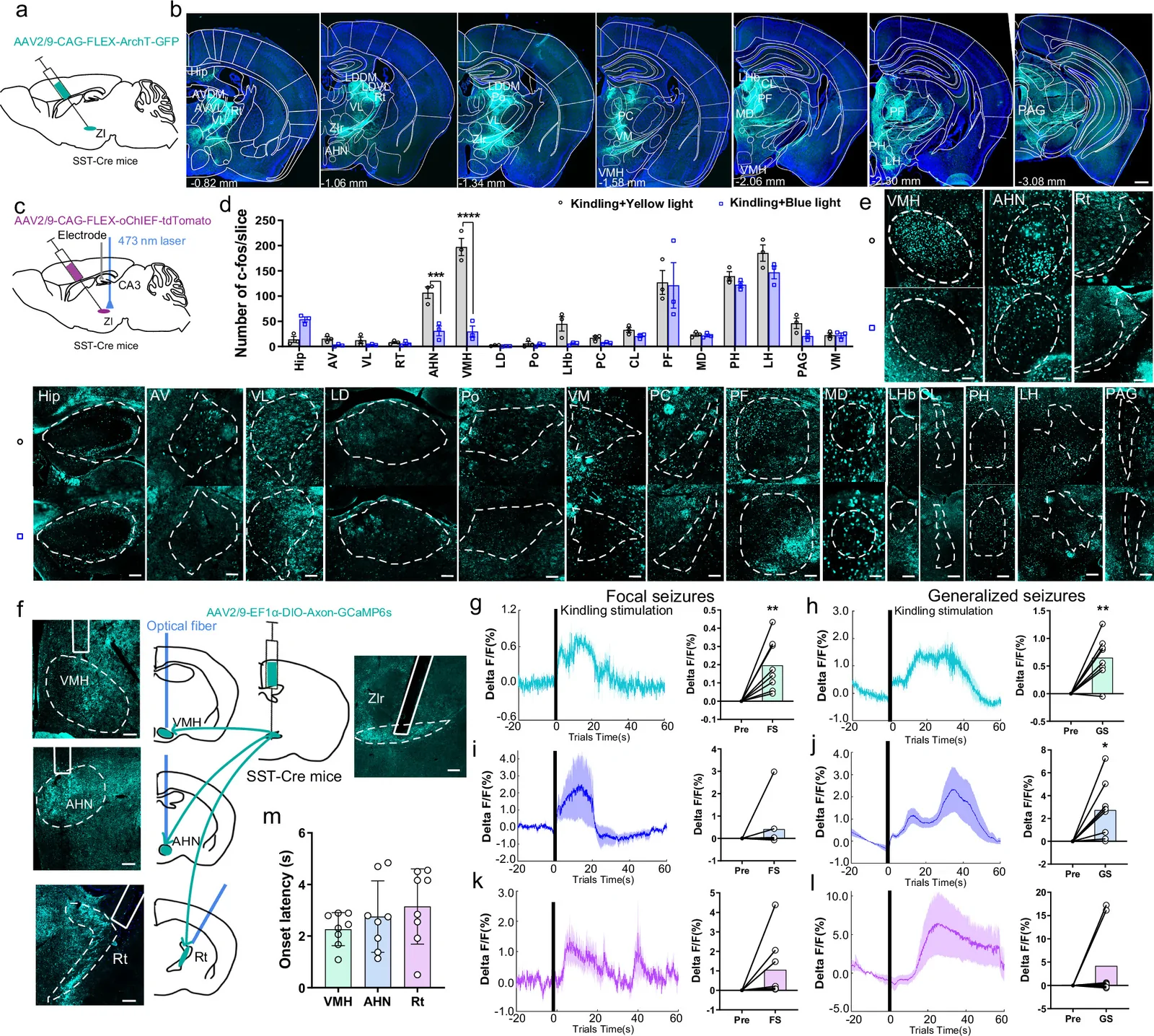
The structural and functional mapping of downstream brain area of ZI SST^+^ GABAergic neurons involved in seizure progress. **a** Schematic of virus injection for *SST-ArchT*^*Z*I^ mice. **b** Axonal terminals mapping of ZI SST^+^ neurons. Scale bar = 600 μm. *n* = 3 mice. **c** Scheme for virus injection and optogenetic activation in *SST-oChIEF*^*Z*I^ mice. **d** Quantification of c-Fos^+^ cells across brain regions innervated by ZI SST^+^ fibres. **e** Representative c-Fos immunostaining images. *n* = 3 mice. Scale bar = 300 μm (VMH, AV, PAG, PH); 200 μm (AHN, Rt, LD, LHb, MD, CL; 400 μm (Hip, LH); 350  μm (VL, Po, PF, VM, PC). **f** Schematic for fiber photometry of ZI^SST^-VMH, ZI^SST^-AHN, or ZI^SST^-Rt axons during seizures. Representative axonGCaMP6s (Cyan) expression. Scale bar = 200 μm. **g**−**l** Averaged axonal calcium activities and group statistics during FSs and GSs. *n* = 8 mice. **m** Onset latency of ZI^SST^-VMH, ZI^SST^-AHN and ZI^SST^-Rt circuits in hippocampal seizures. *n* = 8 mice. Statistical analyses: Two-way ANOVA with post-hoc Bonferroni test for (**d**), Two-tailed paired Student’s *t*-test for (**g**−**l**). Data are mean ± SEM. Exact *P* values are provided in Supplementary Data 1. Significance levels were presented as ^*^P < 0.05, ^**^P < 0.01, ^***^P < 0.001, or ^****^P < 0.0001. Source data are provided as a Source Data file. FS focal seizure, GS generalized seizure, SST somatostatin, ZI zona incerta, CA3 hippocampal CA3, AVDM anteroventral thalamic nucleus, dorsomedial part, AVVL anteroventral thalamic nucleus, ventrolateral part, AHN anterior hypothalamic area, CL centrolateral thalamic nucleus, LH lateral hypothalamic area, LHb lateral habenular nucleus, LDDM laterodorsal thalamic nucleus, dorsomedial part, LDVL laterodorsal thalamic nucleus, ventrolateral part, MD mediodorsal thalamic nucleus, PAG periaqueductal gray, PC paracentral thalamic nucleus, PF parafascicular thalamic nucleus, PH posterior hypothalamic nucleus, Po posterior thalamic nuclear group, Rt reticular nucleus (prethalamus), VL ventrolateral thalamic nucleus, VM ventromedial thalamic nucleus, VMH ventromedial hypothalamic nucleus.

Next, we aimed to test the downstream circuit mechanism by which ZI SST^+^ GABAergic neurons, which are involved in hippocampal seizure progression. We first injected the AAV2/9-CAG-FLEX-ArchT-GFP virus into the ZI of *SST-Cre* mice to map the downstream fiber distribution of ZI SST^+^ GABAergic neurons (Fig. 4a). GFP fluorescence was apparent at soma in ZI and in projections within the thalamus and hypothalamus, including the parafascicular thalamic nucleus (PF), ventrolateral thalamic nucleus (VL), ventromedial hypothalamic nucleus (VMH), anterior hypothalamic area (AHN), and the reticularis nucleus (Rt), and other regions (Fig. 4b). To delineate the seizure-regulating downstream regions, we performed selective optogenetic activation of ZI SST^+^ GABAergic neurons during kindling-induced hippocampal seizures (Fig. 4c). Ninety minutes after optogenetic manipulation, the mice were euthanized for c-Fos immunohistochemical analysis of the above downstream brain regions (Fig. 4e). Notably, seizure-responsive c-Fos activation in the VMH and AHN was greatly decreased following optogenetic activation of ZI SST^+^ GABAergic neuronal soma, whereas other downstream nucleus within ZI SST^+^ GABAergic neuronal fiber showed no statistically significant changes in c-Fos expression (Fig. 4d). Therefore, these results revealed that the VMH and AHN may be the principal efferent targets of ZI SST^+^ GABAergic neurons involved in seizure-suppressing effects.

To assess which downstream circuits mediate the seizure-modulating effects of ZI SST^+^ GABAergic neurons, we first assessed the activity of ZI^SST^-VMH, ZI^SST^-AHN and ZI^SST^-Rt circuits in hippocampal seizures. We prioritized ZI^SST^-VMH and ZI^SST^-AHN circuit, which demonstrated both abundant synaptic terminals and reduced downstream c-Fos expression upon somatic activation of ZI SST^+^ GABAergic neurons, whereas the ZI^SST^-Rt circuit, which showed comparable projection strength but lacked a c-Fos response, served as an isointensity negative control. To specifically monitor the ZI SST⁺ projections activity during hippocampal seizures, we injected axon-targeted GCaMP6s (AAV-EF1ɑ-DIO-axon-GCaMP6s) into ZI SST^+^ neurons in *SST-Cre* mice, enabling ZI and downstream circuits terminal calcium activity monitoring by implantation of fiber photometry fibers into the VMH, AHN, and Rt during the hippocampal seizures (Fig. 4f). The results showed that the calcium signal of the ZI^SST^-VMH circuit was activated during both FS and GS, whereas the ZI^SST^-AHN circuit was only activated during GS (Fig. 4g−m).

**Fig. 5:**
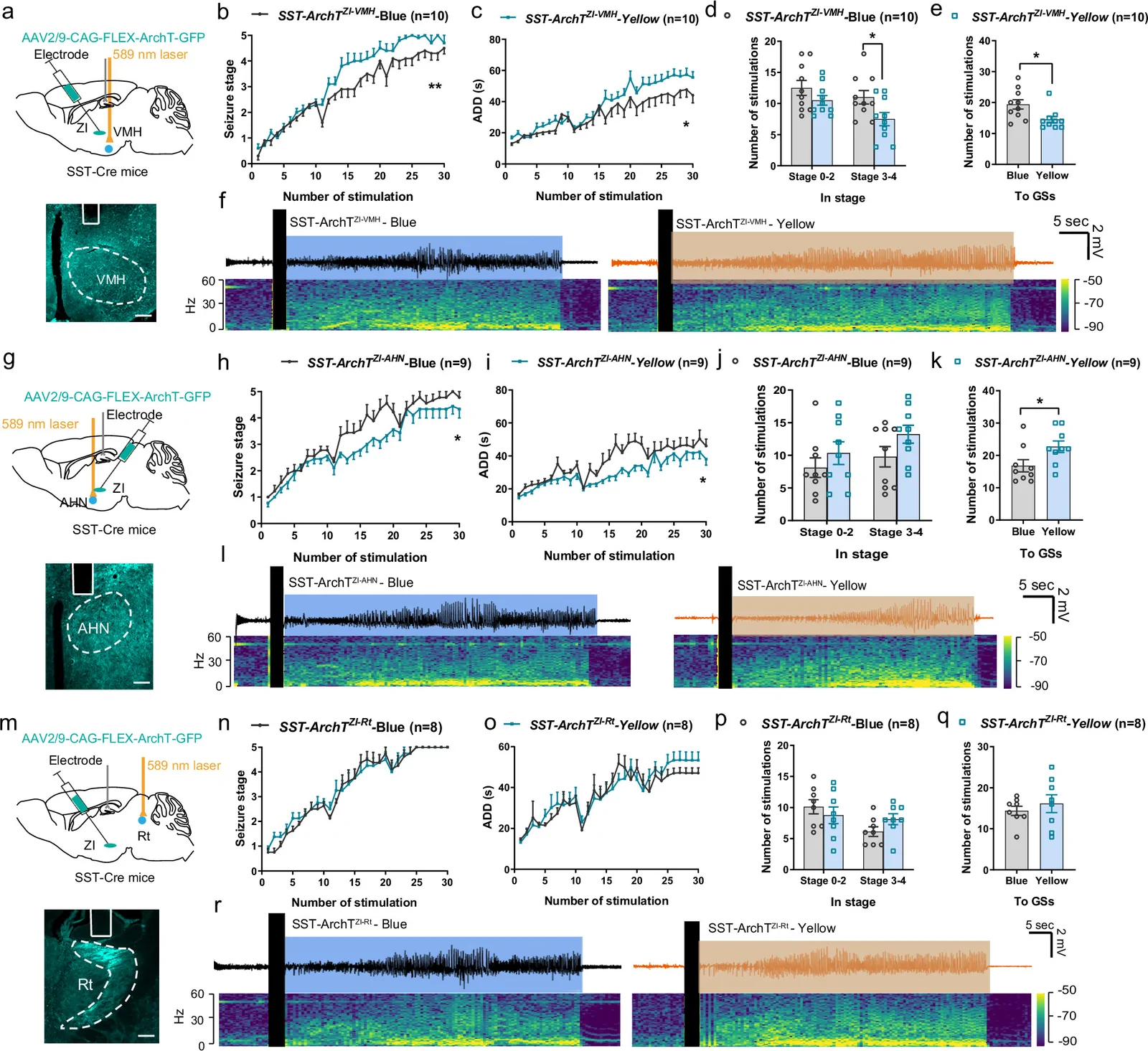
Optogenetic inhibition of the ZI^SST^-VMH, ZI^SST^-AHN or ZI^SST^-Rt circuit produces divergent effects on seizures progression. **a** Left: Schematic of viral injection, optical fiber implantation and optogenetic inhibition in *SST-ArchT*^ZI-VMH^ mice; Right: Representative image of fiber location in the VMH. Scale bar = 200 μm. Effects of opto-inhibition of ZI^SST^-VMH circuit on the development of seizure stage (**b**), ADD (**c**), stage-specific stimulation numbers (**d**) and the number of stimulations of GSs onset (**e**) (*n* = 10 mice per group). **f** Representative EEGs and power spectrogram during seizures for *SST-ArchT*^*ZI-VMH*^ mice. **g** Left: Schematic of viral injection, optical fiber implantation and opto-inhibition in *SST-ArchT*^*ZI-AHN*^ mice; Right: Representative fiber position in the AHN. Scale bar = 200 μm. Effects of opto-inhibition of ZI^SST^-AHN circuit on the development of seizure stage (**h**), ADD (**i**), stage-specific stimulation numbers (**j**) and the number of stimulations of GS onset (**k**) (*n* = 9 mice per group). **l** Representative EEGs and power spectrogram during seizures of *SST-ArchT*^*ZI-AHN*^ mice. **m** Left: Schematic of viral injection, optical fiber implantation and opto-inhibition in *SST-ArchT*^*ZI*-Rt^ mice; Right: Representative fiber position in the Rt. Scale bar = 200 μm. Effects of opto-inhibition of ZI^SST^-Rt criuit on the development of seizure stage (**n**), ADD (**o**), stage-specific stimulation numbers (**p**) and stimulations to GS onset (**q**) (*n* = 8 mice per group). **r** Representative EEGs and power spectrogram during seizures of *SST-ArchT*^*ZI*-Rt^ mice. Statistical analyses: Two-way ANOVA for repeated measures for (**b**, **c**, **h**, **i**, **n**, **o**); Two-way ANOVA with post-hoc Bonferroni test for (**d**, **j**, **p**); Two-tailed Mann–Whitney U test for (**k**, **e**); Two-tailed unpaired Student’s *t*-test for (**q**). Significance levels were presented as ^*^P < 0.05, or ^**^P < 0.01. Data are presented as means ± SEM. Exact *P* values are provided in Supplementary Data 1. Source data are provided as a Source Data file. (ZI zona incerta, CA3 hippocampal CA3, PV parvalbumin, SST somatostatin, ADD after-discharge duration, GS generalized seizure, VMH ventromedial hypothalamus, AHN anterior hypothalamic area, Rt reticularis nucleus).

Furthermore, we assessed the effects of inhibiting ZI SST^*+*^ projection fibers innervating the VMH, AHN, and Rt on hippocampal seizures. Optical probes were implanted into the VMH (referred to as *SST-ArchT*^*ZI-VMH*^ mice, Fig. 5a), AHN (referred to as *SST-ArchT*^*ZI-AHN*^ mice, Fig. 5g), and Rt (referred to as *SST-ArchT*^*ZI-Rt*^ mice, Fig. 5m) of *SST-Cre* mice to enable targeted optogenetic inhibition of ZI^SST^-VMH, ZI^SST^-AHN, and ZI^SST^-Rt circuits (Supplementary Fig. 11b−d). The results showed that photo-inhibition of ZI^SST^-VMH circuit significantly accelerated the progression of seizure stage, prolonged ADD, and decreased the number of stimulations in 3−4 seizure stage and reaching GS (Fig. 5b−f), while photo-inhibition of ZI^SST^-AHN circuit even slightly retarded the progression of seizure stage, shortened ADD, and increased the number of stimulations reaching GS (Fig. 5h−l). Additionally, photo-inhibition of ZI^SST^-Rt circuit had no significant effect on the progression of hippocampal seizures (Fig. 5n−r).

Given that optogenetic inhibition of the ZI^SST^-VMH circuit produced comparable seizure-promoting effects to direct soma inhibition of ZI SST^+^ GABAergic neurons, we next investigated whether circuit-specific activation could recapitulate the anti-seizure outcomes observed with soma manipulation. We found that photo-activation of the ZI^SST^-VMH circuit (referred to as *SST-oChIEF*^*ZI-VMH*^ mice, Supplementary Fig. 12a) strongly retarded the progression of seizure stage, shortened ADD, and increased the number of stimulations in 3-4 seizure stage and reaching GS (Supplementary Figs. 11a and 12b−f). To further test whether GABA signaling or neuropeptide somatostatin (SST) signaling mediates the seizure-modulating effects of the ZI^SST^-VMH circuit, we performed pharmacological interventions during optogenetic activation of this circuit. Specifically, during the ZI^SST^-VMH circuit activation, bicuculline (GABA_A_ receptor antagonist) or CYN-154806 (somatostatin receptor antagonist) was locally infused into the VMH, while the control group received saline (Supplementary Fig. 13). Compared with the blue-light group, bicuculline mainly reversed the later phase of kindling-induced seizure development, as evidenced by a significant reduction in the number of stimulations required to stay in stages 3−4, while CYN-154806 reversed the whole phase of kindling-induced seizure development, significantly reducing the number of stimulations required to stay in both seizure stages 0−2 and stages 3−4. Antagonizing SST receptors locally also reproduced a pro-seizure effect on ADD. These results indicate that both GABA_A_ receptors and somatostatin receptors may contribute to the anti-seizure effect of the ZI^SST^-VMH circuit.

The ZI^SST^-AHN circuit produces seizure-modulating effects distinct from those of the ZI-VMH circuit. To explore the anatomical basis underlying such functional divergence, we injected AAV2/2Retro-hEF1α-DIO-EGFP and AAV2/2Retro-hEF1α-DIO-mCherry into the VMH and AHN of *SST-Cre* mice, respectively. Quantitative anatomical mapping across the ZI revealed heterogeneous projection patterns: AHN-projecting SST neurons predominantly originated from the ZIv (30.58%) and ZIr (64.29%), whereas VMH-projecting SST neurons were concentrated in the ZIr (56.07%) (Supplementary Fig. 14). These anatomical differences likely serve as the structural basis for the opposing functional roles of the two circuits.

### ZI SST^+^ neurons mediate anti‑seizure effects through inhibition of downstream VHM glutamatergic populations

**Fig. 6:**
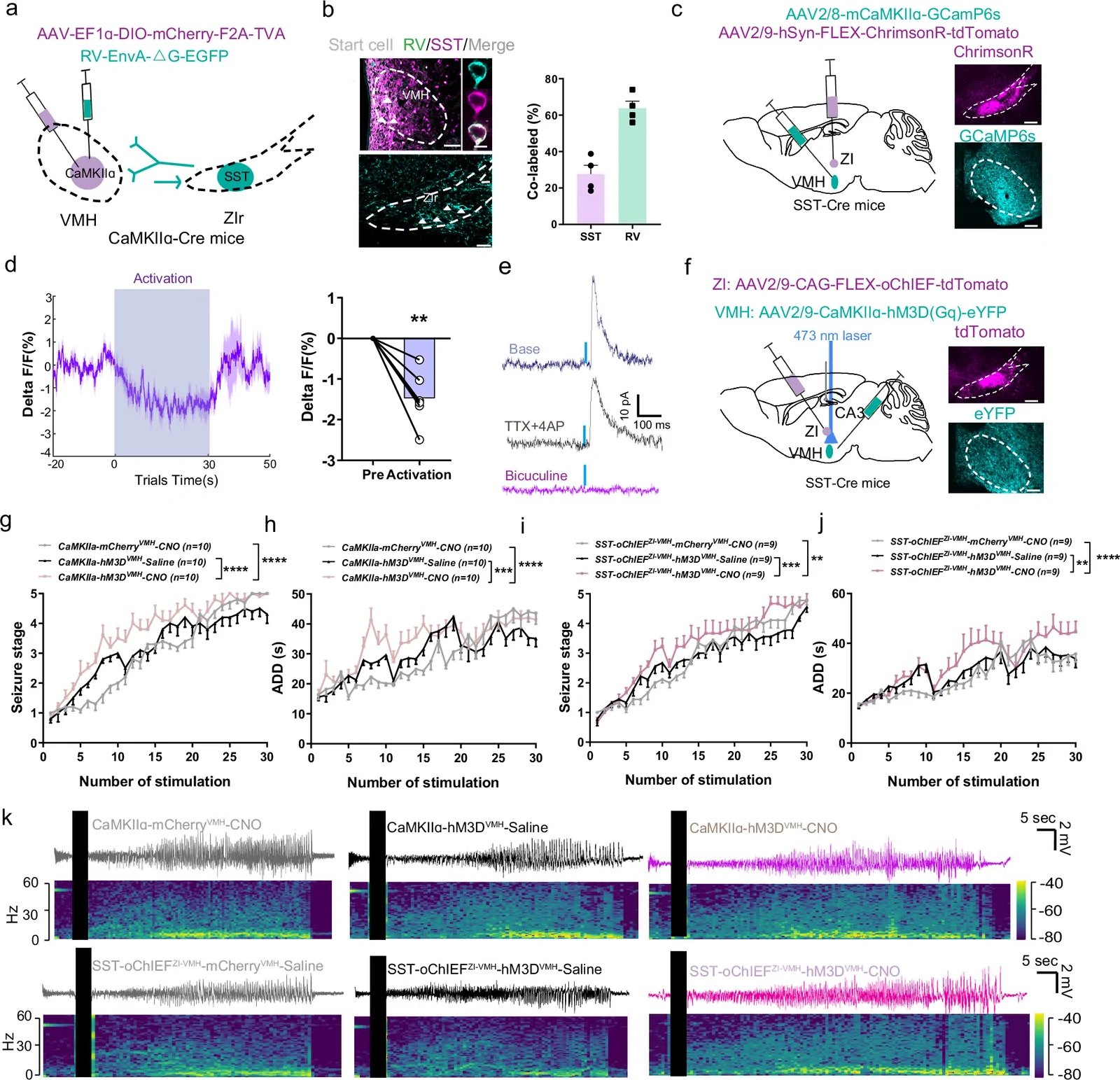
VMH glutamatergic neurons mediate anti-seizure effects of ZI-VMH^SST^ circuit. **a** Schematic of retrograde monosynaptic rabies tracing in *CaMKIIɑ-Cre* mice. **b** Left: Representative images of VMH injected with the helper virus and rabies virus. The double-infected (gloss white) glutamatergic cells represent the starter cells. Scale bar = 200 μm. Higher-magnification images showing SST^+^ GABAergic neurons infected by the rabies virus (EGFP). Scale bar = 20 μm. Right: Quantification of rabies-labelled SST neurons in the VMH. *n* = 4 mice. **c** Schematic of viral injection, fiber implantation and photo-stimulation in *SST-GCaMP*^*VMH*^*-ChrismsonR*^*ZI*^ mice, with representative ZI and VMH histological images. Scale bar = 200 μm. **d** Left: Representative calcium activities of VMH glutamatergic neurons during activation of ZI^SST^-VMH circuit. Right: group data of trials showing quantification of the average *∆F/F* of during activation of ZI^SST^-VMH circuit. *n* = 6 mice. **e** Optogenetic pulse-laser-evoked IPSC recorded from the clamped neuron persisted after TTX and 4-AP treatment, confirming monosynaptic GABAergic input, and was abolished by the GABA_A_ receptor antagonist bicuculline. **f** Left: Schematic of virus injection and optical fiber implantation in *SST-oChIEF*^*ZI*^*-hM3D*^*VMH*^ mice; Right: Representative images of the ZI and VMH from a *SST-oChIEF*^*ZI*^*-hM3D*^*VMH*^ mouse. Scale bar = 200 μm. Effects of opto-activation of ZI^SST^-VMH circuit and pharmacogenetic activation of VMH glutamatergic neurons on the seizure stage (**g**, **i**) and ADD (**h**, **j**). *n* = 10 mice (CaMKIIα-hM3D group), *n* = 9 mice (SST-oChIEF-hM3D group). **k** Representative EEGs and power spectrogram during seizures. Statistical analyses: Two-tailed paired Student’s *t*-test for (**d**); Two-way ANOVA for repeated measures followed by Tukey’s multiple comparisons test for (**g**−**j**). Significance levels were presented as **P < 0.01, ^***^P < 0.001, or ^****^P < 0.0001. Data are presented as means ± SEM. Exact *P* values are provided in Supplementary Data 1. Source data are provided as a Source Data file. (ZIr rostral zona incerta, CA3 hippocampal CA3, SST somatostatin, ADD after-discharge duration, VMH ventromedial hypothalamus, CNO clozapine-N-oxide).

To identify how ZI SST^+^ neurons innervate VMH neurons and thus participate in hippocampal seizures, we examined the cell types within the VMH that receive ZI SST^+^ synaptic inputs. The VMH is known to be mainly composed of glutamatergic neurons^29,30^. To test the structural connection between ZI SST^+^ neurons and VMH glutamatergic neurons, we performed monosynaptic viral tracing by injecting AAV-EF1a-DIO-mCherry-F2A-TVA and EGFP-expressing rabies virus (RV-EnvA-ΔG EGFP, RV) in the VMH in *CaMKIIα-Cre* mice (Fig. 6a). After 4 weeks, the mCherry/GFP co-labeled cells in the VMH represented the starter cells, and trans-synaptic labeling of green fluorescent RV-infected cells were found in the ipsilateral ZI of *CaMKIIa-Cre* mice, and 63.64% of retrogradely labeled neurons in the ZI were SST^+^ neurons (Fig. 6b). As the VMH glutamatergic neurons mainly express VGLUT1 and VGLUT2^31^, we further verified the specificity of our labeling. Using viruses driven by the CaMKII promoter to label glutamatergic neurons in the VMH, we performed co-immunostaining for VGLUT1 and VGLUT2. Immunofluorescence results showed that 95.66% of CaMKII^+^ neurons simultaneously co-expressed VGLUT1, and 76.24% of CaMKII^+^ neurons co-expressed VGLUT2 (Supplementary Fig. 15). These structural connections evidence indicate that ZI SST^+^ GABAergic neurons innervate the downstream VMH glutamatergic neurons.

To test the functional connection of the projection from ZI SST^+^ GABAergic neurons to VMH glutamatergic neurons, we injected AAV2/9-hSyn-FLEX-ChrimsonR-tdTomato into the ZI and AAV2/8-mCaMKIIα-GCaMP6s into the VMH to record the activity of VMH glutamatergic neurons during photo-activation of the ZI^SST^-VMH circuit (Fig. 6c). The fiber photometry results indicated that photo-activation of the ZI^SST^-VMH circuit significantly decreased the activity of VMH glutamatergic neurons (Fig. 6d). For further electrophysiological validation, we injected AAV2/9-DIO-ChR2-EYFP virus into the ZIr and AAV2/9-CaMKIIα-mCherry virus into the VMH of *SST-Cre* mice and prepared brain slices for whole-cell recording. The results showed that optogenetic pulse-laser-evoked inhibitory postsynaptic currents (IPSCs) recorded from the VMH glutamatergic neurons, which is persisted after TTX and 4-AP treatment but were abolished by the GABA_A_ receptor antagonist bicuculline, confirming a monosynaptic GABAergic connection (Fig. 6e). These data suggest that ZI SST^+^ neurons directly project to and functionally inhibit VMH glutamatergic neurons.

To test whether VMH glutamatergic neurons mediate the seizure-regulatory functions of ZI^SST^-VMH circuit, we first activated the VMH glutamatergic neurons by chemogenetic modulation. The results showed that activation of VMH glutamatergic neurons significantly accelerated the progression of seizure stage and prolonged ADD (Fig. 6f−h, k). Then, we simultaneously activated the ZI^SST^-VMH circuit by optogenetics while activating VMH glutamatergic neurons by intraperitoneal injection of CNO to evaluate whether VMH glutamatergic neurons mediated the anti-seizure effects of ZI^SST^-VMH circuit (Fig. 6f). We found that chemogenetic activation of VMH glutamatergic neurons fully reversed the anti-seizure effects induced by ZI^SST^-VMH circuit photo-activation (Fig. 6i−k). To exclude potential CNO off-target effects, a CNO-only control experiment was conducted in mice lacking DREADD receptor expression. CNO administration at the same dosage showed no significant impact on seizure progression (Fig. 6f−k). Furthermore, we performed additional experiments in which AAV2/9-CAG-DIO-taCaspase3-TEVp virus was injected into the ZI to ablate SST^+^ neurons, whereas AAV2/9-hEF1α-hM4D(Gi)-mCherry virus was delivered to the VMH to inhibit glutamatergic neurons. Our results showed that following ZI SST^+^ neuronal ablation, chemogenetic inhibition of VMH glutamatergic activity significantly attenuated seizure progression (Supplementary Fig. 16). This key observation strongly suggests that the role of the VMH in facilitating seizures may not be solely dependent on the ZI, as the VMH retains its independent pro-seizure function even after complete ablation of ZI SST^+^ innervation. Finally, we selectively inhibited ZI SST^+^ neurons with chemogenetics and monitored calcium activity of VMH glutamatergic neurons during kindling-induced hippocampal seizures. In the saline control group, VMH glutamatergic neurons exhibited elevated calcium activity during hippocampal seizures. CNO-mediated chemogenetic inhibition of ZI SST^+^ neurons, further potentiated the calcium activity of VMH glutamatergic neurons (Supplementary Fig. 17). These findings indicate that the hippocampal seizure-promoting effect induced by ZI SST^+^ neurons inhibition is attributable to the excessive activation of VMH glutamatergic neurons. Therefore, these evidences demonstrate that VMH glutamatergic neurons act as the critical downstream effector of the ZI^SST^-VMH circuit for modulating seizure progression.

The VMH sends projections to several key regions, including the piriform cortex (Pir), dorsomedial hypothalamus (DM), central amygdala (CeA), and paraventricular hypothalamic nucleus (PVN) (Allen Brain Atlas: https://connectivity.brain-map.org/)^32,33^. To investigate whether these downstream targets are functionally involved in seizures, we mapped these downstream targets via c-Fos-based functional neuroanatomy. Therefore, we addressed the change of c-Fos expression after activation of the soma of ZI SST^+^ neurons, and found that a dominant change in c-Fos levels in the VMH downstream nuclei, including the Pir, and DM (Supplementary Fig. 18). Notably, the Pir is considered as an ictogenic trigger and seizure spread zone in the TLE^34^, implying that the Pir might serve as the effector of the anti-seizure effect of ZI^SST^-VMH circuit. Together, the above data indicate that the ZI^SST^-VMH circuit modulates seizure progression by these downstream seizure-spreading nuclei.

## Discussions

GABAergic signaling exerts a critical homeostatic influence on epileptic seizures, yet its function varies across brain regions and neuronal subtypes^35^. Dissecting region- and circuit-specific mechanisms is essential to elucidate seizure pathogenesis. As a brain nucleus densely populated with GABAergic neurons, ZI has been increasingly implicated in seizure activity. For example, low-threshold electrical stimulation (5−50 µA) at the ZI can trigger tonic-clonic seizures^36^, suggesting that the ZI may serve as a critical nucleus involved in seizures. We previously demonstrated that deep brain stimulation of the ZI alleviates the severity of seizures through the enhancement of GABAergic neuronal activity^23^, while the precise underlying mechanisms remain elusive. Our earlier work did not distinguish GABAergic neuron subtypes, and thus, we could not exclude whether deep brain stimulation acts by enhancing the function of specific subpopulations. Extending our previous work, we delineated a cell-type- and projection-defined circuit mechanism that positions the ZI as a key regulator of epileptic activity, which will help lay the foundation for precise neuromodulation in the future.

Firstly, we identified that ZI GABAergic neurons are robustly recruited during seizure progression. Given that the ZI does not receive direct hippocampal inputs, we further examined whether its recruitment during seizures was mediated by upstream structures. The superior colliculus (SC) as a key node, as it receives broad inputs and projects densely to the ZI^37,38^. In our model, we observed that kindling-induced seizure activated SC neurons, accompanied by concurrent increases in the activity of CA3, ZI and VMH (Supplementary Fig. 19a−g), reflecting seizure signals propagated rapidly across the network. Selective ablation of SC neurons significantly attenuated hippocampal seizure-induced activation in the ZI (Supplementary Fig. 19h−k), suggesting that the SC acts as a critical upstream relay, receiving hippocampal seizure signals to the ZI. Functionally, our results reveal that optogenetic activation of ZI GABAergic neurons alleviated seizure progression, whereas their inhibition promoted it, with this bidirectional regulatory effect being mainly reflected in the later phase of GS spread. Our results are consistent with previous pharmacological modulation studies in investigating the functional role of ZI in seizures. Microinjection of GABA_A_ receptor agonists into the ZI exhibits pro-convulsant effects, while administration of antagonists demonstrates anti-convulsant properties^22^. Nevertheless, contradictory findings have been reported. For instance, administration of cholinergic agonists in the ZI can trigger generalized epileptic seizures^20^. These discrepancies may stem from the functional and mechanistic diversity among different GABAergic neuronal subtypes or subregion-specific GABAergic populations within the ZI.

The ZI harbors remarkable cellular heterogeneity among GABAergic neurons, which are primarily composed of PV^+^ and SST^+^ GABAergic neurons. Our study has elucidated the functional heterogeneity of ZI SST^+^ and PV^+^ GABAergic neurons during hippocampal seizures. Although both SST^+^ and PV^+^ GABAergic neurons were significantly activated during seizures, their intrinsic dynamics exhibit distinct patterns. During GS seizures, PV^+^ GABAergic neurons displayed an immediate and sustained slow rise in calcium signals, whereas SST^+^ GABAergic neurons exhibited a delayed but rapid increase. Moreover, these two subtypes also exhibited distinct functional contributions to seizure progression. Specifically, activation of either SST^+^ or PV^+^ GABAergic neurons significantly delayed seizure progression, particularly during the spreading stage of the seizures, with SST^+^ GABAergic neurons exhibiting more prominent anti-seizure efficacy. Notably, the activation of ZI PV^+^ and SST^+^ neurons during seizure likely reflects an endogenous anti-seizure mechanism that is engaged but insufficient to terminate seizures. As support, optogenetic activation of ZI PV^+^ and SST^+^ neurons delayed but failed to fully abort seizure development, indicating that the ZI acts as a “regulator” of seizure development. In the hippocampal kindling model, repetitive electrical stimulation chronically enhances seizure susceptibility. This ‘auxiliary inhibitory’ role likely reflects a compensatory mechanism, operating alongside other parallel inhibitory systems that are recruited to finally stop seizure^39^, whereas ZI activity alone is insufficient to prevent seizure progression. Furthermore, we also tested the anti-seizure effects under 10 Hz, 20 Hz, and 40 Hz optogenetic stimulation. PV^+^ neurons inhibited seizure progression across all frequencies, consistent with their intrinsic high-frequency firing characteristics^40^, particularly their suppression of after-discharges was weakened at 40 Hz. In contrast, SST^+^ neurons exerted anti-seizure effects only under 10 Hz and 20 Hz stimulation, while 40 Hz stimulation failed to inhibit seizures, in accordance with their inherent low-frequency discharge characteristic^41,42^. These findings highlight that the anti-seizure efficacy of ZI neurons relies on the compatibility between stimulation frequency and cell-type-specific electrophysiological properties, rather than on neuronal activation alone. Of note, the frequency-dependent anti-seizure profiles of ZI PV^+^ and SST^+^ neurons can be partially interpreted from their inherent coupling to low-frequency oscillatory networks. Our previous work demonstrated that low-frequency electrical stimulation (1 Hz and 5 Hz) of the ZI alleviates seizures by activating local GABAergic neurons^23^. In addition, activation of ventral ZI Lhx6^+^ GABAergic neurons, which largely overlap with the SST^+^ subtype, promotes NREM slow-wave sleep, a physiological state primarily characterized by delta-band oscillations (0.5−4 Hz)^43^. GABAergic activation modulates the overall excitability of slow-rhythm circuits, thereby directly suppressing delta oscillatory activity. Therefore, optogenetic activation of ZI PV^+^ and SST^+^ neurons preferentially alters delta-band EEG signals by exerting robust inhibitory control over downstream slow-rhythm networks, including thalamocortical circuits that generate slow-wave delta oscillations^43^. Although GABAergic neurons are classically tied to gamma activity in many other regions^44,45^, our findings suggest that GABAergic neuron-dependent rhythmic regulation exhibits brain-region specificity. In addition, previous studies have reported that the PV^+^ neurons form direct monosynaptic connections with SST^+^ neurons in the cortex^45–47^. We hypothesized that the PV^+^ neurons may directly innervate SST^+^ neurons within ZI. Our monosynaptic retrograde rabies tracing indicated that only 17.93% of ZIv PV^+^ neurons form direct monosynaptic innervations onto ZIr SST^+^ neurons (Supplementary Fig. 20), demonstrating the complex local disinhibitory circuits. As activation of either PV or SST is anti-seizure, and these PV-SST disinhibitory circuits are not dense, we guess that the anti-seizure effect of PV^+^ neurons may be mediated by other downstream circuits. Furthermore, ZI GABAergic neuron subtypes exhibit distinct anatomical distributions: PV^+^ neurons are predominantly located in the ZIv, whereas SST^+^ neurons are mainly distributed in the ZIr^48^, suggesting potential functional diversity in seizure modulation. Functional experiments further confirmed that selective ablation or inhibition of SST^+^ neurons significantly accelerated seizure progression, especially during the spreading stage of the seizures, whereas manipulation of PV^+^ neurons showed no significant impact on seizure progression. This functional divergence is primarily attributable to their differential efferent connectivity patterns. Anterograde tracing demonstrated that ZI SST^+^ neurons predominantly project to anterior brain regions, such as the VMH, hippocampus, anteroventral thalamic nucleus, etc., which are involved in integrating cognitive, emotional and homeostatic centers^49–51^. In contrast, ZI PV^+^ neurons primarily innervate posterior areas, such as the reticular nucleus of the thalamus and the reticular structure of the brainstem (Supplementary Fig. 21), which are implicated in sensory, motor and arousal centers. This fundamental difference in output connectivity provides an anatomical substrate for their distinct functional roles. Although the exact circuit mechanism underlying ZI PV^+^ neuron-mediated seizure modulation remains to be fully clarified, our study reveals the pivotal role of the VMH in mediating the anti-seizure effect of ZI SST^+^ neuron. In addition, c-Fos staining results further indicated that SST^+^ neurons are preferentially recruited during seizures. Collectively, these data provide evidenc to explain the different roles of PV^+^ and SST^+^ neurons in hippocampal seizures; SST^+^ GABAergic neurons predominantly mediate the primary GABAergic control of seizures within the ZI.

Similarly, functional differences between SST^+^ and PV^+^ neurons have also been observed in other neural circuits^52,53^. Recent studies have further revealed a more complex scenario, indicating that GABAergic neuronal activation can paradoxically contribute to seizure generation under certain conditions. For instance, selective opto-activation of either PV^+^ or SST^+^ neurons has been reported to promote seizures^54^. Furthermore, optogenetic or pharmacogenetic activation of PV^+^ GABAergic neurons promotes seizures via the substantia nigra reticulos-parafascicular circuit^55^. The research by Yekhlef et al. and Chen et al. primarily focused on SST^+^ and PV^+^ interneurons in the entorhinal cortex or substantia nigra^54,55^, whereas our research focused on the ZI. The PV^+^ or SST^+^ neurons in the entorhinal cortex that are activated trigger a large amount of GABA release, causing depolarization of thepyramidal neurons and a significant increase in extracellular K^+^ concentration. This further depolarizes the surrounding neurons, forming a positive feedback loop, ultimately leading to synchronized neuronal bursting and epileptiform discharges. The PV^+^ neurons in the substantia nigra project to the GABAergic neurons in the downstream PF, inhibiting their functions and thereby promoting the synchronization and progression of the epileptic network. However, the mechanism by which the activated ZI SST^+^ neurons in our study exert anti-seizure effects is through projecting to the VMH and inhibiting glutamatergic neurons, thereby suppressing the epileptic network. These findings collectively demonstrate the remarkable complexity and functional diversity of GABAergic subtypes in seizures, which may be attributed to multiple factors, including region-specific characteristics, circuit-dependent mechanisms, and the intrinsic diversity of GABAergic neuronal subtypes across different brain areas. Consequently, systematic investigation of cell-type-specific functions of GABAergic neurons across distinct brain regions in seizures, coupled with elucidation of their underlying neural circuit mechanisms, will provide a crucial theoretical framework for advancing our understanding of the complex pathogenesis of epilepsy.

Beyond seizure modulation, ZI GABAergic neuronal subtypes also exhibit distinct functional diversity in regulating other behaviors^48^. Functionally, PV^+^ GABAergic neurons have been implicated in defensive behaviors^56^ and pain/itch modulation^57^, while SST^+^ GABAergic neurons play crucial roles in defensive responses^11^ and anxiety-related behaviors^58^. Notably, ZI SST^+^ neurons send extensive projections to multiple thalamic nuclei, the amygdala, and other regions, playing crucial roles in diverse physiological functions^11,59^. The functional significance of these projections is further highlighted by their involvement in complex neural circuits. Future work could further trace their upstream inputs to map the complete ZI circuit, and in combination with single-cell sequencing, identify key neuronal markers in the ZI through which SST^+^ and PV^+^ neurons regulate specific behaviors.

Our study reveals circuit-specific mechanisms of ZI SST^+^ GABAergic neurons in hippocampal seizure modulation, showing the ZI^SST^-VMH, but not ZI^SST^-AHN or ZI^SST^-Rt, emerging as the predominant anti-seizure pathway, highlighting the complex network underlying seizure control mechanisms. Notably, we speculate that the VMH may not be exclusively under inhibitory control of ZI SST^+^ neurons; it may receive strong direct excitatory innervation from the hippocampal CA3 and other epileptogenic regions. This explains the nearly synchronous activation of ZI, VMH, SC and CA3 in kindling models (Supplementary Fig. 19). Although the ZI serves as an upstream regulatory node, GCaMP6 signals in the VMH, AHN and Rt occur faster than those in ZI SST^+^ neurons. This delayed signal observed in ZI SST^+^ neurons could be explained by two possibilities: (1) local inhibitory feedback microcircuits exist within the ZI, limiting the quick increase in Ca^2+^ level. (2) A more rapid direct pathway, such as the direct hippocampal-VMH/AHN/Rt circuit, may account for the earlier activation in these regions compared to ZI SST^+^ neurons^60^. Nevertheless, we demonstrate a causal role for the ZI-VMH circuit in modulating seizure activity. For the functional diversity at the circuitry level, our retrograde tracing results revealed that the neurons projected to AHN are mainly from both ZIv and ZIr, while VMH-projecting SST neurons are concentrated in ZIr. This structural basis is particularly relevant given the VMH’s established role as a hub for seizure progression between various forebrain and brainstem systems^61^, where both c-Fos expression and glutamatergic neuronal activity were consistently enhanced during seizure progression^30,61,62^, highlighting the contribution of VMH glutamatergic neurons in seizures. Further chemogenetic experiments confirmed that ZI SST^+^ GABAergic neurons exert anti-seizure effects primarily through inhibiting the excitability of VMH glutamatergic neurons, highlighting the important role of ZI^SST^-VMH circuit. Specifically, optogenetic activation of the ZI^SST^-VMH circuit inhibits VMH glutamatergic neuronal activity and significantly delays seizure progression, establishing a functional inhibitory connection underlying the anti-seizure circuit. Conversely, direct activation of VMH glutamatergic neurons is sufficient to promote seizure activity and abolish the protective effect induced by ZI^SST^-VMH circuit activation. Together, these findings provide convergent evidence that ZI SST+ neurons-mediated inhibition of VMH glutamatergic neuronal activity serves as a core mechanism for hippocampal seizure suppression.

Interestingly, our data demonstrate that anti-seizure effects driven by activation of ZI SST^+^ GABAergic neurons were associated with significant c-Fos induction in brain regions that do not receive direct ZI SST^+^ neuronal inputs (e.g., Pir, neocortex, medial preoptic nucleus, lateral hypothalamic area). This implies that ZI SST^+^ GABAergic neurons may engage polysynaptic pathways-possibly via intermediary nuclei-to indirectly modulate activity in these distal regions and elicit c-Fos expression. Given the VMH’s established role in integrating autonomic and limbic signals, it could serve as a critical relay hub, receiving inhibitory input from ZI SST^+^ GABAergic neurons and subsequently exciting downstream targets such as the Pir, DM, CeA or PVN (Allen Brain Atlas: https://connectivity.brain-map.org/)^32,33^. The Pir is one of the most epileptogenic and densely interconnected limbic structure. It is strongly activated during the hippocampal kindling-induced seizure^63^, and low-frequency electrical stimulation of the ipsilateral Pir inhibit seizure progression^64^. Therefore, the ZI^SST^-VMH circuit mediates seizure inhibition primarily by inhibiting VMH glutamatergic neuronal activity and excitability of downstream regions including the Pir^33^. This multi-nodal, network-level modulation highlights the role of ZI not merely as a standalone regulator but as a dynamic participant in distributed circuits that coordinates seizure suppression.

In conclusion, our study establishes the cell-type-specific regulatory framework for ZI GABAergic neurons in seizure progression. Specifically, ZI SST^+^ GABAergic projections to the VMH exert robust anti-seizure effects, especially the spread of GS during later phase by inhibiting the excitability of VMH glutamatergic neurons. These findings provide a theoretical foundation for anti-seizure circuit and identify ZI SST^+^ GABAergic neurons as potential therapeutic targets.

## Methods

### Animals

The C57BL/6 J (Wild-type), *Vgat-Cre* (Jax mice stock number: 016962), *PV-Cre* (Jax mice stock number: 008069), *SST-Cre* (Jax mice stock number: 013044), and *CaMKIIα-Cre* (Jax mice stock number: 005539) mice were used and genotyped according to the protocols provided by Jackson Laboratory. All of the mice used in this study were healthy mice (male and female, 2−4 months) with normal immune status. All mice were housed in a 12 h light/12 h dark cycle (lights on from 8:00 to 20:00) at the temperature of 22 ± 2 °C, humidity 55 ± 5% and with ad libitum food and water. Mice were individually housed after surgery to facilitate recovery and meanwhile reducing the failure rate of fiber/electrode implantation. All behavior experiments were conducted between 9:00 and 18:00, approved by the Zhejiang Chinese Medical University Animal Experimentation Committee (No.20230424-06) and were in complete compliance with the National Institutes of Health Guide for the Care and Use of Laboratory Animal and followed the ARRIVE guidelines.

### Virus vectors

For calcium photometry, AAV2/9-hSyn-DIO-GCaMP6m (viral titers: 1.36 × 10^13^ particles/mL) were stereotactically injected into the ZI of *Vgat-Cre*, *PV-Cre* and *SST-Cre* mice; AAV2/9-mDlx-GCaMP6m (viral titers: 1.54 × 10^13^ particles/mL) was stereotactically injected into the SC of *SST-Cre* mice; AAV2/8-mCaMKIIα-GCaMP6m (viral titers: 2.82 × 10^12^ particles/mL) was stereotactically injected into the VMH and CA3 of *SST-Cre* mice. AAV2/9-EF1α-DIO-Axon-GCaMP6s (viral titers: 5.09 × 10^12^ particles/mL) and AAV2/8-CaMKIIα-GCaMP6s (viral titers: 2.82 × 10^12^ particles/mL) were stereotactically injected into the ZI and VMH of *SST-Cre* and wild type mice, respectively. For optogenetic activation of GABAergic neurons or projection terminals of SST^+^ neurons, AAV2/9-CAG-FLEX-oChIEF-tdTomato (viral titers: 3.02 × 10^12^ particles/mL) was stereotactically injected into the ZI of *Vgat-Cre* and *SST-Cre* mice, respectively. For optogenetic activation of PV^+^ and SST^+^ GABAergic neurons, AAV2/9-hEF1α-DIO-hChR2(H134R)-EGFP (viral titers: 2.06 × 10^12^ particles/mL) and AAV2/9-CAG-FLEX-oChIEF-tdTomato (viral titers: 3.02 × 10^12^ particles/mL) were stereotactically injected into the ZI of *PV-Cre* and *SST-Cre* mice, respectively. For optogenetic inactivation of GABAergic neurons and their subtypes PV^+^ and SST^+^ neurons, AAV2/9-CAG-FLEX-ArchT-GFP (viral titers: 1.29 × 10^13^ particles/mL) was stereotactically injected into the ZI of *Vgat-Cre*, *PV-Cre* and *SST-Cre* mice. For chemogenetic activation of VMH glutamatergic neurons, AAV2/9-CaMKIIα-hM3D(Gq)-eYFP (viral titers: 2.16 × 10^12^ particles/mL) or AAV2/9-CaMKIIα-mCherry (viral titers: 5.15 × 10^12^ particles/mL) were stereotactically injected into the VMH of *SST-Cre* mice. For chemogenetic activation or inactivation of ZI SST^+^ neurons, AAV2/9-hEF1a-DIO-hM3D(Gq)-mCherry (viral titers: 1.00 × 10^13^ particles/mL) or AAV2/9-hEF1α-DIO-hM4D(Gi)-mCherry (viral titers: 1.00 × 10^13^ particles/mL) were stereotactically injected into the ZI of *SST-Cre* mice. For selective ablation of the GABAergic neurons and their subtypes PV^+^ and SST^+^ neurons, AAV2/9-CAG-DIO-taCaspase3-TEVp (viral titers: 3.26 × 10^13^ particles/mL) was stereotactically injected into the ZI of *PV-Cre* and *SST-Cre* mice. AAV2/9-hEF1α-DIO-EYFP (viral titers: 1.46 × 10^13^ particles/mL) was used as control virus. For the retrograde tracing of ZI PV^+^ neurons to the ZI SST^+^ neurons, AAV-EF1α-DIO-EGFP-T2A-TVA (viral titers: 2.04 × 10^13^ particles/mL), AAV-EF1α-DIO-N2cG (viral titers: 5.16 × 10^12^ particles/mL) and CVS-EnvA-∆G-tdTomato (viral titers: 2.00 × 10^8^ particles/mL) were stereotactically injected into the ZIr of *SST-Cre* mice. For the retrograde tracing of ZI SST^+^ neurons to the glutamatergic neurons in the VMH, AAV-EF1α-DIO-mCherry-F2A-TVA (viral titers: 2.00 × 10^12^ particles/mL) and RV-EnvA-∆G-EGFP (viral titers: 2.00 × 10^08^ particles/mL) were stereotactically injected into the VMH. For retrograde tracing of VMH and AHN, AAV2/2Retro-hEF1α-DIO-EGFP (viral titers: 1.19 × 10^13^ particles/mL) and AAV2/2Retro-hEF1α-DIO-mCherry (viral titers: 2.82 × 10^12^ particles/mL) were stereotactically injected into the VMH and AHN of *SST-Cre* mice, respectively. Viruses were purchased from Taitool Bioscience (China), except for AAV2/9-EF1α-DIO-axon-GCaMP6s, AAV2/9-CaMKIIα-hM3D(Gq)-eYFP (China), and AAV2/9-CaMKIIα-Cre-mCherry from Brain TVA, AAV-EF1α-DIO-mCherry-F2A-TVA from Brain Case (China), and AAV2/9-CAG-FLEX-ArchT-GFP from OBio Technology (China).

### Stereotactic injection and surgeries

For virus injection, mice were anesthetized with sodium pentobarbital (60 mg/kg, i.p.) and head-fixed in a stereotaxic apparatus (Catalog No.68506, RWD Life Science, China). The viruses (200 nL) were injected into the ventral ZI (ZIv, AP: −2.2 mm; L: −1.6 mm; V: −4.3 mm), rostral ZI (ZIr, AP: −0.8 mm; L: −0.7 mm; V: −4.5 mm) and VMH (AP: −1.2 mm; L: −0.4 mm; V: −5.6 mm), SC (AP: −3.4 mm; L: −0.7 mm; V: −2.3 mm), CA3 (AP: −2.9 mm; L: −3.2 mm; V: −3.2 mm) using a 1 µL syringe at 40 nL/min by an injection pump. After injection, the syringe was left in place for an additional 5 min to allow diffusion of the viruses, and then slowly withdrawn.

For implanting electrodes or optical fiber, after 3 weeks of viral delivery, twisted-bipolar electrodes (Catalog No.795500, 0.127 mm diameter, A.M Systems, USA) were implanted in the right ventral hippocampus (CA3, AP: −2.9 mm, ML: −3.2 mm, V: −3.4 mm) for both kindling stimulation and EEG recording. The optical probes (0.2 mm diameter, Inper, China) were separately implanted above the ZIv (AP: −2.2 mm; L: −1.6 mm; V: −4.3 mm), ZIr (AP: −0.8 mm; L: −0.7 mm; V: −4.5 mm), VMH (AP: −1.2 mm; L: −0.4 mm; V: −5.6 mm), AHN (AP: −0.7 mm; L: −0.4 mm; V: −5.2 mm), Rt (A 18° diagonal turn to the right, AP: −0.45 mm; L: −2.5 mm; V: −3.2 mm), and SC (AP: −3.4 mm; L: −0.7 mm; V: −2.3 mm) allowing for light stimulation. The coordinates were measured from the bregma according to the mouse atlas^65^.

For bicuculline and CYN-154806 injection, after 3 weeks of viral delivery, cannulas (Cat No.62003, RWD Life Science, China) were implanted in the right VMH (AP: −1.2 mm; L: −0.4 mm; V: −5.6 mm) for microinjection. After the animal recovered for 1 week, using a 1-µL microliter syringe (Gaoge Industrial and Trading Co. LTD, Shanghai, China) at a speed of 50 nL/min with an injection pump (Micro 4, World Precision Instruments, USA), bicuculline (6 ng/200 nL, 200 nL, MedChemExpress, HY-N-219)^66,67^ or CYN-154806 (12 ng/500 nL, 200 nL, MedChemExpress, HY-P1202)^68^ was injected into the VMH.

Mice were kept on a heating pad at 37 °C throughout the entire surgery procedures and returned to their home cages after post-surgery recovery. The location of electrodes and optical fiber, and viral expression, were histologically verified after the behavioral studies. Only the mice with the correct locations of electrodes and ceramic ferrule, and viral expression are taken into analysis. These criteria were pre-established.

### Fiber photometry

Fiber photometry of calcium signals was produced 7 days after electrode and ceramic ferrule implantation surgeries in mice expressing GCaMP6s. The fiber photometry system (Nanjing Thinkertech) used a 488 nm diode laser (OBIS 488LS; Coherent), reflected by a dichroic mirror (MD498, Thorlabs), and coupled into a 0.23 mm 0.37 NA optical fiber using a ×10 objective between the fiber tip and the animal, ranging from 0.01 to 0.03 mW, to minimize bleaching^25,55^. The collected GCaMP fluorescence was converted to voltage signals by a digital amplifier (C7319, Hamamatsu, Japan). The converted signals were recorded at 100 Hz for 240 s (120 s baseline and 120 s after kindling stimulation). For data analysis, we marked the start of the hippocampal-kindling event as 0 s. All subsequent fluorescence data were aligned to this reference point. Data were analyzed based on events of individual trials of kindling stimulation and derived the values of fluorescence change (*ΔF/F*) by calculating (*F* − *F0*)/*F0*, among which *F* represented the current value of the signal, *F0* represented the average value of the baseline signal between 100 and 120 s. The data were presented as a linear graph of average fluorescence and statistical plots.

### Hippocampal kindling model

After 7 days of surgery recovery, the after-discharge threshold (ADT) of each mouse was measured by a constant current stimulator (monophasic square-wave pulses, 20 Hz, 1 ms/pulse, 40 pulses) with a bipolar electrode implanted into the right hippocampal CA3, and EEG was recorded by a digital amplifier. The stimulation current started at 40 µA and was subsequently increased by 20 µA every 1 min until at least 5 s of ADD occured, which was defined as ADT. The mice were grouped reasonably according to the ADT. All mice received ten suprathreshold stimulations (monophasic square-wave pulses, 400 µA, 20 Hz, 1 ms/pulse, 40 pulses, at least 30  min-interval per stimulation) daily from the next day on. Seizure severity was scored according to the modified Racine scale^69^ as follows: (1) facial movement; (2) head nodding; (3) unilateral forelimb clonus; (4) bilateral forelimb clonus and rearing; and (5) rearing and falling. Stages 1−3 were considered as FSs and stages 4−5 were considered as GSs^70^. When mice exhibited three consecutive stage 5 seizures, they were regarded as fully kindled. The seizure stage was scored by an investigator blinded to the group allocation.

### Optogenetic and chemogenetic manipulation

Blue (473 nm) or yellow (589 nm) laser light was delivered into the targeted brain region via a 200 µm diameter optic fiber (Inper, Co., Ltd, China). The optical fiber was cut flat and the laser power was adjusted to about 5 mW measured by a power meter (THORLABS, USA). Before placing the mice in the chamber, an optical fiber was directly inserted into a ceramic ferrule. The optical fiber was secured to ensure no movement of the fiber occurred during the experiment. In the hippocampal kindling model, blue or yellow optical stimulation was delivered in real time immediately after the end of each kindling stimulation, and the optical stimulation was stopped immediately when the ADD ended. For optogenetic activation experiments, 473 nm blue light (10 Hz, 20 Hz, or 40 Hz, 10 ms/pulse, 5 mW, in real time) was used. For optogenetic inactivation experiments, 589 nm yellow light (continuous, 5 mW, in real time) was used.

For chemogenetic activation experiments, in the hippocampal kindling model, clozapine-N-oxide (CNO; 1 mg/kg) was injected intraperitoneally daily, 30 min before the first hippocampal kindling stimulation.

### Brain slice electrophysiology

Four weeks after viral delivery, mice were deeply anesthetized and transcardially perfused with ice-cold artificial cerebrospinal fluid (ACSF; in mM: 120 NaCl, 11 glucose, 2.5 KCl, 1.28 MgSO₄, 3.3 CaCl₂, 1 NaH₂PO₄, and 14.3 NaHCO₃). Brains were rapidly extracted and sectioned into 300-μm-thick coronal slices using a vibratome (VT1000S, Leica). The slices were immediately transferred to ice-cold, oxygenated ACSF (95% O₂ and 5% CO₂) for 1 min. Slices containing the VMH were then allowed to recover in continuously oxygenated ACSF at 37 °C for 30 min, followed by an additional equilibration period at 25 °C for 1 h. For electrophysiological recordings, slices were placed in a recording chamber maintained at 25 °C. Whole-cell recordings were obtained using a HEKA EPC-10 amplifier (HEKA Instruments, Germany). Light-evoked synaptic responses were recorded in a modified low-divalent ACSF composed of (in mM): 125 NaCl, 3.5 KCl, 1.25 NaH₂PO₄, 0.5 MgCl₂, 26 NaHCO₃, 25 glucose, and 1 CaCl₂. Recording electrodes were filled with a cesium-based intracellular solution containing (in mM): 100 CsCH₃SO₃, 20 KCl, 10 HEPES, 4 Mg-ATP, 0.3 Tris-GTP, 7 Tris₂-phosphocreatine, and 3 QX-314. Inhibitory postsynaptic currents (IPSCs) were recorded at a holding potential of + 10 mV. Spontaneous synaptic activity was first monitored for approximately 20 s. Optogenetic stimulation was subsequently delivered using blue light (473 nm, 10 ms/pulse) to activate axon terminals originating from the ZI. To assess monosynaptic connectivity and minimize polysynaptic contributions, tetrodotoxin (TTX, 1 μM) and 4-aminopyridine (4-AP, 50 μM) were applied following detection of light-evoked responses. Pharmacological identification of synaptic currents was further performed by bath application of bicuculline (20 μM) to block GABA_A_ receptor-mediated IPSCs. Synaptic events were detected using MiniAnalysis software (version 6.0.3).

### Immunohistochemistry and imaging

The mice were deeply anesthetized with 1% sodium pentobarbital and transcranially perfused with 0.9% saline and followed by 4% paraformaldehyde (PFA) in 0.1 M phosphate buffer (PBS). The brains were removed, immersed in 4% PFA for further fixation overnight at 4 °C, and cryoprotected them with 30% (wt/vol) sucrose. 30-µm coronal sections were cut on a sliding freezing microtome (NX70, Thermo Fisher Scientific, USA). We processed sections for immunofluorescence for primary antibodies as follows: anti-c-Fos (1:500, Abcam, Ab208942), anti-GAD67 (1:200, HUABIO, ED1703-71), anti-Parvalbumin (1:500, Abcam, Ab181086), anti-Somatostatin 28 (1:500, HUABIO, HA721460), anti-VGLUT1 (1:200, HUABIO, HA601449), anti-VGLUT2 (1:200, HUABIO, HA601451), anti-VGLUT2 (1:200, OriGene, TA322266S), anti-CaMKII (1:500, Abcam, Ab52476), and anti-nNOS (1:200, HUABIO, R1510-28), and then incubated the primary antibodies solution containing 1% Triton X-100 overnight at 4 °C, rinsed them with 0.1 M PBS. The Goat anti-mouse Alexa Fluor^®^−488 (Abcam, Ab150113), Goat anti-rabbit Alexa Fluor^®^−594 (Abcam, Ab150080) or Goat anti-rabbit Alexa Fluor^®^−647 (Abcam, Ab150079) conjugated secondary antibodies were used at 1:1000 for 2 h at room temperature. Then, they were rinsed with 0.1 M PBS and mounted on slides with media containing DAPI (Yeasen Biotech Co., Ltd, China). Images acquisition and immunofluorescence evaluation used a fluorescence microscope (DM6B, Leica, Germany).

### Statistic

Data are presented as means ± SEM. The number of experimental subjects independently processed in each experimental condition (*n*) is represented in each figure and figure legend. Statistical comparisons were performed using Prism (version 8.0) and SPSS (version 25.0) with appropriate statistical methods as indicated in figure legends. Statistical tests were preceded by a normal distribution check (Shapiro–Wilk normality test). For normally distributed data, comparisons between two groups utilized paired or unpaired two-tailed Student’s *t*-tests, comparisons between multiple groups were performed using One-way or Two-way ANOVA, with post hoc tests (Tukey’s, Dunnett’s, or Bonferroni) where applicable. Comparisons between two groups utilized Pearson linear correlation analysis. In case of non-normal data distribution, non-parametric tests were used: Mann–Whitney U test was used for single comparisons, and the Scheirer–Ray–Hare test for Two-way analysis of variance. No statistical methods were used to predetermine sample size or randomize. For all analyses, the tests were two-tailed and *P* value < 0.05 was considered statistically significant. Detailed statistical parameters are provided in Supplementary Data 1 with the paper.

### Reporting summary

Further information on research design is available in the Nature Portfolio Reporting Summary linked to this article.

## Supplementary information

Supplementary Information

Description of Additional Supplementary Files

Supplementary Data 1

Reporting Summary

Transparent Peer Review file

## Source data

Source Data

## Data Availability

The source data generated in this study are provided in the Supplementary Information/Source Data file. Additional data related with this paper is available from the corresponding author. Source data are provided with this paper.
