# Supplementary material for: Zona incerta somatostatin+ neurons modulate seizure progression through hypothalamic circuits
Source: Nat Commun. 2026 Sep 29;17:11234. doi: 10.1038/s41467-026-78174-9 (PMC13720055; doi:10.1038/s41467-026-78174-9)
Supplement: Supplementary file 1 [file 41467_2026_78174_MOESM1_ESM.pdf]

Zona Incerta somatostatin^{+} neurons modulate seizure progression through hypothalamic circuits

Mengdi Zhang[#1,2], Lin Yang[#1], Yuan Zhou[#1], Yeping Ruan[1,3], Dongxiao Jiang[1], Lilong Yu[1], Jiajia Ying[1], Wenjing Wang[1], Tianyu Tang[1], Menghan Li[1], Zhong Chen[1], Fan Fei*[1], Cenglin Xu*[1], Yi Wang*[1]

[1]Zhejiang Collaborative Innovation Center for the Brain Diseases with Integrative Medicine, Zhejiang Key Laboratory of Neuropsychopharmacology, School of Pharmaceutical Sciences, The First Affiliated Hospital of Zhejiang Chinese Medical University, Hangzhou, Zhejiang, 310058, China

[2]Medical Research Center, Shandong Medical and Pharmaceutical University Hospital, Binzhou, Shandong, 256603, China

[3]College of Pharmaceutical Sciences, Zhejiang Pharmaceutical University, Ningbo, Zhejiang, 315500, China

#These authors contributed equally to this work.

*Corresponding author:

Prof. Yi Wang, wang-yi@zju.edu.cn

Prof. Cenglin Xu, xucenglin5zz@zju.edu.cn

Prof. Fan Fei, fei-fan@zju.edu.cn

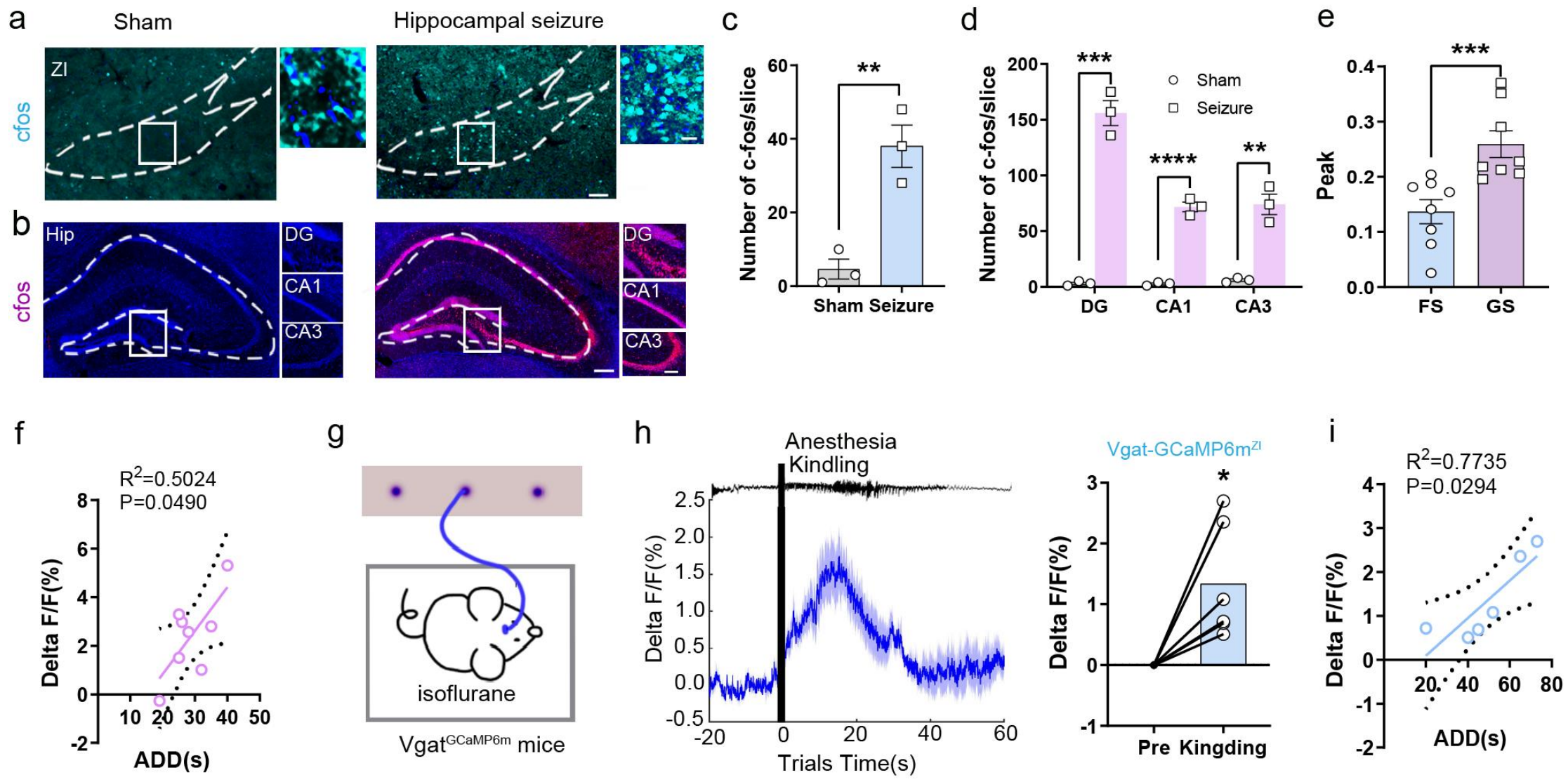

Supplementary Figure 1. ZI GABAergic neurons are gradually activated during seizure progression. (**a, b**) Representative images of c-Fos expression in the ZI (**a**) and hippocampus (**b**) in control (Sham), and kindled mice with hippocampal seizure. ZI Scale bar = 100 μm, Right: enlarged scale bar = 20 μm; hippocampus scale bar = 500 μm, Right: enlarged scale bar = 50 μm. (**c, d**) Quantification of c-Fos in the ZI, hippocampus DG, CA1, and CA3 in sham and kindled mice. n = 3 mice. (**e**) The peak calcium signals in FS and GS of ZI GABAergic neurons. n = 8 mice. (**f**) Correlation analysis between the average ΔF/F (%) and ADD of ZI GABAergic neurons in hippocampal seizures. The dotted line region indicates the 95% confidence intervals. n = 8 mice. (**g**) Scheme of experiment for optical fiber recording of *Vgat-GCaMP6m^{ZI}* mice anesthetized with isoflurane. (**h**) Left: the typical EEG and average calcium signals during kindling-induced hippocampal seizure; Right: statistic data of trials showing quantification of the average ΔF/F of ZI GABAergic neuron during hippocampal seizure. n = 6 mice. (**i**) The correlation analysis between the average ΔF/F (%) and ADD of *Vgat-GCaMP6m^{ZI}* mice anesthetized with isoflurane in hippocampal seizures. The dotted line region indicates the 95% confidence intervals. n = 6 mice. Statistical analyses: Two-tailed unpaired student's t test for (**c, d**); Two-tailed Mann-Whitney U test for (**e**); Pearson for (**f, i**); Two-tailed paired student's t test for (**h**). Significance levels were presented as *P < 0.05, **P < 0.01, ***P < 0.001, or ****P < 0.0001. Data are presented as means ± SEM. Exact P values are provided in Supplementary Data 1. Source data are provided as a Source Data file. (Abbreviations: ZI, zona incerta; DG, hippocampal DG; CA1, hippocampal CA1; CA3, hippocampal CA3; FS, focal seizure; GS, generalized seizure; ADD, after-discharge duration).

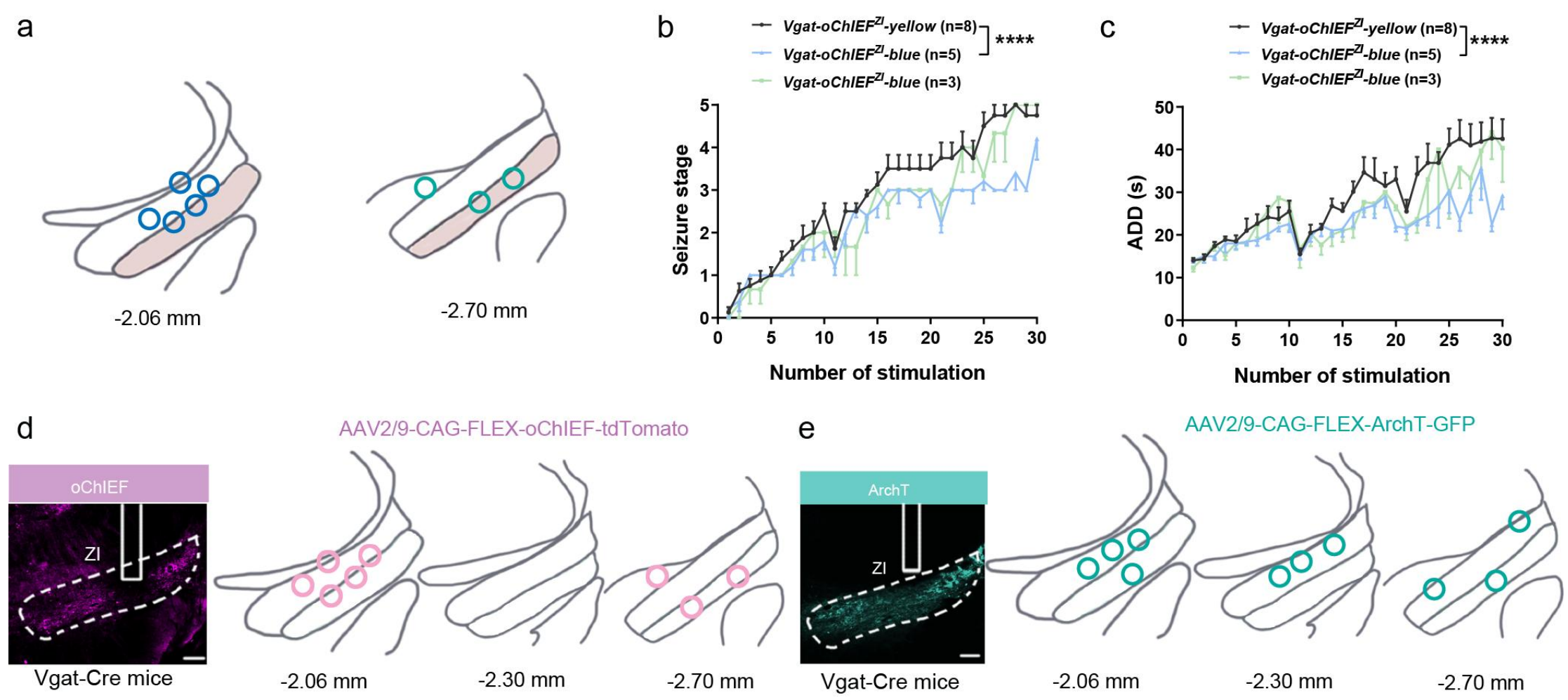

Supplementary Figure 2. Optical fiber implantation positions for *Vgat-oChIEF^{ZI}* and *Vgat-ArchT^{ZI} mice.* (**a**) Schematic diagram of the optical fiber implantation locations of optogenetic activation *Vgat-Cre mice* included in the statistics. (**b, c**) Effects of optogenetic activation ZI GABAergic neurons on the development of seizure stage (**b**), ADD, (**c**). *Vgat^{oChIEF}-yellow* mice, n = 8 mice; optical fibers targeting the anterior ZI in *Vgat^{oChIEF}-blue* mice, n = 5 mice; optical fibers targeting the edges of the ZI in *Vgat^{oChIEF}-blue* mice, n = 3 mice. Two-way ANOVA with Tukey's multiple comparison test for (**b, c**). (**d, e**) Schematic diagram of the optical fiber implantation locations of optogenetic activation (**d**) or inhibition (**e**) in *Vgat-mice* included in the statistics. Scale bar = 200 μm. *Vgat^{oChIEF}* mice, n = 8 mice; *Vgat^{ArchT}* mice, n = 10 mice. Significance levels were presented as ****P < 0.0001. Data are presented as means ± SEM. Exact P values are provided in Supplementary Data 1. Source data are provided as a Source Data file. (Abbreviations: ZI, zona incerta; ADD, after-discharge duration).

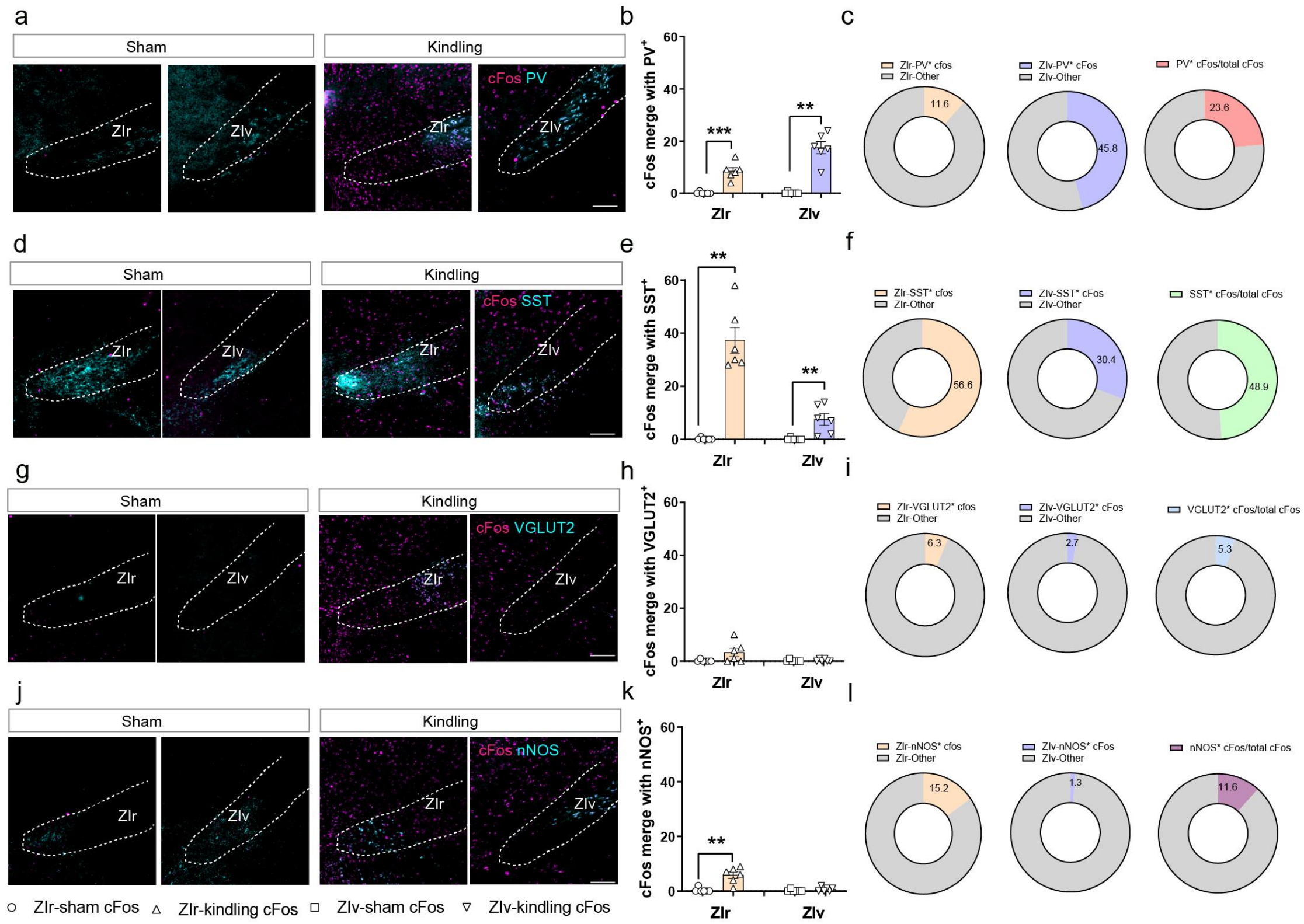

Supplementary Figure 3. The c-Fos expression of ZI neuronal subtypes during hippocampal seizure. (**a, b**) Representative images and quantification of c-Fos co-stanning with PV in the ZIr and ZIv sections during hippocampal seizure. Scale bar = 100 μm. Two-tailed unpaired student's t test for ZIr, Two-tailed Mann-Whitney U test for ZIv. (**c**) Proportion of PV^+ c-Fos in the kindling groups in ZIr and ZIv sections. (**d, e**) Representative images and quantification of c-Fos co-stanning with SST in the ZIr and ZIv sections during hippocampal seizure. Scale bar = 100 μm. Two-tailed Mann-Whitney U test. (**f**) Proportion of SST^+ c-Fos in the kindling groups in ZIr and ZIv sections. (**g, h**) Representative images and quantification of c-Fos co-stanning with VGLUT2 in the ZIr and ZIv sections during hippocampal seizure. Scale bar = 100 μm. Two-tailed Mann-Whitney U test. (**i**) Proportion of $VGLUT2^+$ c-Fos in the kindling groups in ZIr and ZIv sections. (**j, k**) Representative images and quantification of c-Fos co-stanning with nNOS in the ZIr and ZIv sections during hippocampal seizure. Scale bar = 100 μm. Two-tailed Mann-Whitney U test. (**l**) Proportion of $nNOS^+$ c-Fos in the kindling groups in ZIr and ZIv sections. n = 6 mice. Significance levels were presented as **$P < 0.01$, or ***$P < 0.001$. Data are presented as means ± SEM. Exact P values are provided in Supplementary Data 1. Source data are provided as a Source Data file. (Abbreviations: ZIr, rostral zona incerta; ZIv, ventral zona incerta; PV, parvalbumin; SST, somatostatin).

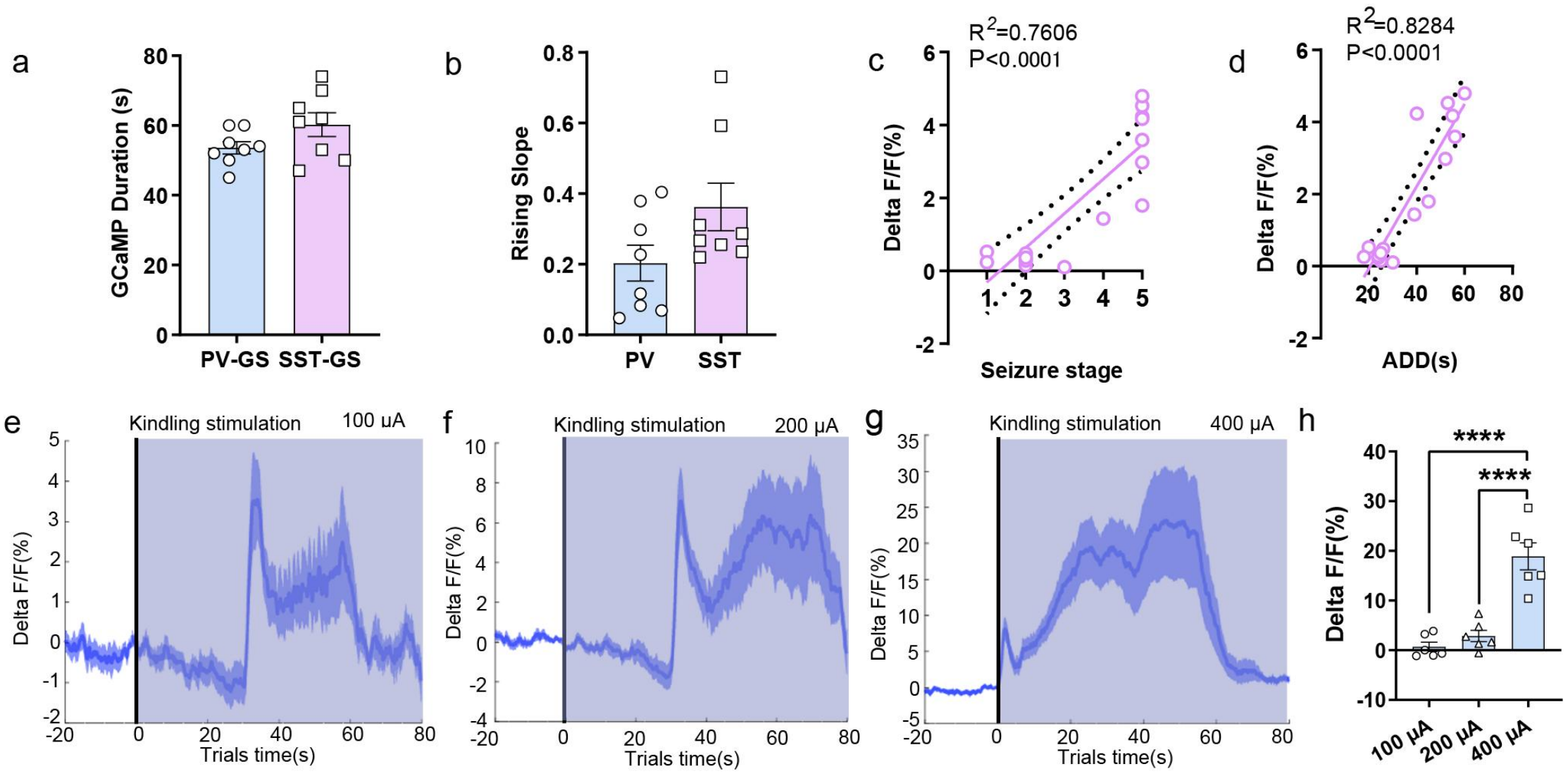

Supplementary Figure 4. A comparison of the differences in calcium signals and seizure severity between PV⁺ and SST⁺ neurons during seizures. (**a**) The duration of GCaMP signaling of PV⁺ and SST⁺ neurons during GS induced by hippocampal kindling. *PV-GCaMP6m*^{ZI} mice, n = 8 mice. S*ST-GCaMP6m*^{ZI} mice, n = 8 mice. (**b**) The ascending slope of calcium signals of ZI PV⁺ and SST⁺ neurons from hippocampal seizure to GS peak. *PV-GCaMP6m*^{ZI} mice, n = 8 mice. S*ST-GCaMP6m*^{ZI} mice, n = 8 mice. (**c**) The correlation between the average ΔF/F (%) and seizure stage in hippocampal seizures of *SST-GCaMP6m*^{ZI} mice. The dotted line region indicates the 95% confidence intervals. n = 8 mice. (**d**) The correlation between the average ΔF/F (%) and ADD in hippocampal seizures of *SST-GCaMP6m*^{ZI} mice. The dotted line region indicates the 95% confidence intervals. n = 8 mice (FS and GS originated from the same mouse). (**e-h**) The average ΔF/F and quantification of ZI SST⁺ GABAergic neuron under 100 μA (**e**), 200 μA (**f**) and 400 μA (**g**) stimulation intensity in kindling-induced seizure. n = 6 mice. Statistical analyses: Two-tailed unpaired student's t test for (**a**); Two-tailed Mann-Whitney U test for (**b**); Pearson for (**c, d**); Ordinary one-way ANOVA with Tukey's multiple comparison test for (**h**). Significance levels were presented as ****P < 0.0001. Data are presented as means ± SEM. Exact P values are provided in Supplementary Data 1. Source data are provided as a Source Data file. (Abbreviations: PV, parvalbumin; SST, somatostatin; GS, generalized seizure; ADD, after-discharge duration).

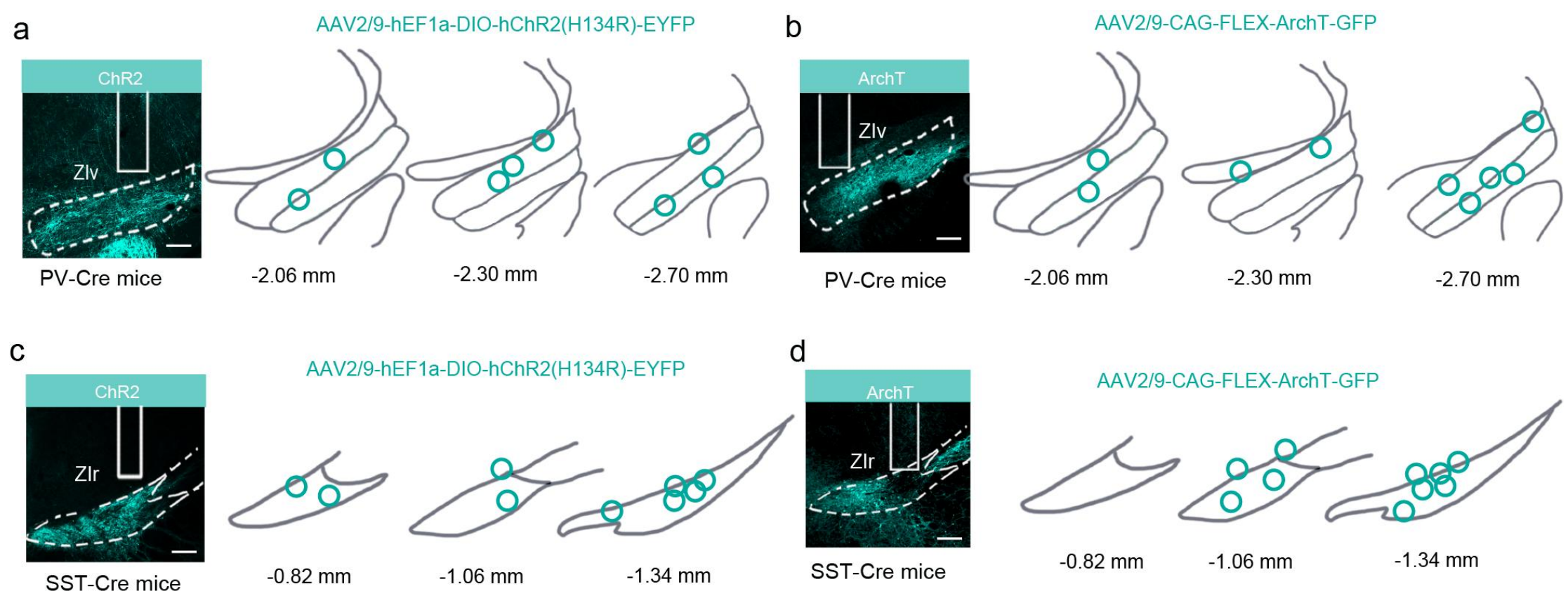

Supplementary Figure 5. Optical fiber implantation positions for *PV*, *SST-ChR2*^{ZI} and *PV*, *SST-ArchT*^{ZI} mice. (**a, b**) Schematic diagram of the optical fiber implantation locations of optogenetic activation (**a**) or inhibition (**b**) of *PV-mice* included in the statistics. Scale bar = 200 µm. (**c, d**) Schematic diagram of the optical fiber implantation locations of optogenetic activation (**c**) or inhibition (**d**) of *SST-mice* included in the statistics. Scale bar = 200 µm. PV^{ChR2} mice, n = 8 mice; PV^{ArchT} mice, n = 9 mice; SST^{ChR2} mice, n = 9 mice; SST^{ArchT} mice, n = 10 mice. (Abbreviations: ZIr, rostral zona incerta; ZIv, ventral zona incerta; PV, parvalbumin; SST, somatostatin).

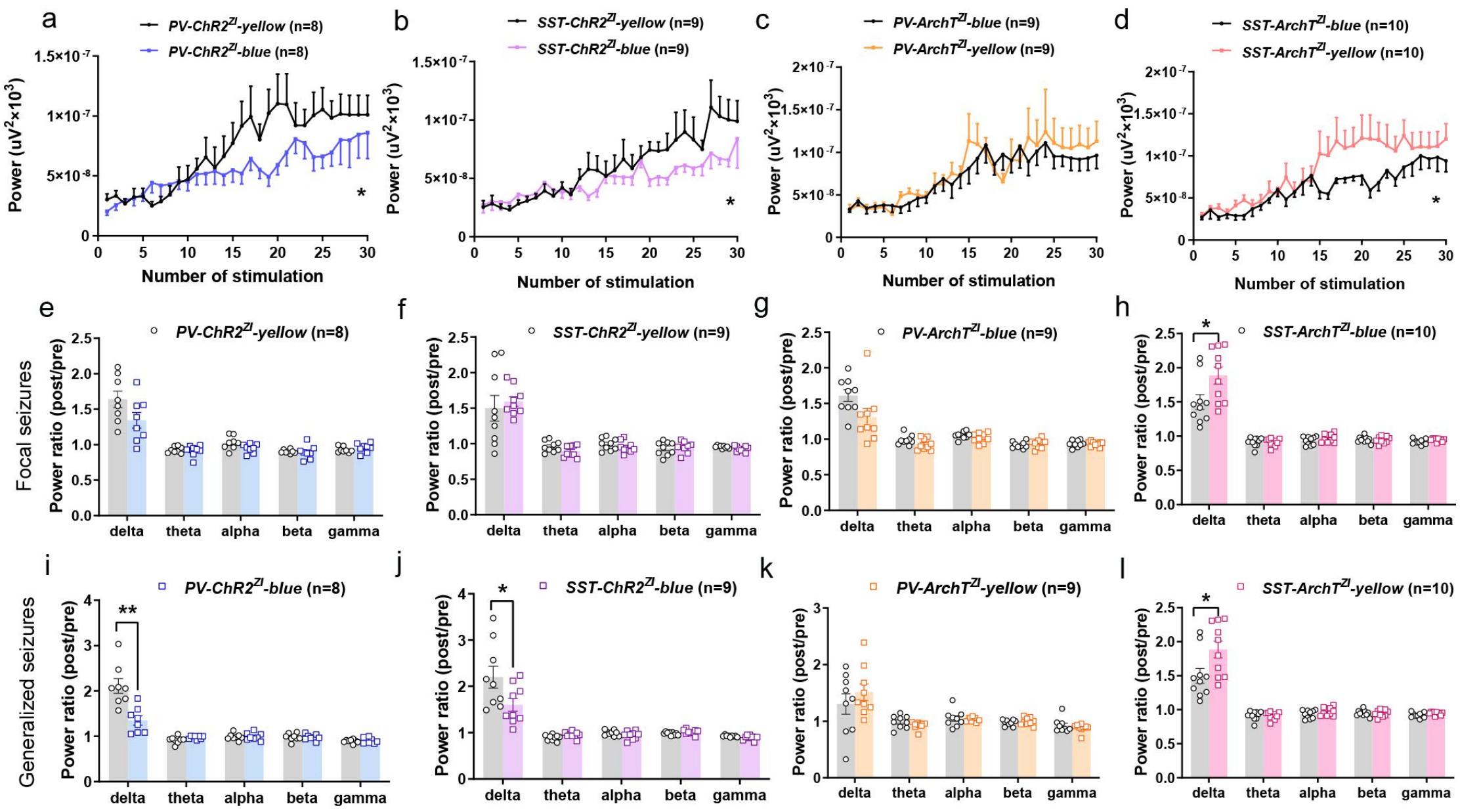

Supplementary Figure 6. Optogenetic regulation of ZI PV$^+$ and SST$^+$ GABAergic alters EEG power spectrum and delta band (**a, b**) The EEG power spectra of optogenetic activated PV and SST neurons in *PV-ChR2^{ZI}* (n = 8 mice) and *SST-ChR2^{ZI}* (n = 9 mice) mice. Two-way ANOVA for repeated measures. (**c, d**) The EEG power spectra of PV and SST neurons optogenetic inhibition (PV: n = 9 mice, SST: n = 10 mice). Two-way ANOVA for repeated measures. (**e, f, i, j**) The post/pre EEG power ratio after PV or SST optogenetic activation (PV: n = 8 mice, SST: n = 9 mice). Two-tailed unpaired student's t test or Mann Whitney U test. (**g, h, k, l**) The post/pre EEG power ratio after PV or SST optogenetic inhibition (PV: n = 9 mice, SST: n = 10 mice). Two-tailed unpaired student's t test or Mann Whitney U test. Significance levels were presented as *P < 0.05 or **P < 0.01. Data are presented as means ± SEM. Exact P values are provided in Supplementary Data 1. Source data are provided as a Source Data file. (Abbreviations: PV, parvalbumin; SST, somatostatin).

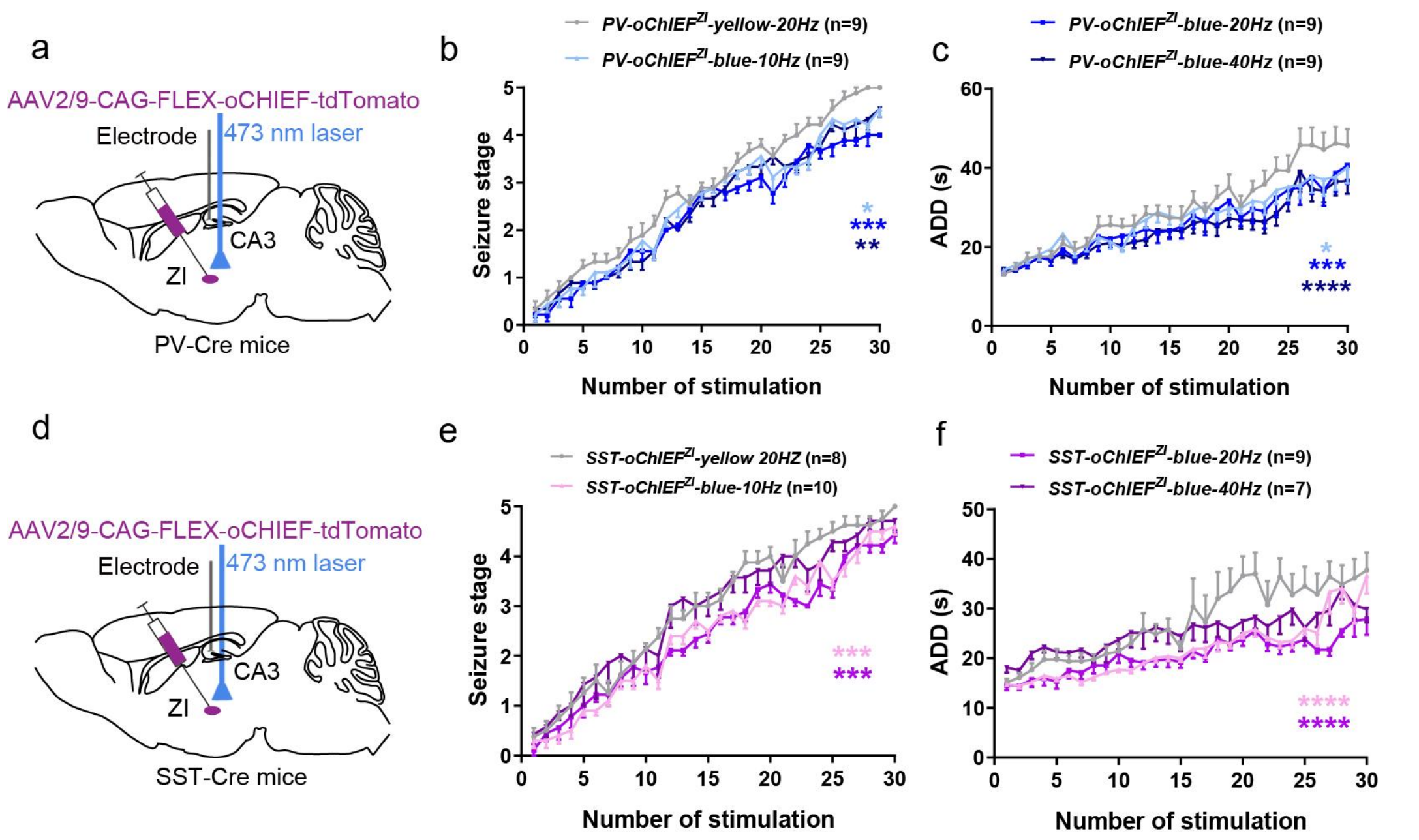

Supplementary Figure 7. Optogenetic activation of ZI PV⁺ or SST⁺ GABAergic neurons exerts divergent frequency-dependent seizure-suppressive effects. (**a**) Schematic of viral injection and photo-stimulation of ZI PV$^+$ GABAergic neuron. (**b, c**) Effects of optogenetic activation of ZI PV$^+$ GABAergic neurons in 10Hz, 20Hz, and 40Hz on the development of seizure stage (**b**), and ADD (**c**). n = 9 mice per group. (**d**) Schematic of viral injection and photo-stimulation of ZI SST$^+$ GABAergic neuron. (**e, f**) Effects of optogenetic activation of ZI SST$^+$ GABAergic neurons in 10Hz, 20Hz, and 40Hz on the development of seizure stage (**e**), and ADD (**f**). *SST-oChIEF^{ZI}-yellow-20Hz*, n = 8 mice; *SST-oChIEF^{ZI}-blue-10Hz*, n = 10 mice; *SST-oChIEF^{ZI}-blue-20Hz*, n = 9 mice; *SST-oChIEF^{ZI}-blue-40Hz*, n = 7 mice. Statistical analyses: Two-way ANOVA with Dunnett's multiple comparisons test for (**b, c, e, f**). Significance levels were presented as *P < 0.05, **P < 0.01, ***P < 0.001 or ****P < 0.0001. Data are presented as means ± SEM. Exact P values are provided in Supplementary Data 1. Source data are provided as a Source Data file. (Abbreviations: ZI, zona incerta; CA3, hippocampal CA3; PV, parvalbumin; SST, somatostatin; ADD, after-discharge duration).

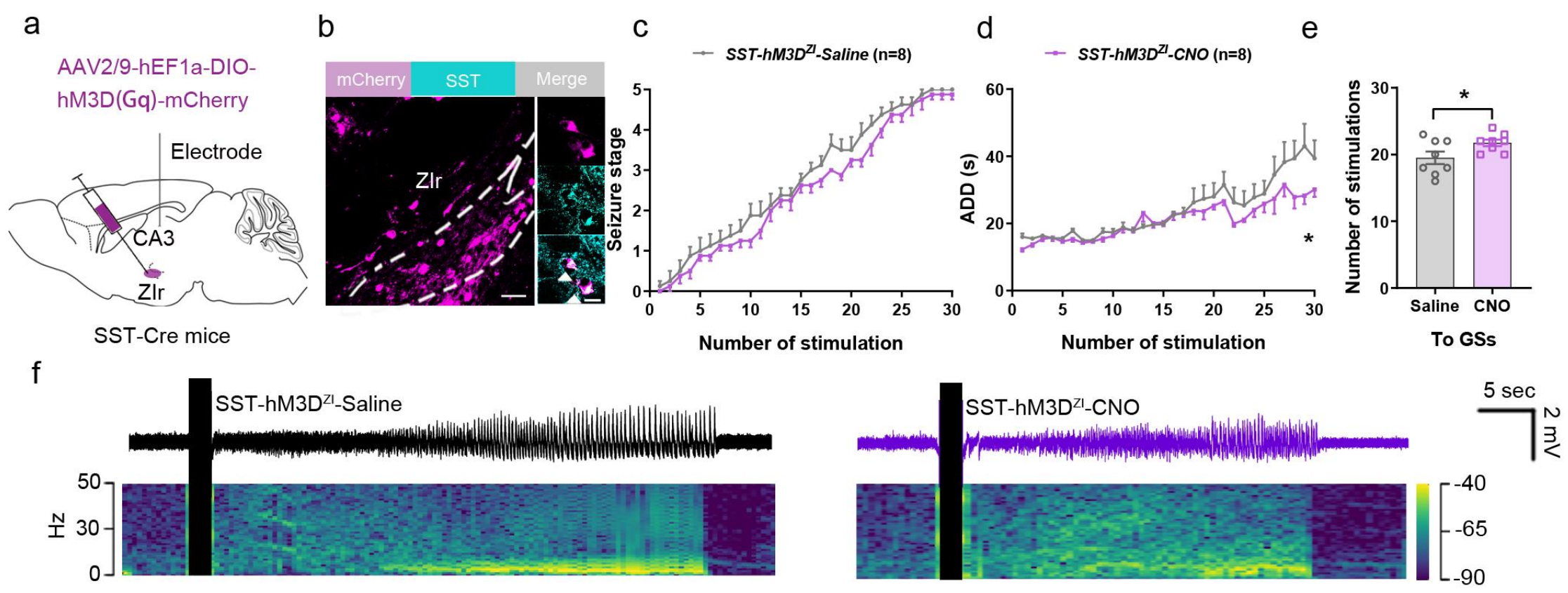

Supplementary Figure 8. Chemogenetic activation of ZI SST$^+$ GABAergic neurons attenuates seizure progress. (**a**) Scheme of experiment for viral injection and electrode implantation of ZI SST$^+$ GABAergic neuron. (**b**) Representative image of the ZI from an *SST-hM3D^{ZI}* mouse. Left: Scale bar = 200 μm; Enlarged image merged with SST. Right: Scale bar = 10 μm. (**c-e**) Effects of chemogenetic activation of ZI SST$^+$ GABAergic neurons on the development of seizure stage (**c**), ADD (**d**), and the number of stimulations of GSs onset. (**e**). n = 8 mice per group. (**f**) Representative EEGs and power spectrogram during seizures. Statistical analyses: Two-way ANOVA for repeated measures for (**c, d**). Two-tailed unpaired student's t test for (**e**). Significance levels were presented as *P < 0.05. Data are presented as means ± SEM. Exact P values are provided in Supplementary Data 1. Source data are provided as a Source Data file. (Abbreviations: ZIr, rostral zona incerta; CA3, hippocampal CA3; SST, somatostatin; ADD, after-discharge duration; GSs, generalized seizures).

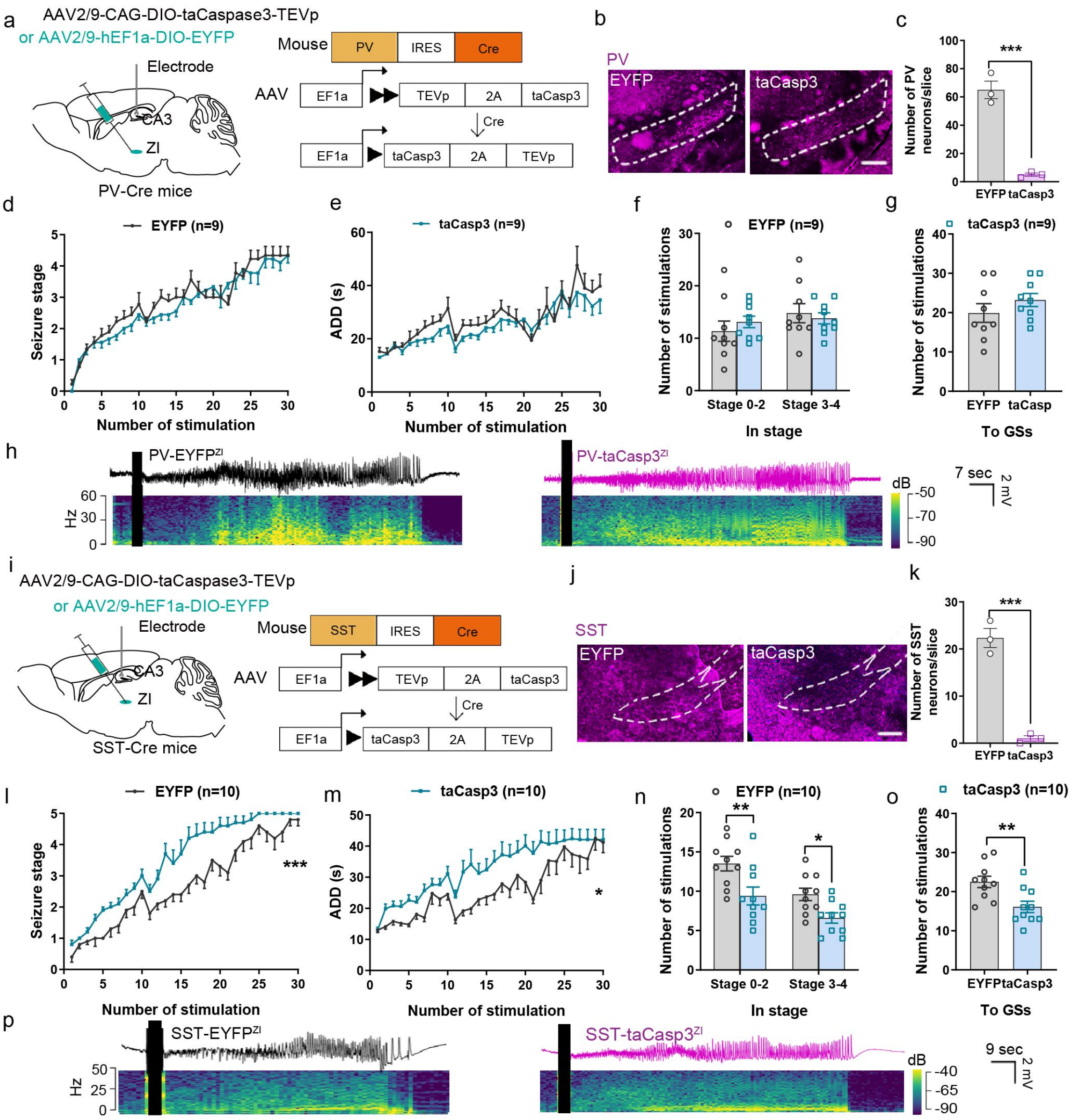

Supplementary Figure 9. Ablation of ZI SST^{+} but not PV^{+} GABAergic neurons amplifies seizure progression. (**a**) Scheme of experiment for ZI PV^{+} GABAergic neuron ablation (Left) and principle of virus ablation (Right). (**b, c**) Representative images of PV immunostaining (**b**) and statistics of PV expression quantity (**c**) in *PV::Cas-3* mouse and *PV::EYFP* mouse. Scale bar = 200 μm. (**d-g**) Effects of ablation of the ZI PV^{+} GABAergic neurons on the development of seizure stage (**d**), ADD (**e**), stage-specific stimulation numbers (**f**) and the number of stimulations of GSs onset (**g**) (n = 9 mice per group). (**h**) Representative EEGs and power spectrogram during seizures of *PV-EYFP^{ZI}* and *PV-taCasp3^{ZI}* mice. (**i**) Scheme of experiment for ZI SST^{+} GABAergic neuron ablation (Left) and principle of virus ablation (Right). Representative images of SST immunostaining (**j**) and statistics of SST expression quantity (**k**) in *SST::Cas-3* mouse and *SST::EYFP* mouse. Scale bar = 200 μm. Effects of ablation of the ZI SST^{+} GABAergic neurons on the development of seizure stage (**l**), ADD (**m**), stage-specific stimulation numbers (**n**) and stimulations to GSs onset (**o**) (n = 10 mice per group). (**p**) Representative EEGs and power spectrogram during seizures of *SST-EYFP^{ZI}* and

SST-taCasp3^{ZI} mice. Statistical analyses: Two-tailed unpaired student's t test for (**c, k**); Two-way ANOVA for repeated measures for (**d, e, l, m**); Two-way ANOVA with post-hoc Bonferroni test for (**f, n**); Two-tailed unpaired student's t test for (**g, o**). Significance levels were presented as *$P < 0.05$, **$P < 0.01$, or ***$P < 0.001$. Data are presented as means ± SEM. Exact P values are provided in Supplementary Data 1. Source data are provided as a Source Data file. (Abbreviations: ZI, zona incerta; CA3, hippocampal CA3; SST, somatostatin; PV, parvalbumin; ADD, after-discharge duration; GSs, generalized seizures).

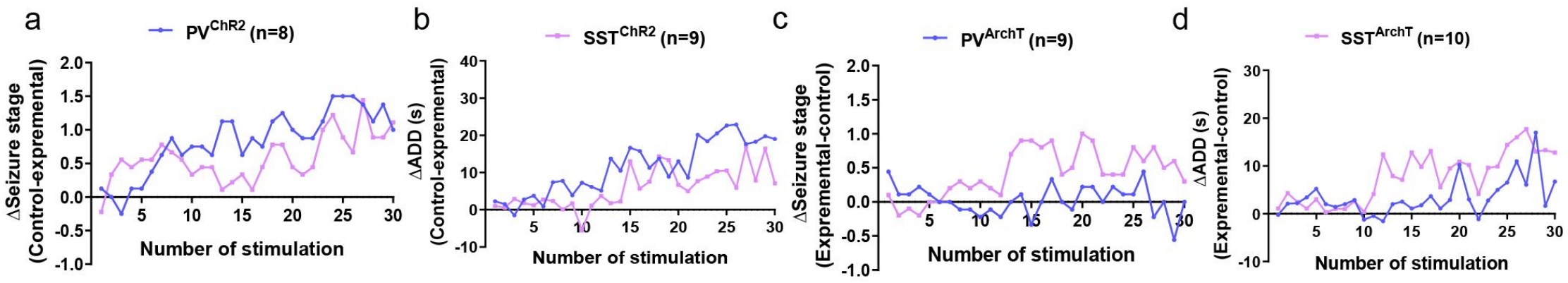

Supplementary Figure 10. The comparison of the effects of optogenetic regulation of PV and SST neurons on the seizure progression. (**a-d**) The relative change of seizure stage and ADD between control group and optical activation groups in *PV-ChR2*^{ZI} and *SST-oChR2*^{ZI} mice. *PV-ChR2*^{ZI}, n = 8 mice; *SST-oChR2*^{ZI}, n = 9 mice; *PV-ArchT*^{ZI}, n = 9 mice; *SST-ArchT*^{ZI}, n = 10 mice. Statistical analyses: Two-way ANOVA for repeated measures for (**a-d**). Data are presented as means ± SEM. Exact P values are provided in Supplementary Data 1. Source data are provided as a Source Data file. (Abbreviations: SST, somatostatin; PV, parvalbumin; ADD, after-discharge duration).

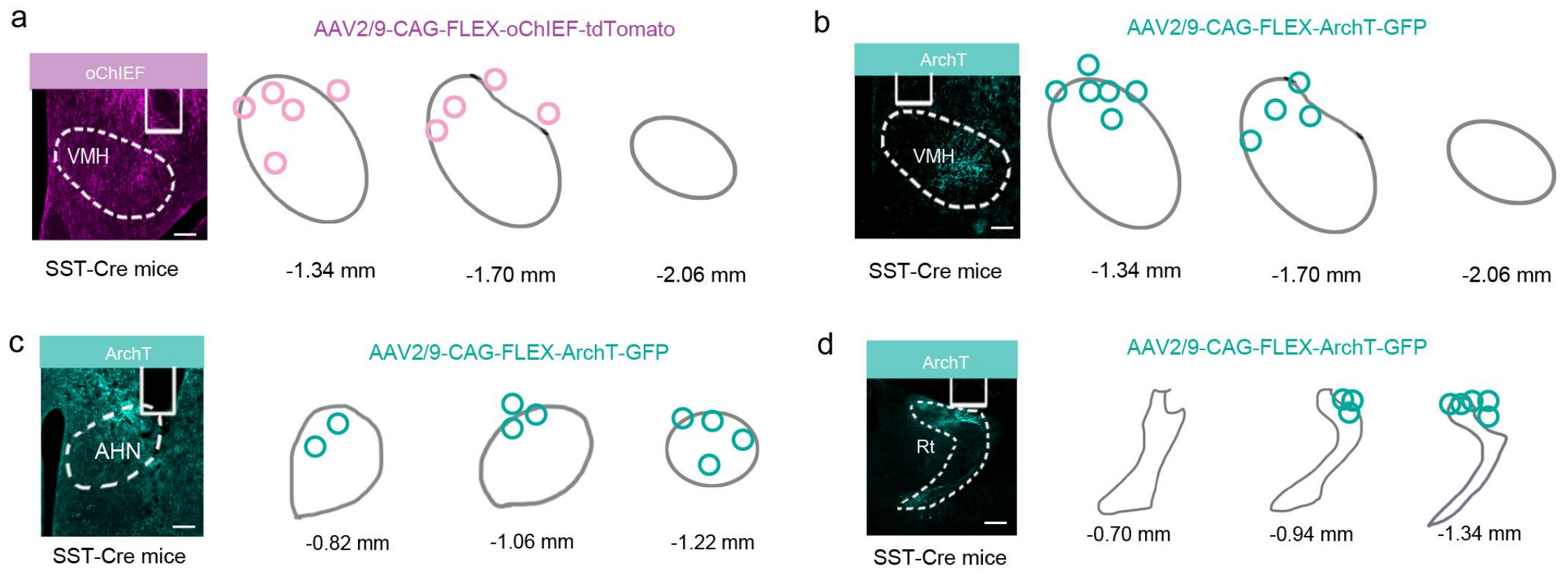

Supplementary Figure 11. Optical fiber implantation sites for ZI^{SST}-VMH, ZI^{SST}-AHN or ZI^{SST}-Rt circuit. (**a, b**) Schematic diagram of the optical fiber implantation locations of optogenetic activation (**a**) or inhibition (**b**) of ZI^{SST}-VMH circuit included in the statistics. (**c, d**) Schematic diagram of the optical fiber implantation locations of optogenetic inhibition of ZI^{SST}-AHN (**c**) and ZI^{SST}-Rt (**d**) circuit included in the statistics. Scale bar = 200 µm for (**a-d**). $SST^{ZI\text{-}VMH\text{-}oChIEF}$ mice, n = 9 mice; $SST^{ZI\text{-}VMH\text{-}ArchT}$ mice, n = 10 mice; $SST^{ZI\text{-}AHN\text{-}ArchT}$ mice, n = 9 mice; $SST^{ZI\text{-}Rt\text{-}ArchT}$ mice, n = 8 mice. (Abbreviations: VMH, ventromedial hypothalamus; AHN, anterior hypothalamic area; Rt, reticularis nucleus).

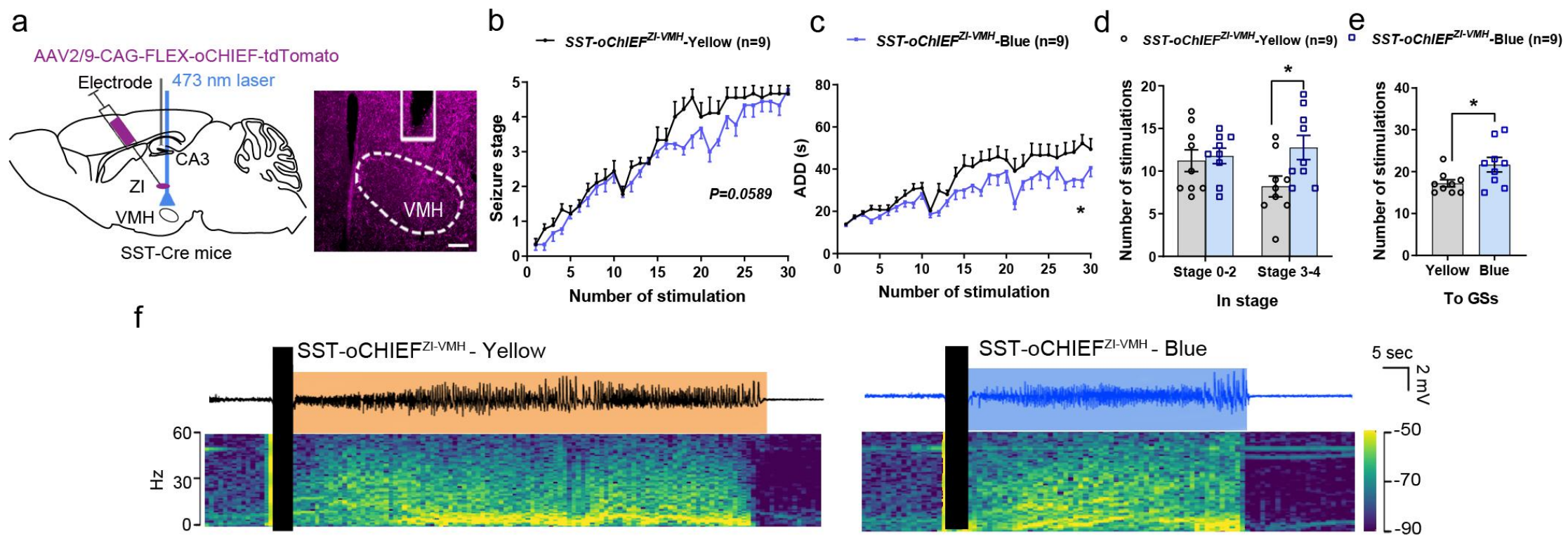

Supplementary Figure 12. Optogenetic activation of the ZI^{SST}-VMH circuit attenuates seizures progress. (**a**) Left: Schematic of viral injection, optical fiber implantation and photo-stimulation in *SST-oChIEF*$^{ZI\text{-}VMH}$ mice; Right: Representative image of optic fibers position in the VMH. Scale bar = 200 μm. (**b-e**) Effects of optogenetic activation of ZI^{SST}-VMH circuit on the seizure stage (**b**), ADD (**c**), stage-specific stimulation numbers (**d**) and the number of stimulations of GS onset (**e**). n = 9 mice. (**f**) Representative EEGs and power spectrogram during seizures of *SST-oChIEF$^{ZI\text{-}VMH}$* mice. Statistical analyses: Two-way ANOVA for repeated measures for (**b, c**); Two-way ANOVA with post-hoc Bonferroni test for (**d**); Two-tailed unpaired student's t test for (**e**). Significance levels were presented as *P < 0.05. Data are presented as means ± SEM. Exact P values are provided in Supplementary Data 1. Source data are provided as a Source Data file. (Abbreviations: ZI, zona incerta; SST, somatostatin; CA3, hippocampal CA3; VMH, ventromedial hypothalamus; ADD, after-discharge duration; GSs, generalized seizures).

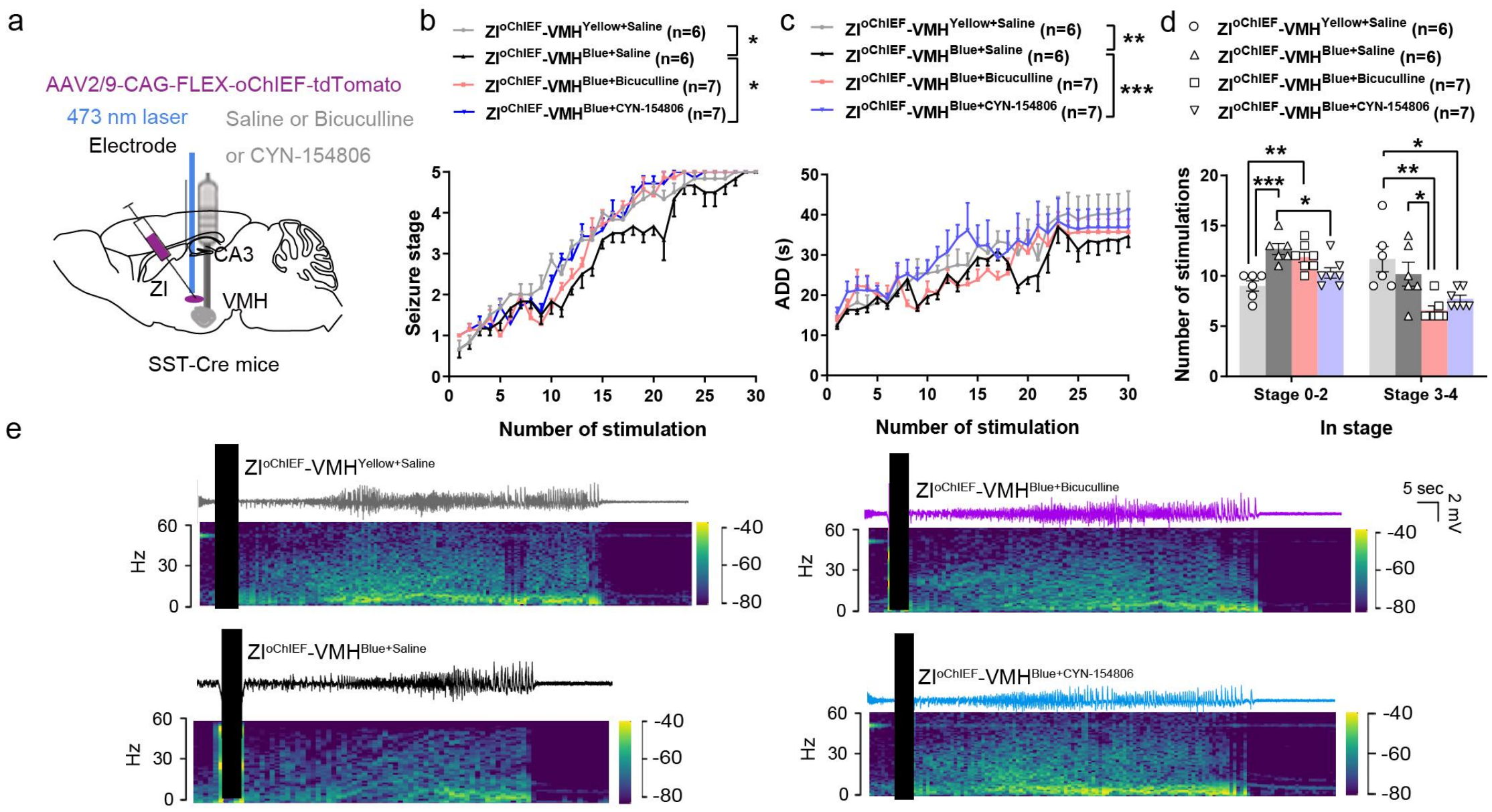

Supplementary Figure 13. Optogenetic activation of the ZI^{SST}-VMH circuit inhibits seizures in an SSTR-dependent manner. (**a**) Schematic of viral injection and cannula implantation in *SST-oChIEF^{ZI}* mice. (**b-d**) Effects of optogenetic activation of ZI^{SST}-VMH circuit and pharmacogenetic antagonize SST or GABA antibodies on the seizure stage (**b**), ADD (**c**), stage-specific stimulation numbers (**d**). *ZI^{oChIEF}-VMH$^{Yellow+Saline}$*, n = 6 mice; *ZI^{oChIEF}-VMH$^{Blue+Saline}$*, n = 6 mice; *ZI^{oChIEF}-VMH$^{Blue+Bicuculline}$*, n = 7 mice; *ZI^{oChIEF}-VMH$^{Blue+CYN-154806}$*, n = 7 mice. (**e**) Representative EEGs and power spectrogram during seizures of four groups of *ZI^{oChIEF}-VMH$^{Yellow+Saline}$*, *ZI^{oChIEF}-VMH$^{Blue+Saline}$*, *ZI^{oChIEF}-VMH$^{Blue+Bicuculline}$*, *ZI^{oChIEF}-VMH$^{Blue+CYN-154806}$* mice. Statistical analyses: Two-way ANOVA for repeated measures followed by Tukey's multiple comparisons test for (**b, c**). One-way ANOVA by Tukey's multiple comparisons test for (**d**). Significance levels were presented as $*P < 0.05$, $**P < 0.01$, or $***P < 0.001$. Data are presented as means ± SEM. Exact P values are provided in Supplementary Data 1. Source data are provided as a Source Data file. (Abbreviations: ZI, zona incerta; SST, somatostatin; CA3, hippocampal CA3; VMH, ventromedial hypothalamus; ADD, after-discharge duration).

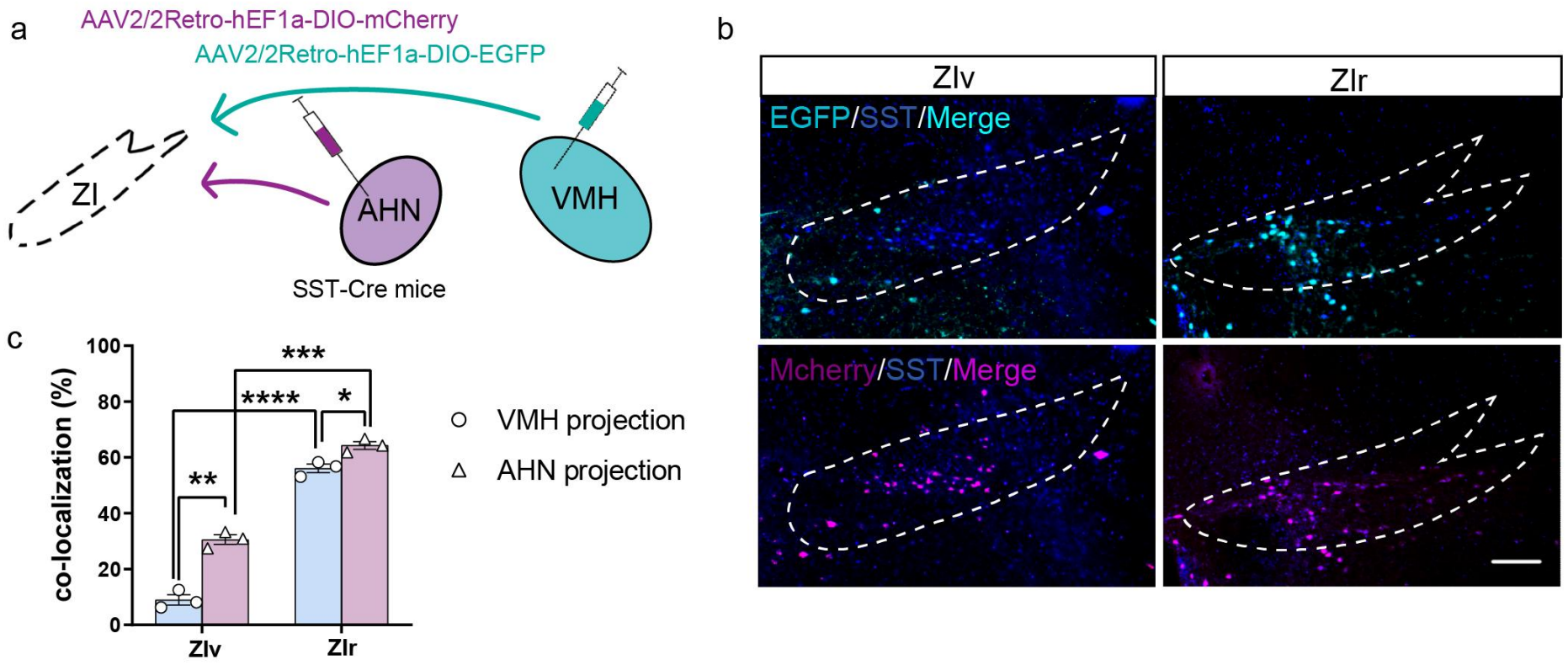

Supplementary Figure 14. Retrograde tracing identifies a subpopulation of ZI SST neurons projecting to the AHN and VMH. (**a**) Schematic diagram of injection of AAV2/2Retro-hEF1α-DIO-EGFP and AAV2/2Retro-hEF1α-DIO-mCherry virus into VMH and AHN receptively to retrograde trace ZI SST neurons. (**b**) Representative image of viral expression in ZIr and ZIv sections. Scale bar = 100 μm. (**c**) The ratio of retro virus co-labeled SST neurons to the total number of SST neurons in the ZIr and ZIv regions. n = 3 mice. Statistical analyses: Two-tailed unpaired student's t test for (**c**). Data are presented as means ± SEM. Exact P values are provided in Supplementary Data 1. Source data are provided as a Source Data file. (Abbreviations: ZIr, rostral zona incerta; ZId, ventral zona incerta; SST, somatostatin; VMH, ventromedial hypothalamus; AHN, anterior hypothalamic area).

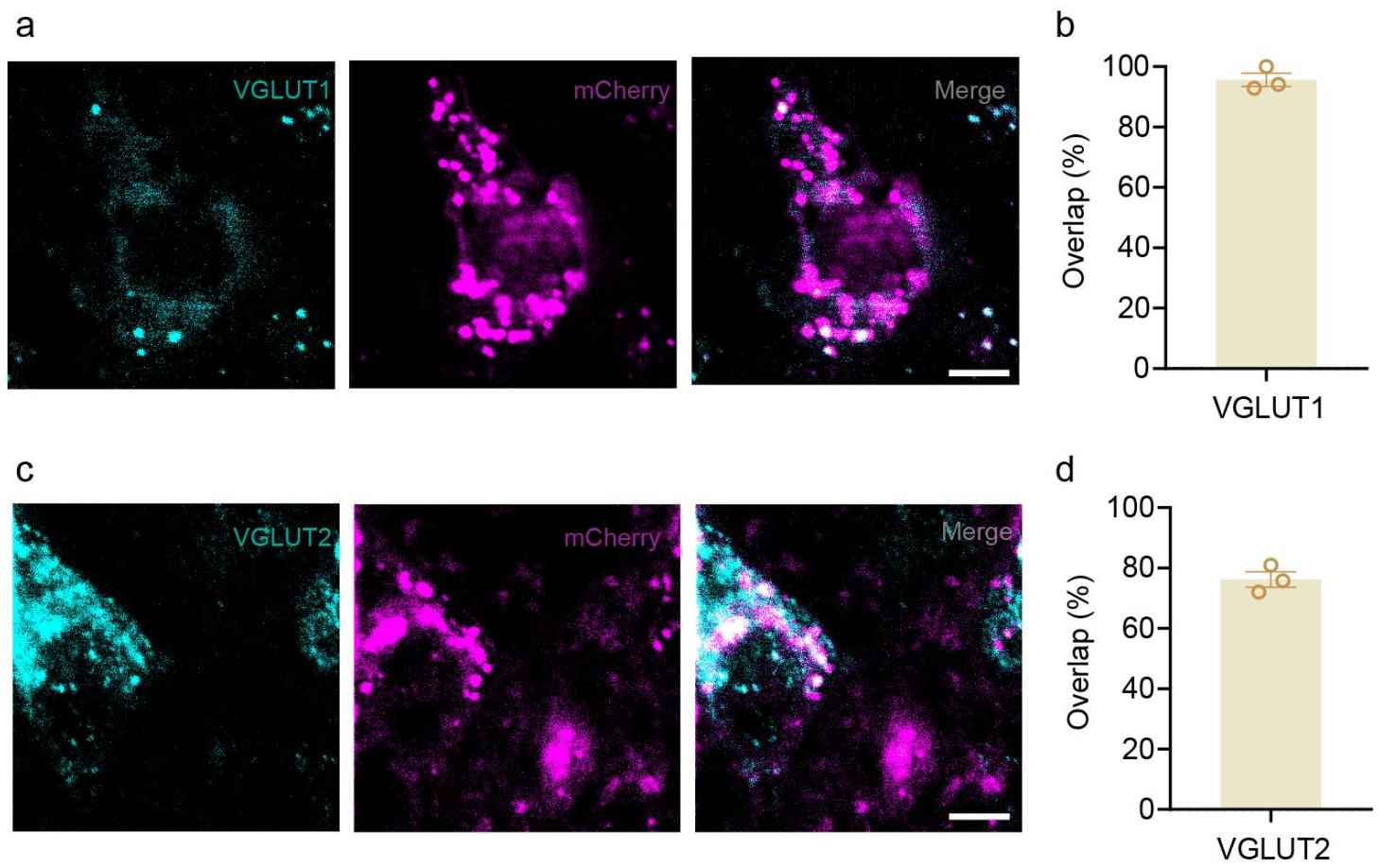

Supplementary Figure 15. VMH glutamatergic neurons co-localize with VGLUT1 or VGLUT2. (**a, b**) Representative images and quantification of VGLUT1 co-stanning with CaMKII-mCherry in the VMH. Scale bar = 5 µm. n = 3 mice. (**c, d**) Representative images and quantification of VGLUT2 co-stanning with CaMKII-mCherry in the VMH. Scale bar = 5 µm. n = 3 mice. Data are presented as means ± SEM. Source data are provided as a Source Data file.

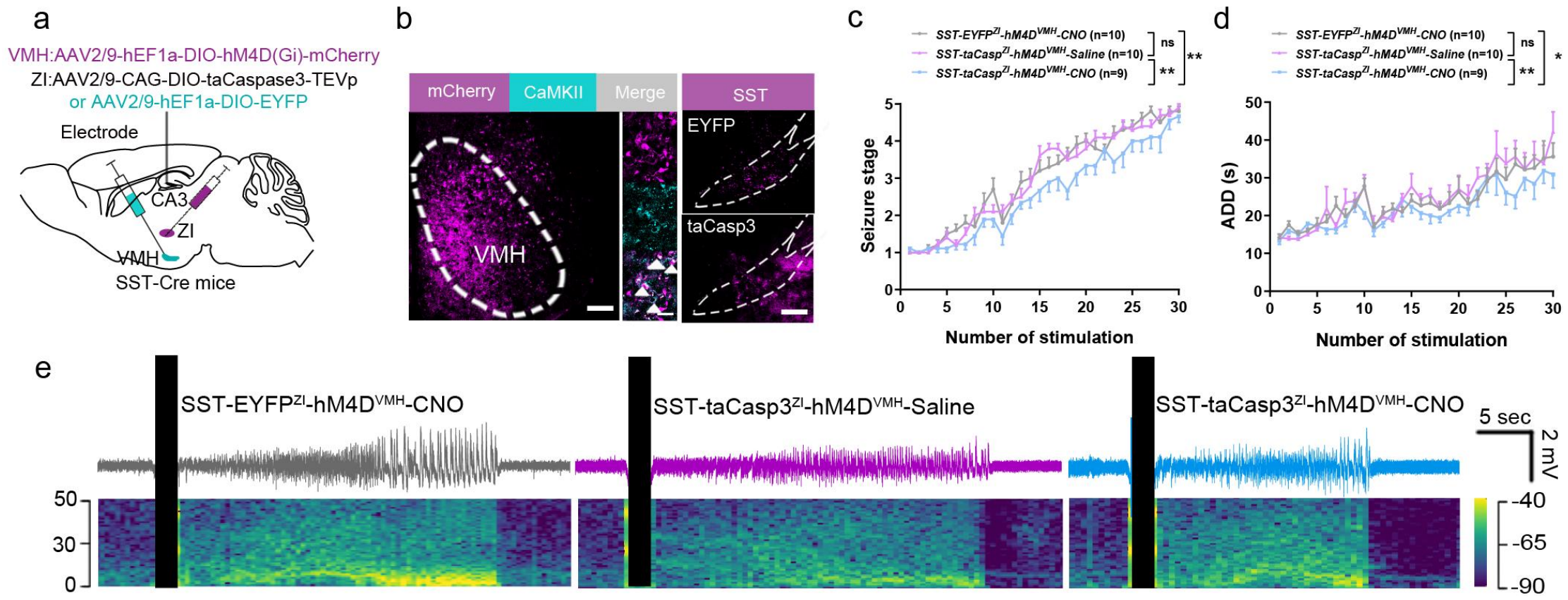

Supplementary Figure 16. Ablation of ZI SST^{+} neurons inhibits hippocampal seizure progression via suppression of VMH glutamatergic neurons. (**a**) Scheme of experiment for viral injection and electrodes implantation in *SST-taCasp^{ZI}-hM4D^{VMH}* mouse. (**b**) Left: Representative image of the VMH from *SST-taCasp^{ZI}-hM4D^{VMH}* mouse. Scale bar = 150 μm; Enlarged image merged with CaMKII. Scale bar = 50 μm. Right: Representative images of SST immunostaining in *SST-taCasp^{ZI}-hM4D^{VMH}* mouse and *SST-EYFP^{ZI}-hM4D^{VMH}* mouse. Scale bar = 200 μm. (**c, d**) Effects of ablation of ZI SST^{+} neurons and chemogenetic inhibition of VMH glutamatergic neurons on the seizure stage (**c**), and ADD (**d**). *SST-EYFP^{ZI}-hM4D^{VMH}-CNO*, n = 10 mice; *SST-taCasp^{ZI}-hM4D^{VMH}-Saline*, n = 10 mice; *SST-taCasp^{ZI}-hM4D^{VMH}-CNO*, n = 9 mice. (**e**) Representative EEGs and power spectrogram during seizures of three groups of *SST-EYFP^{ZI}-hM4D^{VMH}-CNO*, *SST-taCasp^{ZI}-hM4D^{VMH}-Saline*, and *SST-taCasp^{ZI}-hM4D^{VMH}-CNO* group. Statistical analyses: Two-way ANOVA for repeated measures followed by Tukey's multiple comparisons test for (**c, d**). Significance levels were presented as $*P < 0.05$, or $**P < 0.01$. Data are presented as means ± SEM. Exact P values are provided in Supplementary Data 1. Source data are provided as a Source Data file. (Abbreviations: ZI, zona incerta; SST, somatostatin; hippocampal CA3; VMH, ventromedial hypothalamus; ADD, after-discharge duration; CNO, clozapine-N-oxide).

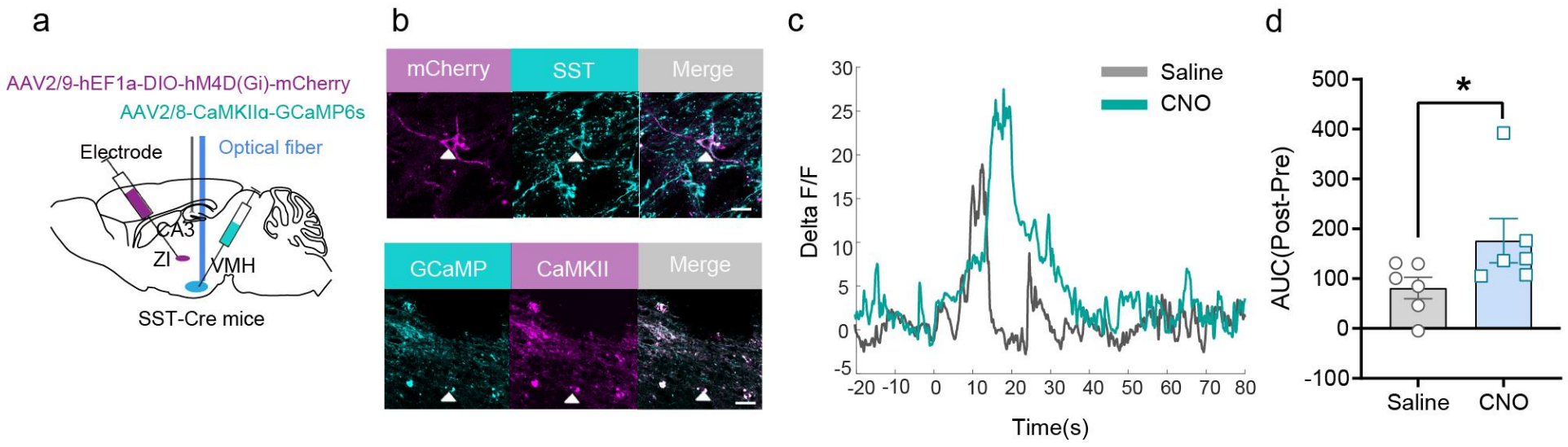

Supplementary Figure 17. Inhibition of ZI SST⁺ neurons increased the activity of VMH glutamatergic neurons. (**a**) Scheme of experiment for viral injection and Ca^{2+} fiber photometry of VMH glutamatergic neurons during kindling-induced hippocampal seizures. (**b**) Top: representative enlarged image of hM4D-mCherry with SST in ZI. Scale bar = 50 μm. Bottom: representative enlarged image of GCaMP with CaMKII in VMH. Scale bar = 50 μm. (**c, d**) The calcium signals ($\Delta F/F$) and group data of trials showing quantification of the average AUC of VMH glutamatergic neurons during hippocampal seizure. n = 6 mice. Statistical analyses: Two-tailed Mann-Whitney test for (**d**). Significance levels were presented as *P < 0.05. Data are presented as means ± SEM. Exact P values are provided in Supplementary Data 1. Source data are provided as a Source Data file. (Abbreviations: ZI, zona incerta; SST, somatostatin; hippocampal CA3; VMH, ventromedial hypothalamus; CNO, clozapine-N-oxide).

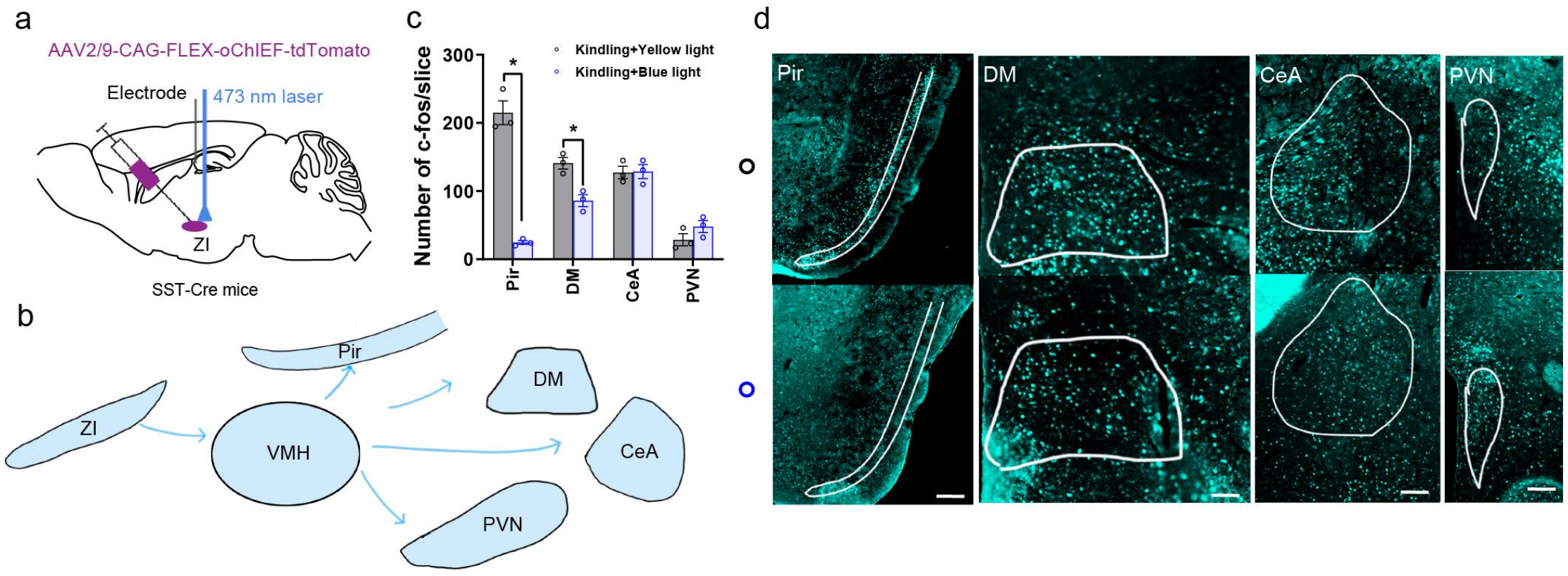

Supplementary Figure 18. The changes of c-fos in the downstream brain region of VMH during hippocampal seizures. (**a**) Scheme of experiment for viral injection and photo-stimulation in *SST-oChIEF*^{ZI} mice. (**b**) Schematic diagram of downstream projection in the VMH. (**c**) Immunohistochemical statistics in the brain regions of axonal terminal distribution and significant changes in c-Fos. n = 3 mice. (**d**) Representative images of brain regions in c-Fos immunostaining. DM (Scale bar = 300 μm), Pir, CeA and PVN (Scale bar = 200 μm). Statistical analyses: Two-way ANOVA with post-hoc Bonferroni test for (**c**). Significance levels were presented as *P < 0.05. Data are presented as means ± SEM. Exact P values are provided in Supplementary Data 1. Source data are provided as a Source Data file. (Abbreviations: ZI, zona incerta; SST, somatostatin; VMH, ventromedial hypothalamus; DM, dorsomedial hypothalamic nucleus; CeA, central amygdala; PVN, paraventricular hypothalamic nucleus; Pir, piriform cortex).

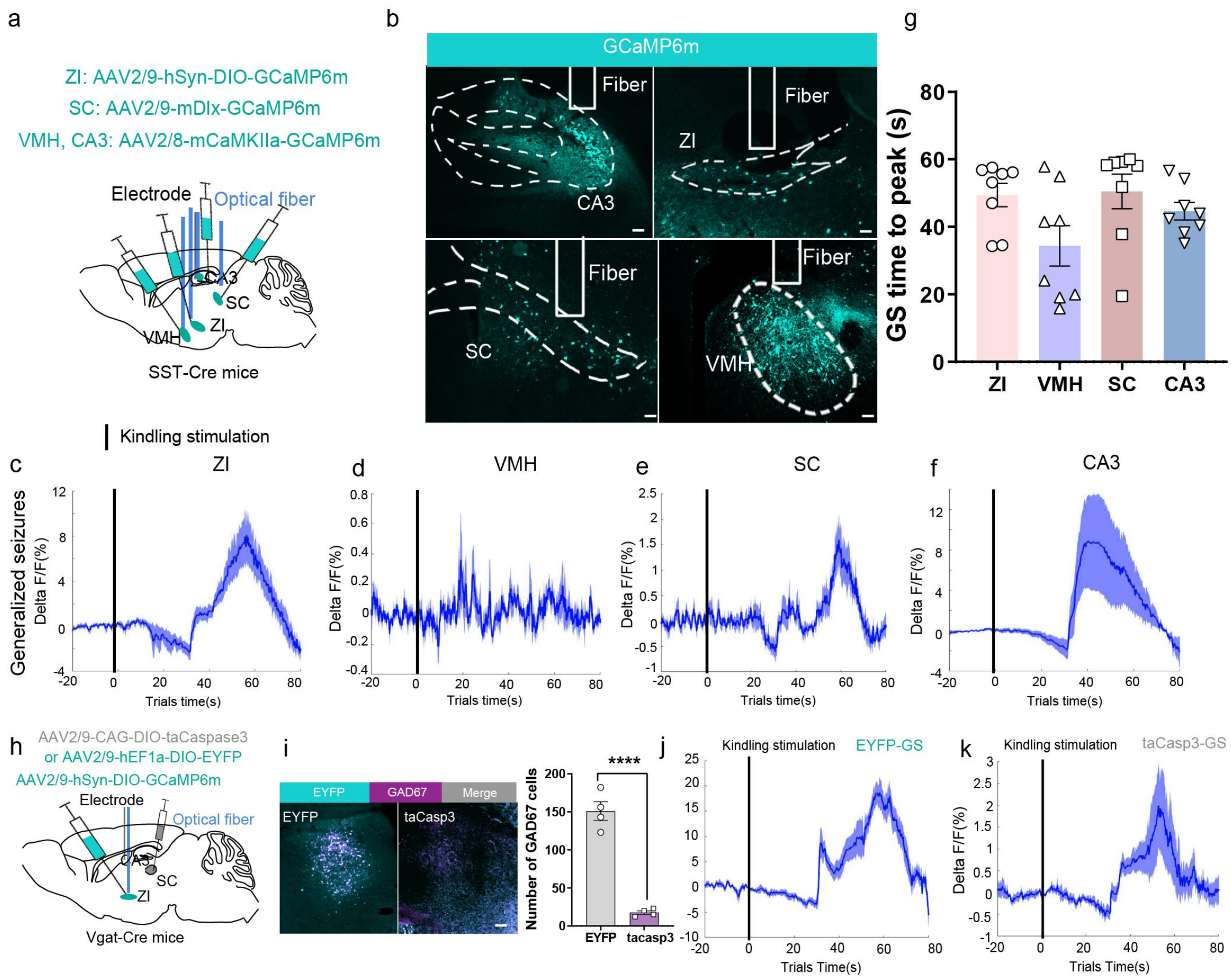

Supplementary Figure 19. SC neurons are involved in the activity of hippocampal epileptic seizures in the ZI-VMH circuit. (**a**) Scheme of experiment for viral injection and Ca^{2+} fiber photometry of ZI, VMH, SC, and CA3 areas during kindling-induced hippocampal seizures. (**b**) Representative image of CaMP6m expression and fiber locations in the ZI, VMH, SC, and CA3. Green = CaMP6m. Scale bar = 100 µm. (**c-f**) The average calcium signals (ΔF/F) of ZI (**c**), VMH (**d**), SC (**e**), and CA3 (**f**) neuron during GSs. (**g**) Latency to peak of the calcium signal in ZI, VMH, SC, and CA3 during GS. (**h**) Scheme of experiment for viral injection of SC neuronal ablation and Ca^{2+} fiber photometry. (**i**) Representative images of GAD67 immunostaining and statistics of GAD67 expression in $Vgat^{GCaMP}$::Cas-3 mouse and $Vgat^{GCaMP}$::EYFP mouse. Scale bar = 200 µm. n = 4 mice. (**j, k**) The average calcium signals (ΔF/F) of ZI GABAergic neurons during GS of EYFP and Cas-3 groups. n = 8 mice. Statistical analyses: Two-tailed unpaired student's t test for (**i**). Significance levels were presented as ****P < 0.0001. Data are presented as means ± SEM. Exact P values are provided in Supplementary Data 1. Source data are provided as a Source Data file. (Abbreviations: ZI, zona incerta; SST, somatostatin; VMH, ventromedial hypothalamus; SC, superior colliculus; CA3, hippocampal CA3; GSs, generalized seizures).

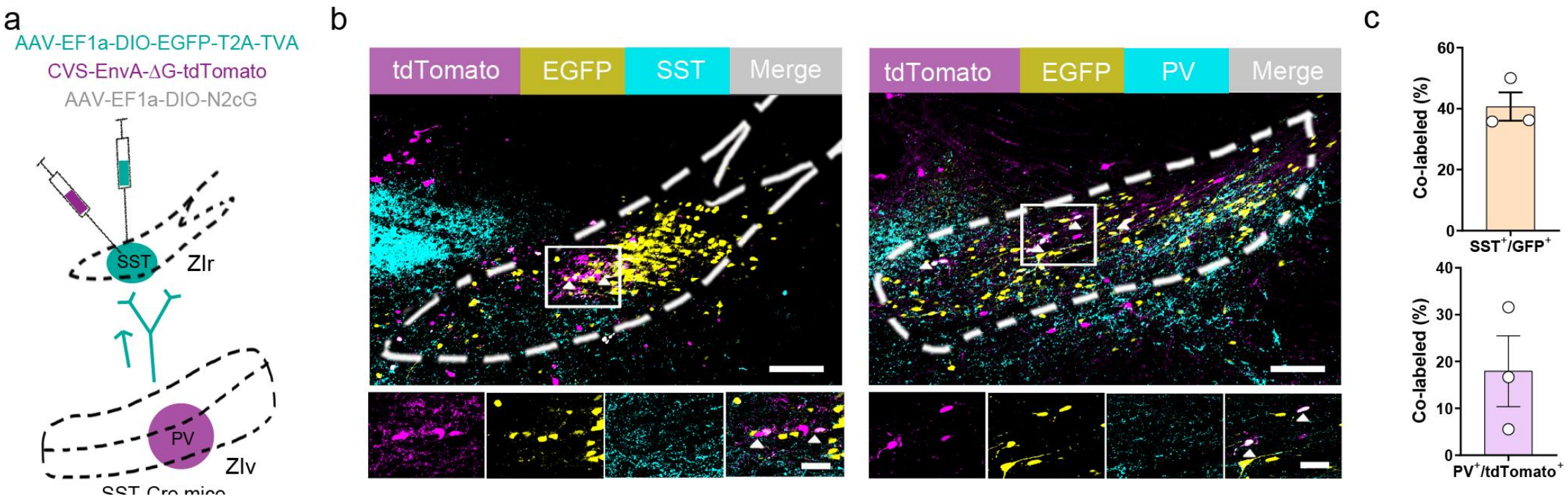

Supplementary Figure 20. PV$^+$ neurons serve as inputs for SST$^+$ neurons within ZI. (**a**) Schematic of the experimental design for ZIr SST neurons retrograde monosynaptic tracing in *SST-Cre* mice using a modified rabies virus system. (**b**) Left: Representative images of the ZIr injected with the helper virus and rabies virus. The double-infected (bright white) SST cells represent the starter cells. Scale bar = 200 µm. Higher-magnification images showing SST$^+$ GABAergic neurons infected by the rabies virus (EGFP). Scale bar = 50 µm. Right: Overlapping PV$^+$/tdTomato$^+$ fluorescence indicates ZIv PV$^+$ neurons directly innervate ZIr SST$^+$ neurons. Scale bar = 200 µm. Higher-magnification images showing PV$^+$ GABAergic neurons infected by the tdTomato. Scale bar = 50 µm. (**c**) Top: The quantitative ratio of SST$^+$/EGFP$^+$ co-positive neurons among total SST$^+$ cells; Bottom: the quantitative ratio of PV$^+$/tdTomato$^+$ co-positive neurons among total PV$^+$ cells, n = 3 mice. Data are presented as means ± SEM. Source data are provided as a Source Data file. (Abbreviations: ZIr, rostral zona incerta; ZId, ventral zona incerta; SST, somatostatin; PV, parvalbumin).

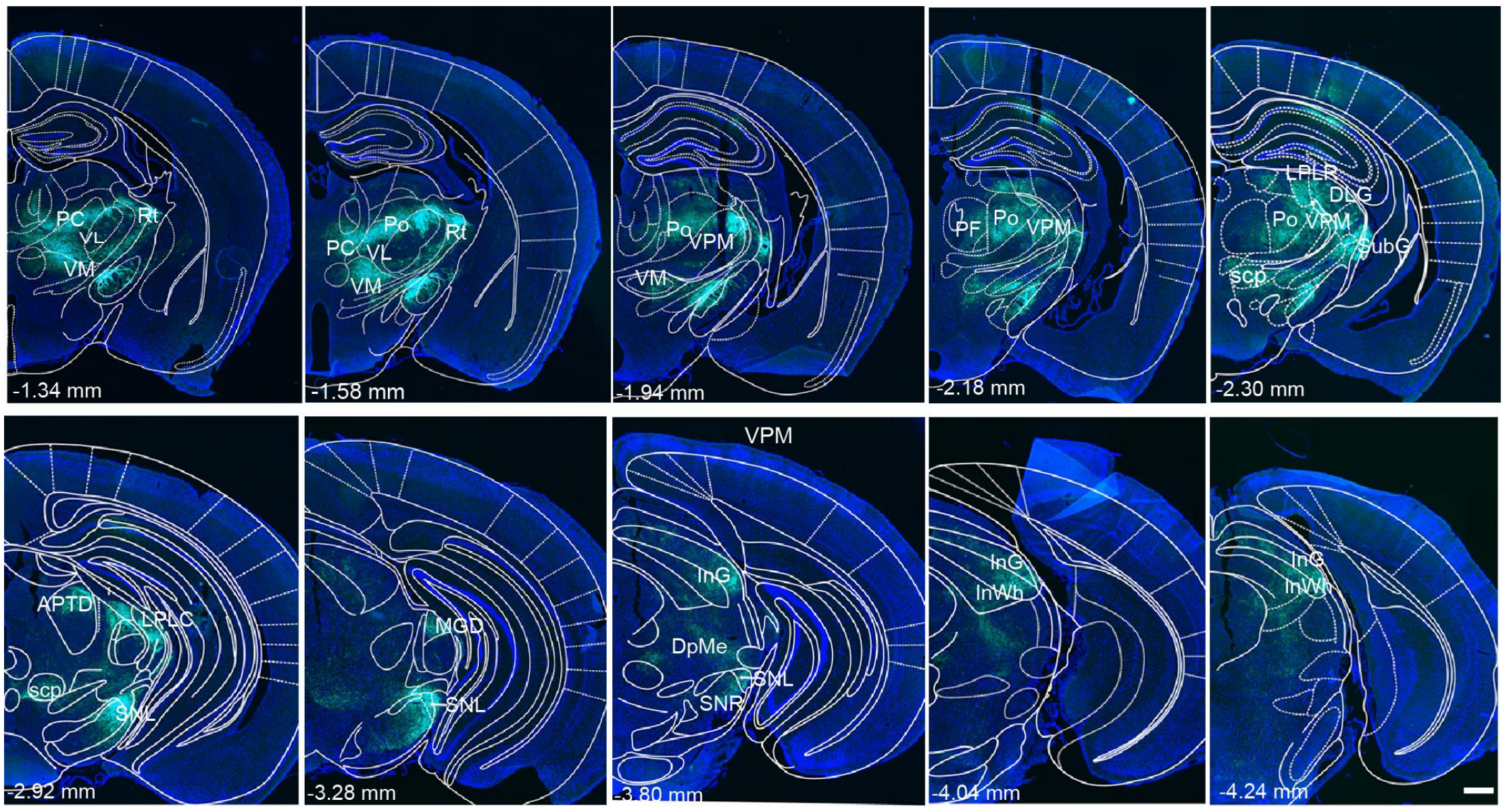

Supplementary Figure 21. The downstream structural mapping of ZI PV$^+$ neurons in *PV-ArchT^{ZI}* mice. Scale bar = 600 μm. (Abbreviations: APTD, anterior pretectal nucleus, dorsal part; DLG, dorsal lateral geniculate nucleus; DpMe, dorsal peduncular cortex; InG, intermediate gray layer of the superior colliculuslateral hypothalamic area; InWh, intermediate white layer of the superior colliculus; LPLC, lateral posterior thalamic nucleus, mediocaudal part; LPLR, ateral posterior thalamic nucleus, laterorostral part; MGD, medial geniculate nucleus, dorsal part; PC, paracentral thalamic nucleus; PF, parafascicular thalamic nucleus; Po, posterior thalamic nuclear group; scp, superior cerebellar peduncle; SNL, substantia nigra, lateral part; SNR, substantia nigra, reticular part; SubG, subgeniculate nucleus of prethalamus (ventrolateral nucleus); VM, ventromedial thalamic nucleus; VL, ventrolateral thalamic nucleus; VPM, ventral posteromedial thalamic nucleus.)
