# Supplementary material for: Zona incerta somatostatin+ neurons modulate seizure progression through hypothalamic circuits
Source: Nat Commun. 2026 Sep 29;17:11234. doi: 10.1038/s41467-026-78174-9 (PMC13720055; doi:10.1038/s41467-026-78174-9)
Supplement: Supplementary file 2 [file 41467_2026_78174_MOESM2_ESM.pdf]

Description of Additional Supplementary Files

Supplementary Data 1: The exact statistic parameter for all statistically significant comparisons.
