# Supplementary material for: Zona incerta somatostatin+ neurons modulate seizure progression through hypothalamic circuits
Source: Nat Commun. 2026 Sep 29;17:11234. doi: 10.1038/s41467-026-78174-9 (PMC13720055; doi:10.1038/s41467-026-78174-9)
Supplement: Supplementary file 3 [file 41467_2026_78174_MOESM4_ESM.pdf]

nature portfolio

Corresponding author(s): Yi Wang

Last updated by author(s): Aug 13, 2026

Reporting Summary

Nature Portfolio wishes to improve the reproducibility of the work that we publish. This form provides structure for consistency and transparency in reporting. For further information on Nature Portfolio policies, see our Editorial Policies and the Editorial Policy Checklist.

Statistics

For all statistical analyses, confirm that the following items are present in the figure legend, table legend, main text, or Methods section.

n/a	Confirmed	
☐	☒	The exact sample size (*n*) for each experimental group/condition, given as a discrete number and unit of measurement
☐	☒	A statement on whether measurements were taken from distinct samples or whether the same sample was measured repeatedly
☐	☒	The statistical test(s) used AND whether they are one- or two-sided<br>*Only common tests should be described solely by name; describe more complex techniques in the Methods section.*
☒	☐	A description of all covariates tested
☐	☒	A description of any assumptions or corrections, such as tests of normality and adjustment for multiple comparisons
☐	☒	A full description of the statistical parameters including central tendency (e.g. means) or other basic estimates (e.g. regression coefficient) AND variation (e.g. standard deviation) or associated estimates of uncertainty (e.g. confidence intervals)
☐	☒	For null hypothesis testing, the test statistic (e.g. *F*, *t*, *r*) with confidence intervals, effect sizes, degrees of freedom and *P* value noted<br>*Give P values as exact values whenever suitable.*
☒	☐	For Bayesian analysis, information on the choice of priors and Markov chain Monte Carlo settings
☒	☐	For hierarchical and complex designs, identification of the appropriate level for tests and full reporting of outcomes
☐	☒	Estimates of effect sizes (e.g. Cohen's *d*, Pearson's *r*), indicating how they were calculated

Our web collection on statistics for biologists contains articles on many of the points above.

Software and code

Policy information about availability of computer code

Data collection	Immunohistochemistry images were captured by fluorescence microscope DM6B (Leica, Germany). Photometry data were acquired with photometry system (Inper Ltd., China).
Data analysis	Immunohistochemistry images analyses and quantification were performed using ImageJ-win64. The calcium data were analyzed byby MATLAB (2021b, MathWork, USA). Statistical comparisons were performed using Graphpad Prism software (version 8) and SPSS (version 25) with appropriate methods describing in methods section and related figure legends.

For manuscripts utilizing custom algorithms or software that are central to the research but not yet described in published literature, software must be made available to editors and reviewers. We strongly encourage code deposition in a community repository (e.g. GitHub). See the Nature Portfolio guidelines for submitting code & software for further information.

Data

Policy information about availability of data

All manuscripts must include a data availability statement. This statement should provide the following information, where applicable:

- Accession codes, unique identifiers, or web links for publicly available datasets
- A description of any restrictions on data availability
- For clinical datasets or third party data, please ensure that the statement adheres to our policy

The source data generated in this study are provided in the Supplementary Information/Source Data file. Additional data related with this paper is available from the corresponding author. Source data are provided with this paper.

Research involving human participants, their data, or biological material

Policy information about studies with human participants or human data. See also policy information about sex, gender (identity/presentation), and sexual orientation and race, ethnicity and racism.

Reporting on sex and gender	No human participant or data were included in this manuscript.
Reporting on race, ethnicity, or other socially relevant groupings	No human participant or data were included in this manuscript.
Population characteristics	No human participant or data were included in this manuscript.
Recruitment	No human participant or data were included in this manuscript.
Ethics oversight	No human participant or data were included in this manuscript.

Note that full information on the approval of the study protocol must also be provided in the manuscript.

Field-specific reporting

Please select the one below that is the best fit for your research. If you are not sure, read the appropriate sections before making your selection.

[x] Life sciences [ ] Behavioural & social sciences [ ] Ecological, evolutionary & environmental sciences

For a reference copy of the document with all sections, see nature.com/documents/nr-reporting-summary-flat.pdf

Life sciences study design

All studies must disclose on these points even when the disclosure is negative.

Sample size	No statistical methods were used to pre-determine sample size. The sample size was determined according to previous studies (PMID:35294025, PMID: 37898623).
Data exclusions	Animals in which histological examination showed that viral expression or position of optic fibers were in the incorrect location were excluded.
Replication	The experiment for each study was successfully repeated for at least two times.
Randomization	All samples used in the study were randomly allocated into different experimental groups.
Blinding	The data collection and analysis were performed in the double-blind manner.

Reporting for specific materials, systems and methods

We require information from authors about some types of materials, experimental systems and methods used in many studies. Here, indicate whether each material, system or method listed is relevant to your study. If you are not sure if a list item applies to your research, read the appropriate section before selecting a response.

Materials & experimental systems

n/a	Involved in the study	
[ ]	[x]	Antibodies
[x]	[ ]	Eukaryotic cell lines
[x]	[ ]	Palaeontology and archaeology
[ ]	[x]	Animals and other organisms
[x]	[ ]	Clinical data
[x]	[ ]	Dual use research of concern
[x]	[ ]	Plants

Methods

n/a	Involved in the study	
[x]	[ ]	ChIP-seq
[x]	[ ]	Flow cytometry
[x]	[ ]	MRI-based neuroimaging

Antibodies

Antibodies used	Primary antibodies: anti-c-Fos (1:500, Ab208942, Abcam); anti-GAD67 (1:200, HUABIO, ED1703-71); anti-Parvalbumin (1:500, Abcam, 181086); anti-Somatostatin 28 (1:500, HUABIO, HA721460); anti-VGLUT1 (1:200, HUABIO, HA601449); anti-VGLUT2 (1:200, HUABIO, HA601451); anti-VGLUT2 (1:200, OriGene, TA322266S); anti-CaMKIIα (1:500, Ab52476, Abcam); anti-nNOS (1:200, HUABIO, R1510-28). Secondary antibodies: goat anti-mouse 488 (1:1000, Ab150113, Abcam), goat anti-rabbit 594 (1:1000, Ab150080, Abcam), and goat anti-rabbit 647 (1:1000, Ab150079, Abcam).
Validation	The antibodies have been validated extensively. For anti-c-Fos, please see references PMID:35338850, PMID: 37488426. For anti-

GAD67, please see reference PMID:37910621; For anti-Parbalbumin, please see reference PMID: 39042031; For anti-Somatostatin 28, please see reference PMID: 39658255; For anti-CaMKIIα please see references PMID: 35338850, PMID: 36516780; For anti-nNOS, please see reference PMID: 36753544. For goat anti-mouse 488, please see references PMID: 31590222, PMID: 336266453. For goat anti-rabbit 594, please see references PMID: 35389758, PMID: 33472123. For goat anti-rabbit 647, please see references PMID: 32051591, PMID: 30086801.

Animals and other research organisms

Policy information about studies involving animals; ARRIVE guidelines recommended for reporting animal research, and Sex and Gender in Research

Laboratory animals	All mice used inin this study were healthy, male C57BL/6J mice, aged 2–4 months. The Vgat-Cre mice (stock number: 016962), PV-Cre mice (stock number: 008069), SST-Cre (stock numberz: 013044), CaMKIIα-Cre mice (stock number: 005539) were genotyped according to the protocols provided by Jackson Laboratory. All mice were housed in a 12h light/12h dark cycle (lights on from 8:00 to 20:00) at the temperature of 22±2 degrees Celsius, humidity 55±5% and with ad libitum food and water.
Wild animals	This study did not involve wild animals.
Reporting on sex	Male and female mice were used in this study.
Field-collected samples	This study did not involve samples collected from the field.
Ethics oversight	All experiments were carried out following the National Institutes of Health guidelines, and all procedures were approved by the local ethics review committee of Zhejiang Chinese Medical University.

Note that full information on the approval of the study protocol must also be provided in the manuscript.

Plants

Seed stocks	No plants were used in this experiment
Novel plant genotypes	No plants were used in this experiment
Authentication	No plants were used in this experiment
