# Supplementary material for: Zona incerta somatostatin+ neurons modulate seizure progression through hypothalamic circuits
Source: Nat Commun. 2026 Sep 29;17:11234. doi: 10.1038/s41467-026-78174-9 (PMC13720055; doi:10.1038/s41467-026-78174-9)
Supplement: Supplementary file 4 [file 41467_2026_78174_MOESM5_ESM.pdf]

nature portfolio

Peer Review File

Zona Incerta somatostatin+ neurons modulate seizure progression through hypothalamic circuits

Corresponding Author: Professor Yi Wang

This file contains all reviewer reports in order by version, followed by all author rebuttals in order by version.

Version 0:

Reviewer comments:

Reviewer #1

(Remarks to the Author)
In the present study, Mengdi Zhang et al. investigate the role of rZI SST+ GABAergic neurons in modulating seizures, a condition for which the neurobiological mechanisms remain incompletely understood. By using distinct mouse lines to subclassify GABAergic neurons in the zona incerta, the authors identify a specific GABAergic subpopulation that modulates seizure progression. They map its downstream circuit and validate the pathway's specificity using optogenetics, chemogenetics, and fiber photometry. These findings will deepen our understanding of the mechanisms underlying seizure propagation.
Major concerns:
1. Since no direct projection exists between the ZI and hippocampal CA3, does the elevated ZI signal observed during kindling result from the electrical stimulation of hippocampus itself or from other upstream inputs? Could the intensity of hippocampal stimulation influence the calcium level of ZI neurons? Could the authors give some explanations? ZIv neurons are densely connected with the SC and have been implicated in head-orienting movements. Could the SC act as an upstream seizure-modulatory region, and does electrical stimulation of hippocampal CA3 influence SC activity?
2. c-Fos staining shows a decrease in the number of activated ZI cells after seizures. Are these activated cells co-labelled with SST or PV neurons, and what is the exact proportion?
3. As a neuropeptide transmitter, does somatostatin (SST) itself mediate the seizure-modulating effect of the ZIr SST–VMH circuit?
4. Increased evidence is showing that ZI different sectors ranging from anterior to posterior may function differently. Could stimulation of other sectors a different bregma site produce an even stronger phenotype?
Minor concerns:
The label of y axis in Fig1P is wrong and should be ADD(s). Fig1O-P, the statistical marker star should be given a range, other similar cases should be also pay attention to.
Row 166 The hyphen in "Chan-nelrhodopsin 2 (ChR2)" should be removed.

Reviewer #2

(Remarks to the Author)
The manuscript entitled "Zona Incerta Somatostatin+ Circuit Attenuates Seizures Progression" investigates the role and neural circuit mechanisms of somatostatin (SST)-positive neurons in the zona incerta (ZI) during seizure progression in a hippocampal kindling model. Using optogenetics combined with fiber photometry calcium imaging, behavioral analysis, and EEG recordings, the authors demonstrate that ZI SST neurons, but not parvalbumin (PV)-positive neurons, bidirectionally modulate the progression of seizures in this model. Additionally, they identify ventromedial hypothalamus (VMH) glutamatergic neurons as a potential downstream target of ZI SST neurons in seizure progression. While the study addresses an important topic, I have several concerns regarding the results presented in this manuscript.

Major points:
1. The study employs the hippocampal kindling model to simulate epilepsy. This model is induced by electrically stimulating the hippocampal CA3 region, indicating that the seizure onset occurs in the hippocampus. The authors have chosen the zona incerta (ZI) as a potential target region for modulating seizure progression, which is a reasonable choice. However,

they need to provide evidence demonstrating how the seizure activity spreads from the hippocampus to the ZI and clarify the exact role of the ZI during seizure progression. The same applies to the downstream region of the ZI, the ventromedial hypothalamus (VMH). The authors should conduct additional experiments or analyses to show the propagation of seizure activity from the hippocampus to the ZI and subsequently to the VMH. For example, simultaneous recording of GCaMP signals in the hippocampus, ZI, and VMH could be performed. Additionally, analyzing the correlation between GCaMP and EEG signals during optogenetic activation of ZI SST neurons at different seizure stages would provide valuable insights.
2. line98-99: It is necessary to first introduce how to induce hippocampal seizure and how to determine the starting point of calcium signals；a co-labeling staining and quantitative analysis of neurons of different types in ZI and fos should be added.
3. How is the expression of fos in other brain regions during kindling-induced hippocampal seizure? It is hoped that the author will add this part of the results to reflect the importance of Zi in this model.
4. While both ZI PV and SST neurons are activated during seizures, only SST neurons bidirectionally modulate seizure progression. However, the manuscript lacks mechanistic explanations for this difference. Additional experiments are necessary to elucidate why SST neurons, but not PV neurons, exhibit bidirectional control.
5. Fiber photometry data indicate that ZI PV and SST neurons are activated during seizures; however, optogenetic activation of these neurons inhibits seizure progression. This apparent contradiction requires clarification. The authors should address and reconcile these observations through further analysis.
6. Optogenetic activation of ZI GABAergic neurons does not completely terminate seizures, suggesting that their role may be auxiliary rather than central. The authors should discuss the functional significance of ZI GABAergic neurons within the hippocampal kindling model and consider potential compensatory mechanisms that might influence seizure modulation.
7. line160-162：PV+ neurons exhibited a gradual but sustained increase in calcium activity, whereas SST+ neurons displayed a delayed but rapid surge in calcium signals (Fig. 2D and N). This definition is rather vague. It is hoped that the author will make further comparisons before drawing relevant conclusions.
8. In Figure2, the optogenetic results should also be compared between PV and SST to reflect the differences in their roles.
9. The manuscript would improve with a more thorough data analysis. (1) Quantify the correlation between kindling EEG and ZI GCaMP6 signals shown in Figure 1; (2) Perform statistical comparisons of the differences in EEG and GCaMP6 signal durations between the PV and SST groups presented in Figure 2; (3) Analyze the impact of optogenetic inhibition of ZI SST/PV neurons on changes in EEG power spectra.
10. The optogenetic experiments are missing proper control groups. Figures 1 and 2 should incorporate a "seizure-only" group to show the activity patterns of Zi neurons during seizures.
11. Does Figure 4F simultaneously record two sites of ZI SST and downstream brain regions? Please provide detailed explanations in the manuscription or make further annotations in the figures.
12. In Figure5, are D-blue and J-blue control groups? There is a significant difference in the number of stimulations in stage 0-2 between D-blue and J-blue. Are there any statistical differences here ? Is this result caused by stimulating different circuits ? It is suggested that inter-group analysis be conducted on different circuit-specific activation to further demonstrate that the effect of a certain circuit-specific activation is more significant.
13. The GCaMP6 signals in the VMH, AHN, and Rt occur more quickly than in ZI SST neurons, even though the ZI is upstream (figure 4G-L and figure 1C,D and M,N). This inconsistency needs to be clarified, as it may indicate the presence of alternative pathways or possible technical errors.
14. ZI SST neurons are GABAergic and are therefore expected to inhibit VMH neurons when activated. Activating ZI SST neurons reduces seizure progression, implying that these neurons act by suppressing VMH glutamatergic neurons. As a result, the activity of VMH neurons should decrease. However, Figure 6E indicates that activating ZI SST neurons and stimulating VMH glutamatergic neurons lead to opposite outcomes. The authors should clarify whether this inconsistency is due to direct connections or indirect circuit mechanisms.
15. The manuscript does not exclude direct hippocampal projections to VMH, AHN, or Rt, which could confound interpretations of the ZI SST→downstream pathway.
16. Using CaMKII alone to identify glutamatergic neurons is inadequate since GABAergic projection neurons, like medium-sized spiny neurons in the striatum, can also express CaMKII. Thus, it is necessary to use additional markers (such as vGluT1/2) for confirmation.
17. Figure 6D does not demonstrate direct innervation from ZI SST neurons to the VMH. To confirm monosynaptic connections, slice electrophysiology techniques, such as optogenetic IPSC recordings, are required.
18. Are there any other types of neurons in the Zi brain region? Does it play a role in hippocampal seizure ?

Minor points:

1. The scale bar value is missing in Fig 1A; high-magnification images for c-Fos staining should be provided.
2. Clarify how hippocampal kindling stimulation was delivered to the ipsilateral ZI (manual vs. automated?). And only 10ms stimulation ?
3. Provide high-magnification co-staining images to confirm GCaMP6 expression in PV/SST neurons (For example, Figs 1B, G, N; 2B, F, L, P; 3B, H).
4. Include c-Fos staining in the hippocampus as a positive control for seizure activity.
5. Does inhibiting ZI PV or SST neurons induce seizures in naïve mice? This would test their intrinsic role in seizure threshold.
6. Show ZI injection/verification images to confirm the accuracy of stimulation sites.
7. Improve resolution and clarity for all immunohistochemistry images.
8. In Fig 2A-B and K-L, the recording sites of GCaMP here are inconsistent between PV-Cre and SST-Cre；in E-F and O-P，here, the expression level of ChR2 varies significantly, which will lead to differences in the results ?
9. In Fig 4E, please provide high-magnification images.

Reviewer #3

(Remarks to the Author)
I co-reviewed this manuscript with one of the reviewers who provided the listed reports. This is part of the Nature Communications initiative to facilitate training in peer review and to provide appropriate recognition for Early Career Researchers who co-review manuscripts.

Reviewer #4

(Remarks to the Author)
This manuscript by Zhang et al. investigates the role of zona incerta (ZI) GABAergic neurons in a hippocampal kindling model of epilepsy. Using calcium fiber photometry, optogenetics, chemogenetics, and viral tracing, the authors show that ZI GABAergic neurons are activated during seizure progression, and that activation of ZI SST+ neurons — but not PV+ neurons — is both necessary and sufficient to suppress seizure spread. Mechanistically, they demonstrate that SST+ projections to the ventromedial hypothalamus (VMH) inhibit glutamatergic neurons, thereby reducing seizure progression. This study addresses an underexplored circuit node in epilepsy, providing valuable mechanistic insights and potential therapeutic targets. However, there are several major issues regarding the lack of proper controls, statistical rigor of analysis, and interpretation of data that need to be addressed.

Major Points
1. PV+ vs SST+ contribution
Optogenetic activation of both PV+ and SST+ neurons reduces seizure progression, yet only SST+ inhibition/ablation has an effect. The manuscript interprets this as PV+ neurons being dispensable. However, this asymmetry requires deeper discussion:
• Could PV+ projections to other targets explain why inhibition shows no effect?
• Is optogenetic inhibition/ablation of PV+ neurons complete and efficient?
• Alternative interpretations should be addressed explicitly.

2. Statistical rigor
The Methods state that no sample size calculation or randomization was performed. For a Nature Communications paper, this is a significant weakness. The authors should:
Clarify whether n represents animals or cells in every figure.
Provide justification for chosen sample sizes.
Clarify whether data were pooled across multiple cells per animal and how independence was handled (nested statistics, mixed models).

For several datasets, especially the photometry datasets, it seems that the sample sizes were especially low.

The graphs in Figure 6F and G does not portray such a difference as indicated by the noted significance in the legends. In particular the data in Figure 6G seem to be highly overlapping between the groups.

3. Controls for optogenetic/chemogenetic experiments
For chemogenetic experiments, please provide vehicle (CNO-only) control data to exclude off-target effects.
For optogenetic experiments, show quantification of EEG traces with and without light stimulation across animals, beyond representative examples.

4. Photometry under anesthesia
In Fig. 1, calcium signals under isoflurane anesthesia are used to rule out movement artifacts. However, seizure dynamics under anesthesia differ substantially from awake states. The rationale and limitations of this control should be better justified in the manuscript.

5. Circuit specificity and downstream claims
The VMH pathway is emphasized as the critical effector, but c-Fos mapping also revealed modulation in AHN and piriform cortex. Claims that the VMH is the exclusive mediator are overstated. The authors should temper conclusions or provide further experiments to strengthen specificity.

Given the proximity of VMH and AHN to each other, how do the authors know the specificity of their viral targeting and imaging for these regions?

Lines 232-235 is incorrect since only VMH signal in generalized seizures is significantly higher.
Regarding Supplemental Figure 3, how do the authors know that they are selectively activating the VMH targets and not the overlying ZI (based on their diagram)?

Diagram in Figure 6 suggests that SST neurons projecting to VMH also project to other brain areas. What are these other brain areas and why don't they exert a similar effect?

6. Relationship to prior work by the same group
The authors previously reported (Zhang et al., 2024) that ZI GABAergic neurons, targeted via Vgat-Cre, are recruited during seizures, with bidirectional manipulation affecting seizure progression. The present manuscript extends this work by

dissecting ZI interneuron subtypes and demonstrating that SST+ neurons — but not PV+ neurons — are both necessary and sufficient for seizure suppression. The connection to their prior work should be made more explicit: how does the broad GABAergic effect map onto the subtype-specific findings? Does this mean that the anti-seizure effect observed in the earlier study was primarily mediated by SST+ neurons, while PV+ contributions were secondary or redundant? Clarifying this progression is essential for readers to understand the added value of the current work.

Minor Points
1. The authors note the Pir as an ictogenic trigger but it is unclear how this is related to their specific hippocampal kindling epilepsy model and to their seizure circuit where Pir is downstream of ZI and VMH.

2. Lines 160-162 in reference to Figure 2D and N need to specify that this is only the case for generalized seizures

3. It is unclear why the authors decided to use continuous application of yellow light for their optogenetic inactivation experiments which may induce phototoxicity and desensitization of the channel.

4. Improve figure legends: clearly state n per group, units, and definitions of error bars (SEM vs SD).

5. Correct terminology: e.g., “glutaminergic” should be replaced with “glutamatergic.”

6. Improve English clarity throughout (many small grammar issues: e.g., “are bidirectionally modulate seizure” → “bidirectionally modulate seizure”).
In the first sentence of the abstract “pivotal regulator” should be “pivotal regulators”.
In line 60 “types” should be “type”
Paragraph 59 to 63 is confusing as it is : I suggest revising to something like: “Among epilepsy subtypes, temporal lobe epilepsy (TLE) is one of the most common drug-resistant forms, with seizures originating in limbic regions—particularly the hippocampus (Englot et al., 2020; Keezer et al., 2016)—and often spreading to generalized seizures (GS) due to the complex epileptogenic networks underlying TLE.”
In line 70 “implicating” should be “implicated” .
Discuss discrepancies with prior literature: several studies (e.g., Yekhlef et al., 2015; Chen et al., 2020) report that SST+ or PV+ activation can promote seizures in other circuits. How do these findings reconcile with the present data?

7. Methods: injection volumes of 200 µL (line 461) seem unusually large for stereotactic mouse injections — this should be checked, possibly a typo for 200 nL.

8. It is unclear why an ArchT virus was used solely for projection mapping in Figure 4A.

9. In Figure 4D is the cFos in hippocampus significantly higher in the blue light condition?

10. Line 245 “decreased” should be “increased”.

Reviewer #5

(Remarks to the Author)
I co-reviewed this manuscript with one of the reviewers who provided the listed reports. This is part of the Nature Communications initiative to facilitate training in peer review and to provide appropriate recognition for Early Career Researchers who co-review manuscripts.

Version 1:

Reviewer comments:

Reviewer #1

(Remarks to the Author)
The authors has well addressed my concerns. I have no additional comments.

Reviewer #2

(Remarks to the Author)
The manuscript has been significantly improved; however, I still have some questions and concerns that I would like to discuss with the author.
1. Regarding the role of Zi SST neurons, the author proposes that Zi SST functions as an inhibitory factor within the epileptic network, acting like a brake during seizures, though its influence is insufficient to fully suppress them. The data indeed show that with increasing intensity of electrical stimulation, the GCaMP signals of Zi SST neurons intensify. Furthermore, optogenetic activation of Zi SST neurons demonstrates a degree of suppression of epileptic activity, a conclusion with which I agree. However, I hold a different view regarding the role of the VMH. In Supplementary Figure 15, the kindling stimulation-induced activation of the Zi, VMH, SC, and CA3 regions appears nearly indistinguishable, leading to two possible interpretations: either the sampling rate of the fiber photometry recordings is insufficient to resolve the activation sequence of

these brain regions, or, as I have previously speculated, the VMH receives not only inhibitory projections from the Zi but also excitatory inputs from CA3 and other sources. Therefore, the role of the VMH in facilitating epilepsy may not be solely dependent on the Zi. I suggest conducting additional experiments to further elucidate the role of the Zi SST-DMH pathway.
First, the authors have already performed experiments activating the Zi SST-DMH axons; they could add another group to inhibit the terminals of Zi SST-DMH neurons, thereby confirming whether the effects of Zi SST ablation are mediated through the DMH.
Second, while ablating Zi SST neurons, chemogenetic or optogenetic inhibition of DMH glutamatergic neuron activity could be tested to determine whether this suppresses epileptic occurrences, thereby clarifying the role of the DMH in this pathway.
Third, in conditions where Zi SST neurons are ablated or inhibited, leading to increased susceptibility to seizures, it would be valuable to determine whether this correlates with a significant increase in DMH activity.

2. The research methods are generally somewhat monotonous. By regulating PV or SST+ neurons in the Zi through optogenetics, no termination of seizures was observed. Could this be related to the selected optogenetic stimulation frequency? The chosen frequency might not have effectively activated the PV+ neurons. It is necessary to conduct additional experiments to observe the effects of optogenetic stimulation at different frequencies and stimulation intensities on anti-seizure effects.

3. the role of PV+ neurons within the Zi regulating the occurrence and development of seizure seizures possible mediated the feedforward effect on SST+ neurons or other mechanisms?

4. The author analyzed EEG power during epileptic episodes but did not present the power distribution across different EEG frequency bands.

5. Regarding HPC c-fos positivity as a control, the provided data focuses on the DG Hilus area. What are the results for the CA3 region?

6. To further clarify the role of SST+ neurons within the Zi in regulating epileptic seizures, can additional regulatory methods, beyond optogenetic modulation, be employed to observe the impact of SST+ neuron excitability on seizure activity?

Reviewer #4

(Remarks to the Author)
While the authors have addressed my concerns, several inaccurate or incorrect statements exist in the revised manuscript which need to be corrected:

1) Lines 218-219: "suggesting that ZI SST+ GABAergic neurons (not PV+ GABAergic neurons) are required for seizure progression." This is incorrect since seizure progression is still intact, it is just enhanced. Any claims regarding requirement for seizure progression need to be revised.
2) Lines 232-233: "SST^+ neurons had a significantly greater impact on seizure delay or acceleration, especially during the progression phase (Supplementary Fig. 8)." This is incorrect since none of the comparisons in Supplementary Fig. 8 are significantly different based on the p values noted in the figure legend.
3) Line 271: As noted in my earlier review, the authors cannot conclude that all the downstream circuits were activated since only VMH during FS and GS and AHN during GS show significant elevated calcium signals. In addition, the comparison noted in the text, about calcium signal being gradually elevated from FS to GS, is not actually shown by the data in Fig. 4 which compares FS and GS separately.
4) Line 314-315: The statement that "ZI SST+ neurons projecting to the VMH, but not to the AHN...contributes to seizure progression" is incorrect since inhibition of the ZI SST+-AHN circuit did lead to changes in seizure progression (just opposite of what was seen with ZI SST+-VMH circuit)
5) Line 334: VGLUT1 should be VGLUT2.
6) While some parts are improved, the text is still difficult to read due to grammatical errors throughout the manuscript. Professional copyediting is recommended.

Reviewer #5

(Remarks to the Author)
I co-reviewed this manuscript with one of the reviewers who provided the listed reports. This is part of the Nature Communications initiative to facilitate training in peer review and to provide appropriate recognition for Early Career Researchers who co-review manuscripts.

Version 2:

Reviewer comments:

Reviewer #2

(Remarks to the Author)
1. The manuscript title differs between the main text and the Supplementary Materials. In addition, the main-text title does not clearly state the key finding and should be revised.

2. In the revised manuscript, the changes prompted by reviewer comments could be more clearly indicated, and it would help if their locations were noted in the response letter. This is especially important for substantive revisions. For example, the authors wrote "Taken together, we totally agree with your view that the role of the VMH in facilitating seizures may not be solely dependent on the ZI," but it is not entirely clear to me where this discussion was actually added to the manuscript. Similarly, regarding Reviewer 4's concerns about Lines 218–219 and Lines 232–233, the authors appear to have revised the conclusion statements themselves, though I may have overlooked a corresponding update in the Discussion. I would suggest that for all key revisions, the exact location in the manuscript be stated explicitly in the response letter.

3. Supplementary Figure 1d shows data for DG, CA1, and CA3, but the magnified insets currently only display DG. It would be helpful to also include representative images for CA1 and CA3.

4. I am curious about one point: optogenetic manipulation of ZI PV^+ or SST^+ neurons mainly shifts the lower-frequency EEG components (delta band) without affecting gamma oscillations. Gamma activity is classically tied to PV^+ interneuron function, so this dissociation is unexpected and worth noting. If the authors feel it is appropriate, a brief discussion of this point in the Discussion would be helpful.

Open Access This Peer Review File is licensed under a Creative Commons Attribution 4.0 International License, which permits use, sharing, adaptation, distribution and reproduction in any medium or format, as long as you give appropriate credit to the original author(s) and the source, provide a link to the Creative Commons license, and indicate if changes were made.
In cases where reviewers are anonymous, credit should be given to 'Anonymous Referee' and the source.
The images or other third party material in this Peer Review File are included in the article's Creative Commons license, unless indicated otherwise in a credit line to the material. If material is not included in the article's Creative Commons license and your intended use is not permitted by statutory regulation or exceeds the permitted use, you will need to obtain permission directly from the copyright holder.
To view a copy of this license, visit https://creativecommons.org/licenses/by/4.0/

Review 1.

In the present study, Mengdi Zhang et al. investigate the role of rZI SST+ GABAergic neurons in modulating seizures, a condition for which the neurobiological mechanisms remain incompletely understood. By using distinct mouse lines to subclassify GABAergic neurons in the zona incerta, the authors identify a specific GABAergic subpopulation that modulates seizure progression. They map its downstream circuit and validate the pathway's specificity using optogenetics, chemogenetics, and fiber photometry. These findings will deepen our understanding of the mechanisms underlying seizure propagation.

Reply: We sincerely appreciate the time and effort you have taken to review our manuscript and for the positive feedback. Your comments are highly valuable and have significantly contributed to the revision and improvement of our paper. In this letter, we address each of your comments individually, followed by our corresponding responses. For clarity, we have restated your comments in bold font and provided our responses in blue.

Major concerns:

1. Since no direct projection exists between the ZI and hippocampal CA3, does the elevated ZI signal observed during kindling result from the electrical stimulation of hippocampus itself or from other upstream inputs? Could the intensity of hippocampal stimulation influence the calcium level of ZI neurons? Could the authors give some explanations? ZIv neurons are densely connected with the SC and have been implicated in head-orienting movements. Could the SC act as an upstream seizure-modulatory region, and does electrical stimulation of hippocampal CA3 influence SC activity?

Reply: We appreciate your valuable comments and suggestions!

(1) Given the lack of direct anatomical projections between the zona incerta (ZI) and hippocampal CA3, the elevated calcium activity observed in ZI neurons during kindling may not result from direct hippocampal input, but instead from other upstream circuits. As the reviewer noted, ventral ZI neurons are densely interconnected with the SC (Arena et al., 2024; Power et al., 2001), supporting a potential role for the SC as an upstream node in seizure-related circuitry. To test this hypothesis, we performed additional experiments to monitor neuronal calcium dynamics in the SC during hippocampal CA3 stimulation. Electrical stimulation of hippocampal CA3 robustly evoked a significant increase in SC calcium activity during seizures (revised Supplementary Figure 15A-G). Notably, the onset of hippocampal after-discharges was consistently accompanied by a rapid and sustained elevation in SC calcium signals, indicating recruitment of the SC into the seizure network. Furthermore, we selectively ablated SC neurons and this significantly attenuated hippocampal seizure-induced ZI activation (revised Supplementary Figure 15H-K). These findings indicate that the SC may act as a potential upstream relay, receiving hippocampal seizure input and transmitting it to the ZI. Although the SC contains both major GABAergic and minor glutamatergic neurons, prior studies have shown that SC GABAergic neurons can facilitate downstream neuronal activity via local disinhibitory microcircuits or other co-released excitatory neurotransmitter (Liu et al., 2022; Schmidt et al., 2001). Consistent with this framework, our data support a model in which SC neurons indirectly promote ZI GABAergic activity, positioning the SC as an important upstream regulator of ZI calcium dynamics during hippocampal kindling-induced seizures.

Accordingly, we have revised the manuscript in the discussion section and marked them in red (Page 14, Lines 395-404).

(2) We further examined the relationship between hippocampal stimulation intensity and ZI calcium activity. Increasing stimulation currents (100, 200, and 400 μA) produced a graded increase in ZI calcium signals (revised Supplementary Figure 4E-H). Accordingly, we have revised the manuscript in the results section and marked them in red (Page 7, Lines 188-191).

Revised Supplementary Figure 15.

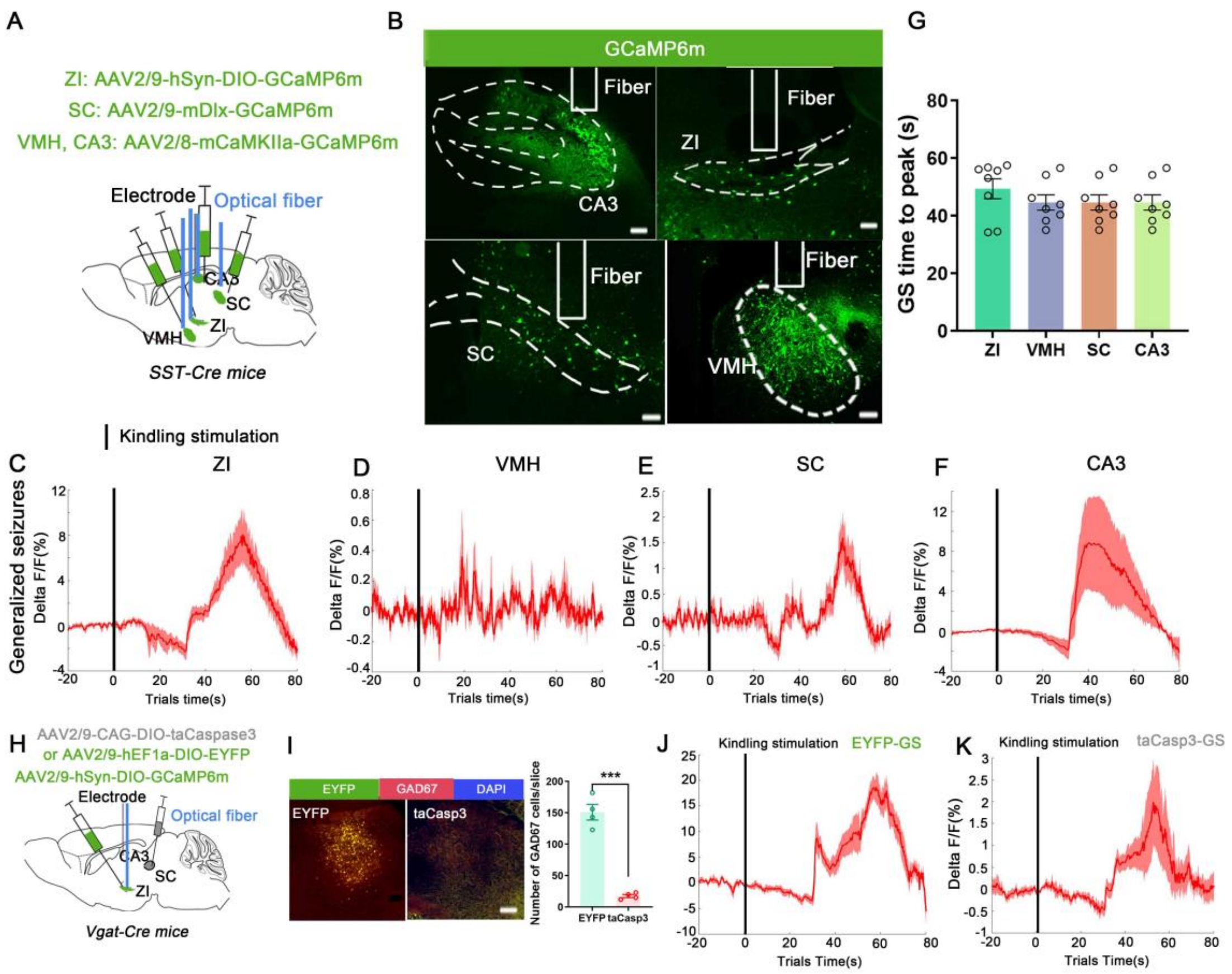

Revised Supplementary Figure 4E-H:

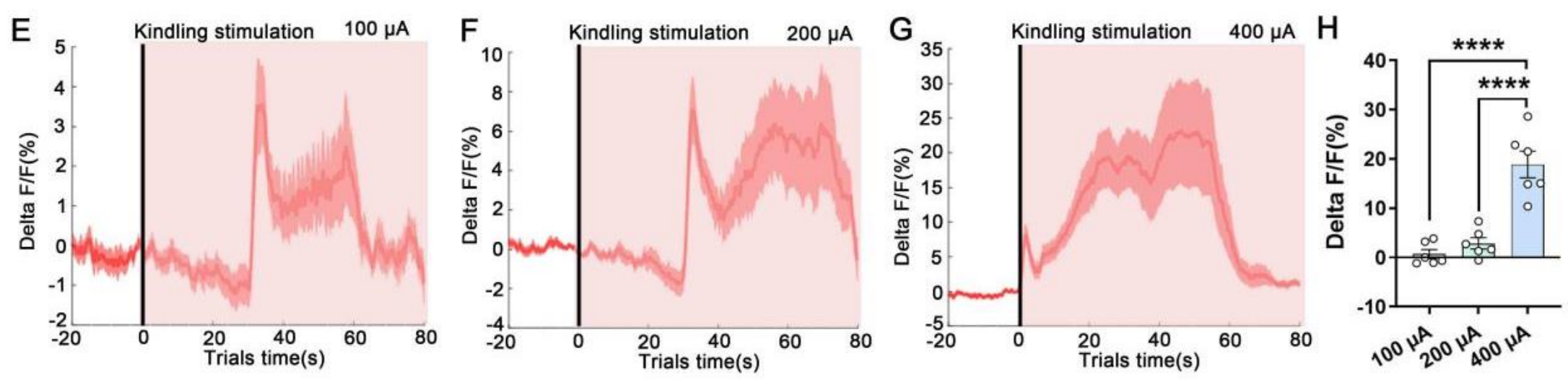

Page 14, Line 395-404: Given that the ZI does not receive direct hippocampal inputs, we examined whether its recruitment during seizures is mediated by upstream structures. The superior colliculus (SC) as a key node, as it receives broad inputs and projects densely to the ZI (Arena et

al., 2024; Power et al., 2001). In our model, we observed that kindling-induced seizure activates SC neurons, accompanied by concurrent increases in the activity of CA3, ZI and VMH (Supplementary Fig. 15A-G), reflecting seizure signals propagated rapidly across the network. Selective ablation of SC neurons significantly attenuated hippocampal seizure-induced ZI activation (Supplementary Fig. 15H-K), suggesting that the SC acts as a critical upstream relay, receiving hippocampal seizure input and transmitting it to the ZI.

Page 7, Lines 188-191: Meanwhile, we investigated whether different stimulation intensities affected calcium signals in the ZI, and found that as the stimulation intensity increased, the calcium signals in the ZI increased accordingly (Supplementary Fig. 4E-H).

2. c-Fos staining shows a decrease in the number of activated ZI cells after seizures. Are these activated cells co-labelled with SST or PV neurons, and what is the exact proportion?

Reply: Thank you for your valuable comments. We performed double immunofluorescence staining for PV or SST with c-Fos in neurons activated after seizures in the ZI. In response to comments from other reviewers, we also co-stained VGLUT2 and nNOS with c-Fos during hippocampal seizures, as shown in the revised Supplementary Figure 3. Quantitative analysis revealed that overall, 23.6% of activated neurons were PV^+, while 48.9% were SST^+, indicating SST^+ GABAergic neurons are much more easily recruited in seizure.

Interestingly, there is spatial differences in activation pattern: seizure-activated PV^+ c-Fos neurons were more abundant in the ZIv (45.8%) than in the ZIr (11.6%), whereas seizure-activated SST^+ c-Fos neurons were more prevalent in the ZIr (56.6%) than in the ZIv (30.4%). Hippocampal seizures also activated 15.2% of nNOS neurons in the ZIr, without affecting VGLUT2 neurons. These findings indicate that SST^+ neurons are activated across both ZIr and ZIv, suggesting that SST^+ neurons may play a more critical role in hippocampal seizure activity among these subtypes. Accordingly, we have revised the results section to reflect these findings and marked the changes in red (Page 6, Lines 164-174).

Revised Supplementary Figure 3:

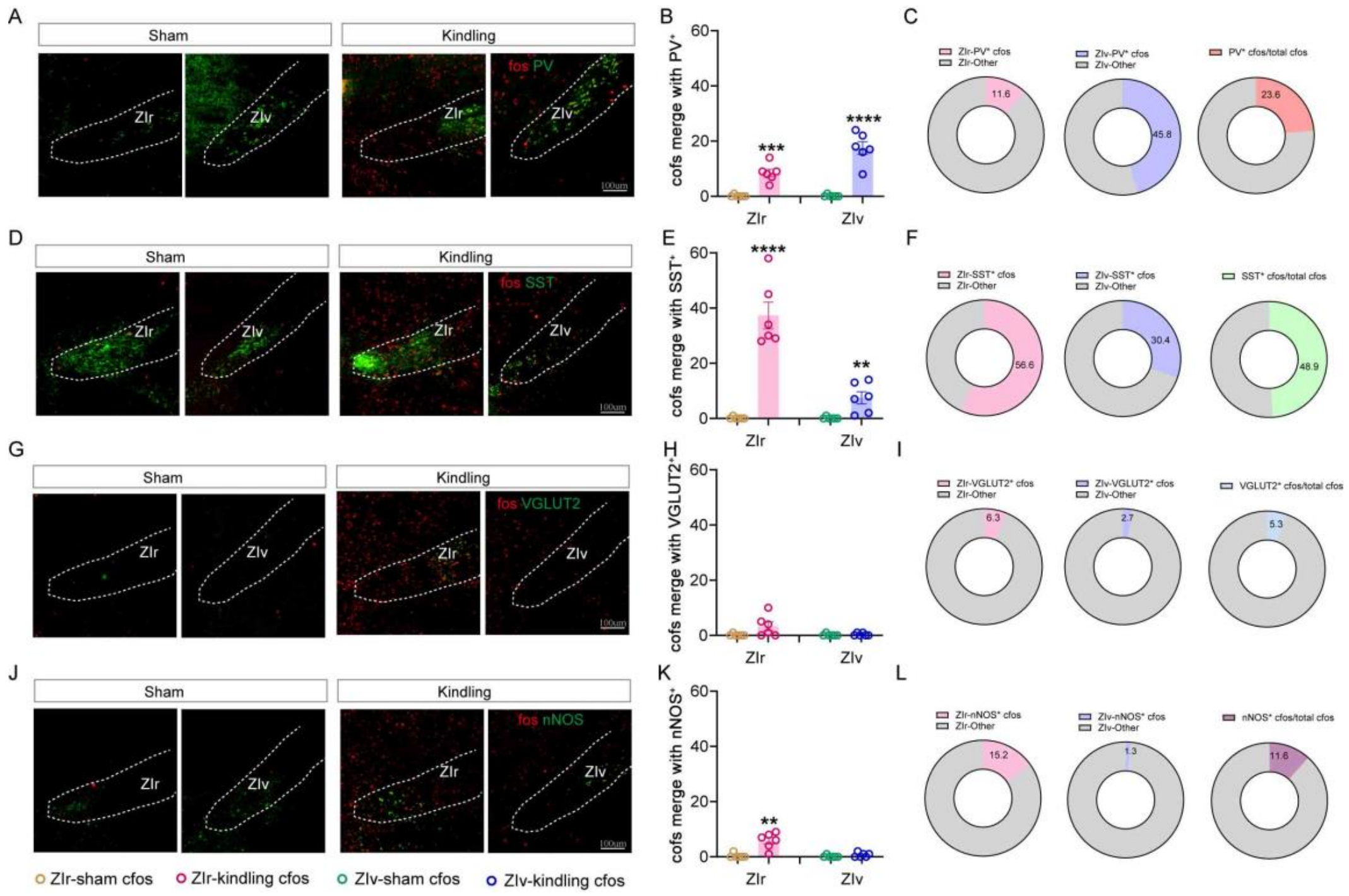

Page 6, Lines 164-174: Firstly, we performed double immunofluorescence staining for PV or SST in neurons activated after seizures in the ZI. The quantitative analysis revealed that overall, 23.6% of the seizure activated neurons were identified as PV^+, while 48.9% were SST^+ (Supplementary Fig. 3A-F), indicating SST^+ GABAergic neurons are much more easily recruited in seizure. Interestingly, c-Fos^+ cells co-localized with the PV^+ or SST^+ neuron subsets represent spatial variation: the c-Fos^+ PV neurons were more distributed in the ZIv (45.8%) than ZIr (11.6%), however, the c-Fos^+ SST^+ neurons were more distributed in the ZIr (56.6%) than ZIv (30.4%) (Supplementary Fig. 3A-F). Hippocampal seizure also activated 15.2% of nNOS neurons in the ZIr during seizure without affecting VGLUT2 neurons (Supplementary Fig. 3G-L).

3. As a neuropeptide transmitter, does somatostatin (SST) itself mediate the seizure-modulating effect of the ZIr SST–VMH circuit?

Reply: We appreciate your valuable comment. To directly test whether neuropeptide somatostatin (SST) is required for the seizure-modulating effect of the ZI^{SST}-VMH circuit, we performed an additional set of pharmacological experiments during optogenetic activation ZI^{SST}-VMH circuit. Specifically, when the ZI^{SST}-VMH circuit was optogenetically activated, bicuculline ($GABA_A$ receptor antagonist) or CYN-154806 (somatostatin receptor antagonist) was administered to the VMH, while the control group received saline, as shown in the revised Supplementary Fig. 11. Compared with the blue-light group, bicuculline mainly reversed later phase of kindling-induced seizure development, as evidenced by a significant reduction in number of stimulations required to stay in stages 3-4, while CYN-154806 reversed the whole phase of kindling-induced seizure development, significantly reducing the number of stimulations required to stay in both seizure stages 0-2 and stages 3-4. Antagonizing SST receptors locally also reproduced a pro-seizure effect in after-discharge duration. These results indicate that both $GABA_A$ receptors and somatostatin receptors may contribute to antiseizure effect of the ZI^{SST}-VMH circuit. Accordingly, we have revised the manuscript in the results section and marked them in red (Page 11, Lines 292-306).

Revised Supplementary Figure 11

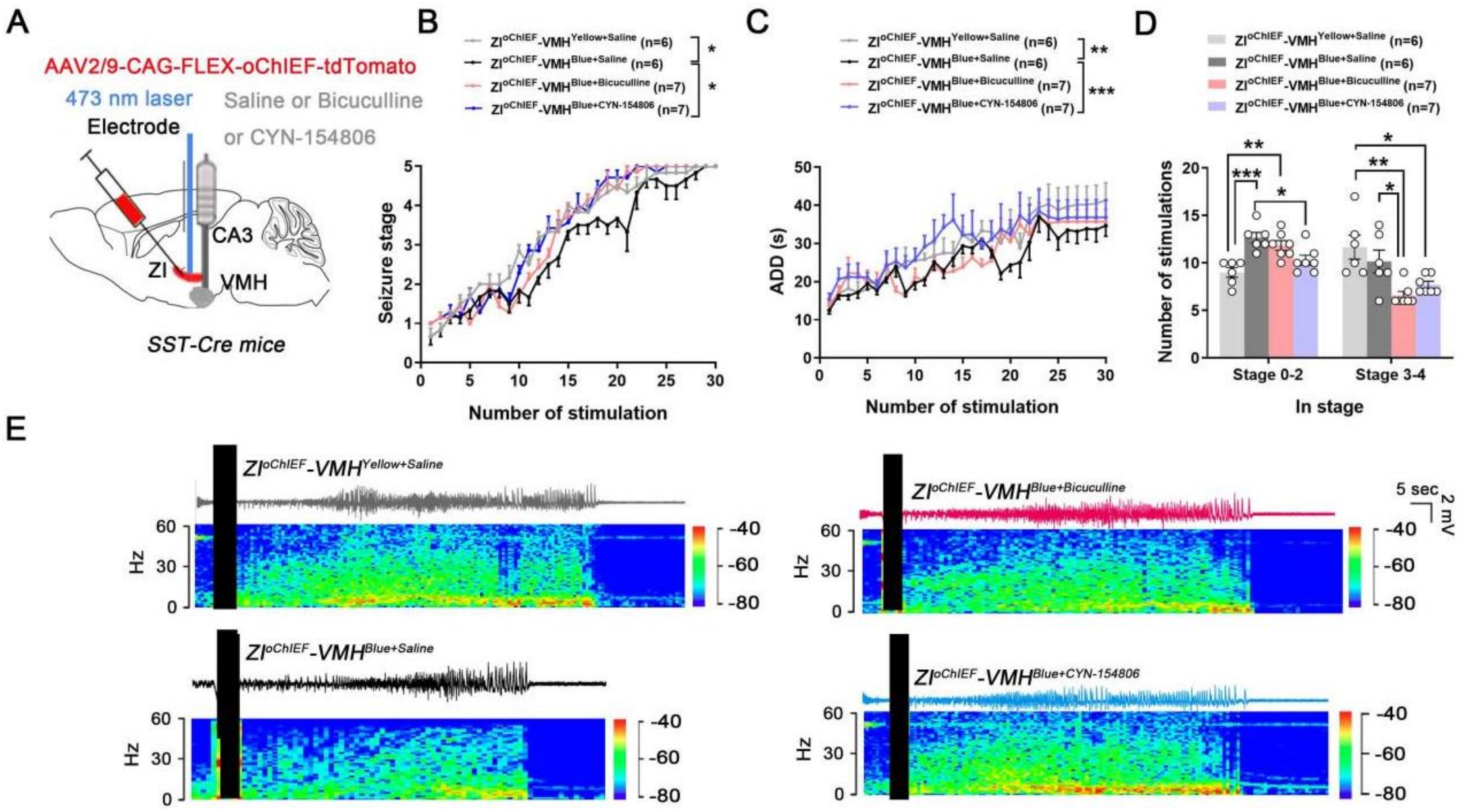

Page 11, Lines 292-306: To directly test whether GABA or neuropeptide somatostatin (SST) is required for the seizure-modulating effect of the ZI^{SST}-VMH circuit, we performed a set of pharmacological experiments during optogenetic activation ZI^{SST}-VMH circuit. Specifically, when the ZI^{SST}-VMH circuit was optogenetically activated, bicuculline ($GABA_A$ receptor antagonist) or CYN-154806 (somatostatin receptor antagonist) was administered to the VMH, while the control group received saline, as shown in the Supplementary Fig. 11. Compared with the blue-light group, bicuculline mainly reversed later phase of kindling-induced seizure development, as evidenced by a significant reduction in number of stimulations required to stay in stages 3-4, while CYN-154806 reversed the whole phase of kindling-induced seizure development, significantly reducing the number of stimulations required to stay in both seizure stages 0-2 and stages 3-4. Antagonizing SST receptors locally also reproduced a pro-seizure effect in after-discharge duration. These results indicate that both $GABA_A$ receptors and somatostatin receptors may contribute to anti-seizure effect of the ZI^{SST}-VMH circuit.

4. Increased evidence is showing that ZI different sectors ranging from anterior to posterior may function differently. Could stimulation of other sectors a different bregma site produce an even stronger phenotype?

Reply: We appreciate your valuable suggestion. We reanalyzed the effects of optical fiber placement in different regions of the ZI in the optogenetic activation experiments. The results show that optical fibers targeting the anterior ZI, where most of the region was illuminated, produced a significant effect on seizure activity, whereas fibers placed at the edges of the ZI had a comparatively smaller impact on seizure behavior. Accordingly, we have revised the manuscript in the results section and marked them in red (Page 6, Lines 142-147).

Revised Supplementary Figure 2A-C:

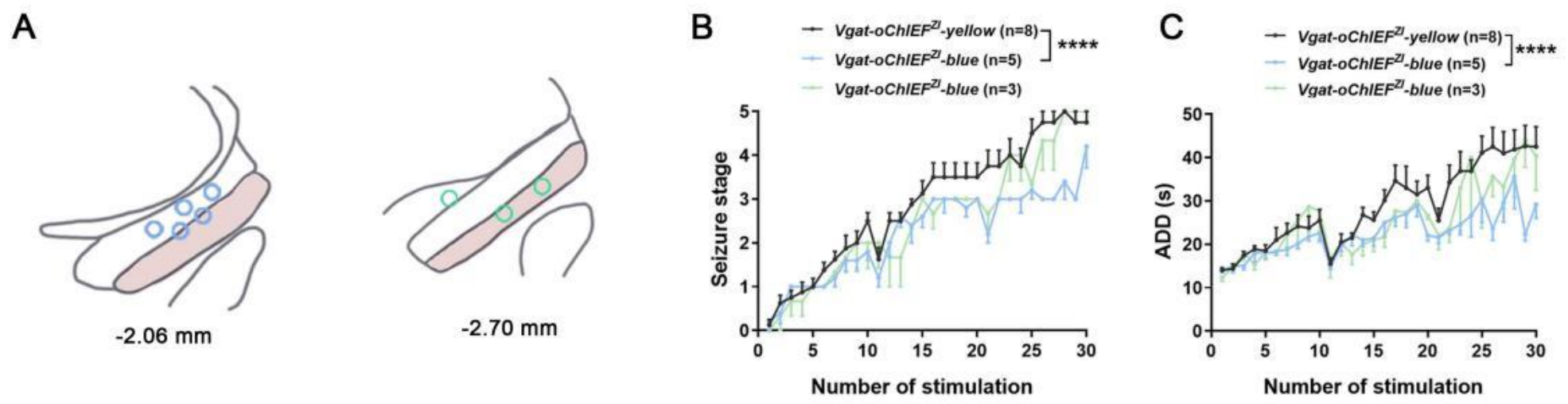

Page 6, Lines 142-147: Interestingly, the optical fiber placed in the anterior part of ZI (covering most of the area) showed a significant impact on the seizure activity, while the optical fiber placed at the edge of ZI showed a relatively weaker effect on the seizure behavior (Supplementary Fig. 2A-C). This indicates that the ZI subregion may differently affects the severity of epileptic seizures, which may be related with the different location of subtype GABAergic neuron in the ZI (Mirofanis et al., 2004).

Minor concerns:

The label of y axis in Fig1P is wrong and should be ADD(s).

Fig1O-P, the statistical marker star should be given a range, other similar cases should be also pay attention to.

Row 166 The hyphen in "Chan-nelrhodopsin 2 (ChR2)" should be removed.

Reply: Thanks very much for these comments.

(1) We have revised the label of y axis “Seizure stages” to “ADD(s)” in revised Figure 1P.

(2) We have clearly defined the statistical significance markers in the revised figure legends (*P < 0.05, **P < 0.01, ***P < 0.001, and ****P < 0.0001). Furthermore, we have carefully reviewed all statistical figures throughout the manuscript and supplementary materials to ensure consistent application of these definitions.

(3) We have revised “Chan-nelrhodopsin 2 (ChR2)” to “ Channelrhodpsin2 (ChR2)” in the revised manuscript and marked them in red (Page 7, Line 195).

Reviewer #2

The manuscript entitled "Zona Incerta Somatostatin+ Circuit Attenuates Seizures Progression" investigates the role and neural circuit mechanisms of somatostatin (SST)-positive neurons in the zona incerta (ZI) during seizure progression in a hippocampal kindling model. Using optogenetics combined with fiber photometry calcium imaging, behavioral analysis, and EEG recordings, the authors demonstrate that ZI SST neurons, but not parvalbumin (PV)-positive neurons, bidirectionally modulate the progression of seizures in this model. Additionally, they identify ventromedial hypothalamus (VMH) glutamatergic neurons as a potential downstream target of ZI SST neurons in seizure progression. While the study addresses an important topic, I have several concerns regarding the results presented in this manuscript.

Reply: We sincerely appreciate the time and effort you have taken to review our manuscript and for the valuable comments, which have significantly contributed to the revision and improvement of our paper. In this letter, we address each of your comments individually, followed by our corresponding responses. For clarity, we have restated your comments in bold font and provided our responses in blue.

Major points:

1.The study employs the hippocampal kindling model to simulate epilepsy. This model is induced by electrically stimulating the hippocampal CA3 region, indicating that the seizure onset occurs in the hippocampus. The authors have chosen the zona incerta (ZI) as a potential target region for modulating seizure progression, which is a reasonable choice. However, they need to provide evidence demonstrating how the seizure activity spreads from the hippocampus to the ZI and clarify the exact role of the ZI during seizure progression. The same applies to the downstream region of the ZI, the ventromedial hypothalamus (VMH). The authors should conduct additional experiments or analyses to show the propagation of seizure activity from the hippocampus to the ZI and subsequently to the VMH. For example, simultaneous recording of GCaMP signals in the hippocampus, ZI, and VMH could be performed. Additionally, analyzing the correlation between GCaMP and EEG signals during optogenetic activation of ZI SST neurons at different seizure stages would provide valuable insights.

Reply: We appreciate your valuable and constructive comments.

(1) In accordance with your suggestion, we simultaneously recorded the GCaMP calcium signals in the hippocampal CA3, ZI, and VMH, as well as the superior thalamus (SC)- the possible connection between CA3 and ZI in the revised Supplementary Figure 15A-G. During hippocampal CA3 kindling, we observed a simultaneous and significant increase in calcium activity across all four recorded regions (CA3, SC, ZI, and VMH). Crucially, our new data indicate that seizure-associated calcium activity did not exhibit a clear, stereotypical typical sequential propagation. Instead, a significant, concurrently increase in calcium signals in all four regions was observed. Furthermore, as noted by Reviewer 1, ventral ZI neurons are densely interconnected with the SC (Arena et al., 2024; Power et al., 2001), supporting a potential role for the SC as an upstream node in seizure-related circuitry. To test this hypothesis, we found that ablation of SC neurons resulted in a reduction of calcium signals in the ZI (revised Supplementary Figure 15H-K), suggesting that the SC is a significant upstream source of neural input to the ZI.

Accordingly, we have revised the manuscript in the discussion section and marked them in red (Page 14, Lines 395-404).

(2) In addition, we analyzed the correlation between the calcium signal of ZI SST neurons and the electroencephalogram (EEG) signal under different seizure stages, as well as ADDs in the revised Supplementary Figure 4. The result shows that calcium signal (GCaMP) of ZI SST neurons was positively correlated with the seizure stage and ADDs. Accordingly, we have revised the manuscript in the results section and marked them in red (Page 7, Lines 186-188).

Revised Supplementary Figure 15:

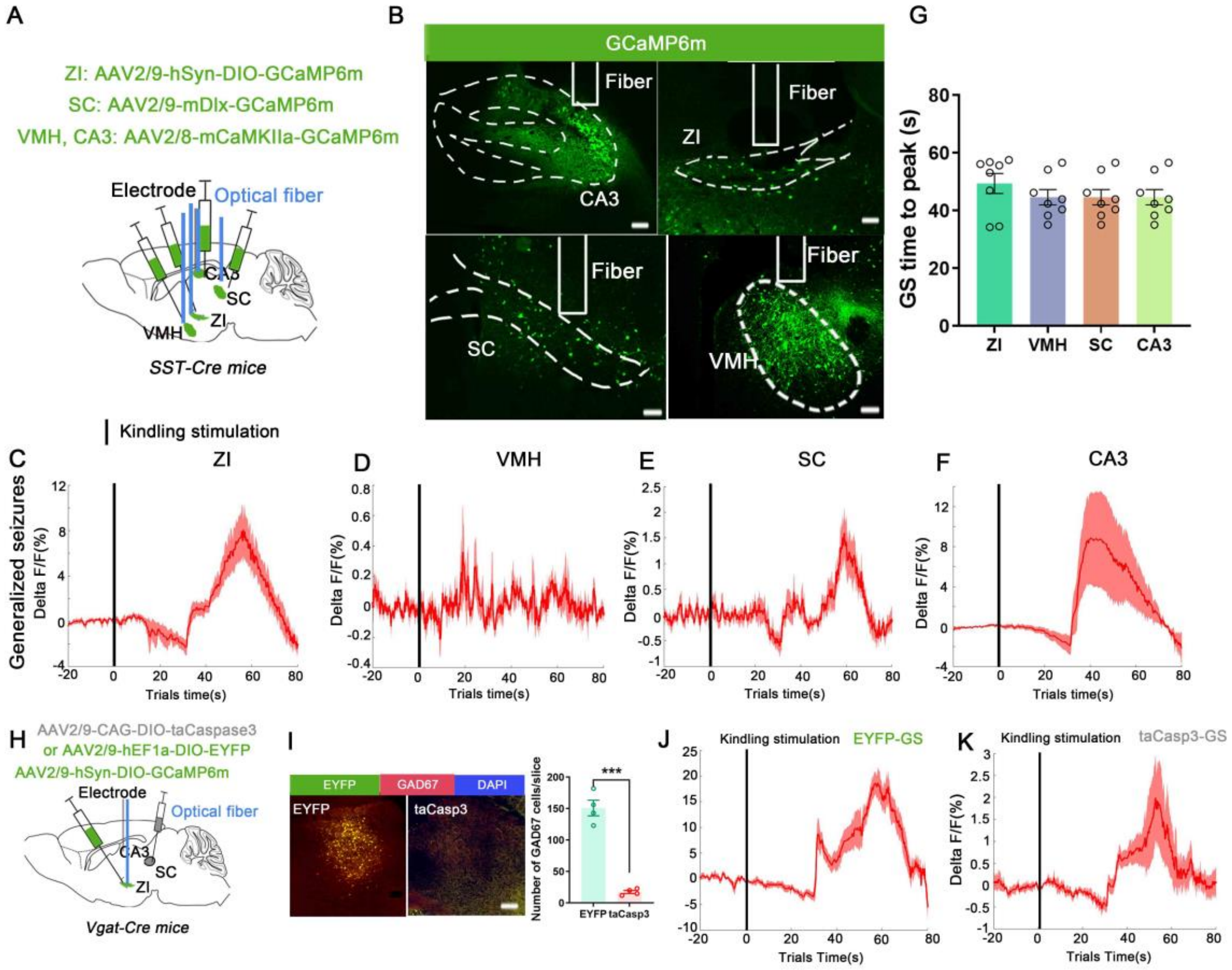

Revised Supplementary Figure 4C, D:

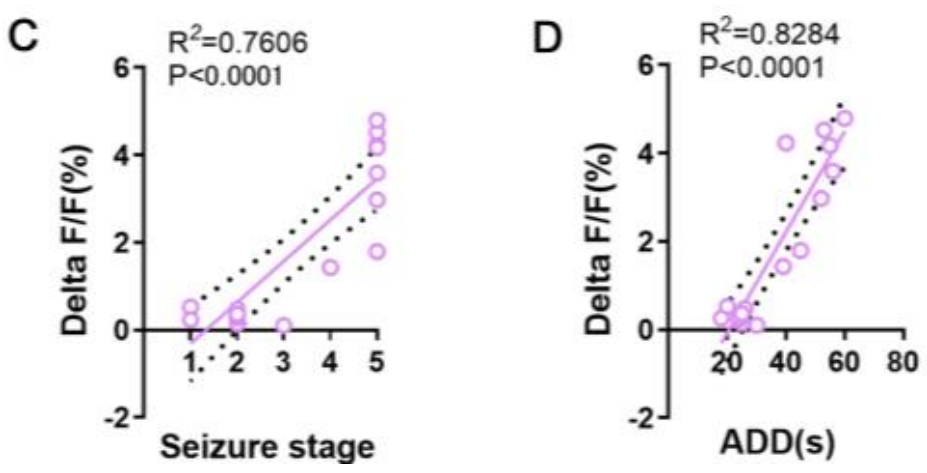

Page 14, Lines 395-404: Given that the ZI does not receive direct hippocampal inputs, we examined whether its recruitment during seizures is mediated by upstream structures. The superior colliculus (SC) as a key node, as it receives broad inputs and projects densely to the ZI (Arena et al., 2024; Power et al., 2001). In our model, we observed that kindling-induced seizure activates SC neurons, accompanied by concurrent increases in the activity in CA3, ZI and VMH (Supplementary Fig. 15A-G), reflecting seizure signals propagated rapidly across the network.

Selective ablation of SC neurons significantly attenuated hippocampal seizure-induced ZI activation (Supplementary Fig. 15H-K), suggesting that the SC acts as a critical upstream relay, receiving hippocampal seizure input and transmitting it to the ZI.

Page 7, Lines 186-188: The changed calcium signals of SST^+ neurons have a significant correlation with the seizure stage and ADD of hippocampal seizures (Supplementary Fig. 4C and D).

2. line98-99: It is necessary to first introduce how to induce hippocampal seizure and how to determine the starting point of calcium signals；a co-labeling staining and quantitative analysis of neurons of different types in ZI and fos should be added.

Reply: We appreciate your valuable suggestion!

(1) The kindling-induced hippocampal seizure model was constructed by implantation of stimulating electrodes in the ventral hippocampus (CA3) of mice and connection to an amplifier and a constant current stimulator. Delivering a 20 Hz current for 2 s (1 ms pulses, 0.4 mA), triggers discharges to induce hippocampus seizure (Farrell et al., 2020; Fei et al., 2022; McNamara et al., 1980). Regarding the starting point of the calcium signals, we marked the start of the hippocampal-kindling event as 0 s. All subsequent fluorescence data were aligned to this reference point. We have revised the manuscript in the results section and marked them in red (Page 4, Lines 95-99; Page 23, Lines 648-650).

(2) We performed double immunofluorescence staining for PV or SST with c-Fos in neurons activated after seizures in the ZI. In response to comments from other reviewers, we also co-stained VGLUT2 and nNOS with c-Fos during hippocampal seizures, as shown in the revised Supplementary Figure 3. Quantitative analysis revealed that overall, 23.6% of activated neurons were PV^+, while 48.9% were SST^+, indicating SST^+ GABAergic neurons are much more easily recruited in seizure. Interestingly, there is spatial differences in activation pattern: seizure-activated PV^+ c-Fos neurons were more abundant in the ZIv (45.8%) than in the ZIr (11.6%), whereas seizure-activated SST^+ c-Fos neurons were more prevalent in the ZIr (56.6%) than in the ZIv (30.4%). Hippocampal seizures also activated 15.2% of nNOS neurons in the ZIr, without affecting VGLUT2 neurons. These findings indicate that SST^+ neurons are activated across both ZIr and ZIv, suggesting that SST^+ neurons may play a more critical role in hippocampal seizure activity among these subtypes. Accordingly, we have revised the manuscript in the results section and marked them in red (Page 6, Lines 164-174).

Revised Supplementary Figure 3:

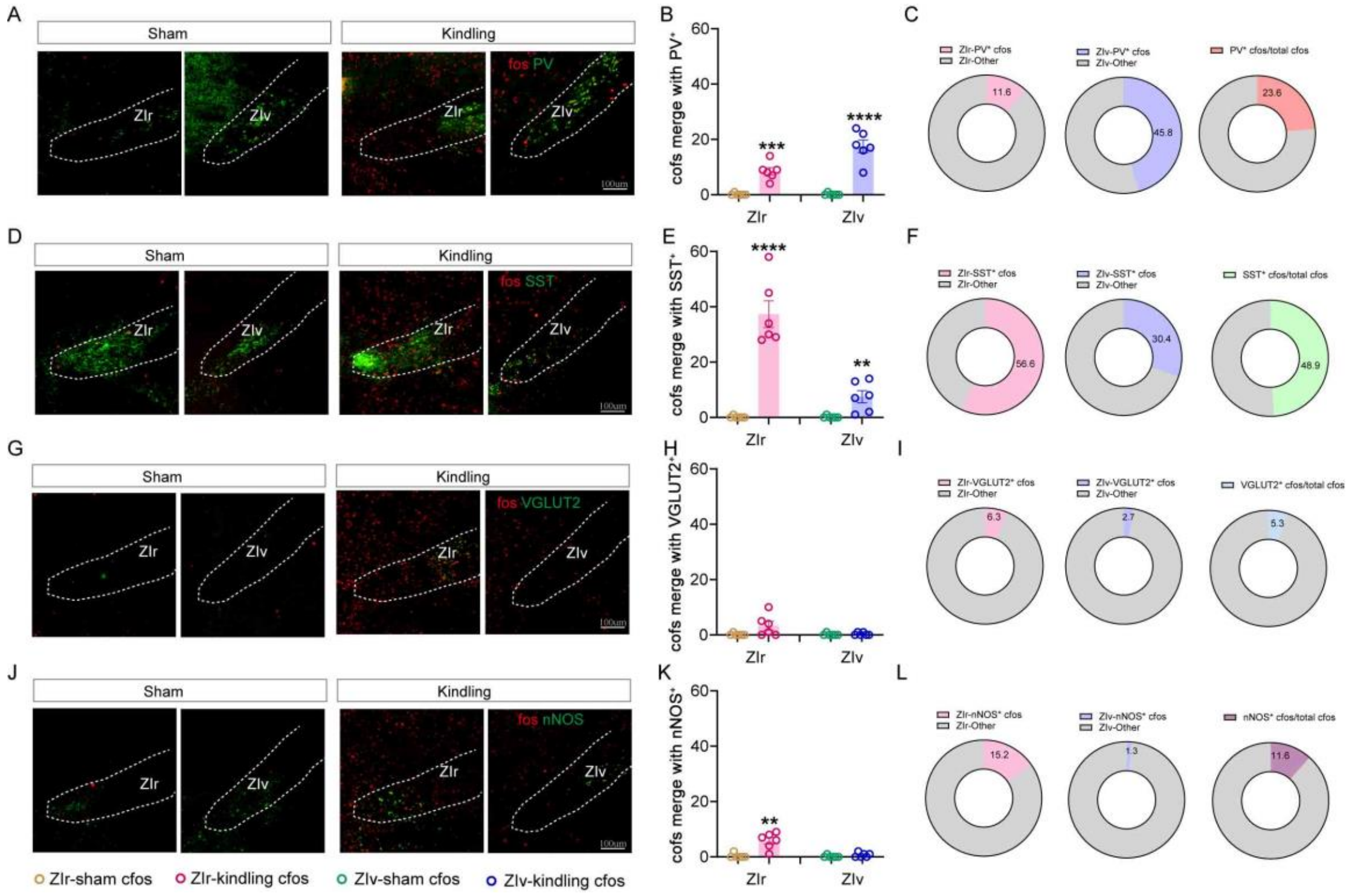

Page 4, Lines 95-99: The model involves long-term implantation of stimulating electrodes in the ventral hippocampus (CA3) of mice and connection to an amplifier, allowing current to flow out from a constant current stimulator. Through a current of 20 Hz for 2 s (1 ms pulses, 0.4 mA), triggers discharges to induce hippocampus seizure (Farrell et al., 2020; Fei et al., 2022; McNamara et al., 1980).

Page 23, Lines 648-650: For data analysis, we marked the start of the hippocampal-kindling event as 0 s. All subsequent fluorescence data were aligned to this reference point.

Page 6, Lines 164-174: Firstly, we performed double immunofluorescence staining for PV or SST in neurons activated after seizures in the ZI. The quantitative analysis revealed that overall, 23.6% of the seizure activated neurons were identified as PV^+, while 48.9% were SST^+ (Supplementary Fig. 3A-F), indicating SST^+ GABAergic neurons are much more easily recruited in seizure. Interestingly, c-Fos^+ cells co-localized with the PV^+ or SST^+ neuron subsets represent spatial variation: the c-Fos^+ PV neurons were more distributed in the ZIv (45.8%) than ZIr (11.6%), however, the c-Fos^+ SST neurons were more distributed in the ZIr (56.6%) than ZIv (30.4%) (Supplementary Fig. 3A-F). Hippocampal seizure also activated 15.2% of nNOS neurons in the ZIr during seizure without affecting VGLUT2 neurons (Supplementary Fig. 3G-L).

3. How is the expression of fos in other brain regions during kindling-induced hippocampal seizure? It is hoped that the author will add this part of the results to reflect the importance of Zi in this model.

Reply: We appreciate your valuable suggestion. Following electrically kindling-induced seizures, brain regions such as the hippocampus, cortex, basal nuclei, basolateral amygdala, piriform cortex, medial thalamus, prefrontal cortex, and striatum exhibit an increase in c-Fos expression (Dragunow

and Robertson, 1987; Qi et al., 2022; Watanabe et al., 1996), indicating a relatively widespread pattern of activation. More importantly, our previous study comprehensively described widespread c-Fos expression across multiple brains, including the hippocampus, thalamus and ZI, which constitute the primary focus of the present study (Cheng et al., 2023). We specifically focused on the ZI for two main reasons. First, hippocampal kindling-induced seizures lead to a marked increase in c-Fos expression in the ZI, indicating its active engagement in the seizure network. Second, as a powerful inhibitory 'hub' located distant from the seizure focus (hippocampus), the ZI can exert broad GABAergic control over multiple brain regions, including the cerebral cortex, thalamus, and brainstem, highlighting its potential role in modulating seizure activity (Mitrofanis, 2005). In epileptic condition characterized by global network hyperexcitability, the ZI may be recruited or modulated as a critical "braking" system. However, the precise mechanisms by which the ZI regulates epileptic activity remain elusive, making their elucidation a principal objective of this study. To introduce this background about widespread c-Fos expression across multiple brains, we have added references and descriptions from literatures on the activation of other brain regions during seizures (Page 3, Lines 72-73).

Page 3, Lines 72-73: More importantly, our previous study also described widespread c-Fos expression across multiple brains including hippocampus, thalamus and ZI (Cheng et al., 2023).

4. While both ZI PV and SST neurons are activated during seizures, only SST neurons bidirectionally modulate seizure progression. However, the manuscript lacks mechanistic explanations for this difference. Additional experiments are necessary to elucidate why SST neurons, but not PV neurons, exhibit bidirectional control.

Reply: We sincerely appreciate your insightful suggestion. In response to this comment, we have conducted a comparative analysis of the downstream projection patterns of ZI SST$^+$ and PV$^+$ neurons, aimed at elucidating a potential different circuit-level mechanism. We discovered a striking anatomical dissociation: ZI SST$^+$ neurons project predominantly to forebrain regions, such as the VMH, hippocampus, anteroventral thalamic nucleus, etc., which are involved in integrating in cognitive, emotional and homeostatic centers (Wang et al., 2022; Eichenbaum, 2004; Hintiryan et al., 2025). Whereas ZI PV$^+$ neurons primarily innervate posterior parts of brain, such as the reticular nucleus of the thalamus and the reticular structure of the brainstem (Revised Supplementary Figure 16), which are implicated in sensory, motor and arousal centers. This fundamental difference in efferent connectivity provides an anatomical substrate for their distinct functional roles. Although the exact circuit mechanism of ZI PV$^+$ neuron in seizure is not clear, our study is the first to reveal the pivotal role of the VMH mediating anti-seizure effect of ZI SST$^+$ neuron. Furthermore, the c-Fos stanning results also indicated that 23.6% of the seizure-activated c-Fos were identified as PV$^+$, while 48.9% were SST$^+$ (Revised Supplementary Figure 3), suggesting that SST$^+$ neurons may play a more critical role in hippocampal seizure. Together, these data provide evidences to explains the different role of PV and SST neurons in hippocampal seizure and their downstream circuit mechanism. Accordingly, we have revised the manuscript in the discussion section and marked them in red (Page 16, Lines 443-459).

Revised Supplementary Figure 16:

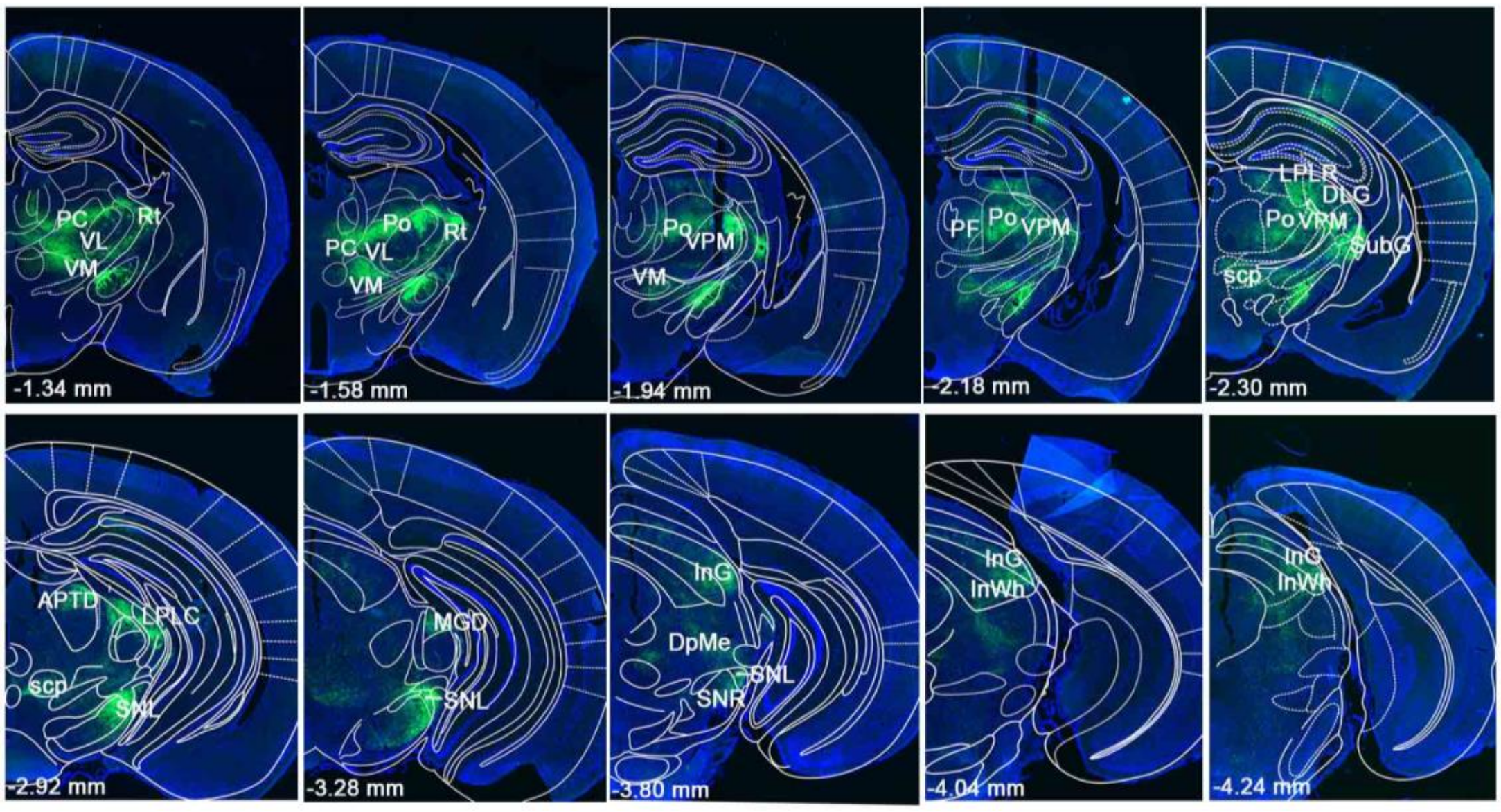

Revised Supplementary Figure 3:

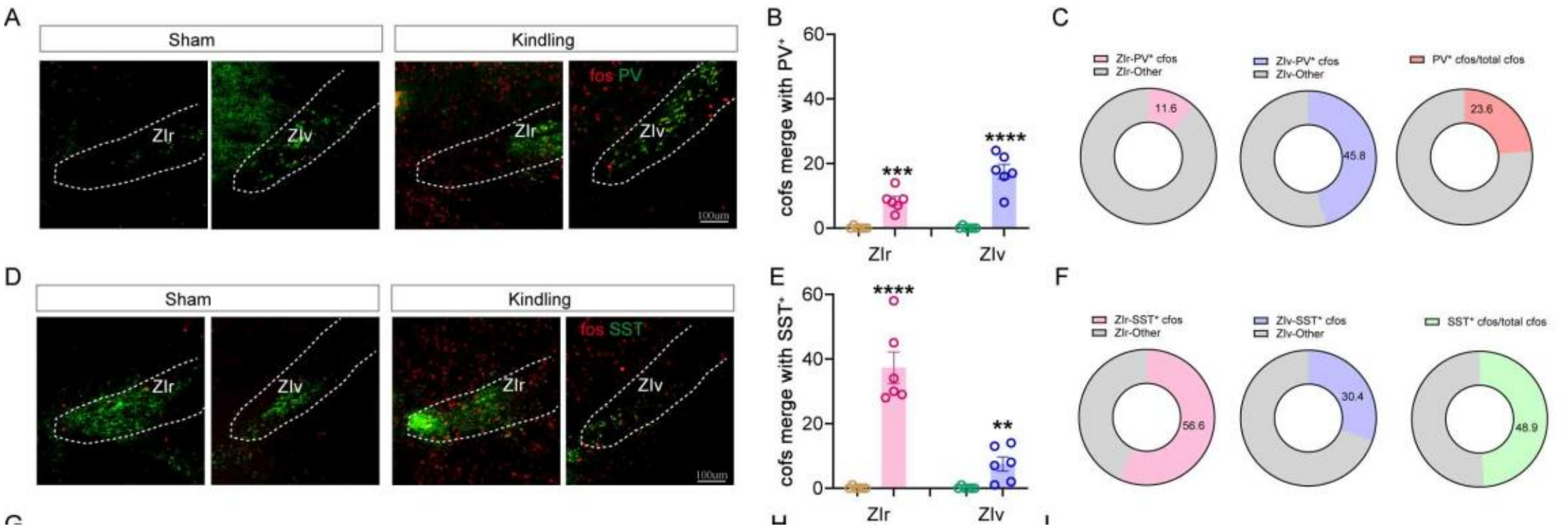

Page 16, Lines 443-459: A primary factor underlying this functional divergence is likely the differential efferent connectivity of ZI SST$^+$ and PV$^+$ GABAergic neurons. Using anterograde tracing, we found that ZI SST$^+$ neurons project predominantly to anterior brain regions, such as the VMH, hippocampus, anteroventral thalamic nucleus, etc., which are involved in integrating cognitive, emotional and homeostatic centers (Wang et al., 2022; Eichenbaum, 2004; Hintiryan et al., 2025). Whereas ZI PV$^+$ neurons primarily innervate posterior areas, such as the reticular nucleus of the thalamus and the reticular structure of the brainstem (Supplementary Figure 16), which are implicated in sensory, motor and arousal centers. This fundamental difference in efferent connectivity provides an anatomical substrate for their distinct functional roles. Although the exact circuit mechanism of ZI PV$^+$ neuron in seizure is not clear, our study is the first to reveal the pivotal role of the VMH mediating anti-seizure effect of ZI SST$^+$ neuron. In addition, the c-Fos stanning results also indicated that SST$^+$ GABAergic neurons are much more easily recruited in seizure. Together, these data provide evidences to explains the different role of PV$^+$ and SST$^+$ neurons in hippocampal seizure, SST$^+$ GABAergic neurons predominantly mediate the primary GABAergic control of seizures within the ZI.

5. Fiber photometry data indicate that ZI PV and SST neurons are activated during seizures; however, optogenetic activation of these neurons inhibits seizure progression. This apparent contradiction requires clarification. The authors should address and reconcile these observations through further analysis.

Reply: We appreciate your insightful comments. Previous study has reported that multiple interneuron subtypes, including PV and SST, are rapidly recruited at seizure onset, exhibiting significant calcium activity during seizure (Khoshkhoo et al., 2017). In addition, it has been found that selective optogenetic activation of inhibitory interneurons can suppress ongoing epileptiform activity and shorten seizure duration, supporting an intrinsic anti-seizure role for GABAergic neurons when artificially enhanced (Ledri et al., 2014). In the present study, the activation of ZI PV^+ and SST^+ neurons during seizure likely reflects the recruitment of the brain's endogenous anti-seizure mechanisms, which, however, are not sufficient to fully prevent seizure occurrence. Supporting this idea, optogenetic activation of PV^+ and SST^+ neurons artificially enhances this intrinsic inhibitory activity, thereby reducing seizure stage and ADD, although it does not completely block seizure onset. These results indicate that as inhibitory neurons, PV^+ and SST^+ neurons possess an intrinsic anti-seizure role. Accordingly, we have revised the manuscript in the discussion section and marked them in red (Page 15, Lines 427-436).

Page 15, Lines 427-436: It is worth noting that, the activation of ZI PV^+ and SST^+ neurons during seizure likely reflects an endogenous anti-seizure mechanism that is engaged but insufficient to terminate seizures. As support, optogenetic activation of ZI PV^+ and SST^+ GABAergic neurons delays, but does not fully abort seizure development, indicating that the ZI acts as a "regulator" of seizure development. In the hippocampal kindling model, where repeated stimulation chronically enhances seizure susceptibility, this 'auxiliary inhibitory' role likely reflects a compensatory mechanism, operating alongside other parallel inhibitory systems that are recruited to finally stop seizure (Wu and Leung, 1997), while ZI activity alone is insufficient to prevent seizure progression.

6. Optogenetic activation of ZI GABAergic neurons does not completely terminate seizures, suggesting that their role may be auxiliary rather than central. The authors should discuss the functional significance of ZI GABAergic neurons within the hippocampal kindling model and consider potential compensatory mechanisms that might influence seizure modulation.

Reply: We appreciate your valuable comment. Our results indeed suggest that ZI GABAergic neurons may not be the core switch responsible for directly "turning off" seizures. Rather than fully preventing seizures, ZI neurons act as an endogenous anti-seizure "network regulator" delaying seizure spread. In the hippocampal kindling model, repeated stimulation progressively increases seizure susceptibility, and the ZI likely represents a compensatory inhibitory mechanism that partially counteracts epileptogenesis. Similar endogenous inhibitory systems may also cooperate with the ZI to modulate seizure activity (Chou et al., 2018; Yang et al., 2022; Chen and Kriegstein, 2015). Accordingly, we have revised the manuscript in the discussion section and marked them in red (Page 15, Lines 429-436).

Page 15, Lines 429-436: As support, optogenetic activation of ZI PV^+ and SST^+ GABAergic neurons delays, but does not fully abort seizure development, indicating that the ZI acts as a

“regulator” of seizure development. In the hippocampal kindling model, where repeated stimulation chronically enhances seizure susceptibility, this ‘auxiliary inhibitory’ role likely reflects a compensatory mechanism, operating alongside other parallel inhibitory systems that are recruited to finally stop seizure (Wu and Leung, 1997), while ZI activity alone is insufficient to prevent seizure progression. .

7. line160-162：PV+ neurons exhibited a gradual but sustained increase in calcium activity, whereas SST+ neurons displayed a delayed but rapid surge in calcium signals (Fig. 2D and N). This definition is rather vague. It is hoped that the author will make further comparisons before drawing relevant conclusions.

Reply: We appreciate your valuable comment. We recalculated the average ascending slopes of calcium signals in PV^+ neurons and SST^+ neurons to quantify the initial dynamic characteristics of the activities of the two types of neurons. The calcium signal of PV^+ neurons showed a gentle upward trend, with an upward slope of 0.2032 ± 0.0509, while the calcium signal of SST^+ neurons exhibited a steeper upward process, with an upward slope of 0.3625 ± 0.0674. Accordingly, we have revised the manuscript in the results section and marked them in red (Page 7, Lines 182-186).

Revised Supplementary Figure 4B:

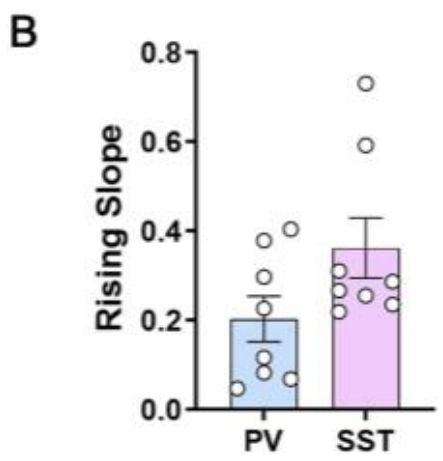

Page 7, Lines 182-186: Further analysis of the calcium signal duration of PV^+ and SST^+ neurons during hippocampal seizure revealed that SST^+ neurons tended to exhibit a faster increase in calcium signals with a slightly longer duration, as indicated by the steeper slope in SST during GS compared to these of PV^+ neurons (Supplementary Fig. 4A and B).

8. In Figure2, the optogenetic results should also be compared between PV and SST to reflect the differences in their roles.

Reply: We appreciate your valuable suggestion. A direct comparison of the effects of optogenetic activation or inhibition of ZI PV^+ and SST^+ neurons was performed, with emphasis on their differential efficacy in delaying seizure progression by analyzing behavioral parameters including ΔSeizure stage, and ΔADD indicating the relative change than their respective control groups in the revised Supplementary Figure 8. Compared with PV^+ neuron manipulation, activation or inhibition of ZI SST^+ neurons had a significantly greater impact on seizure delay or acceleration, especially during the progression phase. Accordingly, we have revised the manuscript in the results section and marked them in red (Page 8, Lines 228-233).

Revised Supplementary Figure 8:

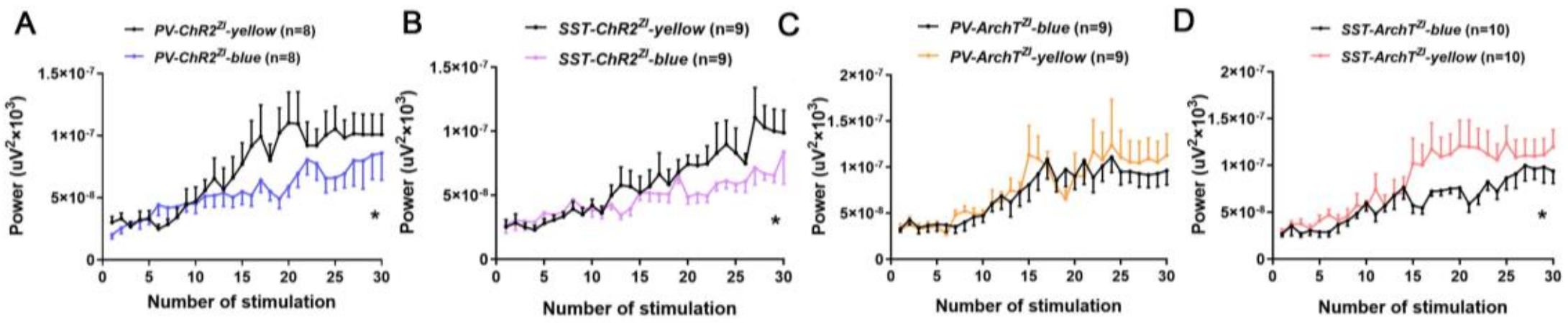

Page 8, Lines 228-233: A direct comparison of the effects of optogenetic activation or inhibition of ZI PV$^+$ and SST$^+$ neurons was performed, with emphasis on their differential efficacy in delaying seizure progression. Our analysis revealed that compared with PV$^+$ neuron manipulation, activation or inhibition of ZI SST$^+$ neurons had a significantly greater impact on seizure delay or acceleration, especially during the progression phase (Supplementary Fig. 8).

9. The manuscript would improve with a more thorough data analysis. (1) Quantify the correlation between kindling EEG and ZI GCaMP6 signals shown in Figure 1; (2) Perform statistical comparisons of the differences in EEG and GCaMP6 signal durations between the PV and SST groups presented in Figure 2; (3) Analyze the impact of optogenetic inhibition of ZI SST/PV neurons on changes in EEG power spectra.

Reply: We appreciate your valuable suggestion and accordingly we have added more detailed data analysis.

(1) We calculated the correlation between the changes in ZI GCaMP6 signal intensity (ΔF/F%) during seizures and ADD, and found a positive correlation (revised Supplementary Figure 1F). We have revised the manuscript in the results section and marked them in red (Page 5, Lines 117-118).

(2) Further analysis of the calcium signal of PV$^+$ and SST$^+$ neurons during hippocampal seizure revealed that SST$^+$ neurons exhibited a longer calcium signal duration during GS compared to PV$^+$ neurons in the revised Supplementary Figure 4A. We have revised the manuscript in the results section and marked them in red (Page 7, Lines 182-186).

(3) We analyzed the influence of optogenetic regulation of PV$^+$ and SST$^+$ neurons on the EEG power spectrum during hippocampal seizures. The results showed that optogenetic activation of both PV$^+$ and SST$^+$ neurons could significantly reduce EEG power spectrum. Inhibiting SST$^+$ neurons (non-PV$^+$ neurons) increases EEG power spectrum in the revised Supplementary Figure 6.

Revised Supplementary Figure 1F:

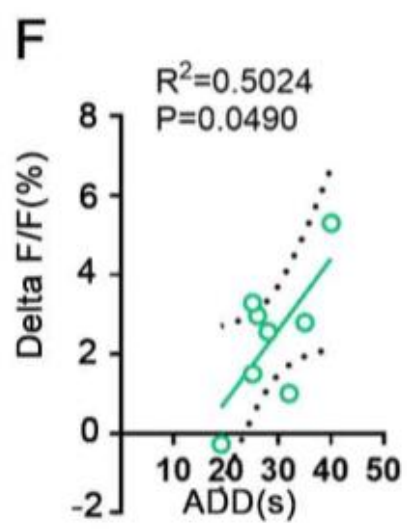

Revised Supplementary Figure 4A:

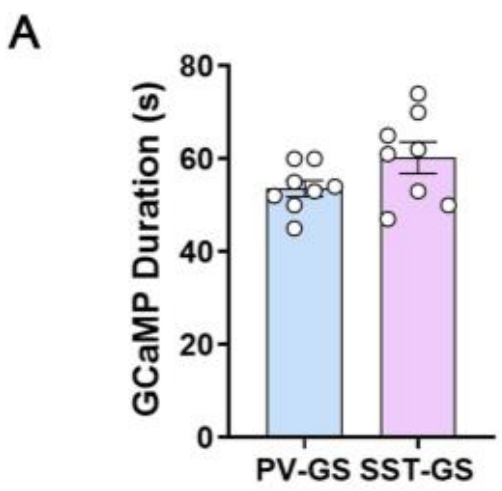

Revised Supplementary Figure 6:

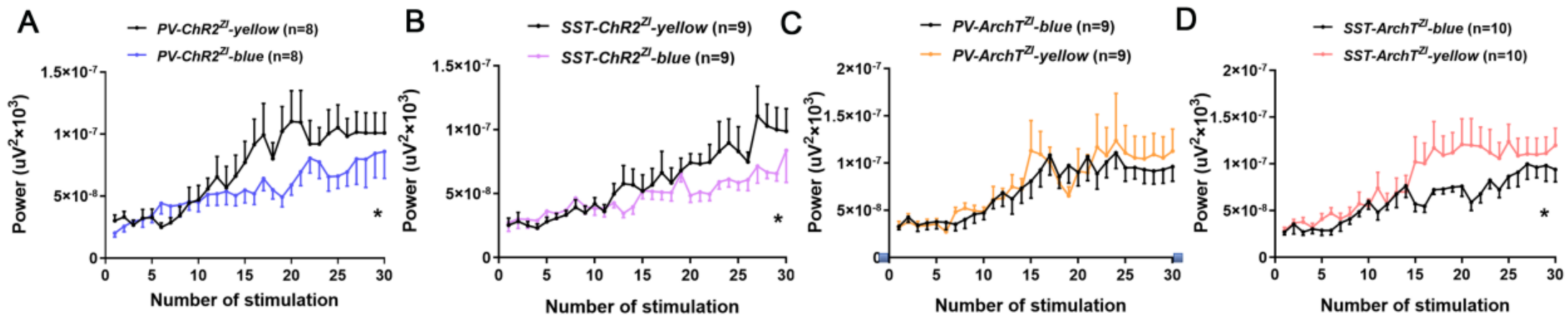

Page 5, Lines 117-118: and the GCaMP6 signal intensity were significantly correlated with the ADD of seizures (Supplementary Fig. 1F),

Page 7, Lines 182-186: Further analysis of the calcium signal duration of PV^+ and SST^+ neurons during hippocampal seizure revealed that SST^+ neurons tended to exhibit a faster increase in calcium signals with a slightly longer duration, as indicated by the steeper slope in SST during GS compared to these of PV^+ neurons (Supplementary Fig. 4A and B).

Page 7, Lines 200-201: and significantly decreased the EEG power spectra (Supplementary Fig. 6A and B).

Page 8, Lines 227-228: and increased the EEG power spectra (Supplementary Fig. 6D)

10. The optogenetic experiments are missing proper control groups. Figures 1 and 2 should incorporate a "seizure-only" group to show the activity patterns of Zi neurons during seizures.

Reply: We appreciate your valuable suggestion. To exclude potential effects of surgery and light delivery, we included a control group with light stimulation, similar as previous studies (Blumberg et al., 2016; Chow et al., 2010). Setting up a "seizure-only" group would indeed be crucial for clarifying the activity patterns of ZI neurons during seizures. Given that our previous analysis showed that no significant difference in the seizure progression between "seizure-only" group and mice receiving control light stimulation, we used the control light -stimulated group as the control group to rigorously match the experimental conditions and exclude potential light-induced effects.

Figure to reviewer:

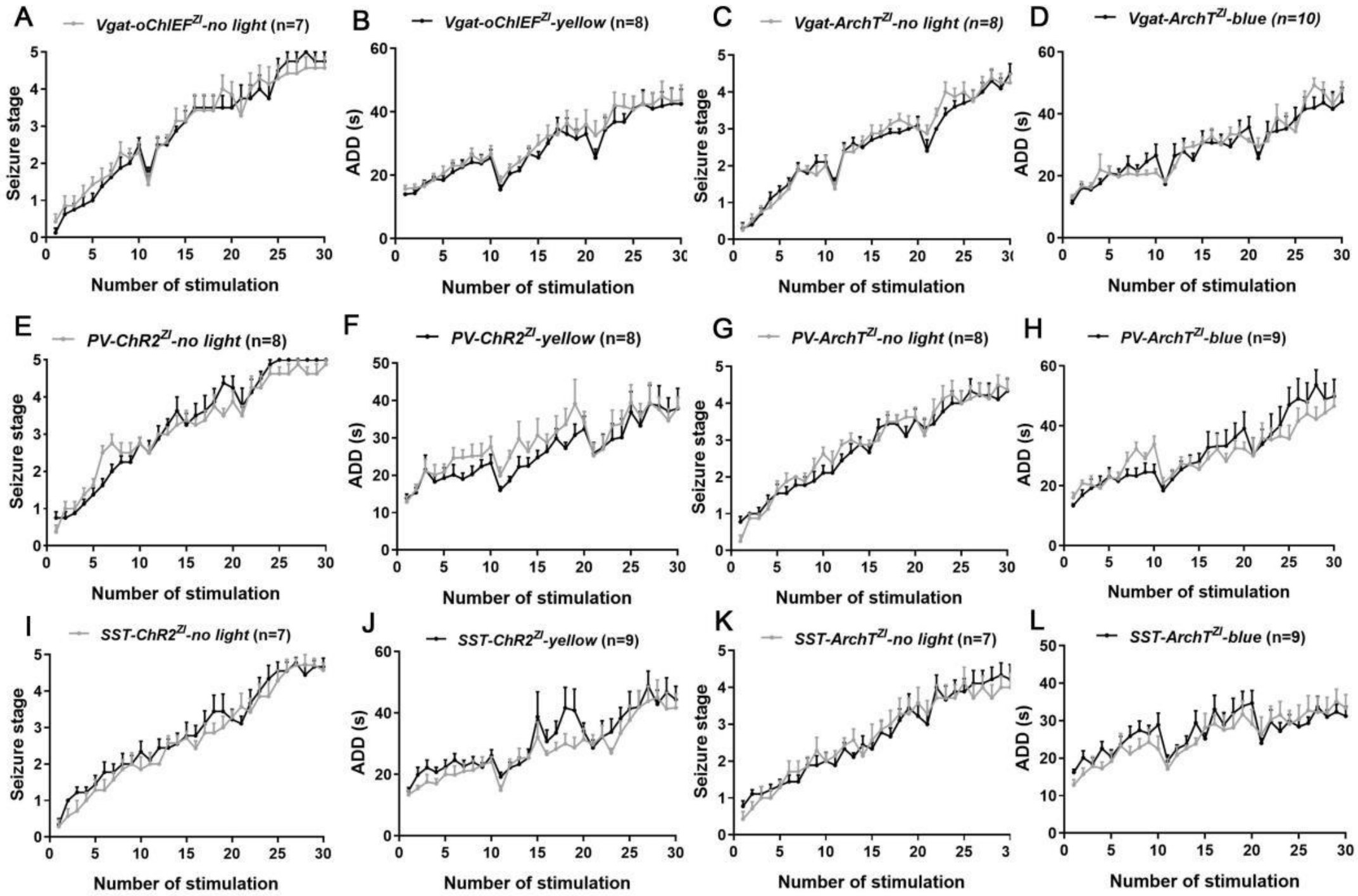

11. Does Figure 4F simultaneously record two sites of ZI SST and downstream brain regions? Please provide detailed explanations in the manuscription or make further annotations in the figures.

Reply: We appreciate your comment. ZI SST^{+} neurons and downstream brain regions were recorded simultaneously. We have revised the manuscript in the results section and marked them in red (Page 10, Lines 265-270).

Revised Figure 4F:

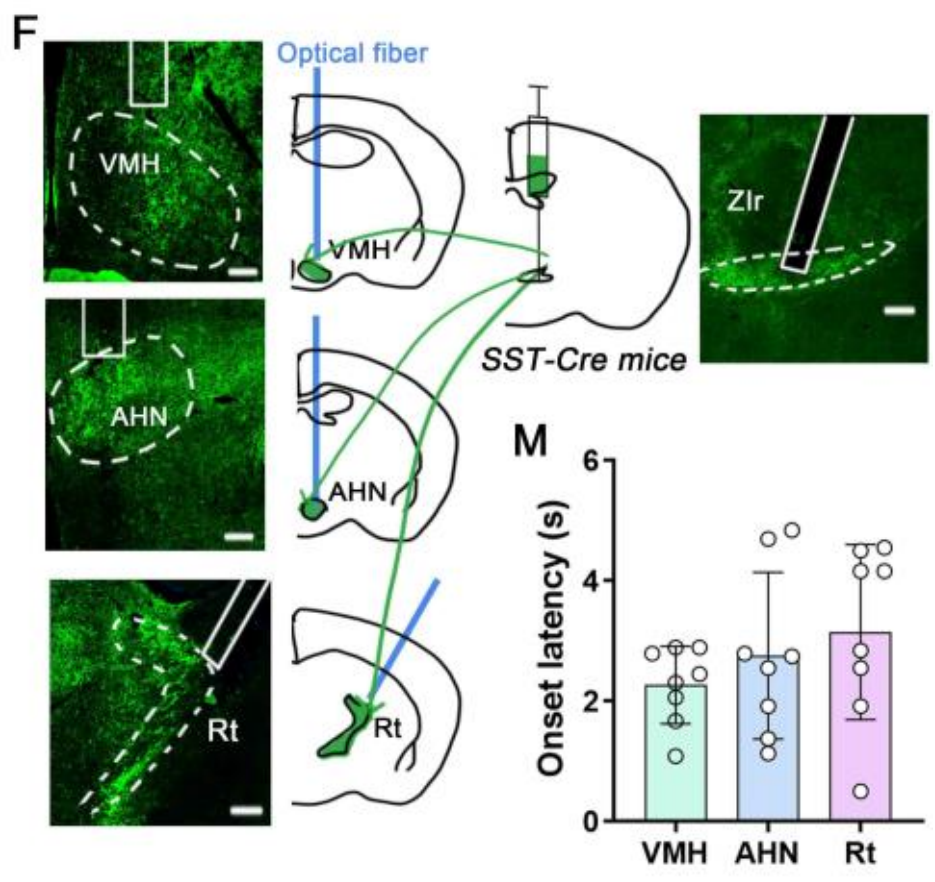

Page 10, Lines 265-270: we delivered axon-targeted GCaMP6s (AAV-EF1α-DIO-axon-GCaMP6s) to SST^{+} neurons in the ZI of *SST-cre* mice, enabling ZI and downstream circuits terminal calcium activity monitoring by implantation of fiber photometry fibers into the VMH, AHN, and Rt during the hippocampal seizures (Fig. 4F). The results showed that the calcium

signal of the above circuits was gradually elevated from FS to GS during seizure progression (Fig. 4G-M),

12. In Figure5, are D-blue and J-blue control groups? There is a significant difference in the number of stimulations in stage 0-2 between D-blue and J-blue. Are there any statistical differences here？Is this result caused by stimulating different circuits？It is suggested that inter-group analysis be conducted on different circuit-specific activation to further demonstrate that the effect of a certain circuit-specific activation is more significant.

Reply: We appreciate your valuable suggestion. The D-blue and J-blue groups respectively represent the control groups of different circuits. After re-tabulating the data, we confirmed that there is no statistically significant difference in the number of stimulations in the stages 0-2 and to GS between the D-blue and J-blue groups. However, the two groups seemed to exhibit different baselines trends, which may be attributable to stimulation of distinct neural circuits.

Figure to reviewer:

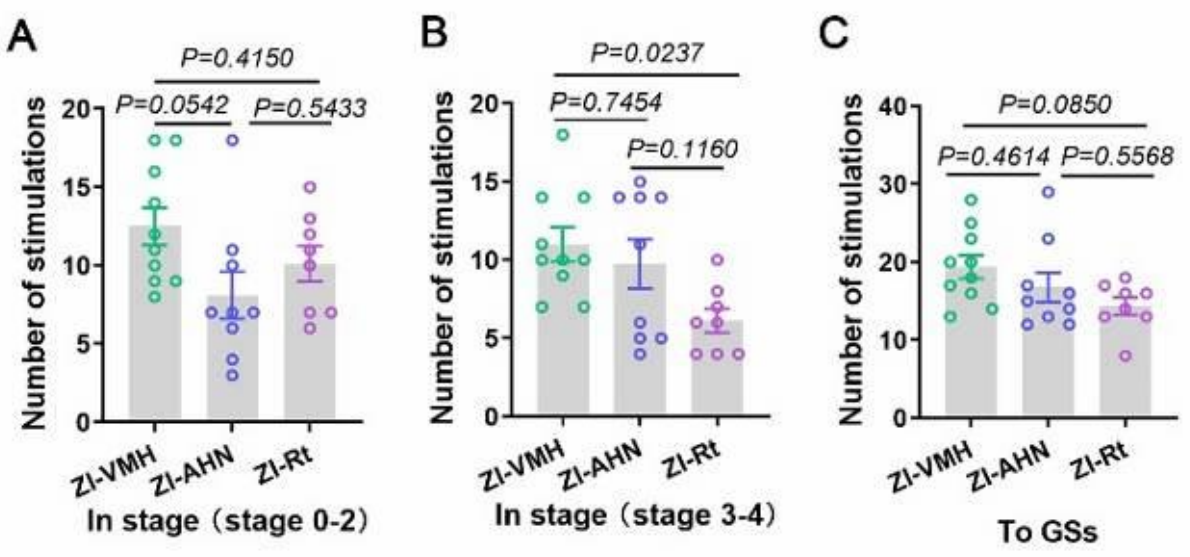

13. The GCaMP6 signals in the VMH, AHN, and Rt occur more quickly than in ZI SST neurons, even though the ZI is upstream (figure 4G-L and figure 1C, D and M, N). This inconsistency needs to be clarified, as it may indicate the presence of alternative pathways or possible technical errors.

Reply: We appreciate your valuable suggestion. For data analysis, we only included mice with correct virus expression and ceramic ferrule positions in the statistics, excluding signal differences caused by technical errors. The delayed signals observed in ZI SST^+ neurons could be explained by two possibilities: (1) local inhibitory feedback microcircuits exist within the ZI, limiting the quick increased Ca^{2+} level. (2) a more rapid direct pathway-such as a hippocampal-VMH/AHN/Rt circuit-may account for the earlier activation in these regions compared to ZI SST^+ neurons (Chang and Gean, 2019). Nevertheless, our key experimental evidence demonstrates a causal role for the direct ZI-VMH circuit in modulating seizure activity. Thus, to bring this point to the attention of our readers, we have added related descriptions in the manuscript in the discussion section and marked them in red (Page 18, Lines 501-508).

Page 18, Lines 501-508: Although the ZI being anatomically upstream, the GCaMP6 signals in the VMH, AHN and Rt occur faster than those in ZI SST^+ neurons. This delayed signal observed in ZI SST^+ neurons could be explained by two possibilities: (1) local inhibitory feedback microcircuits exist within the ZI, limiting the quick increased Ca^{2+} level. (2) a more rapid direct pathway-such as a hippocampal-VMH/AHN/Rt circuit-may account for the earlier activation in

these regions compared to ZI SST^+ neurons (Chang and Gean, 2019). Nevertheless, we demonstrate a causal role for the ZI-VMH circuit in modulating seizure activity.

14. ZI SST neurons are GABAergic and are therefore expected to inhibit VMH neurons when activated. Activating ZI SST neurons reduces seizure progression, implying that these neurons act by suppressing VMH glutamatergic neurons. As a result, the activity of VMH neurons should decrease. However, Figure 6E indicates that activating ZI SST neurons and stimulating VMH glutamatergic neurons lead to opposite outcomes. The authors should clarify whether this inconsistency is due to direct connections or indirect circuit mechanisms.
Reply: We appreciate your valuable suggestion. The apparently opposing effects of activating the ZI^{SST}-VMH circuit versus activating VMH glutamatergic neurons do not represent a logical contradiction; rather, they provide complementary evidence supporting our central conclusion that ZI SST^+ neurons exert an anti-seizure effect by inhibiting VMH glutamatergic neurons. Specifically, our optogenetic experiments demonstrate that activation of the ZI^{SST}-VMH circuit suppresses VMH glutamatergic neuronal activity (revised Figure 6C and D) and significantly delays seizure progression (revised Figure 6F, I and J), establishing a functional inhibitory connection underlying the anti-seizure circuit. Conversely, direct activation of VMH glutamatergic neurons is sufficient to promote seizure activity and can override the protective effect of ZI^{SST}-VMH circuit activation (revised Figure 6F, G and H). Together, these findings provide convergent evidence that inhibition of VMH glutamatergic neurons by ZI SST^+ neurons is a key mechanism mediating seizure suppression. We have revised the manuscript in the discussion section and marked them in red (Page 18, Lines 518-525).

Page 18, Lines 518-525: Specifically, our optogenetic experiments demonstrate that activation of the ZI^{SST}-VMH circuit suppresses VMH glutamatergic neuronal activity and significantly delays seizure progression, establishing a functional inhibitory connection underlying the anti-seizure circuit. Conversely, direct activation of VMH glutamatergic neurons is sufficient to promote seizure activity and can override the protective effect of ZI^{SST}-VMH circuit activation. Together, these findings provide convergent evidence that inhibition of VMH glutamatergic neurons by ZI SST^+ neurons is an important mechanism mediating seizure suppression.

15. The manuscript does not exclude direct hippocampal projections to VMH, AHN, or Rt, which could confound interpretations of the ZI SST→downstream pathway.
Reply: We appreciate your valuable comment. We fully acknowledge that direct hippocampal projections to the VMH, AHN, or Rt could potentially confound interpretations of the ZI SST→downstream pathway. To address this concern, we carefully examined the existing literature and reviewed our own experimental data. This study primarily focuses on the ZI, a region distant from the epileptic focus, and investigates the role and mechanisms of SST neurons within the ZI, revealing that their effects are mediated through the VMH. In addition, VMH and AHN are composed of major glutamatergic neurons that guarantee rapid responses. Anatomical studies confirm the fornix fibers of the hippocampus directly project to the VMH (Swanson, 2000), whole-brain tracing of hippocampal single neurons reveals a direct projection between the hippocampus and the Rt (Qiu et al., 2024). However, these connections are relatively sparse and are thought to serve a modulatory role rather than acting as a primary driver of downstream

activity. Indeed, we found that direct projection might be involved in the regulation of dynamic calcium signals in downstream VMH. Dynamically, the onset latency of calcium signal responses in the VMH was observed to be the shortest among the those recorded in other regions such as AHN or the Rt (Revised Figure 4M). It is suggested that the neural response of VMH has earlier temporal characteristics. To better describe this point, we have revised the manuscript in the discussion section and marked them in red (Page 18, Lines 501-508).

Revised Figure 4F-M:

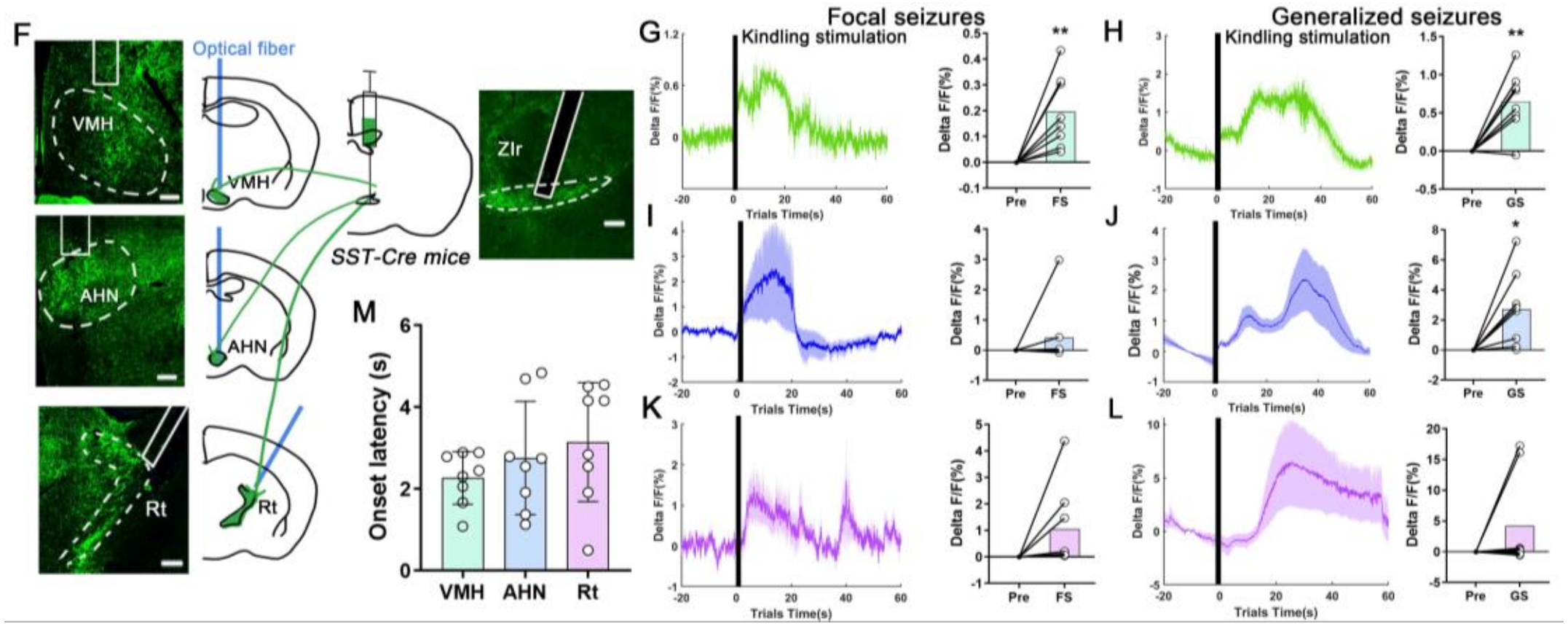

Page 18, Lines 501-508: Although the ZI being anatomically upstream, the GCaMP6 signals in the VMH, AHN and Rt occur faster than those in ZI SST^{+} neurons. This delayed signal observed in ZI SST neurons could be explained by two possibilities: (1) local inhibitory feedback microcircuits exist within the ZI, limiting the quick increased Ca^{2+} level. (2) a more rapid direct pathway-such as a hippocampal-VMH/AHN/Rt circuit-may account for the earlier activation in these regions compared to ZI SST^{+} neurons (Chang and Gean, 2019). Nevertheless, we demonstrate a causal role for the ZI-VMH circuit in modulating seizure activity.

16. Using CaMKII alone to identify glutamatergic neurons is inadequate since GABAergic projection neurons, like medium-sized spiny neurons in the striatum, can also express CaMKII. Thus, it is necessary to use additional markers (such as vGluT1/2) for confirmation.

Reply: We appreciate your valuable suggestion. Indeed, using only CaMKII alone as a marker for glutamatergic neurons is not rigorous enough, especially in brain regions such as the striatum you mentioned. Accordingly, we conducted an immunofluorescence co-labeling staining experiment of CaMKII with VGLUT1 or VGLUT2. The results showed that 95.66% ± 2.201 of $CaMKII^{+}$ neurons simultaneously expressed VGLUT1, and 76.24% ± 2.595 of $CaMKII^{+}$ neurons simultaneously expressed VGLUT2. We have revised the manuscript in the results section and marked them in red (Page 12, Lines 328-334).

Revised Supplementary Figure 13:

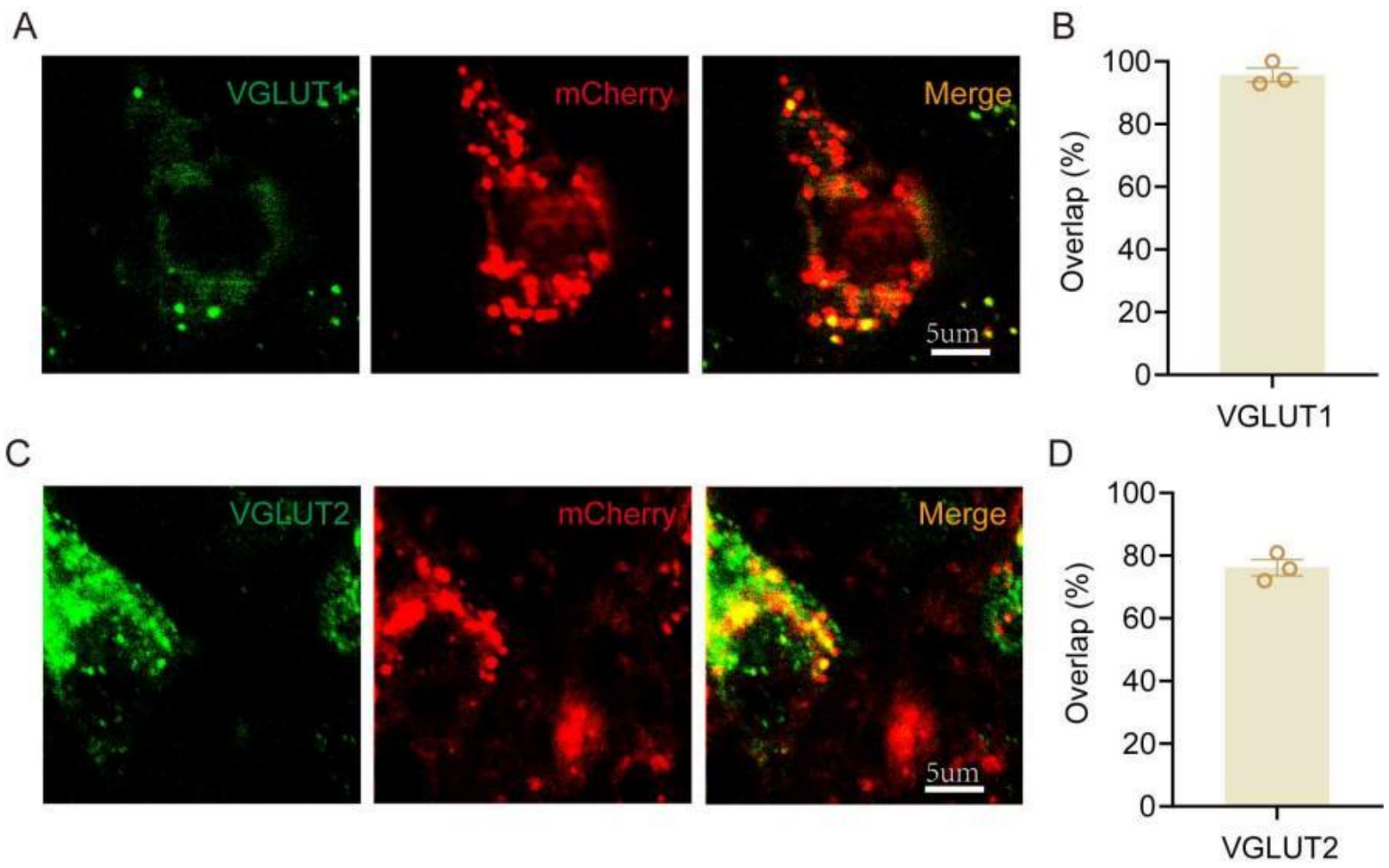

Page 12, Lines 328-334: As the VMH is mainly composed of glutamatergic neurons and mainly expresses VGLUT1 and VGLUT2 (Zhang and Geller, 2010), to verify the specificity of our labeling with viruses driven by the CaMKII promoter used to label glutamatergic neurons in the VMH, we performed co-immunostaining for VGLUT1 and VGLUT2. The results showed that 95.66% ± 2.201 of $CaMKII^+$ neurons simultaneously expressed VGLUT1, and 76.24% ± 2.595 of $CaMKII^+$ neurons simultaneously expressed VGLUT1 (Supplementary Fig. 13).

17. Figure 6D does not demonstrate direct innervation from ZI SST neurons to the VMH. To confirm monosynaptic connections, slice electrophysiology techniques, such as optogenetic IPSC recordings, are required.

Reply: We appreciate your valuable suggestion. Based on your suggestion, we conducted a slice electrophysiology experiment. We injected AAV2/9-DIO-ChR2-EYFP virus into the ZIr of *SST-cre* mice, the AAV2/9-CaMKIIa-mCherry virus into the VMH and prepared brain slices for whole-cell recording. The results showed that optogenetic pulse-laser-evoked IPSC recorded from the clamped VMH glutamatergic neuron persisted after TTX and 4-AP treatment, and was abolished by the $GABA_A$ receptor antagonist bicuculline, confirming monosynaptic GABAergic input from ZI to VMH. We have revised the manuscript in the results section and marked them in red (Page 12, Lines 343-348).

Revised Figure 6E:

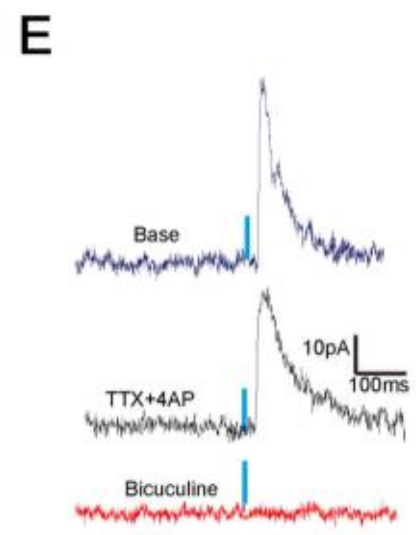

Page 12, Lines 343-348: Further, we injected AAV2/9-DIO-ChR2-EYFP virus into the ZIr, and AAV2/9-CaMKIIa-mCherry virus into the VMH of *SST-cre* mice and prepared brain slices for whole-cell recording. The results showed that optogenetic pulse-laser-evoked IPSC recorded from the VMH glutamatergic neuron, which is persisted after TTX and 4-AP treatment, but was

abolished by the $GABA_A$ receptor antagonist bicuculline, confirming monosynaptic GABAergic connection (Fig. 6E).

18. Are there any other types of neurons in the Zi brain region? Does it play a role in hippocampal seizure？

Reply: We appreciate your comment. ZI is a brain region with high heterogeneity in cellular composition. It is mainly composed of inhibitory neurons, expressing various molecular markers such as PV^+, SST^+, nitric oxide synthase $(nNOs)^+$, etc (Mitrofanis et al., 2004; Lin et al., 1990; Zhu et al., 2025; Li et al., 2021). In addition, there are a small number of glutamatergic neurons (Mitrofanis et al., 2004). Despite this cellular diversity, the specific roles of these particular types of ZI neurons in hippocampal seizure remain largely unknown. Therefore, c-Fos in the ZI was co-stained with PV, SST, nNOS or VGLUT2, respectively after kindling induced generalized seizures. The results showed that c-Fos positive cells co-localized with the PV^+ or SST^+ neuron subsets represent spatial variation: the seizure activated PV^+ c-Fos were more distributed in the ZIv (45.8%) than ZIr (11.6%), however, the seizure activated SST^+ c-Fos were more distributed in the ZIr (56.6%) than ZIv (30.4%) (Revised Supplementary Fig. 3A-F). Hippocampal seizure activated 15.2% of nNOS neurons in the ZIr during seizure without affecting VGLUT2 neurons (Revised Supplementary Fig. 3G-L). These results indicates that SST^+ neurons were activated across ZIr and ZIv suggesting SST^+ neurons may play a more crucial role in hippocampal seizure among these subtypes. We have revised the manuscript in the results section and marked them in red (Page 6, Lines 164-174).

Revised Supplementary Figure 3:

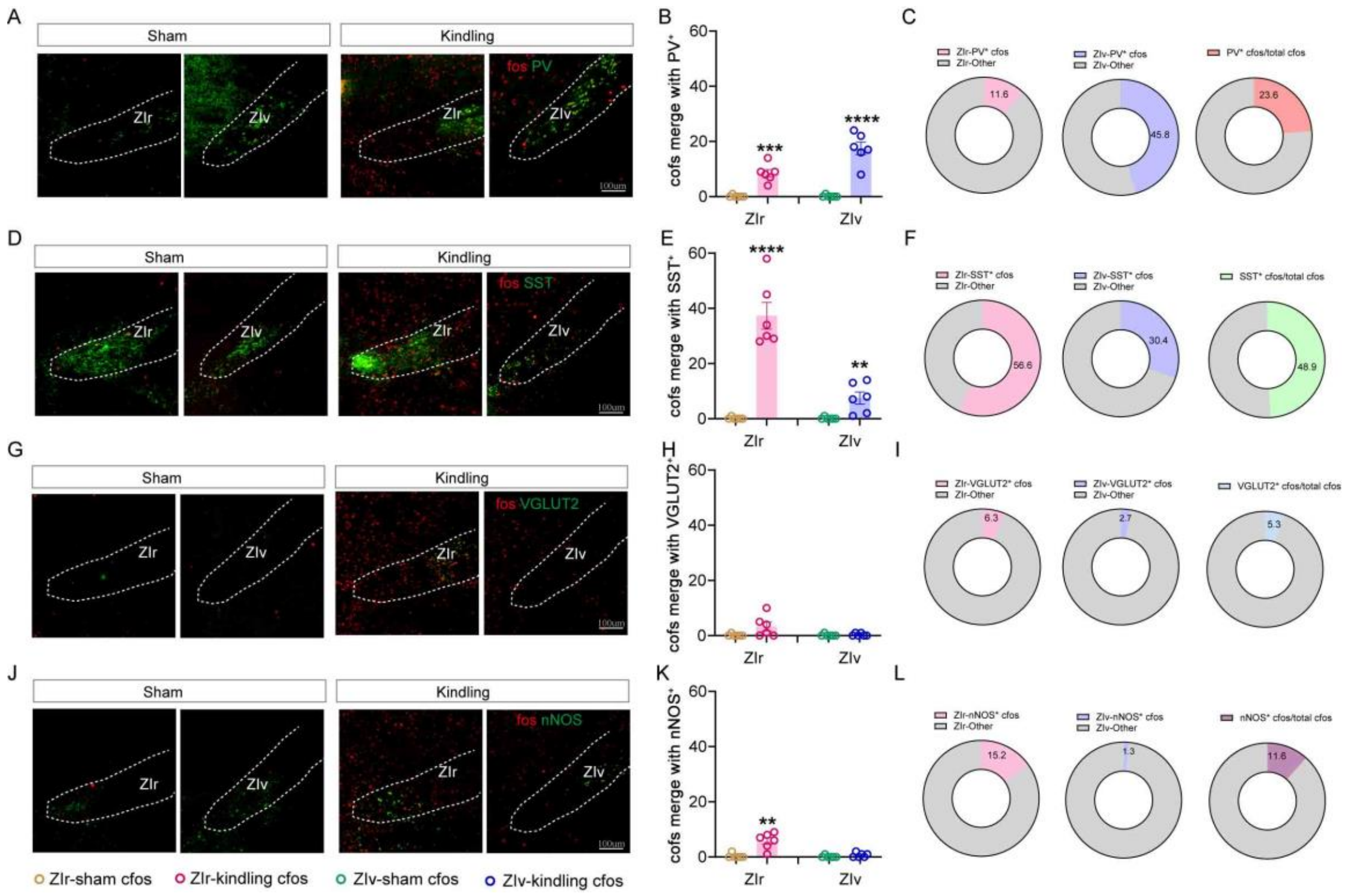

Page 6, Lines 164-174: Firstly, we performed double immunofluorescence staining for PV or SST in neurons activated after seizures in the ZI. The quantitative analysis revealed that overall, 23.6% of the seizure activated neurons were identified as PV^+, while 48.9% were SST^+ (Supplementary Fig. 3A-F), indicating SST^+ GABAergic neurons are much more easily recruited in seizure. Interestingly, c-Fos^+ cells co-localized with the PV^+ or SST^+ neuron subsets represent spatial variation: the c-Fos^+ PV neurons were more distributed in the ZIv (45.8%) than ZIr (11.6%), however, the c-Fos^+ SST neurons were more distributed in the ZIr (56.6%) than ZIv (30.4%) (Supplementary Fig. 3A-F). Hippocampal seizure also activated 15.2% of nNOS neurons in the ZIr during seizure without affecting VGLUT2 neurons (Supplementary Fig. 3G-L).

Minor points:

1. The scale bar value is missing in Fig 1A; high-magnification images for c-Fos staining should be provided.

Reply: We appreciate your comments. We have added clear scale bar values in revised Supplementary Figure 1A and explained them in the revised Figure legends. In addition, we have added representative high-magnification images for c-Fos staining images in the revised Supplementary Figure 1A.

Revised Supplementary Figure 1A:

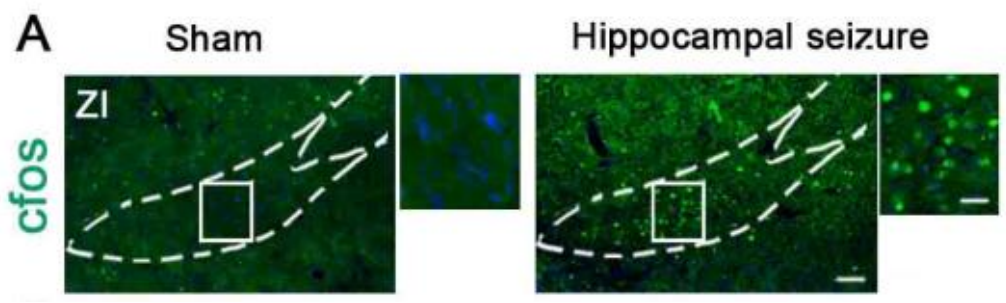

2. Clarify how hippocampal kindling stimulation was delivered to the ipsilateral ZI (manual vs. automated?). And only 10ms stimulation?

Reply: We appreciate this comment. Hippocampal kindling stimulation (electrical stimulation) was delivered via an electrode implanted in the CA3. The stimulation parameters (monophasic square-wave pulses, 400 μA, 20 Hz, 1 ms/pulse, 40 pulses) were pre-set on a constant-current stimulator prior to the experiment. This is a standard parameter in the literature for establishing and validating the kindling model (Etemadi et al., 2015, Chen et al., 2020; Fei et al., 2022).

For optogenetic activation experiments, 473 nm blue light (20 Hz, 10 ms/pulse, 5 mW, in real time) was used. For optogenetic inactivation experiments, 589 nm yellow light (continuous, 5 mW, in real time) was used. This is a commonly used parameter in optogenetics manipulation (Zhao et al., 2022; Grimm et al., 2024; Furman et al., 2024).

3. Provide high-magnification co-staining images to confirm GCaMP6 expression in PV/SST neurons (For example, Figs 1B, G, N; 2B, F, L, P; 3B, H).

Reply: We appreciate your valuable suggestion. To confirm the expression of GCaMP6, ChR2, ArchT, and oChIEF in GABAergic neurons, PV neurons and SST neurons, we obtained high-magnification co-staining images using confocal microscopy. The corresponding modifications are shown in revised Figure 1B, G, N; Figure 2B, F, L, P; Figure 3B, H.

Revised Figure 1B, G, N:

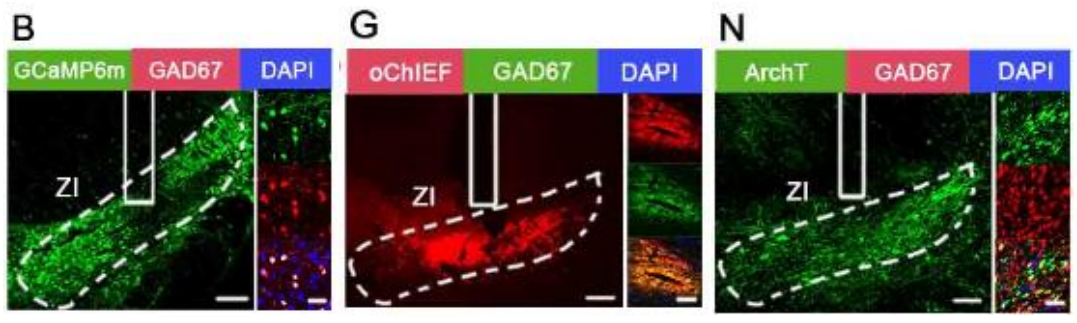

Revised Figure 2B, F, L, P:

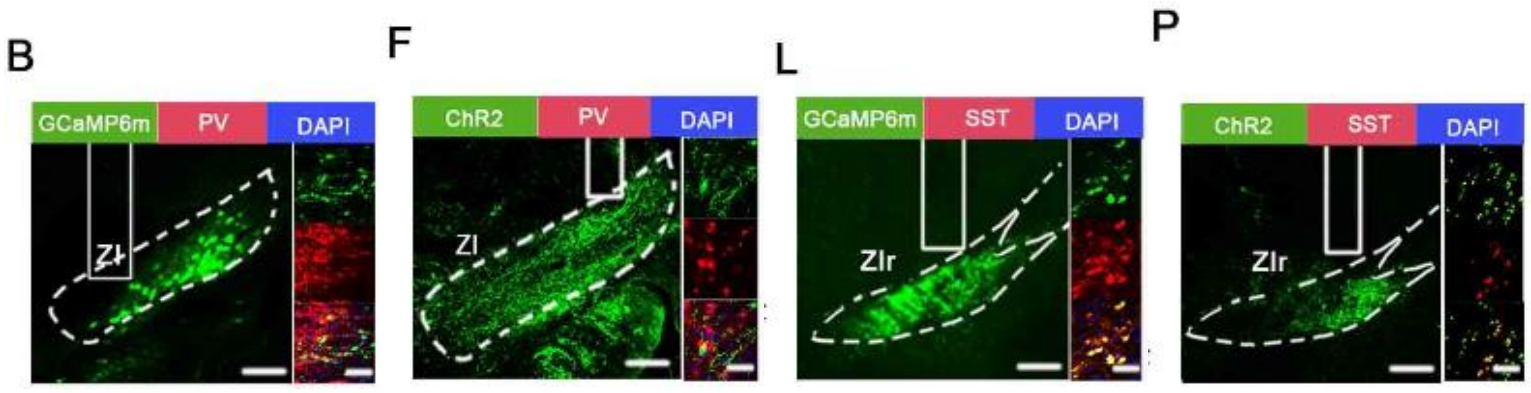

Revised Figure 3B, I:

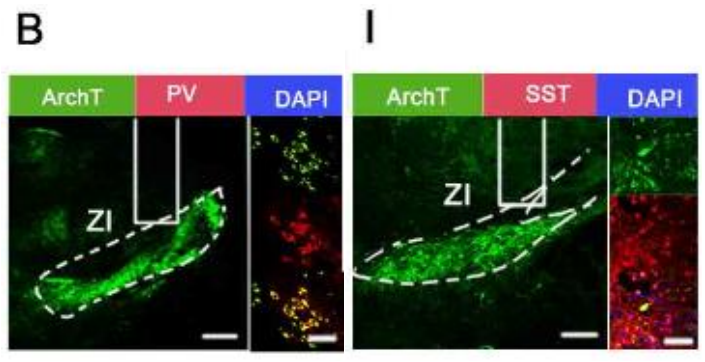

4. Include c-Fos staining in the hippocampus as a positive control for seizure activity.

Reply: We appreciate your valuable suggestion. We have detected c-Fos immunofluorescence and revealed a marked increase in the number of c-Fos-positive neurons within the hippocampus of mice subjected to kindling-induced seizures compared to control group. Accordingly, we have revised the manuscript in the results section and marked them in red (Page 4, Lines 102-104).

Revised Supplementary Figure 1:

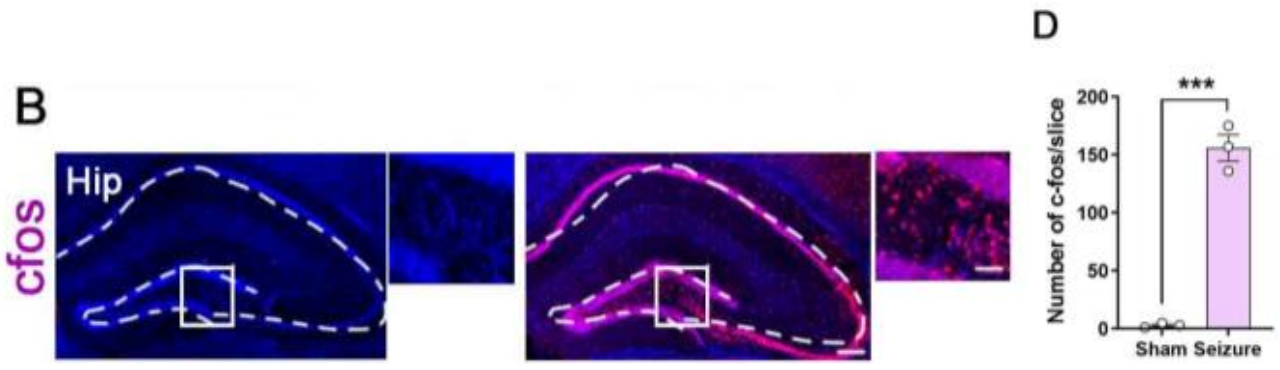

Page 4, Lines 102-104: Correspondingly, the hippocampus, used as a positive control, also exhibited a significant increase in c-Fos expression following seizures (Supplementary Fig. 1B and D).

5. Does inhibiting ZI PV or SST neurons induce seizures in naïve mice? This would test their intrinsic role in seizure threshold.

Reply: We appreciate your comment. We have conducted additional experiments in naïve *PV-cre* or *SST-cre* mice to directly verify the intrinsic role of ZI PV or SST neurons in the basal seizure threshold. Optical fibers were implanted into the ZI of naïve *PV-cre* or *SST-cre* mice to specifically inhibited PV or SST neurons via yellow light stimulation. The results showed that inhibition of ZI PV or SST neurons did not directly induce seizures in naïve mice, suggesting potential auxiliary role in modulating seizure spread.

Figure to reviewer:

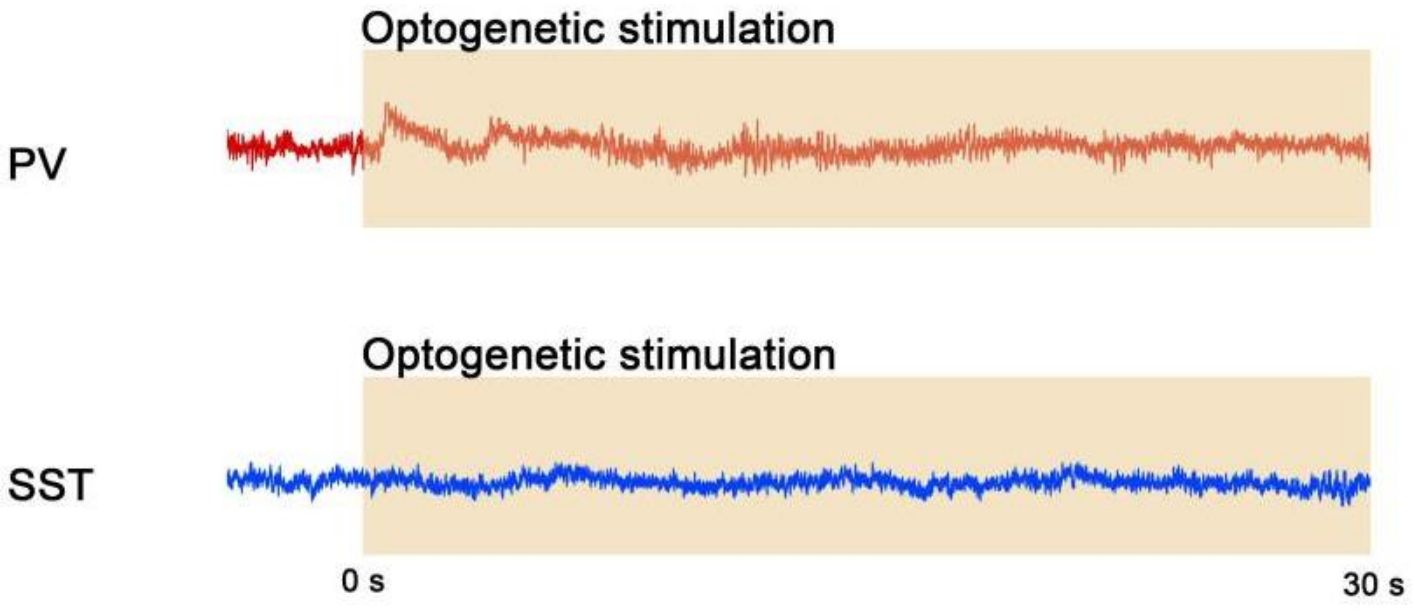

6. Show ZI injection/verification images to confirm the accuracy of stimulation sites.

Reply: We appreciate your comment. All the representative immunohistochemical figures of the experiments have been presented in the main manuscript. All figures include images showing viral expression and optic fiber sites in the stimulation sites which have been provided in the supplementary figures. Importantly, our analyses included only subjects with viral expression and histologically confirmed correct targeting within the ZI, ensuring the accuracy and reliability of the stimulation sites and the corresponding results.

Revised Supplementary Figure 2:

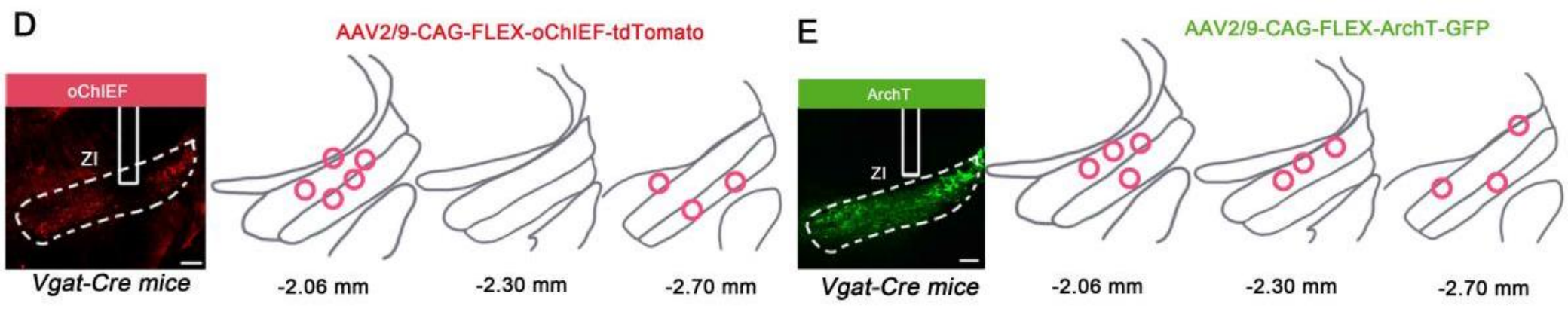

Revised Supplementary Figure 5:

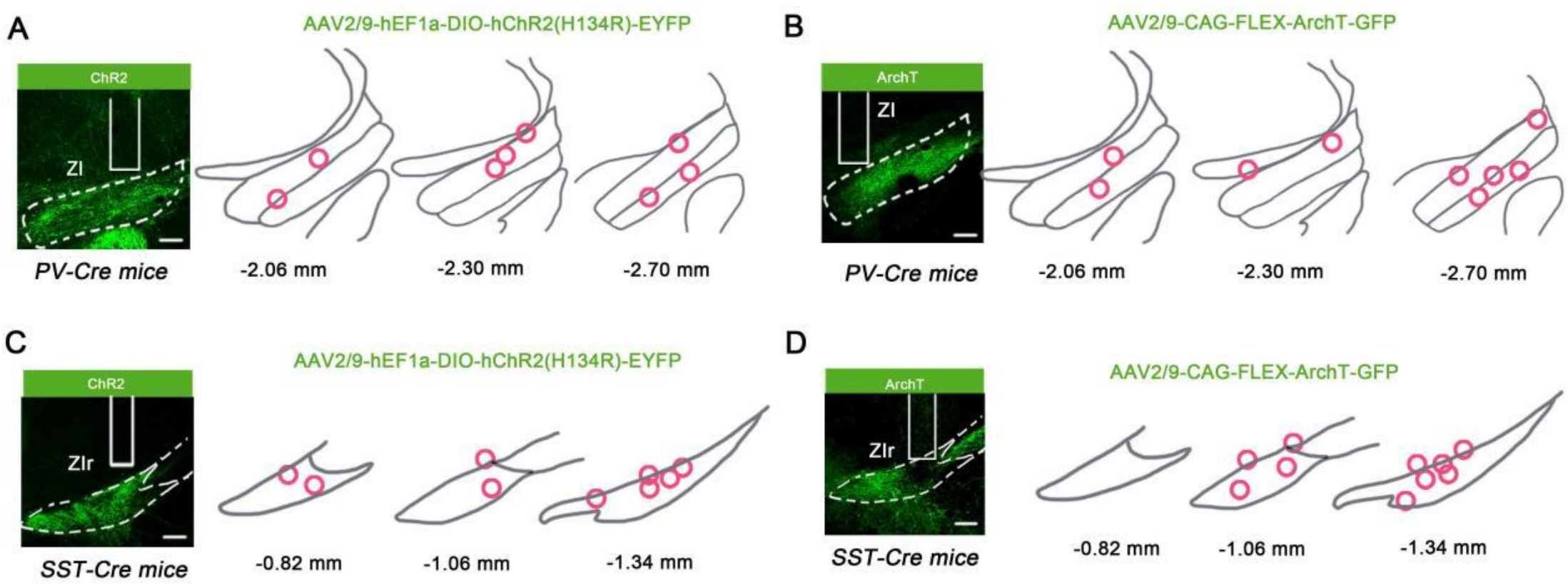

Revised Supplementary Figure 9:

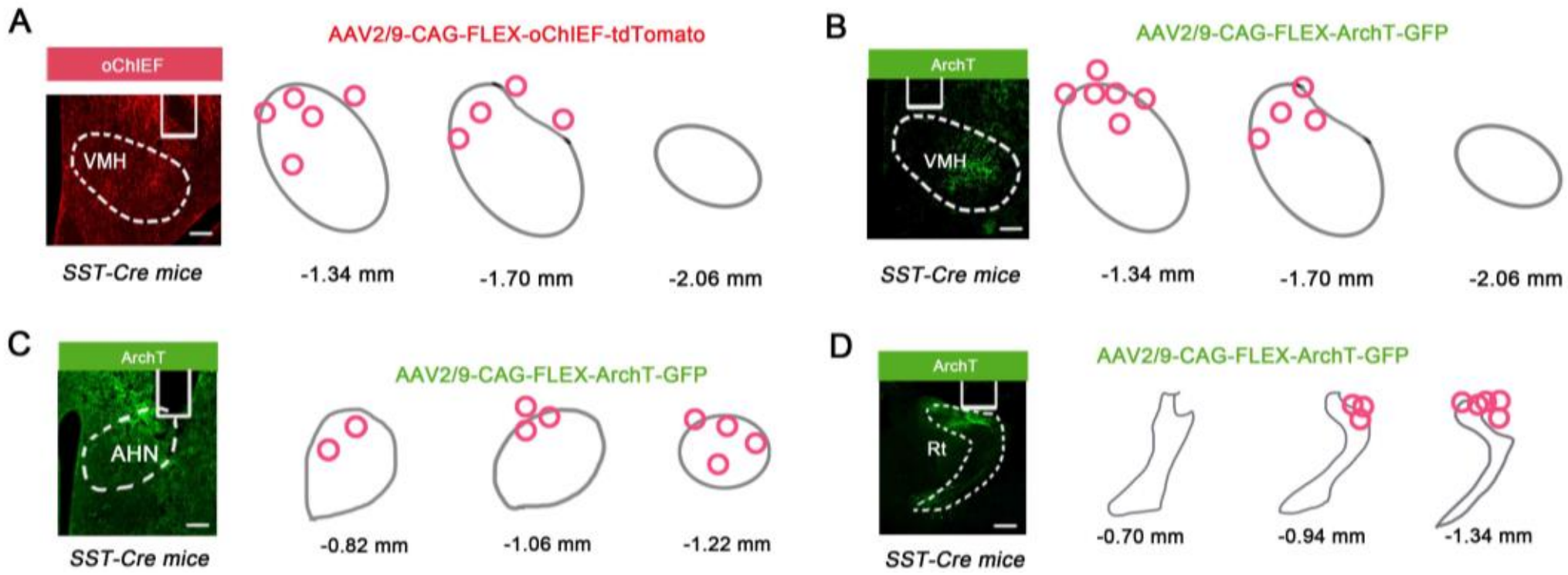

7. Improve resolution and clarity for all immunohistochemistry images.

Reply: We appreciate your valuable suggestion. We have replaced all the immunohistochemistry images in the original manuscript with the optimized high-quality immunohistochemistry images. Meanwhile, we ensure that all the immunohistochemistry images have clear scales.

8. In Fig 2A-B and K-L, the recording sites of GCaMP here are inconsistent between PV-Cre and SST-Cre；in E-F and O-P，here, the expression level of ChR2 varies significantly, which will lead to differences in the results？

Reply: We appreciate your valuable comment. Consistent with established literature documenting SST expression in the rostral ZI while PV expression in the ventral ZI (Mitrofanis et al., 2004), we strategically performed viral injection and fiber photometry at slightly different anatomical sections of the ZI to ensure maximal specificity in manipulating each neuronal subtype. Notably, both the activation profiles from c-Fos experiments (Revised Figure 4E) and the outcomes of downstream neuronal mapping confirm the heterogeneity of SST and PV neurons (Revised Supplementary Figure 16), implying distinct functional roles. This was directly substantiated by our optogenetic studies, which demonstrated that the functions of PV and SST neurons are not identical.

To enhance the robustness of our findings, we have supplemented the sample size for fiber photometry recording and replaced the recording sites with regions where GCaMP expression was enriched within the ZI. The inconsistent expression levels of ChR2 in the ZI are due to the inherent anatomical differences in the distribution of PV^+ and SST^+ neurons within the ZI PV^+ neurons are usually more concentratedly distributed on the ZIv, while SST^+ neurons are more frequently distributed on the ZIr (Revised Supplementary Figure 3). Therefore, due to the differences in anatomical locations, the expression levels are indeed inconsistent. However, the areas illuminated by optical fibers are all regions where the neurons are densely expressed at the core. Accordingly, we have revised the manuscript in the discussion section and marked them in red (Page 16, Lines 443-459).

Revised Supplementary Figure 16:

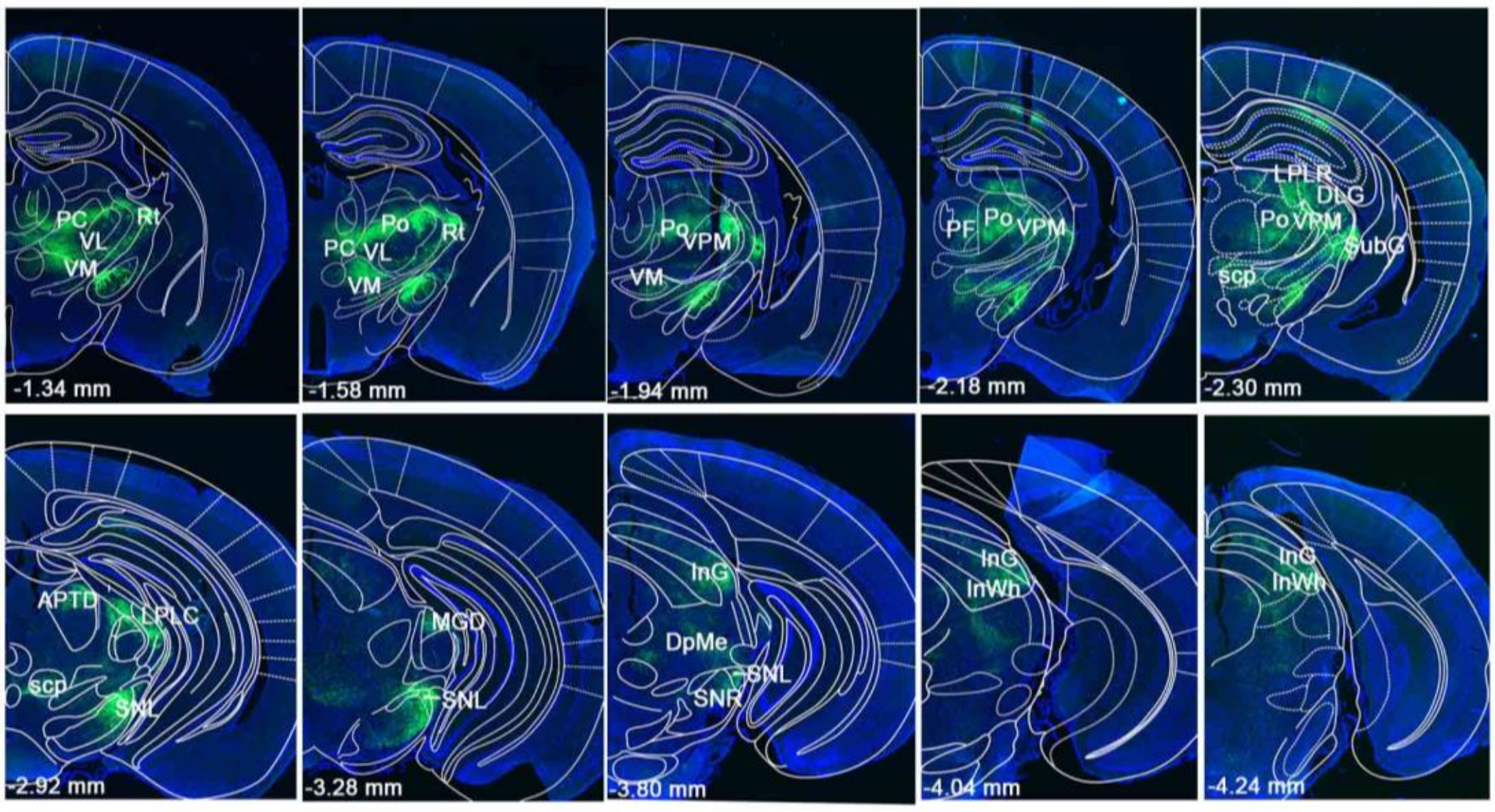

Revised Supplementary Figure 3:

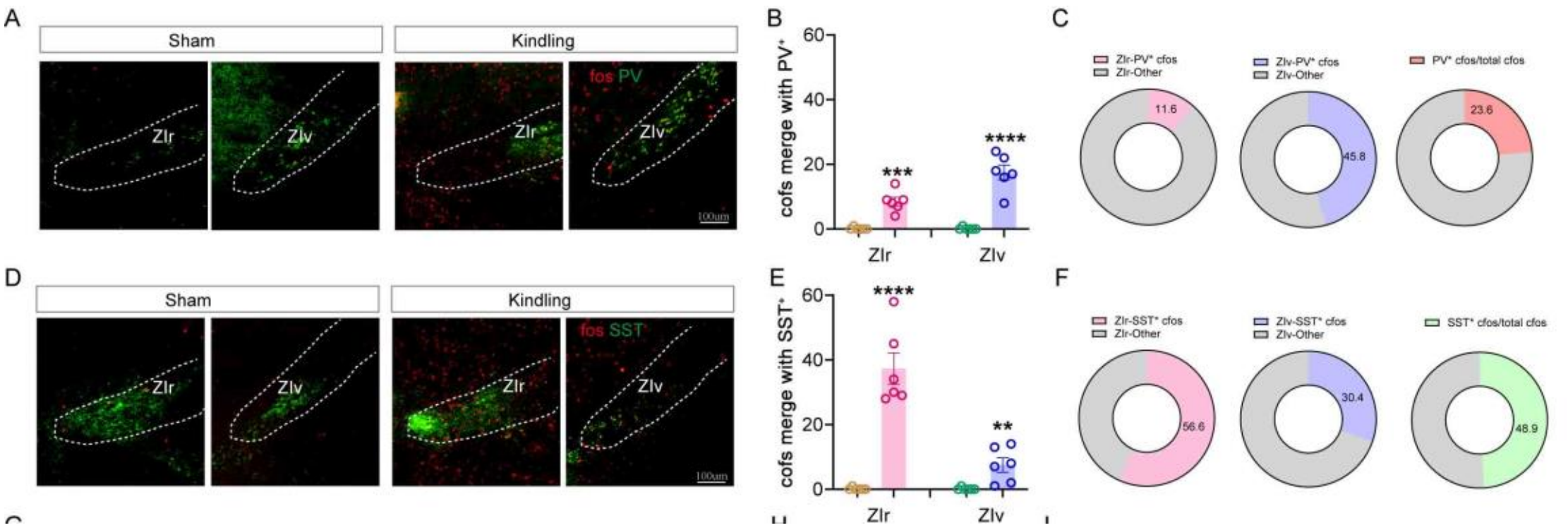

Page 16, Lines 443-459: A primary factor underlying this functional divergence is likely the differential efferent connectivity of ZI SST^{+} and PV^{+} GABAergic neurons. Using anterograde tracing, we found that ZI SST^{+} neurons project predominantly to anterior brain regions, such as the VMH, hippocampus, anteroventral thalamic nucleus, etc., which are involved in integrating cognitive, emotional and homeostatic centers (Wang et al., 2022; Eichenbaum, 2004; Hintiryan et al., 2025). Whereas ZI PV^{+} neurons primarily innervate posterior areas, such as the reticular nucleus of the thalamus and the reticular structure of the brainstem (Supplementary Figure 16), which are implicated in sensory, motor and arousal centers. This fundamental difference in efferent connectivity provides an anatomical substrate for their distinct functional roles. Although the exact circuit mechanism of ZI PV^{+} neuron in seizure is not clear, our study is the first to reveal the pivotal role of the VMH mediating anti-seizure effect of ZI SST^{+} neuron. In addition, the c-Fos stanning results also indicated that SST^{+} GABAergic neurons are much more easily recruited in seizure. Together, these data provide evidences to explains the different role of PV^{+} and SST^{+} neurons in hippocampal seizure, SST^{+} GABAergic neurons predominantly mediate the primary GABAergic control of seizures within the ZI.

9. In Fig 4E, please provide high-magnification images.

Reply: We appreciate your valuable suggestion. We have added representative high-magnification images for c-Fos staining images in the revised Figure 4E.

Revised Figure 4E:

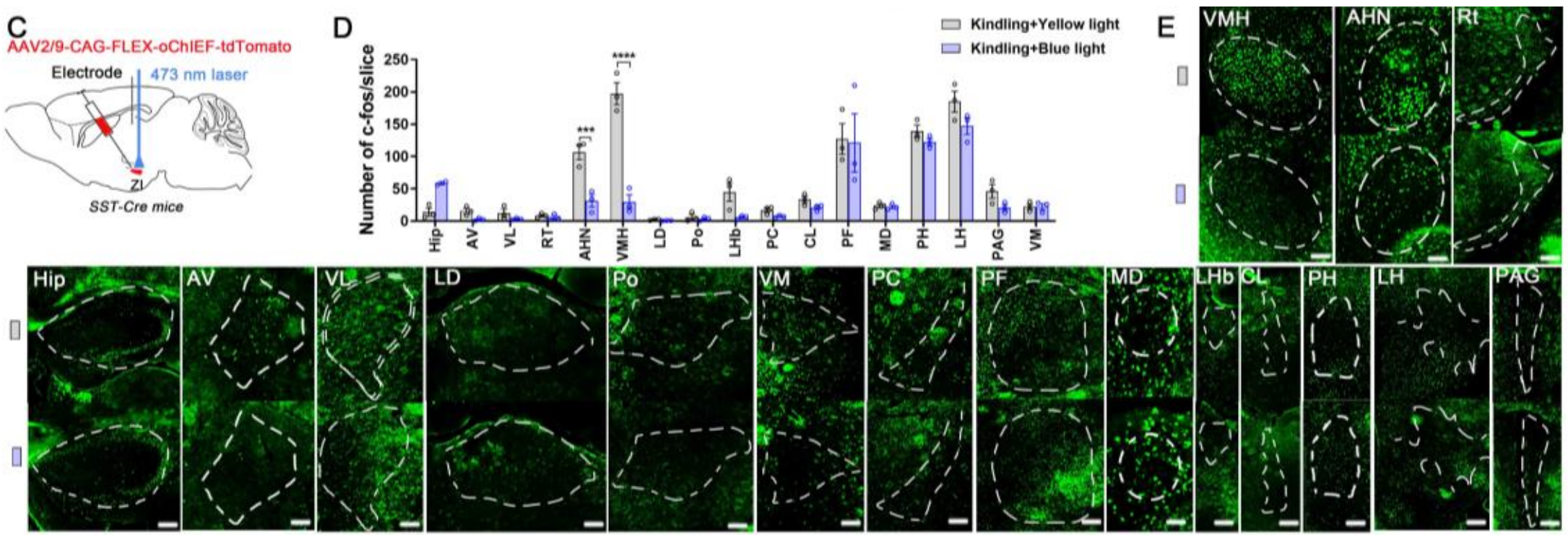

Reviewer #4

This manuscript by Zhang et al. investigates the role of zona incerta (ZI) GABAergic neurons in a hippocampal kindling model of epilepsy. Using calcium fiber photometry, optogenetics, chemogenetics, and viral tracing, the authors show that ZI GABAergic neurons are activated during seizure progression, and that activation of ZI SST+ neurons — but not PV+ neurons — is both necessary and sufficient to suppress seizure spread. Mechanistically, they demonstrate that SST+ projections to the ventromedial hypothalamus (VMH) inhibit glutamatergic neurons, thereby reducing seizure progression. This study addresses an underexplored circuit node in epilepsy, providing valuable mechanistic insights and potential therapeutic targets. However, there are several major issues regarding the lack of proper controls, statistical rigor of analysis, and interpretation of data that need to be addressed.

Reply: We sincerely appreciate the time and effort you have taken to review our manuscript and for the positive feedback. Your comments are highly valuable and have significantly contributed to the revision and improvement of our paper. In this letter, we address each of your comments individually, followed by our corresponding responses. For clarity, we have restated your comments in bold font and provided our responses in blue.

Major Points

1. PV+ vs SST+ contribution

Optogenetic activation of both PV+ and SST+ neurons reduces seizure progression, yet only SST+ inhibition/ablation has an effect. The manuscript interprets this as PV+ neurons being dispensable. However, this asymmetry requires deeper discussion:

• Could PV+ projections to other targets explain why inhibition shows no effect?

• Is optogenetic inhibition/ablation of PV+ neurons complete and efficient?

• Alternative interpretations should be addressed explicitly.

Reply: We appreciate your valuable comments.

(1) Our experiments found that optogenetic activation of both PV^+ and SST^+ neurons attenuate seizure progression, yet only SST^+ neurons inhibition/ablation are effective. Regarding the suggestion that PV^+ projections to other targets may explain difference in the effect of optogenetic inhibition of SST and PV neurons, we performed whole-brain mapping of ZI PV^+ neuronal projections. Unlike SST^+ neurons, PV^+ neurons send dense axonal projections predominantly to posterior regions, including specific thalamic nuclei and midbrain areas (Supplementary Figure 16). In addition, complementary c-Fos analysis revealed that seizure-activated PV^+ neurons are more concentrated in the ZIv (45.8%) than ZIr (11.6%), whereas SST^+ neurons show the opposite pattern, with greater activation in the ZIr (56.6%) than ZIv (30.4%) (Supplementary Figure 3A-F). These anatomical and functional data may explain the distinct contributions of PV^+ and SST^+ neurons during hippocampal seizures.

(2) Multiple measures were taken to ensure experimental rigor and reliability. Optogenetic inhibition of PV^+ neurons employed the same viral constructs and parameters successfully used for SST^+ neurons, with all animals histologically verified for correct targeting. Additionally, we performed parallel experiments using AAV2/9-CAG-DIO-taCaspase3-TEVp to specifically ablate PV^+ neurons, confirming high efficiency comparable to SST^+ neuron ablation. Together, these convergent approaches demonstrate that, PV^+ neurons are structurally and functionally different

from SST neurons for modulating seizure progression.

Accordingly, we have revised the manuscript in the discussion section and marked them in red (Page 16, Lines 443-459).

Revised Supplementary Figure 16:

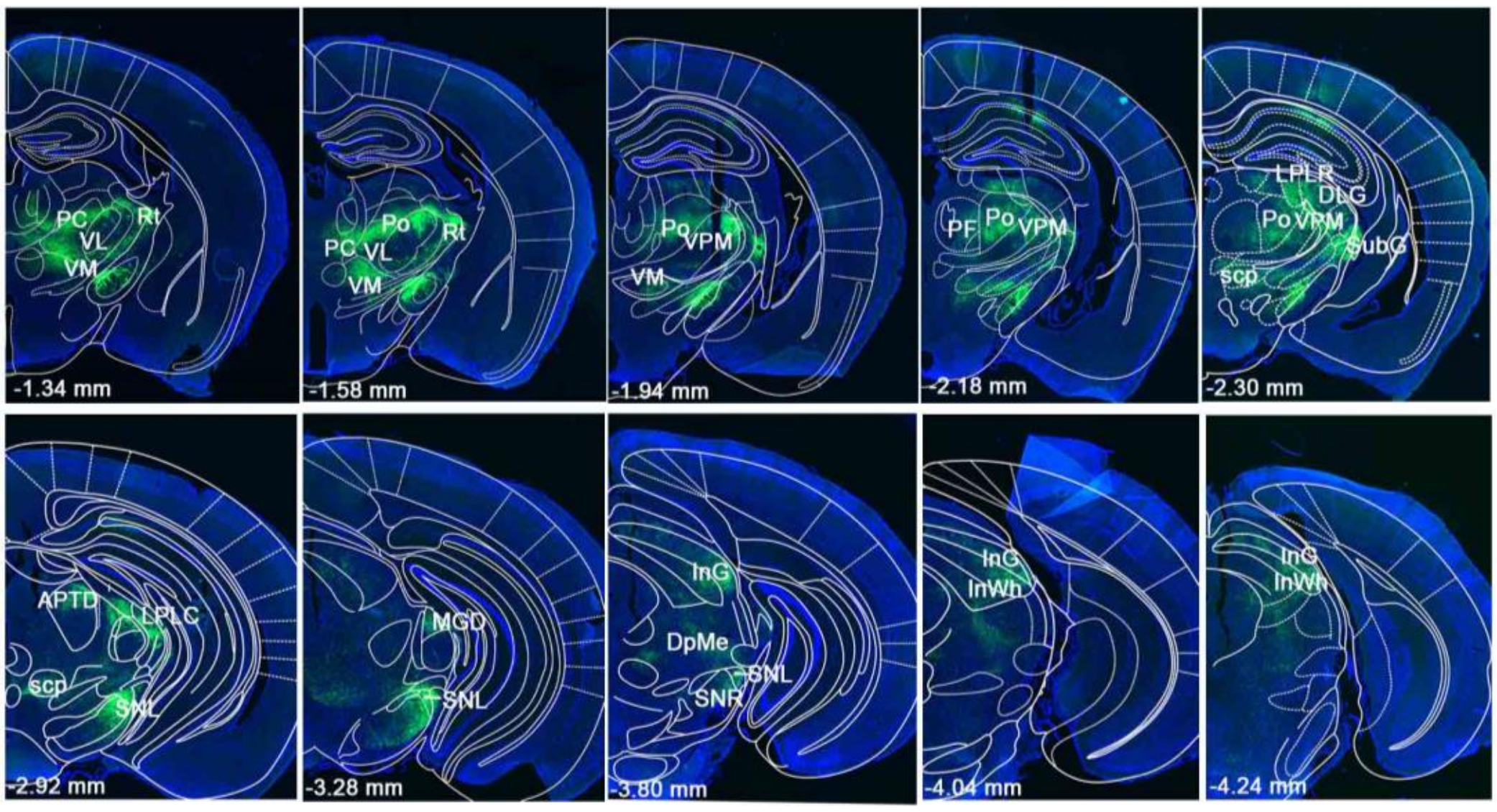

Revised Supplementary Figure 3:

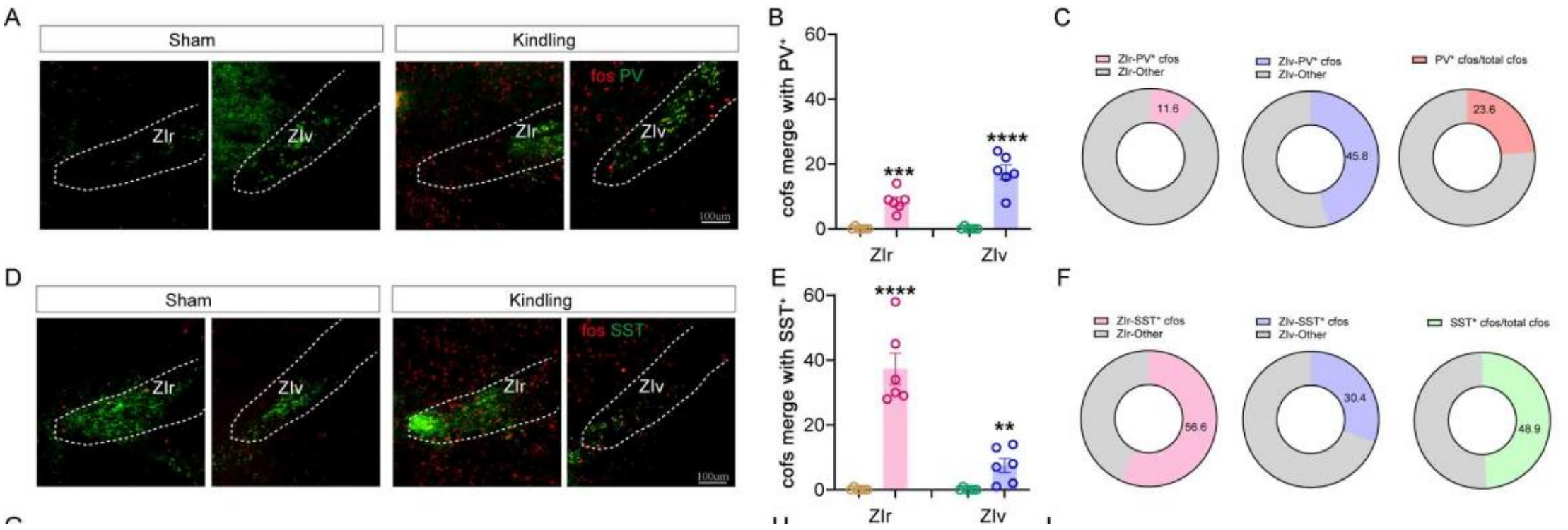

Page 16, Lines 443-459: A primary factor underlying this functional divergence is likely the differential efferent connectivity of ZI SST$^+$ and PV$^+$ GABAergic neurons. Using anterograde tracing, we found that ZI SST$^+$ neurons project predominantly to anterior brain regions, such as the VMH, hippocampus, anteroventral thalamic nucleus, etc., which are involved in integrating cognitive, emotional and homeostatic centers (Wang et al., 2022; Eichenbaum, 2004; Hintiryan et al., 2025). Whereas ZI PV$^+$ neurons primarily innervate posterior areas, such as the reticular nucleus of the thalamus and the reticular structure of the brainstem (Supplementary Fig. 16), which are implicated in sensory, motor and arousal centers. This fundamental difference in efferent connectivity provides an anatomical substrate for their distinct functional roles. Although the exact circuit mechanism of ZI PV$^+$ neuron in seizure is not clear, our study is the first to reveal the pivotal role of the VMH mediating anti-seizure effect of ZI SST$^+$ neuron. In addition, the c-Fos stanning results also indicated that SST$^+$ GABAergic neurons are much more easily recruited in seizure. Together, these data provide evidences to explains the different role of PV$^+$ and SST$^+$

neurons in hippocampal seizure, SST^+ GABAergic neurons predominantly mediate the primary GABAergic control of seizures within the ZI.

2. Statistical rigor

The Methods state that no sample size calculation or randomization was performed. For a Nature Communications paper, this is a significant weakness. The authors should:

Clarify whether n represents animals or cells in every figure.

Provide justification for chosen sample sizes.

Clarify whether data were pooled across multiple cells per animal and how independence was handled (nested statistics, mixed models).

Reply: We appreciate your valuable suggestion. We have revised the manuscript in the figure legend section and marked statistical parameter in detail in red.

Sample sizes were chosen based on established standards in the field and on our prior experience with similar experiments, and they are consistent with those used in related published studies (Duan et al., 2025; Ribierre et al., 2024; Krook-Magnuson et al., 2013). All analyses adhered strictly to the principle of biological replication. For behavioral assays from hippocampal kindling-induced seizure (e.g., seizure stage, ADD, number of stimulations to GS or in stage, and EEG power), each data point represents a single, independent animal. Therefore, the sample size (n) for these experiments refers to the number of mice. For immunohistochemical quantification, three tissue sections per animal were quantified and averaged to generate a single value, which was used for inter-group comparisons. We have added these details for sample size and statistics in the method section and marked them in red.

For several datasets, especially the photometry datasets, it seems that the sample sizes were especially low.

Reply: We appreciate your valuable suggestion. We have increased the sample sizes and analysis in the revised optical fiber recording experiment to enhance the reliability and stability of our photometric datasets.

Revised Figure 1C and D:

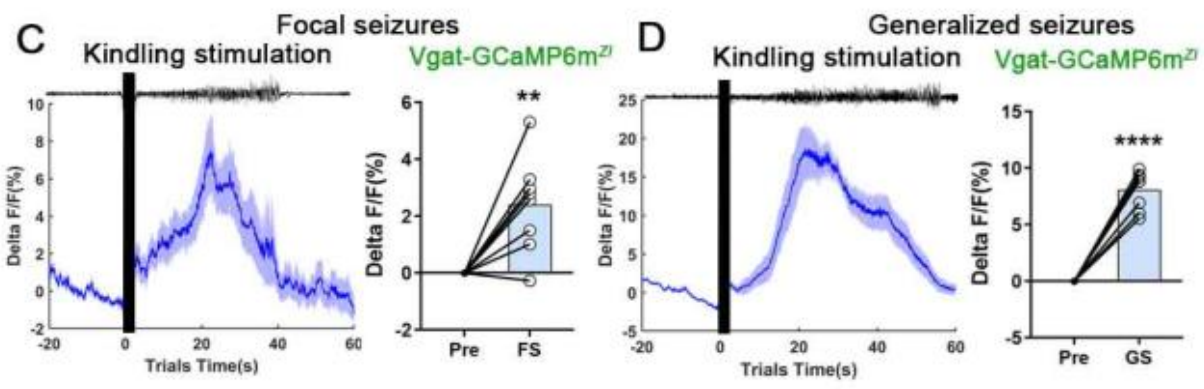

Revised Figure 2C, D, M and N:

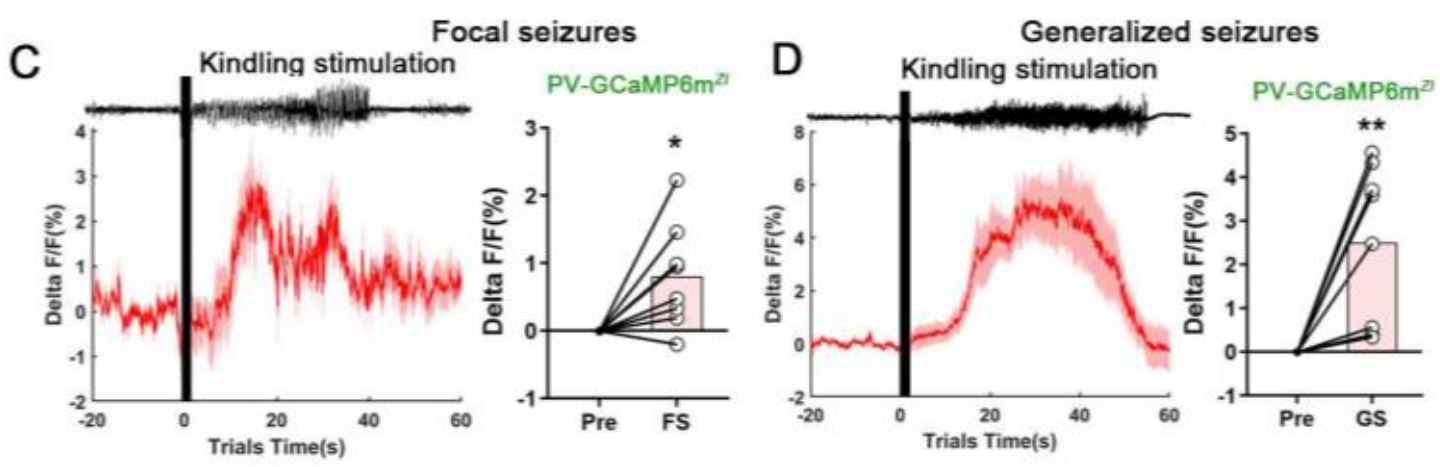

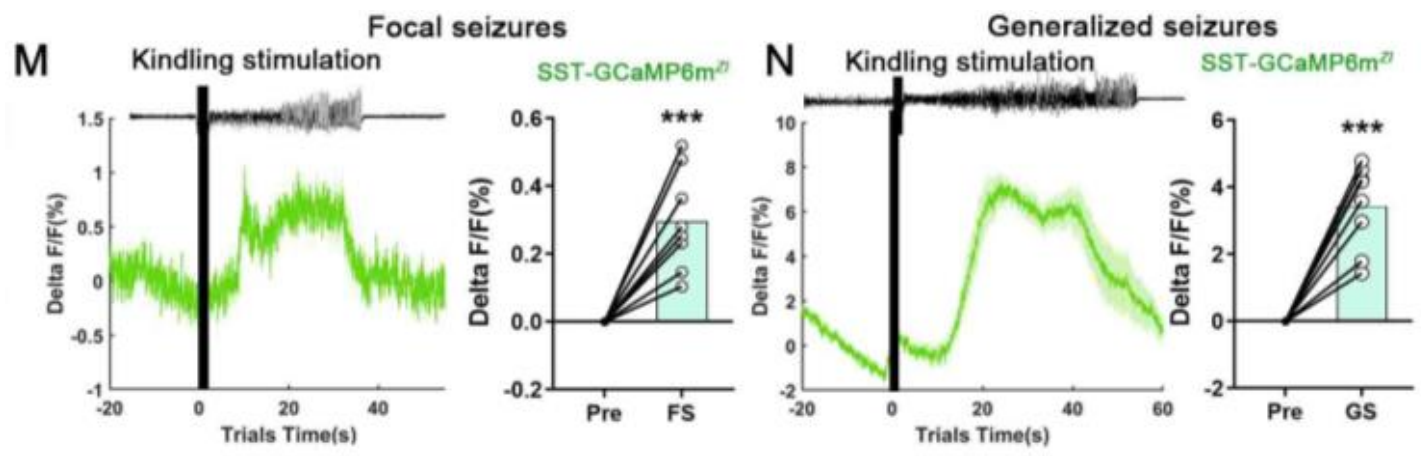

Revised Figure 4G-L:

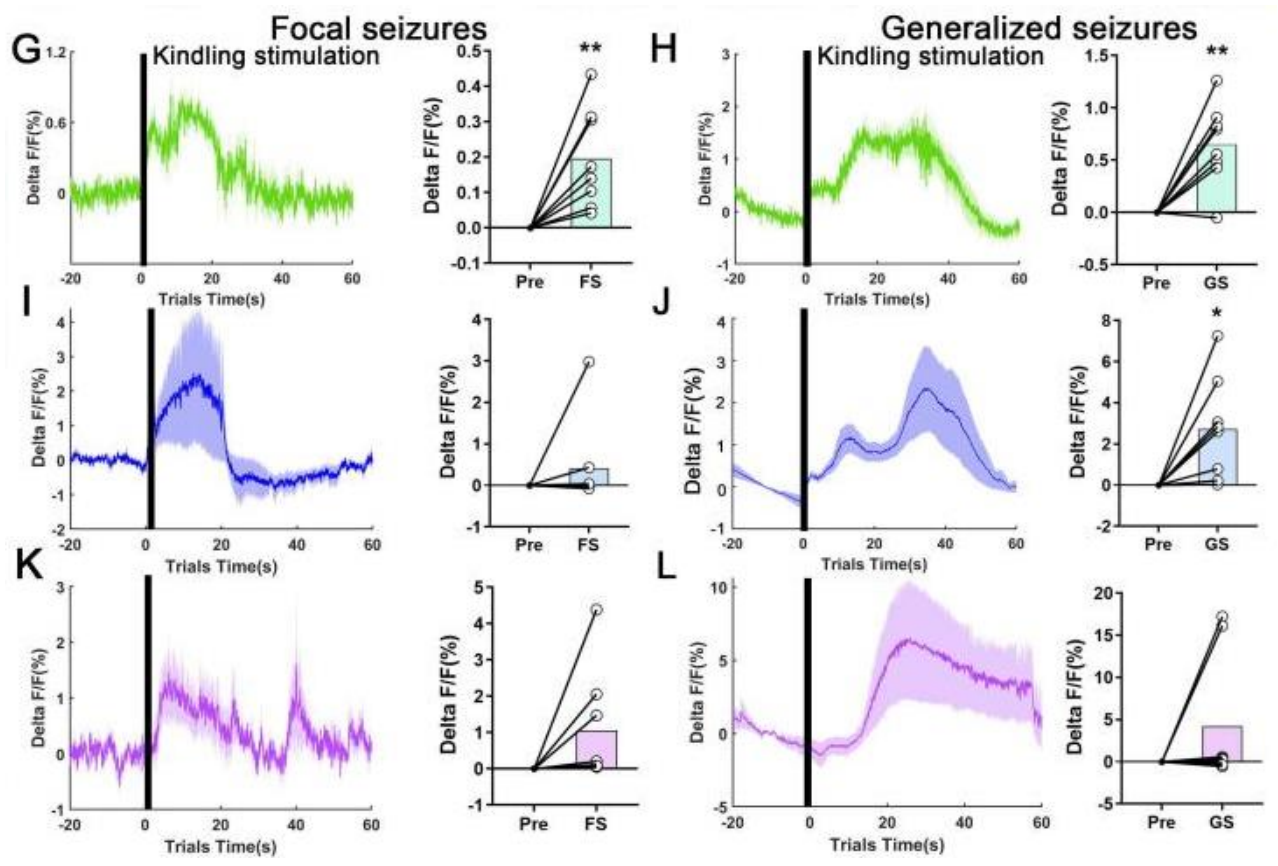

Revised Figure 6D:

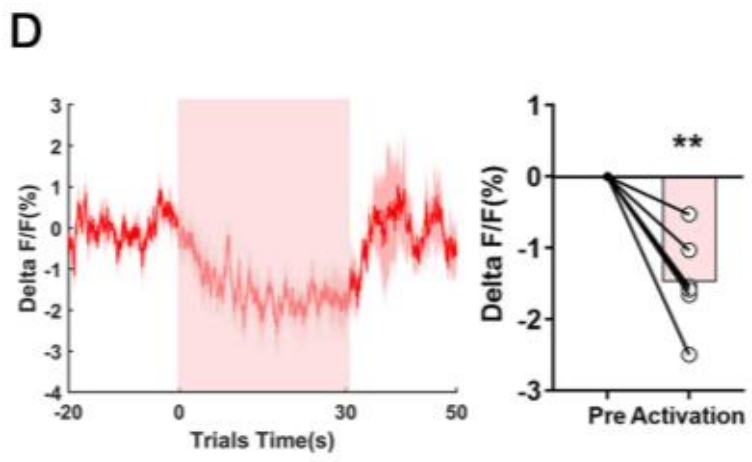

The graphs in Figure 6F and G does not portray such a difference as indicated by the noted significance in the legends. In particular the data in Figure 6G seem to be highly overlapping between the groups.

Reply: We appreciate your comment. We used the two-way repeated measures ANOVA post hoc Scheffe's test. Combining the suggestion of other reviewer, we added a control group of CNO-only. We have revised the manuscript in the results section and marked them in red (Page 13, Lines 360-363).

Page 13, Lines 360-363: To exclude potential off-target effects of CNO, CNO-only control group was conducted in animals without expressing DREADD receptors, where administration of the same CNO dose showed no significant impact on seizure progression (Fig. 6F-K).

Revised Figure 6F-K:

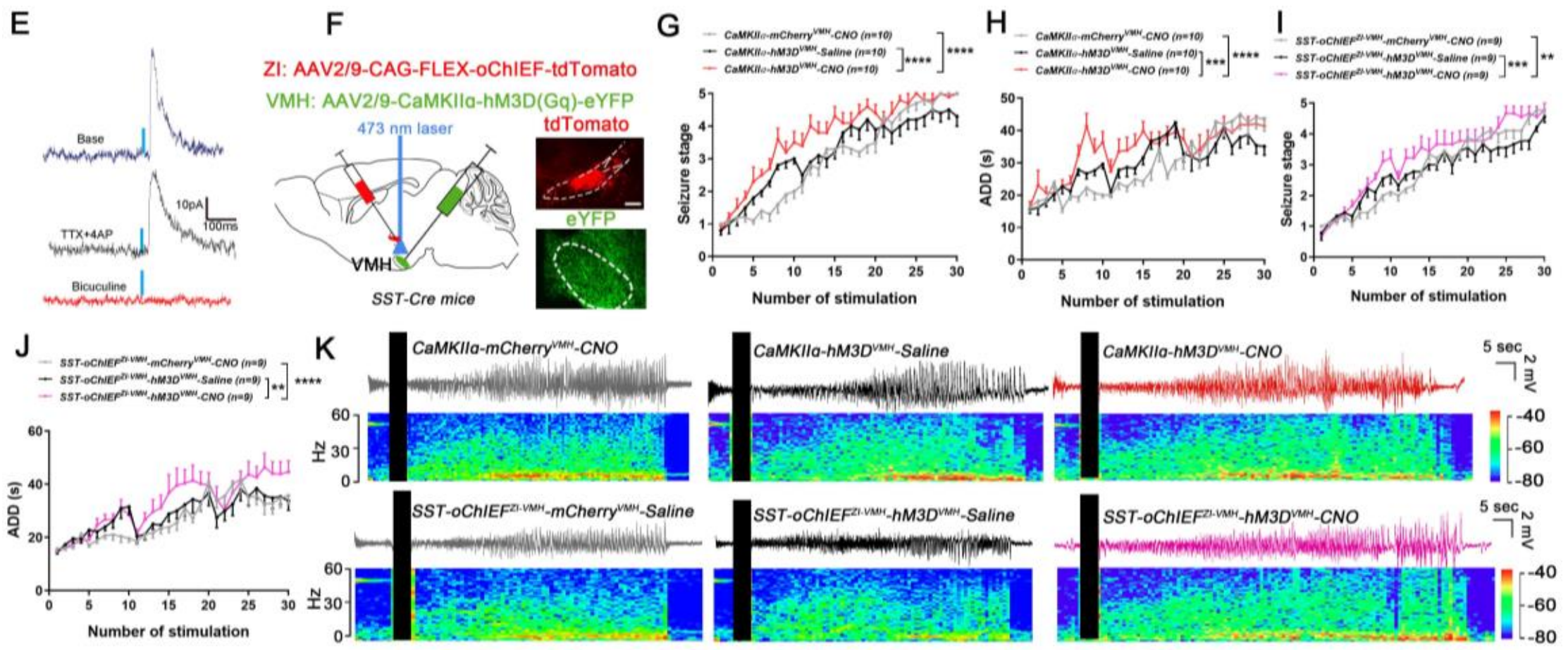

3. Controls for optogenetic/chemogenetic experiments

For chemogenetic experiments, please provide vehicle (CNO-only) control data to exclude off-target effects.

Reply: We appreciate your valuable suggestion. Following your suggestion, we performed additional control experiments. Specifically, in *SST-cre* mice lacking DREADD receptors expression (injected with AAV2/9-CAG-FLEX-oChIEF-tdTomato in the ZI and AAV2/9-CaMKIIa-eYFP in the VMH), we administered the same dose of CNO used in the CNO-only groups. We found that CNO administration in the absence of DREADD receptors did not significantly alter the seizures progression, as compared to both saline-treated controls without DREADD expression and those expressing DREADD receptors. These results confirm that CNO itself has no effects on seizures progression. Accordingly, we have revised the manuscript in the results section and marked them in red (Page 13, Lines 360-363).

Revised Figure 6F-K:

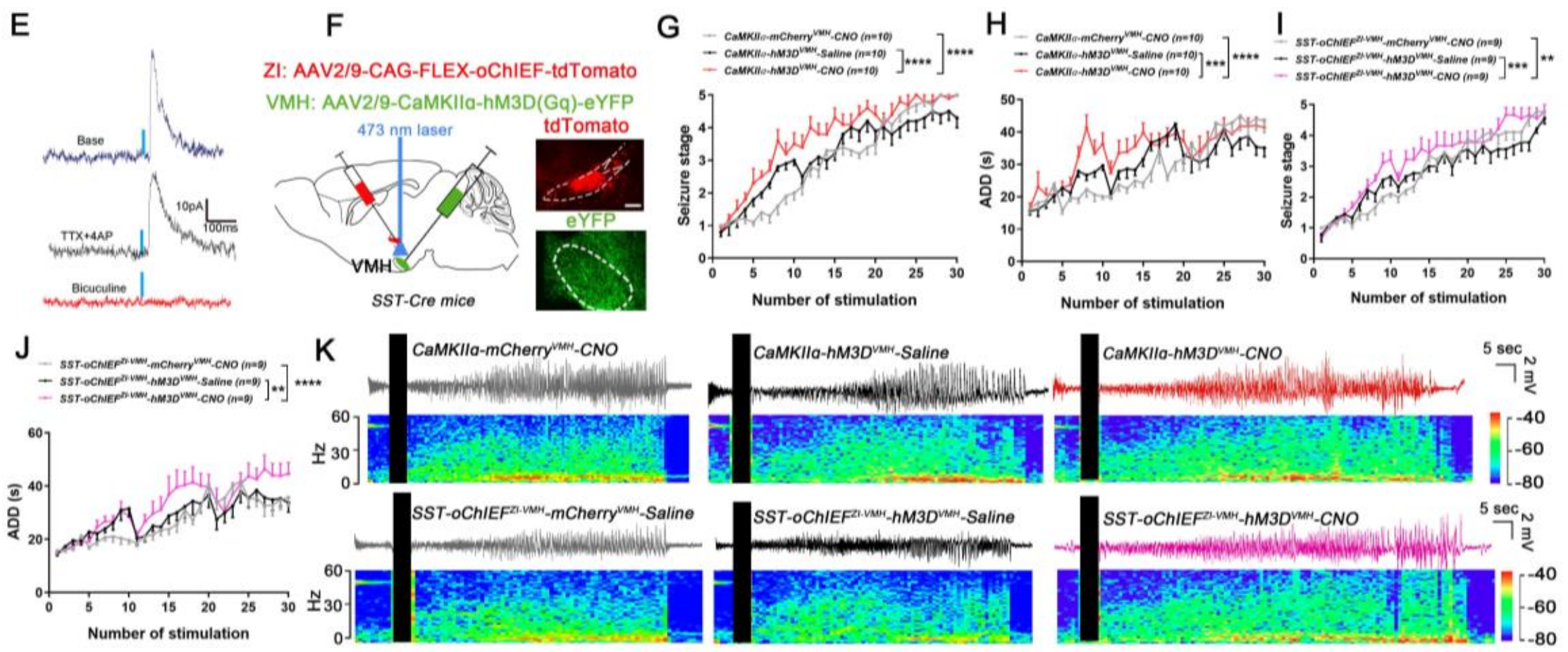

Page 13, Lines 360-363: To exclude potential off-target effects of CNO, CNO-only control group was conducted in animals without expressing DREADD receptors, where administration of the same CNO dose showed no significant impact on seizure progression (Fig. 6F-K).

For optogenetic experiments, show quantification of EEG traces with and without light

stimulation across animals, beyond representative examples.

Reply: We appreciate your valuable suggestion. All after-discharge duration (ADD) measurements reported in this study were quantified from EEG recordings obtained from every animal and every seizure event in the optogenetic experiments, rather than from representative traces alone. Thus, the ADD analysis already reflects group-level EEG quantification across animals in the optogenetic experiment.

To further substantiate the effect of optogenetic manipulation of PV and SST neurons along with the suggestion form other reviewer, we additionally analyzed EEG power during optogenetic activation of PV^+ and SST^+ neurons. The results showed that optogenetic activation of PV^+ or SST^+ neurons significantly reduced the EEG power spectra during hippocampal seizures, while optogenetic inhibition of SST^+ neurons (non-PV^+ neurons) significantly increased the EEG spectra. These quantitative analyses are now included in the revised manuscript.

Revised Supplementary Figure 6:

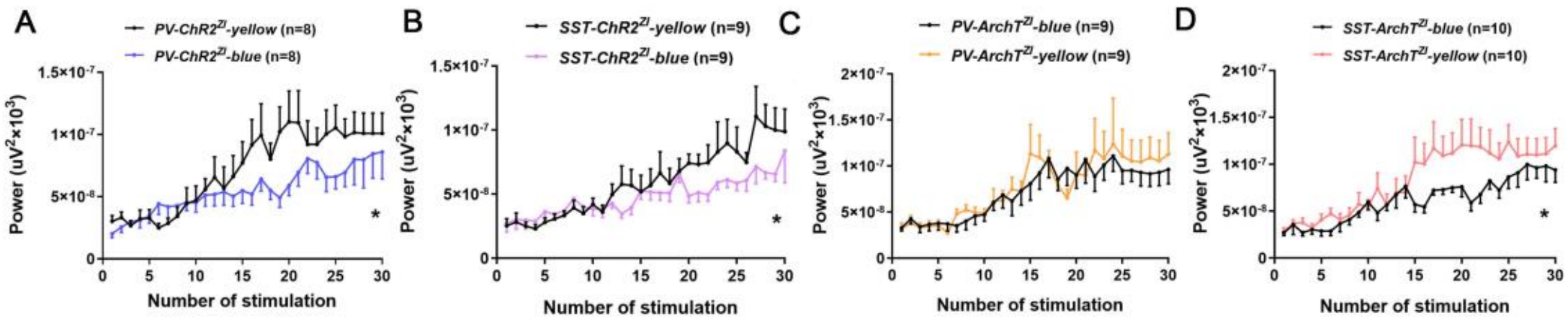

Page 7, Lines 200-201: and significantly decreased the EEG power spectra (Supplementary Fig. 6A and B).

Page 8, Lines 227-228: and increased the EEG power spectra (Supplementary Fig. 6D)

4. Photometry under anesthesia

In Fig. 1, calcium signals under isoflurane anesthesia are used to rule out movement artifacts. However, seizure dynamics under anesthesia differ substantially from awake states. The rationale and limitations of this control should be better justified in the manuscript.

Reply: We appreciate your comment. In conscious mice, intense seizures are accompanied by vigorous head and body movements, which themselves may lead to changes of the ZI calcium activity. Recording under isoflurane anesthesia ensures that the GCaMP signals reflect neuronal calcium activity rather than movement-related fluctuations. We acknowledge that isoflurane alters neuronal excitability, synaptic transmission, and network dynamics. To further validate the relevance of these signals with seizure intensity, we performed a correlation analysis between calcium activity and after-discharge duration (ADD) during seizures, revealing a positive correlation: calcium signal intensity increased with ADD under anesthesia (revised Supplementary Figure 1I). This demonstrates that, even under anesthesia, calcium signals reliably reflect seizure severity. We have updated the results section accordingly and marked them in red (Page 5, Lines 124-127).

Page 5, Lines 124-127: We also verified the increase of GCaMP fluorescence signaling in ZI GABAergic neuron during seizure in isoflurane-anesthesia mice (Supplementary Fig. 1G and H),

and the intensity of calcium signal was positively correlated with ADD during seizures (Supplementary Fig. 1I).

Revised Supplementary Figure 1I:

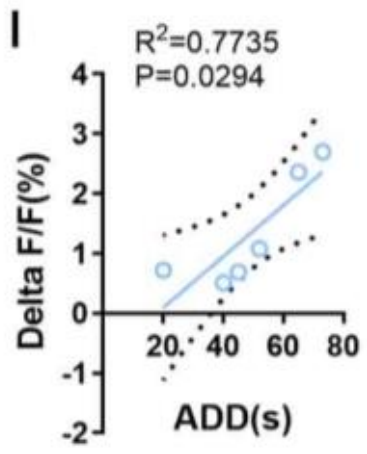

5. Circuit specificity and downstream claims

The VMH pathway is emphasized as the critical effector, but c-Fos mapping also revealed modulation in AHN and piriform cortex. Claims that the VMH is the exclusive mediator are overstated. The authors should temper conclusions or provide further experiments to strengthen specificity.

Reply: We appreciate your valuable suggestion. We agree that our initial statement that VMH was the exclusive mediator was overstated, especially in light of our own c-Fos data showing activation in AHN and piriform cortex. Mechanistically, we found that ZI SST^+ neurons project specifically to the VMH, but not to the AHN or RT, and that this circuit mediates the anti-seizure effect. For rigor and clarity, we have revised the manuscript to emphasize that the VMH represents a important downstream effector of ZI SST^+ neurons in modulating seizure propagation, without claiming it is the sole mediator. We have revised the manuscript in the results and discussion section and marked them in red (Page 4, Lines 85-88; Page 19, 535-541).

Page 4, Lines 85-88: At the circuit level, the SST^+ projection from the ZI to the ventromedial hypothalamic nucleus (VMH) represents an important circuit that contributes to bidirectional modulating seizure progression in TLE, via directly inhibition of VMH glutamatergic neurons.

Page 19, Lines 535-541: Among which, the Pir is one of the most epileptogenic and densely interconnected limbic structures. The Pir is strongly activated during the hippocampal kindling-induced seizure (Lai et al., 2022). Low-frequency electrical stimulation of the ipsilateral Pir can inhibit the seizure progression (Ghorbani et al., 2007). Therefore, after the activation of ZI^{SST}-VMH circuit exerts seizure control primarily by inhibiting VMH glutamatergic neurons and excitability of downstream regions including Pir (Canteras et al., 1994).

Given the proximity of VMH and AHN to each other, how do the authors know the specificity of their viral targeting and imaging for these regions?

Reply: We appreciate your valuable suggestion. In our immunohistochemistry analysis, viral expression and axon terminals were confined to the intended target regions in all animals. For the optic fiber placements targeting AHN were located at Bregma -0.94 mm, a rostrocaudal level at which the VMH is absent. For the optic fiber placements targeting VMH were located at Bregma -1.46 mm, a level at which the AHN is absent

Revised Supplementary Figure 9:

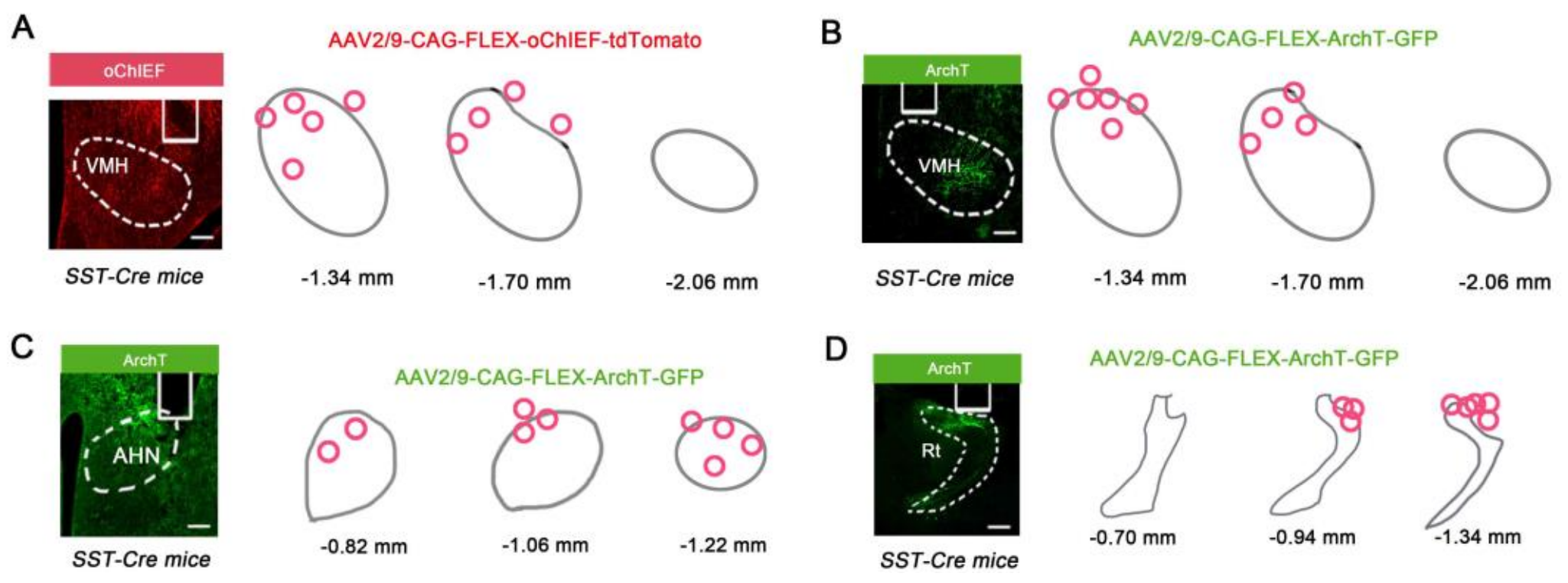

Lines 232-235 is incorrect since only VMH signal in generalized seizures is significantly higher.

Reply: We appreciate your valuable suggestion. Upon expanding our animal number and re-analyzing our fiber photometry data, we found that the calcium signal of the ZI downstream circuits was gradually elevated from FS to GS during seizure progression, especially VMH. We have revised the manuscript in the results and discussion section and marked them in red (Page 10, Lines 269-270).

Page 10, Lines 269-270: The results showed that the calcium signal of the above circuits was gradually elevated from FS to GS during seizure progression, especially VMH (Fig. 4G-M)

Regarding Supplemental Figure 3, how do the authors know that they are selectively activating the VMH targets and not the overlying ZI (based on their diagram)?

Reply: We appreciate your comment. In our immunohistochemical analysis, the viral expression and axonal terminals of all animals were confined to the predetermined target areas. For VMH targeting, the ceramic collar implant was positioned at Bregma -1.46 mm to ensure precise aiming without including AHN. The coronal cross-sections of the VMH and ZI in the same diagram, and the optical fibers trajectory and terminal have also been verified. The terminals of optical fibers are all located below ZI, so the range of light irradiation does not include ZI.

Figure to reviewer:

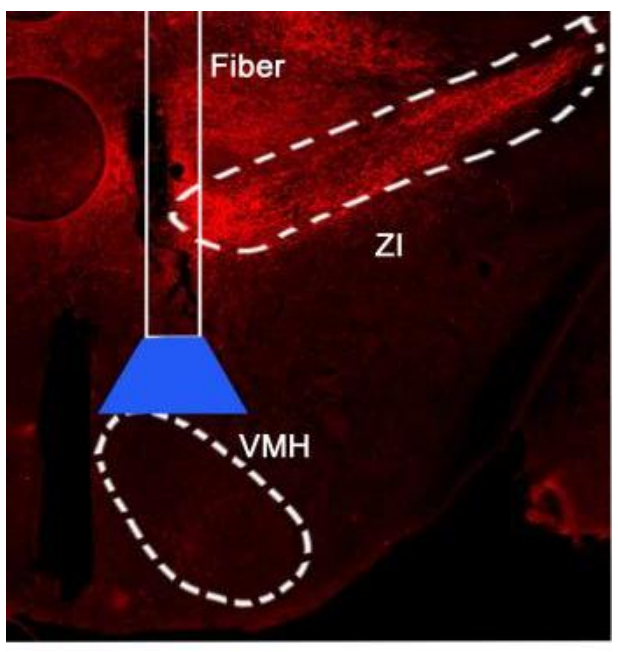

Revised Supplementary Figure 9A and B

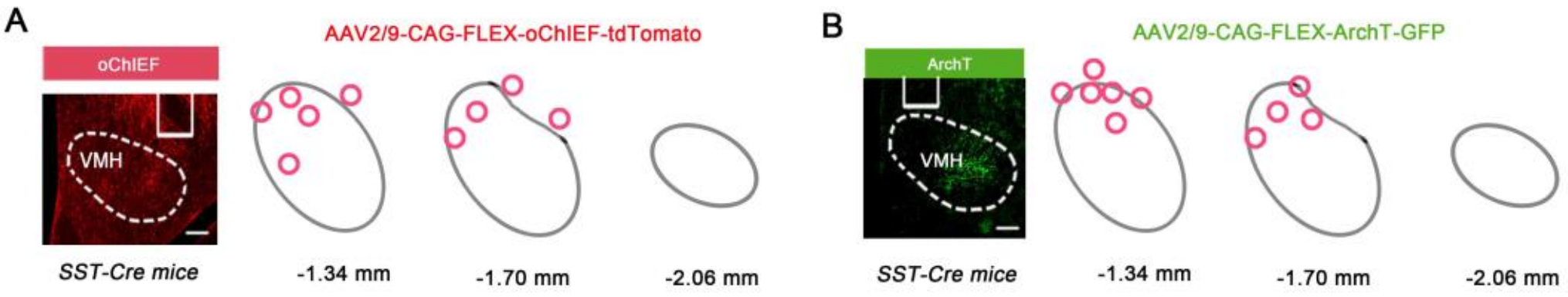

Diagram in Figure 6 suggests that SST neurons projecting to VMH also project to other brain areas. What are these other brain areas and why don't they exert a similar effect?

Reply: We appreciate your valuable comments. SST neurons project to both the VMH and AHN, but modulation of the ZI^{SST}-AHN and ZI^{SST}-VMH circuit produces distinct effects on seizures. To investigate the mechanistic basis of these differences, we injected AAV2/2Retro-hEF1a-DIO-EGFP and AAV2/2Retro-hEF1a-DIO-mCherry into the VMH and AHN, respectively. Quantitative analysis across ZI revealed heterogeneous projection patterns: AHN-projecting SST neurons originate from the ZIv (30.58%) and ZIr (64.29%), whereas those VMH-projecting SST neurons are concentrated in the ZIr (56.07%) (revised Supplementary Figure 12), suggesting different subpopulations of SST neurons project to AHN and VMH respectively. Furthermore, we observed that after hippocampal seizures, c-Fos activation in SST^{+} neurons was specifically and significantly enriched in the ZIr. This finding is highly consistent with our retrograde tracing and optogenetic functional results.

Meanwhile, the VMH is mainly composed of excitatory glutamatergic neurons, accounting for approximately 90% (Kim et al., 2019), while in the AHN, about 92% of the neurons are inhibitory GABAergic neurons and 8% are excitatory glutamatergic neurons (Xie et al., 2022). Our experiments revealed that activation of the ZI^{SST}-VMH circuit alleviated seizures by inhibiting the VMH glutamatergic neurons; while activation of ZI^{SST}-AHN circuit exacerbates epileptic seizures may through inhibiting the AHN GABAergic intermediate neurons. The above provide explanation for distinct effects mediating by ZI^{SST}-AHN and ZI^{SST}-VMH circuit. We have revised the manuscript in the results and discussion section and marked them in red (Page 11, Lines 307-313).

Revised Supplementary Figure 12:

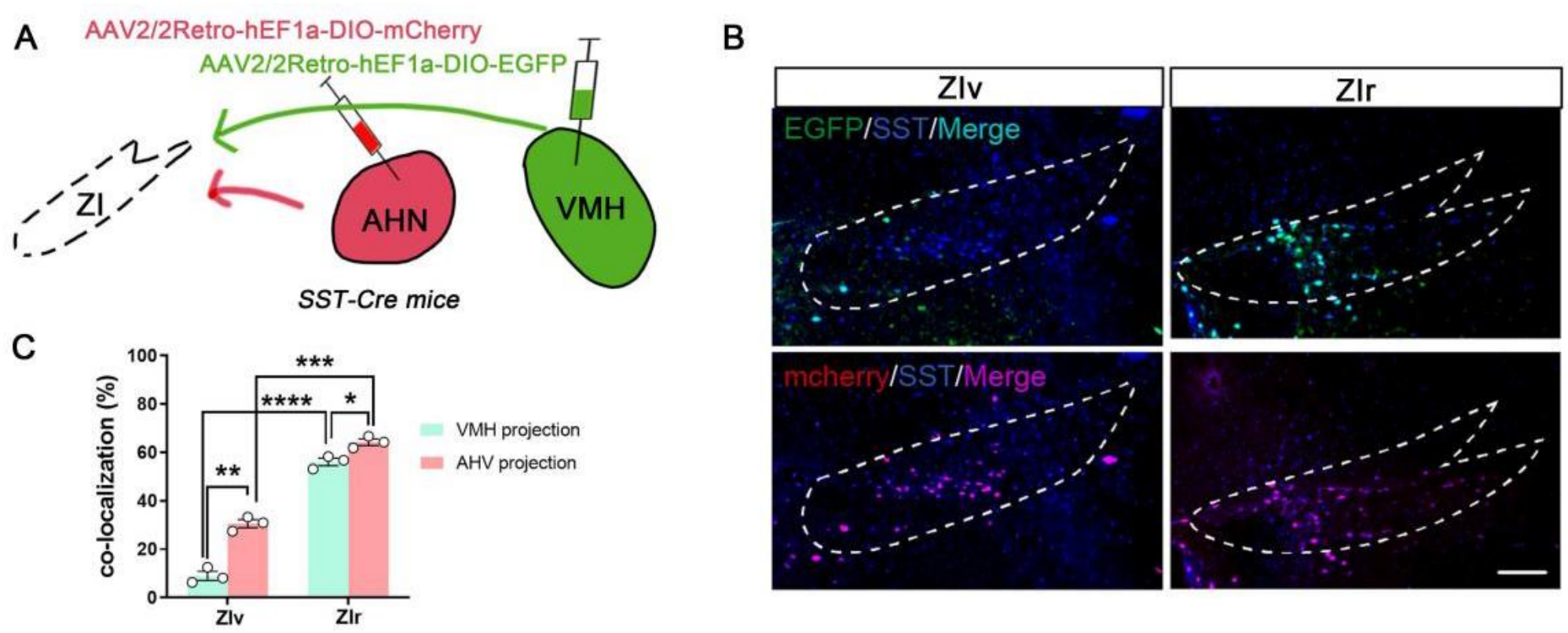

Page 11, Lines 307-313: Regarding modulation of the ZI-AHN circuit produces effects distinct from those of the ZI-VMH circuit. To explore the mechanistic basis of this divergence, we injected AAV2/2Retro-hEF1α-DIO-EGFP and AAV2/2Retro-hEF1α-DIO-mCherry into the VMH

and AHN, respectively. Quantitative mapping across the ZI revealed heterogeneous projection patterns: AHN-projecting SST neurons predominantly originate from the ZIv (30.58%) and ZIr (64.29%), whereas VMH-projecting SST neurons are concentrated in the ZIr (56.07%) (Supplementary Fig. 12).

6. Relationship to prior work by the same group

The authors previously reported (Zhang et al., 2024) that ZI GABAergic neurons, targeted via Vgat-Cre, are recruited during seizures, with bidirectional manipulation affecting seizure progression. The present manuscript extends this work by dissecting ZI interneuron subtypes and demonstrating that SST+ neurons — but not PV+ neurons — are both necessary and sufficient for seizure suppression. The connection to their prior work should be made more explicit: how does the broad GABAergic effect map onto the subtype-specific findings? Does this mean that the anti-seizure effect observed in the earlier study was primarily mediated by SST+ neurons, while PV+ contributions were secondary or redundant? Clarifying this progression is essential for readers to understand the added value of the current work.

Reply: We appreciate your valuable suggestions. Indeed, ZI GABAergic neurons bidirectionally modulated seizure, which were primarily driven by subtypes of SST^+ neurons. This functional specificity may be related to the different projection targets of SST^+ and PV^+ neurons (revised Figure 4E and revised Supplementary Figure 16). SST^+ neurons may mainly send long-range projections to the anterior part of the brain nucleus to exert more precise and powerful inhibitory control on key pathways in the seizure network, while PV^+ neurons mainly send projections to the posterior part of the brain nucleus to participate in other functions. Interestingly, however, activation of either PV^+ or SST^+ neurons exerts an anti-seizure effect. Our earlier work did not distinguish GABAergic neuron subtypes with precision, and thus we could not exclude whether DBS acts by enhancing the function of specific subpopulations. The present study further resolves this cell-type-specific role in seizure, which will help lay the foundation for precise neuromodulation in the future. Based on your comments, we have added more discussion related with our previous work in the revised manuscript (Page 14, Lines 389-394).

Page 14, Lines 389-394: Our earlier work did not distinguish GABAergic neuron subtypes, and thus we could not exclude whether DBS acts by enhancing the function of specific subpopulations. Extending our previous work, we delineate a cell-type- and projection-defined circuit mechanism that positions the ZI as a key regulator of epileptic activity which will help lay the foundation for precise neuromodulation in the future.

Minor Points

1. The authors note the Pir as an ictogenic trigger but it is unclear how this is related to their specific hippocampal kindling epilepsy model and to their seizure circuit where Pir is downstream of ZI and VMH.

Reply: We appreciate your valuable suggestion. The Pir is one of the most epileptogenic and densely interconnected limbic structures. It is strongly activated during the hippocampal kindling-induced seizure (Lai et al., 2022). Low-frequency electrical stimulation of the ipsilateral Pir can inhibit the seizure progression (Ghorbani et al., 2007). Therefore, after the ZI^{SST}-VMH circuit is

activated, it exerts seizure control primarily by inhibiting VMH glutamatergic neurons. This in turn, decreases the excitability of downstream regions including the Pir (Canteras et al., 1994), thereby suppressing the progression of epileptic seizures. We have revised the manuscript in the discussion section and marked them in red (Page 18, Lines 535-541).

Page 18, Lines 535-541: Among which, the Pir is one of the most epileptogenic and densely interconnected limbic structures. The Pir is strongly activated during the hippocampal kindling-induced seizure (Lai et al., 2022). Low-frequency electrical stimulation of the ipsilateral Pir can inhibit the seizure progression (Ghorbani et al., 2007). Therefore, after the activation of ZI^{SST}-VMH circuit exerts seizure control primarily by inhibiting VMH glutamatergic neurons and excitability of downstream regions including Pir (Canteras et al., 1994).

2. Lines 160-162 in reference to Figure 2D and N need to specify that this is only the case for generalized seizures.

Reply: We appreciate your valuable suggestion. We have revised original description to “Further analysis of the calcium signal duration of PV^+ and SST^+ neurons during hippocampal seizure revealed that SST^+ neurons tended to exhibit longer calcium signal duration and a faster increase in calcium signals, as indicated by the steeper slope in SST during GS compared to PV^+ neurons (Supplementary Fig. 4A and B).” in Page 7, Lines 188-191.

Page 7, Lines 188-191: Further analysis of the calcium signal duration of PV^+ and SST^+ neurons during hippocampal seizure revealed that SST^+ neurons tended to exhibit a faster increase in calcium signals with a slightly longer duration, as indicated by the steeper slope in SST during GS compared to these of PV^+ neurons (Supplementary Fig. 4A and B).

3. It is unclear why the authors decided to use continuous application of yellow light for their optogenetic inactivation experiments which may induce phototoxicity and desensitization of the channel.

Reply: We appreciate your comment. As a light-driven proton pump, Arch requires continuous illumination to activate and hyperpolarize target neurons (Eldar et al., 2026). Our optogenetic parameters-including continuous light stimulation-were strictly aligned with the established protocol from Kinman et al., 2025; Stegemann et al., 2023; and McHugh et al., 2022, which has been demonstrated to be effective and well-tolerated. Furthermore, we provide real-time light exposure based on the duration of seizures induced by the kindling-induced seizure model, which usually lasts for about 30 seconds. Crucially, all control animals received identical light stimulation (same duration and intensity), thus excluding any observed behavioral differences as being attributable to non-specific light effects.

4. Improve figure legends: clearly state n per group, units, and definitions of error bars (SEM vs SD).

Reply: We appreciate your valuable suggestion. We have comprehensively revised all the figure legends in the revised manuscript in accordance with your suggestions. It clearly states the sample size of each group. The units have been supplemented. All error bars are clearly defined as standard errors (SEM). The corresponding modifications can be found in the figure legends

section of the revised manuscript.

5. Correct terminology: e.g., "glutaminergic" should be replaced with "glutamatergic."
Reply: Thanks. We have revised "glutaminergic" to "glutamatergic". We have revised the manuscript in the results section and marked them in red (Page 13, Lines 352-359).

6.Improve English clarity throughout (many small grammar issues: e.g., "are bidirectionally modulate seizure" → "bidirectionally modulate seizure").
Reply: Thanks. We have revised "are bidirectionally modulate seizure" to "bidirectionally modulate seizure" and checked the grammatical issues throughout the manuscript to enhance the readability.

In the first sentence of the abstract "pivotal regulator" should be "pivotal regulators".
Reply: Thanks. We have revised "pivotal regulator" to "pivotal regulators" We have revised the manuscript in the abstract section and marked them in red (Page 2, Line 22).

In line 60 "types" should be "type"
Paragraph 59 to 63 is confusing as it is: I suggest revising to something like: "Among epilepsy subtypes, temporal lobe epilepsy (TLE) is one of the most common drug-resistant forms, with seizures originating in limbic regions—particularly the hippocampus (Englot et al., 2020; Keezer et al., 2016)—and often spreading to generalized seizures (GS) due to the complex epileptogenic networks underlying TLE."
Reply: Thanks. We have revised original description in the introduction section and marked them in red (Page 3, Lines 58-61).

In line 70 "implicating" should be "implicated".
Reply: Thanks. We have revised "implicating" to "implicated" in the revised manuscript (Page 3, Line 68).

Discuss discrepancies with prior literature: several studies (e.g., Yekhlef et al., 2015; Chen et al., 2020) report that SST+ or PV+ activation can promote seizures in other circuits. How do these findings reconcile with the present data?
Reply: We appreciate your valuable comment. Previous studies (Chen et al., 2020; Yekhlef et al., 2015) have showed that activated of SST^+ or PV^+ neurons can promote seizures in other circuits, in contrast to the anti-seizures effect observed in our study. The research of Yekhlef, Chen et al. focused primarily on SST^+/PV^+ interneurons within the entorhinal cortex or substantia nigra, while our research focused on ZI. The PV^+ or SST^+ neurons in the entorhinal cortex that are activated will trigger a large amount of GABA release, causing depolarization of the spinal neurons and a significant increase in extracellular K^+ concentration. This further depolarizes the surrounding neurons, forming a positive feedback loop, which induces synchronous bursts and epileptic-like discharges. The PV^+ neurons in the substantia nigra, project to the GABAergic neurons in the downstream PF, inhibiting their functions and thereby promoting the synchronization and progression of the epileptic network. However, the mechanism by which the activated ZI SST^+ neurons in our study exert anti-seizure effects is through projecting to and inhibiting the VMH

glutamatergic neurons, thereby suppressing the epileptic network. The apparent contradiction actually highlights the nucleus-specific of neural circuits, which may also be likely attributed to the heterogeneity of connection patterns, depolarized GABAergic signaling, co-expression of neurotransmitters et al.. In order to better integrate the previous studies, we have revised the manuscript in the discussion section and marked them in red (Page 16, Lines 467-478).

Page 16, Lines 467-478: The research of Yekhlef, Chen et al. focused primarily on SST^+/PV^+ interneurons within the entorhinal cortex or nigra, while our research focused on the ZI. The PV^+ or SST^+ neurons in the entorhinal cortex that are activated will trigger a large amount of GABA release, causing depolarization of the spinal neurons and a significant increase in extracellular K^+ concentration. This further depolarizes the surrounding neurons, forming a positive feedback loop, which induces synchronous bursts and epileptic-like discharges. The PV^+ neurons in the substantia nigra project to the GABAergic neurons in the downstream PF, inhibiting their functions and thereby promoting the synchronization and progression of the epileptic network. However, the mechanism by which the activated ZI SST^+ neurons in our study exert anti-seizure effects is through projecting to the VMH and inhibiting glutamatergic neurons, thereby suppressing the epileptic network.

7. Methods: injection volumes of 200 μL (line 461) seem unusually large for stereotactic mouse injections — this should be checked, possibly a typo for 200 nL.
Reply: We sincerely apologize for this writing error. The actual volume used for stereotactic injections in mice was 200 nL, we have corrected this mistake in the revised manuscript Page 21 Line 607 and double-checked all other part-related descriptions in the Methods section to ensure no similar errors exist.

8. It is unclear why an ArchT virus was used solely for projection mapping in Figure 4A.
Reply: We appreciate your valuable suggestion. The AAV2/9-CAG-FLEX-ArchT-GFP virus with a strong CAG promoter, which drives high expression of the fused GFP, which guarantees brighter fluorescent labeling of axonal projections compared to some low-expression fluorescent reporter viruses, allowing fine axonal arborizations in the downstream areas (the target region in revised Figure 4B).

9. In Figure 4D is the c-Fos in hippocampus significantly higher in the blue light condition?
Reply: We appreciate your valuable suggestion. Although we observed an increasing trend of hippocampal c-Fos in the blue light activation group without statistical significance (revised Figure 4D, $P = 0.2656$). As an immediate early gene, c-Fos expression is an activity-dependent marker that reports neuronal engagement over a broad temporal window, but does not distinguish between physiological firing and pathological hypersynchronous activity. The slight, non-significant increase in hippocampal c-Fos following ZI SST activation therefore suggests that ZI SST neurons suppress seizures by reducing abnormal network synchronization rather than by inhibiting hippocampal neuronal activity. Such circuit modulation is consistent with the known role of SST neurons in limiting dendritic integration and burst firing while sparing baseline activity (Adler et al., 2019).

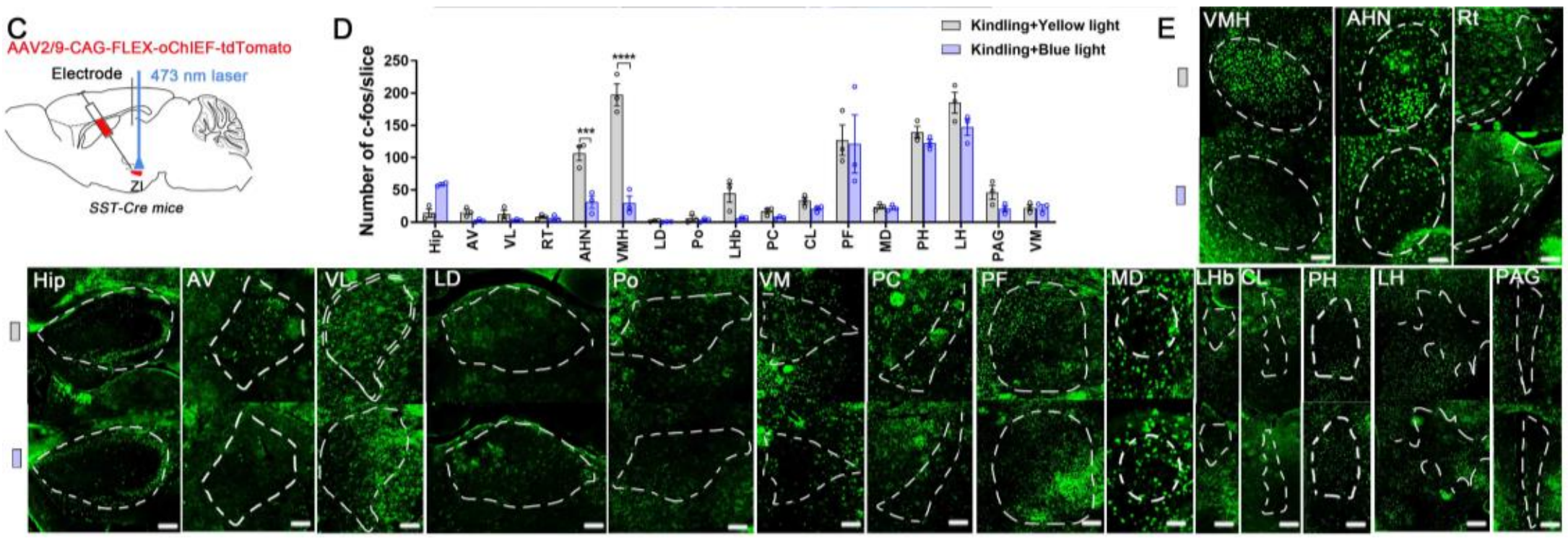

10. Line 245 "decreased" should be "increased".

Reply: We sincerely thank you for pointing out this careless error. We have revised "decreased" to "increased" in the results section and marked them in red (Page 10, Lines 282).

Reference

Adler A, Zhao R, Shin ME, Yasuda R, and Gan WB. (2019). Somatostatin-Expressing Interneurons Enable and Maintain Learning-Dependent Sequential Activation of Pyramidal Neurons. *Neuron, 102*(1), 202-216 e207.

Arena G, Londei F, Ceccarelli F, Ferrucci L, Borra E, and Genovesio A. (2024). Disentangling the identity of the zona incerta: a review of the known connections and latest implications. *Ageing Res Rev, 93*, 102140. doi:10.1016/j.arr.2023.102140

Blumberg BJ, Flynn SP, Barriere SJ, Mouchati PR, Scott RC, Holmes GL, et al. (2016). Efficacy of nonselective optogenetic control of the medial septum over hippocampal oscillations: the influence of speed and implications for cognitive enhancement. *Physiol Rep, 4*(23), e13048. doi: 10.14814/phy2.13048.

Canteras NS, Simerly RB, and Swanson LW. (1994). Organization of projections from the ventromedial nucleus of the hypothalamus: a Phaseolus vulgaris-leucoagglutinin study in the rat. *J Comp Neurol, 348*(1), 41-79. doi:10.1002/cne.903480103

Chang CH, and Gean PW. (2019). The Ventral Hippocampus Controls Stress-Provoked Impulsive Aggression through the Ventromedial Hypothalamus in Post-Weaning Social Isolation Mice. *Cell Rep, 28*(5), 1195-1205 e1193. doi:10.1016/j.celrep.2019.07.005

Chen B, Xu C, Wang Y, Lin W, Wang Y, Chen L, et al. (2020). A disinhibitory nigra-parafascicular pathway amplifies seizure in temporal lobe epilepsy. *Nat Commun, 11*(1), 923. doi:10.1038/s41467-020-14648-8

Chen J, Kriegstein AR. (2015). A GABAergic projection from the zona incerta to cortex promotes cortical neuron development. *Science, 350*(6260), 554-558

Cheng H, Lou Q, Lai N, Chen L, Zhang S, Fei F, et al. (2023). Projection-defined median raphe Pet(+) subpopulations are diversely implicated in seizure. *Neurobiol Dis, 189*, 106358. doi:10.1016/j.nbd.2023.106358

Chou XL, Wang X, Zhang ZG, Shen L, Zingg B, Huang J, et al. (2018). Inhibitory gain modulation of defense behaviors by zona incerta. *Nat Commun, 9*(1), 1151. doi: 10.1038/s41467-018-03581-6

Chow BY, Han X, Dobry AS, Qian X, Chuong AS, Li M, et al. (2010). High-performance genetically targetable optical neural silencing by light-driven proton pumps. *Nature, 463*(7277):98-102. doi: 10.1038/nature08652.

Dragunow M, and Robertson HA. (1987). Kindling stimulation induces c-fos protein(s) in granule cells of the rat dentate gyrus. *Nature, 329*(6138), 441-442. doi:10.1038/329441a0

Duan X, Zhang C, Wu Y, Ju J, Xu Z, Li X, et al. (2025). Suppression of epileptic seizures by transcranial activation of K(+)-selective channelrhodopsin. *Nat Commun, 16*(1):559. doi: 10.1038/s41467-025-55818-w

Eichenbaum H. (2004). Hippocampus: cognitive processes and neural representations that underlie declarative memory. *Neuron, 44*(1), 109-20. doi: 10.1016/j.neuron.2004.08.028

Eldar D, Albert S, Tatyana A, Galina S, Albert R, and Yana M. (2026). Optogenetic approaches for neural tissue regeneration: A review of basic optogenetic principles and target cells for therapy. *Neural Regeneration Research, 21*(2), 521-533.

Etemadi F, Sayyah M, Gholami Pourbadie H, and Babapour V. (2015). Assessment of the Optimal Stimulus Pattern to Achieve Rapid Dorsal Hippocampal Kindling in Rats. *Basic Clin Neurosci, 6*(2):107-112

Farrell JS, Colangeli R, Dudok B, Wolff MD, Nguyen SL, Jackson J, et al. (2020). In vivo assessment of mechanisms underlying the neurovascular basis of postictal amnesia. *Sci Rep, 10*(1), 14992.

Fei F, Wang X, Xu C, Shi J, Gong Y, Cheng H, et al. (2022). Discrete subicular circuits control generalization of hippocampal seizures. *Nat Commun, 13*(1), 5010. doi:10.1038/s41467-022-32742-x

Furman KL, Baron L, Lyons HC, Cha T, Evans JR, Manna J, et al. (2024). Melanin concentrating hormone projections to the nucleus accumbens enhance the reward value of food consumption and do not induce feeding or REM sleep. *bioRxiv.* doi: 10.1101/2024.11.11.622987

Ghorbani P, Mohammad-Zadeh M, Mirnajafi-Zadeh J, and Fathollahi Y. (2007). Effect of different patterns of low-frequency stimulation on piriform cortex kindled seizures. *Neurosci Lett, 425*(3), 162-166. doi:10.1016/j.neulet.2007.08.023

Grimm C, Duss SN, Privitera M, Munn BR, Karalis N, Frässle S, et al. (2024). Tonic and burst-like locus coeruleus stimulation distinctly shift network activity across the cortical hierarchy. *Nat Neurosci, 27*(11), 2167-2177. doi: 10.1038/s41593-024-01755-8

Hintiryan H, Rudd M, Nanda S, Gutierrez AE, Lo D, Boesen T, et al. (2025). Distinct subnetworks of the mouse anterior thalamic nuclei. *Nat Commun, 16*(1), 6018. doi: 10.1038/s41467-025-60774-6

Khoshkhoo S, Vogt D, and Sohal VS. (2017). Dynamic, Cell-Type-Specific Roles for GABAergic Interneurons in a Mouse Model of Optogenetically Inducible Seizures. *Neuron, 93*(2), 291-298. doi: 10.1016/j.neuron.2016.11.043.

Kim DW, Yao Z, Graybuck LT, Kim TK, Nguyen TN, Smith KA, et al. (2019). Multimodal Analysis of Cell Types in a Hypothalamic Node Controlling Social Behavior. *Cell, 179*(3):713-728.e17. doi: 10.1016/j.cell.2019.09.020

Krook-Magnuson E, Armstrong C, Oijala M, and Soltesz I. (2013).On-demand optogenetic control of spontaneous seizures in temporal lobe epilepsy. *Nat Commun, 4,* 1376. doi: 10.1038/ncomms2376

Kinman AI. Merryweather DN. Erwin SR. Campbell RE. Sullivan KE. Kraus L, et al. (2025). Atypical hippocampal excitatory neurons express and govern object memory. *Nat Commun, 16*(1), 1195. doi: 10.1038/s41467-025-56260-8

Lai N, Cheng H, Li Z, Wang X, Ruan Y, Qi Y, et al. (2022). Interictal-period-activated neuronal ensemble in piriform cortex retards further seizure development. *Cell Rep, 41*(11):111798. doi: 10.1016/j.celrep.2022.111798.

Ledri M, Madsen MG, Nikitidou L, Kirik D, and Kokaia M. (2014). Global optogenetic activation of inhibitory interneurons during epileptiform activity. *J Neurosci, 34*(9), 3364-3377.

Li ZL, Rizzi G, and Tan KR. (2021). Zona incerta subpopulations differentially encode and modulate anxiety. *Sci Adv. 7*(37):eabf6709. doi: 10.1126/sciadv.abf6709

Lin, C. S. Nicolelis, M. A. Schneider, J. S. Chapin, J. K. (1990). A major direct GABAergic pathway from zona incerta to neocortex. *Science, 248*(4962), 1553-1556. doi: 10.1126/science.2360049.

Lo L, Yao S, Kim DW, Cetin A, Harris J, Zeng H, et al. (2019). Connectional architecture of a mouse hypothalamic circuit node controlling social behavior. *Proc Natl Acad Sci U S A, 116*(15), 7503-7512. doi: 10.1073/pnas.1817503116

McHugh SB, Lopes-Dos-Santos V, Gava GP, Hartwich K, Tam SKE, Bannerman DM. (2022). Adult-born dentate granule cells promote hippocampal population sparsity. *Nat Neurosci, 25*(11), 1481-1491. doi: 10.1038/s41593-022-01176-5

McNamara JO, Byrne MC, Dasheiff RM, and Fitz JG. (1980). The kindling model of epilepsy: a review. *Prog Neurobiol, 15*(2), 139-159. doi:10.1016/0301-0082(80)90006-4

Mitrofanis J. (2005). Some certainty for the "zone of uncertainty"? Exploring the function of the zona incerta. *Neuroscience, 130*(1), 1-15. doi:10.1016/j.neuroscience.2004.08.017

Mitrofanis J, Ashkan K, Wallace BA, and Benabid AL. (2004). Chemoarchitectonic heterogeneities in the primate zona incerta: clinical and functional implications. *J Neurocytol, 33*(4), 429-440. doi:10.1023/B:NEUR.0000046573.28081.dd

Power BD, Leamey CA, and Mitrofanis J. (2001). Evidence for a visual subsector within the zona incerta. *Vis Neurosci, 18*(2), 179-186. doi:10.1017/s0952523801182027

Qi Y, Cheng H, Lou Q, Wang X, Lai N, Gao C, et al. (2022). Paradoxical effects of posterior intralaminar thalamic calretinin neurons on hippocampal seizure via distinct downstream circuits. *iScience, 25*(5), 104218.

Ribierre, T. Bacq, A. Donneger, F. Doladilhe, M. Maletic, M. Roussel, D. et al. (2024). Targeting pathological cells with senolytic drugs reduces seizures in neurodevelopmental mTOR-related epilepsy. *Nat Neurosci, 27*(6),1125-1136. doi: 10.1038/s41593-024-01634-2

Stegemann A, Liu S, Retana Romero OA, Oswald MJ, Han Y, Beretta CA, et al. (2023). Prefrontal engrams of long-term fear memory perpetuate pain perception. Nat Neurosci, 26(5), 820-829. doi: 10.1038/s41593-023-01291-x

Watanabe Y, Johnson RS, Butler LS, Binder DK, Spiegelman BM, Papaioannou VE, et al. (1996). Null mutation of c-fos impairs structural and functional plasticities in the kindling model of epilepsy. J Neurosci, 16(12), 3827-3836.

Wang Q, Zhang B, Stutz B, Liu ZW, Horvath TL, and Yang X. (2022). Ventromedial hypothalamic OGT drives adipose tissue lipolysis and curbs obesity. Sci Adv, 8(35), eabn8092. doi: 10.1126/sciadv.abn8092

Wu C, Leung LS. (1997). Partial hippocampal kindling decreases efficacy of presynaptic GABAB autoreceptors in CA1. J Neurosci, 17(23), 9261-9. doi: 10.1523/JNEUROSCI.17-23-09261.1997.

Xie Z, Gu H, Huang M, Cheng X, Shang C, Tao T, et al. (2022). Mechanically evoked defensive attack is controlled by GABAergic neurons in the anterior hypothalamic nucleus. Nat Neurosci, 25(1):72-85.doi: 10.1038/s41593-021-00985-4

Yang Y, Jiang T, Jia X, Yuan J, Li X, Gong H. (2022). Whole-Brain Connectome of GABAergic Neurons in the Mouse Zona Incerta. *Neurosci Bull, 38*(11), 1315-1329.

Yekhlef L, Breschi GL, Lagostena L, Russo G, and Taverna S. (2015). Selective activation of parvalbumin- or somatostatin-expressing interneurons triggers epileptic seizurelike activity in mouse medial entorhinal cortex. *J Neurophysiol, 113*(5), 1616-1630. doi:10.1152/jn.00841.2014

Zhao J, Sun J, Zheng Y, Zheng Y, Shao Y, Li Y, et al. (2022). Activated astrocytes attenuate neocortical seizures in rodent models through driving Na(+)-K(+)-ATPase. *Nat Commun, 13*(1), 7136. doi:10.1038/s41467-022-34662-2

Zhu M, Peng J, Wang M, Lin S, Zhang H, Zhou Y. (2025). Transcriptomic and spatial GABAergic neuron subtypes in zona incerta mediate distinct innate behaviors. *Nat Commun,*

16(1):3107. doi: 10.1038/s41467-025-57896-2.

Reviewer #1

The authors has well addressed my concerns. I have no additional comments.

Reply: Thank you very much for your positive feedback. We greatly appreciate your time and effort in reviewing our manuscript, and we are pleased that our revised version has satisfactorily addressed your concerns.

Reviewer #2

The manuscript has been significantly improved; however, I still have some questions and concerns that I would like to discuss with the author.

Reply: We sincerely appreciate the time and effort you have taken to review our manuscript and for the valuable comments, which have significantly contributed to the revision and improvement of our paper. In this letter, we address each of your comments individually, followed by our corresponding responses. For clarity, we have restated your comments in bold font and provided our responses in blue.

1. Regarding the role of Zi SST neurons, the author proposes that Zi SST functions as an inhibitory factor within the epileptic network, acting like a brake during seizures, though its influence is insufficient to fully suppress them. The data indeed show that with increasing intensity of electrical stimulation, the GCaMP signals of Zi SST neurons intensify. Furthermore, optogenetic activation of Zi SST neurons demonstrates a degree of suppression of epileptic activity, a conclusion with which I agree. However, I hold a different view regarding the role of the VMH. In Supplementary Figure 15, the kindling stimulation-induced activation of the Zi, VMH, SC, and CA3 regions appears nearly indistinguishable, leading to two possible interpretations: either the sampling rate of the fiber photometry recordings is insufficient to resolve the activation sequence of these brain regions, or, as I have previously speculated, the VMH receives not only inhibitory projections from the Zi but also excitatory inputs from CA3 and other sources. Therefore, the role of the VMH in facilitating epilepsy may not be solely dependent on the Zi. I suggest conducting additional experiments to further elucidate the role of the Zi SST-DMH pathway.

First, the authors have already performed experiments activating the Zi SST-DMH axons; they could add another group to inhibit the terminals of Zi SST-DMH neurons, thereby confirming whether the effects of Zi SST ablation are mediated through the DMH. Second, while ablating Zi SST neurons, chemogenetic or optogenetic inhibition of DMH glutamatergic neuron activity could be tested to determine whether this suppresses epileptic occurrences, thereby clarifying the role of the DMH in this pathway. Third, in conditions where Zi SST neurons are ablated or inhibited, leading to increased susceptibility to seizures, it would be valuable to determine whether this correlates with a significant increase in DMH activity.

Reply: We appreciate your valuable suggestion. Based on your constructive suggestions, we have added experiments in the following three aspects:

(1) The optogenetic inhibition experiment targeting the ZI^{SST}-VMH circuit has already been included in our original manuscript. Our results demonstrated that optogenetic inhibit ZI^{SST}-VMH circuit significantly amplifies seizure progression, which verifies that the inhibitory effect of ZI

SST neurons is mediated via the VMH.

(2) Accordingly, we performed additional experiments in which we injected AAV2/9-CAG-DIO-taCaspase3-TEVp virus into the ZI to ablated SST+ neurons, and AAV2/9-hEF1a-hM4D(Gi)-mCherry virus into the VMH to inhibited glutamatergic neurons. Our results showed that following ZI SST+ neuron ablation, chemogenetic inhibition of VMH glutamatergic neuron activity still significantly attenuate seizure progression (**Revised Supplementary Figure 16**). These findings support that the VMH alone plays an important role as a downstream node in the circuit.

(3) Accordingly, we inhibited ZI SST+ neurons with chemogenetics and monitored calcium activity of VMH glutamatergic neurons during the kindling-induced hippocampal seizures. In saline control group, VMH glutamatergic neurons exhibited elevated calcium activity in response to hippocampal seizures. Upon CNO-mediated chemogenetic inhibition of ZI SST+ neurons, the calcium activity of VMH glutamatergic neurons was further increased (**Revised Supplementary Figure 17**). These findings imply that the enhanced VMH glutamatergic neuronal activity during seizure is at least controlled by ZI SST+ neurons.

Taken together, we totally agree with your view that the role of the VMH in facilitating seizures may not be solely dependent on the ZI. While our paper focus on ZI-VMH circuit is contribute greatly to hippocampal seizures, VMH glutamatergic neuronal activity during seizure is partially controlled by ZI SST+ neurons. Thus, we have revised all new data in the revised manuscript and marked them in red.

Revised Figure 5a-f:

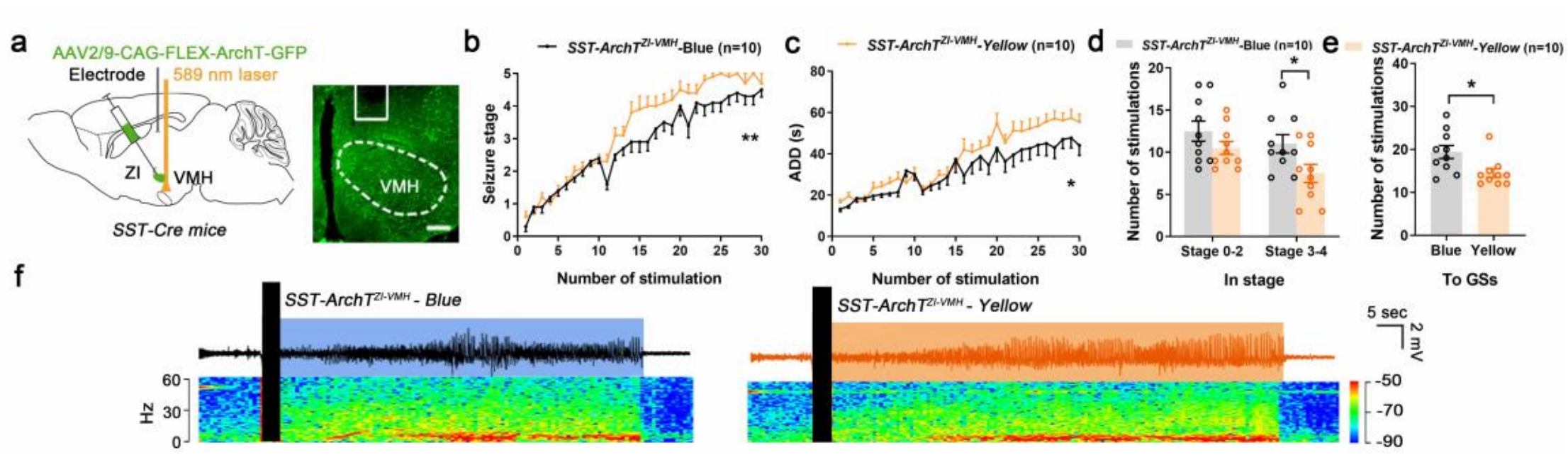

Revised Supplementary Figure 16:

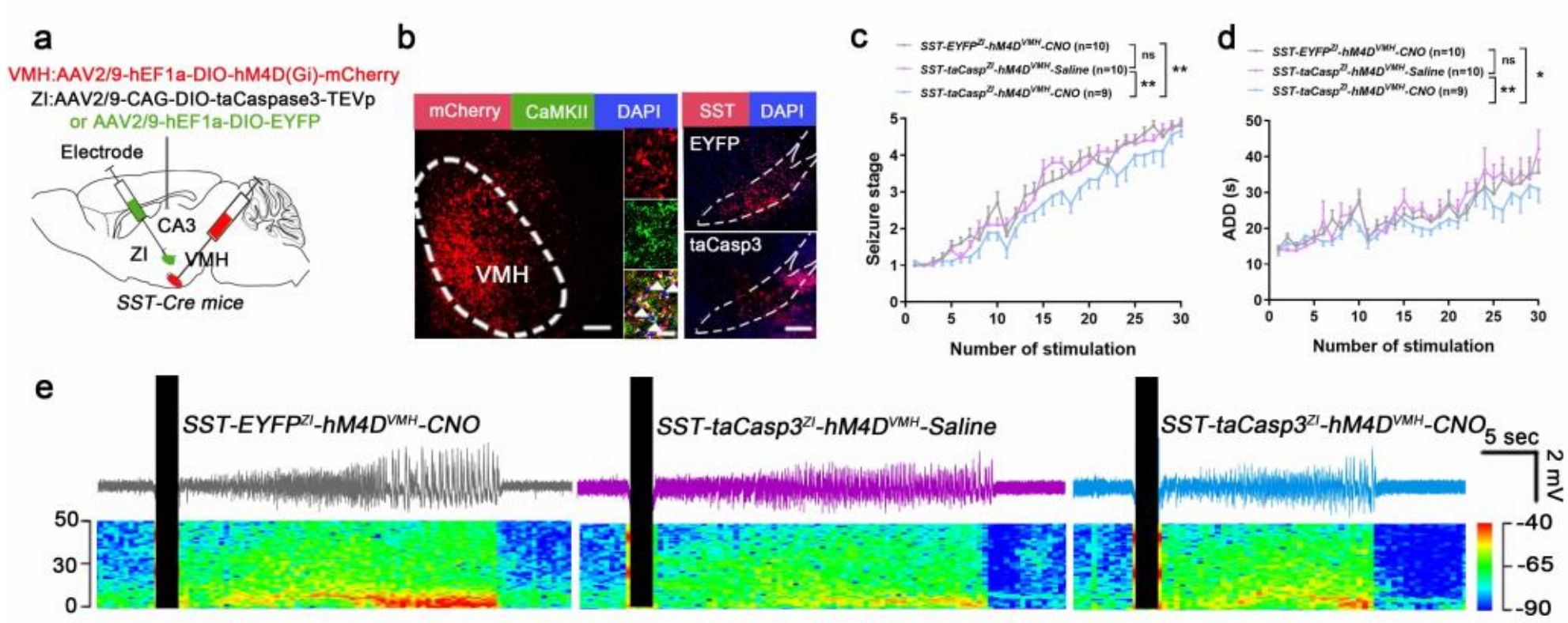

Revised Supplementary Figure 17:

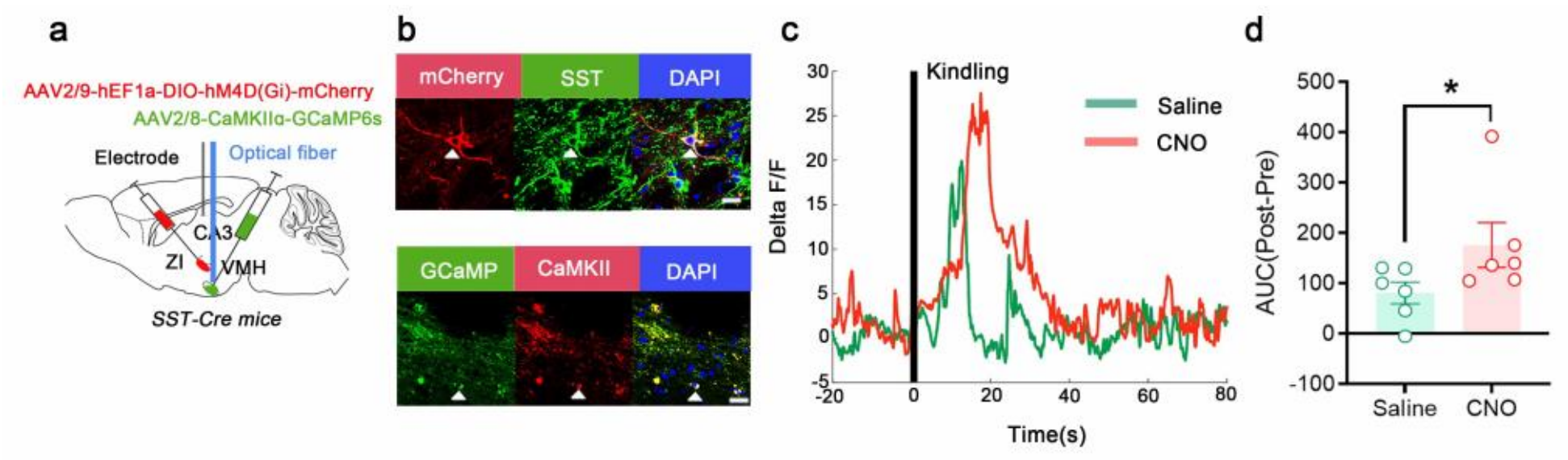

2. The research methods are generally somewhat monotonous. By regulating PV or SST+ neurons in the Zi through optogenetics, no termination of seizures was observed. Could this be related to the selected optogenetic stimulation frequency? The chosen frequency might not have effectively activated the PV+ neurons. It is necessary to conduct additional experiments to observe the effects of optogenetic stimulation at different frequencies and stimulation intensities on anti-seizure effects.

Reply: We appreciate your valuable comment. Considering that seizure inhibitory efficacy may rely on the intrinsic firing features of ZI PV^{+} and SST^{+} neurons, we have supplemented optogenetic experiments with three distinct stimulation frequencies (10 Hz, 20 Hz and 40 Hz) to explore their regulatory roles in hippocampal seizures. Our results demonstrate that optogenetic activation of PV^{+} neurons at all three frequencies significantly attenuate seizure progression, whereas its inhibitory effect against ADDs decreased in a frequency-dependent manner. This is consistent with previous study showing that PV^{+} neurons are "high-frequency oscillators" (Li et al., 2015). For SST^{+} neurons, 10 Hz and 20 Hz photo-stimulation produced obvious anti-seizure effects, while 40 Hz stimulation had no effect on hippocampal seizures (**Revised Supplementary Figure 7**). This observation aligns with recent research indicating that SST expressing ChR2 neurons exhibiting low-frequency discharge characteristics (Lee et al., 2018), cannot sustain action potential firing at 40 Hz, with spike success rates dropping to approximately 25 ± 7% at this frequency (Heldman et al., 2025). Thus, we have revised all new data in the revised manuscript and marked them in red.

Revised Supplementary Figure 7:

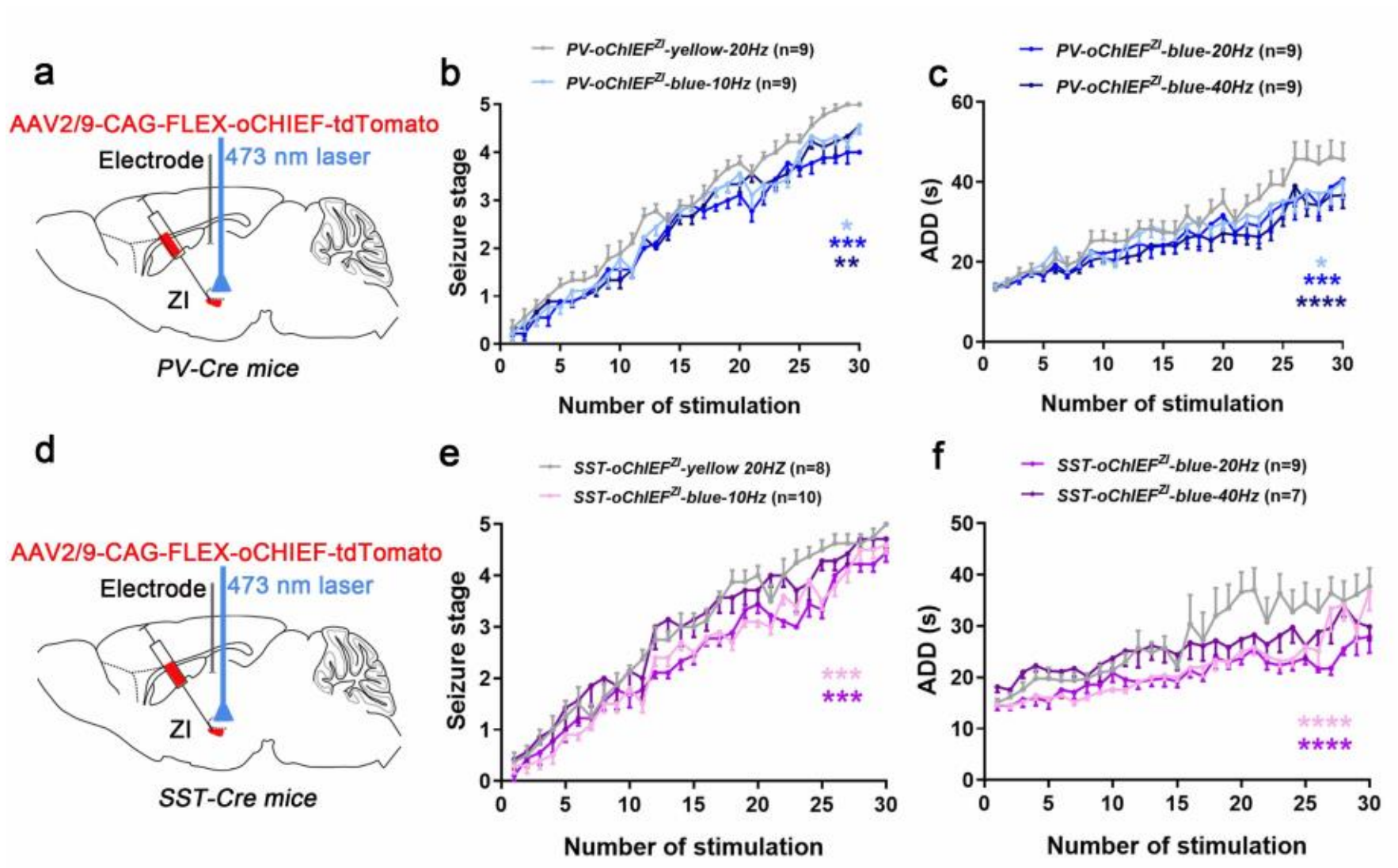

3. the role of PV+ neurons within the Zi regulating the occurrence and development of seizure seizures possible mediated the feedforward effect on SST+ neurons or other mechanisms?

Reply: We appreciate your valuable comment. Indeed, previous studies have reported that the PV+ neurons form direct monosynaptic connections with SST^+ neurons in the cortex (Pouchelon et al., 2021; Miao et al., 2016; Onorato et al., 2025). To address your interesting question, we supplemented an anatomical experiment in the revised manuscript. We performed monosynaptic retrograde rabies tracing in *SST-Cre* mice by co-injecting AAV-EF1a-DIO-EGFP-T2A-TVA, AAV-EF1a-DIO-N2cG and CVS-EnvA-ΔG-tdTomato into ZIr to map direct upstream inputs of ZIr SST^+ neurons. Our monosynaptic retrograde rabies tracing indicated that only 17.93% of ZIv PV^+ neurons form direct monosynaptic innervations onto ZIr SST^+ neurons (**Revised Supplementary Figure 20**) demonstrating that the complex local disinhibitory circuits. As acitivation of either PV or SST is anti-seizure, and this PV-SST disinhibitory circuits are not dense, we guess that anti-seizure effect of PV^+ neurons may mediate by other downstream circuits. Thus, we have revised all new data in the revised manuscript and marked them in red.

Revised Supplementary Figure 20:

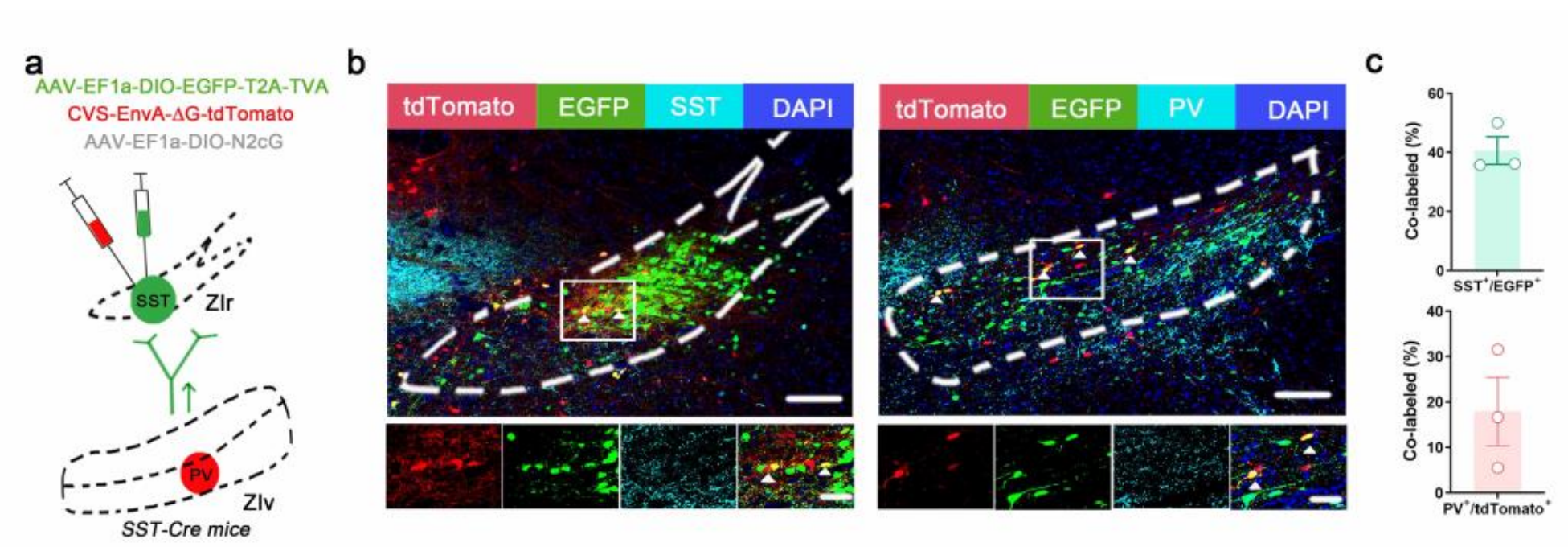

4. The author analyzed EEG power during epileptic episodes but did not present the power distribution across different EEG frequency bands.

Reply: We appreciate your valuable suggestion. We have supplemented additional analyses of EEG frequency band power in the revised manuscript. The result show that cell-type specific optogenetic activation ZI PV^+ or SST^+ neurons broadly attenuated EEG power spectra (**Revised Supplementary Fig. 6a and b**), with pronounced suppression of delta band power during GS (**Revised Supplementary Fig. 6i and j**). Correspondingly, inhibition of ZI SST^+ neurons elevated global EEG power spectra (**Revised Supplementary Fig. 6d**), alongside a pronounced rise in delta band power during both FS and GS (**Revised Supplementary Fig. 6h and l**).

Revised Supplementary Figure 6:

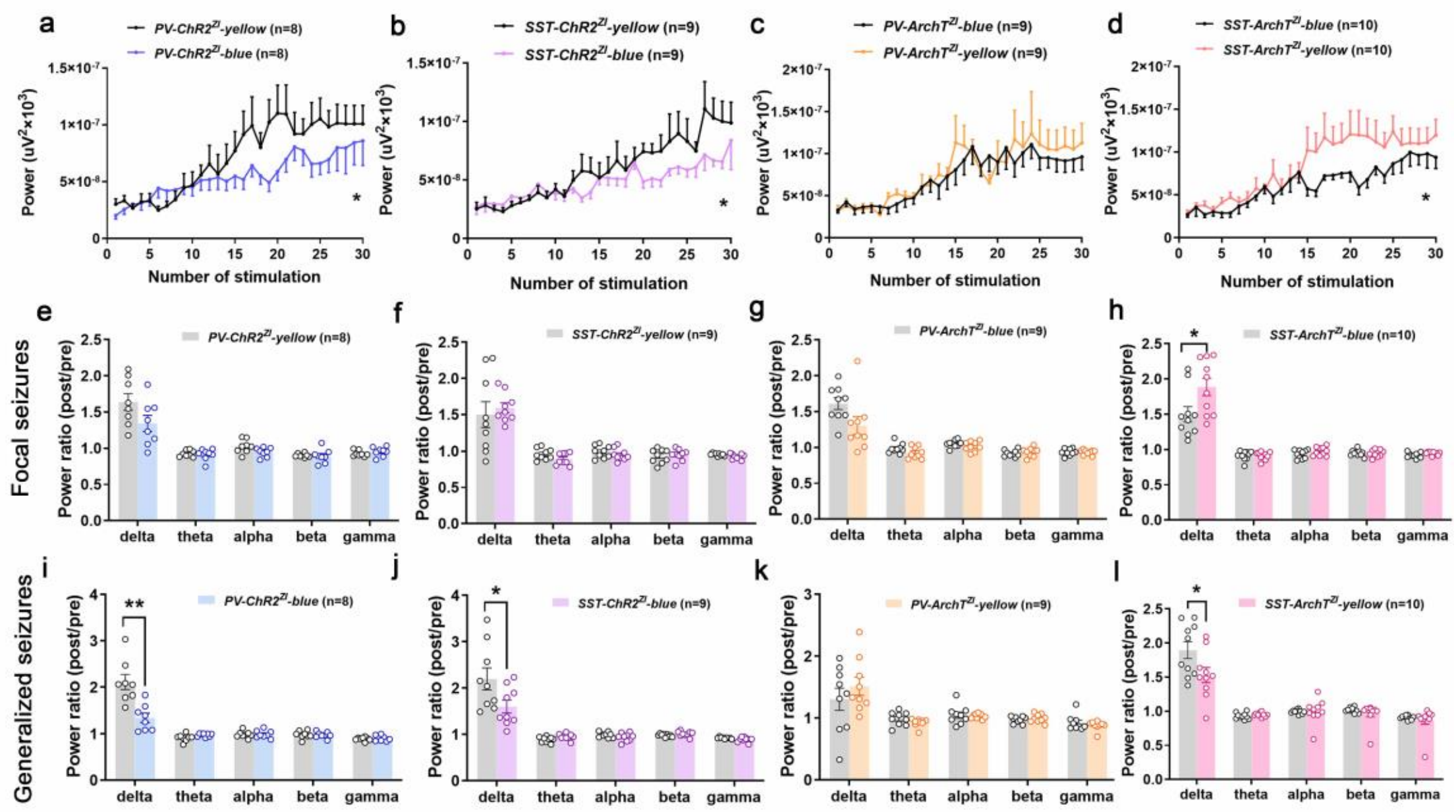

5. Regarding HPC c-fos positivity as a control, the provided data focuses on the DG Hilus area. What are the results for the CA3 region?

Reply: We appreciate your valuable comment. We have supplemented quantitative c-Fos data of the CA1 and CA3 region. The results show that the CA1 and CA3 region also exhibited a significant increase in c-Fos expression following seizures (**Revised Supplementary Figure 1b and d**).

Revised Supplementary Figure 1a-d:

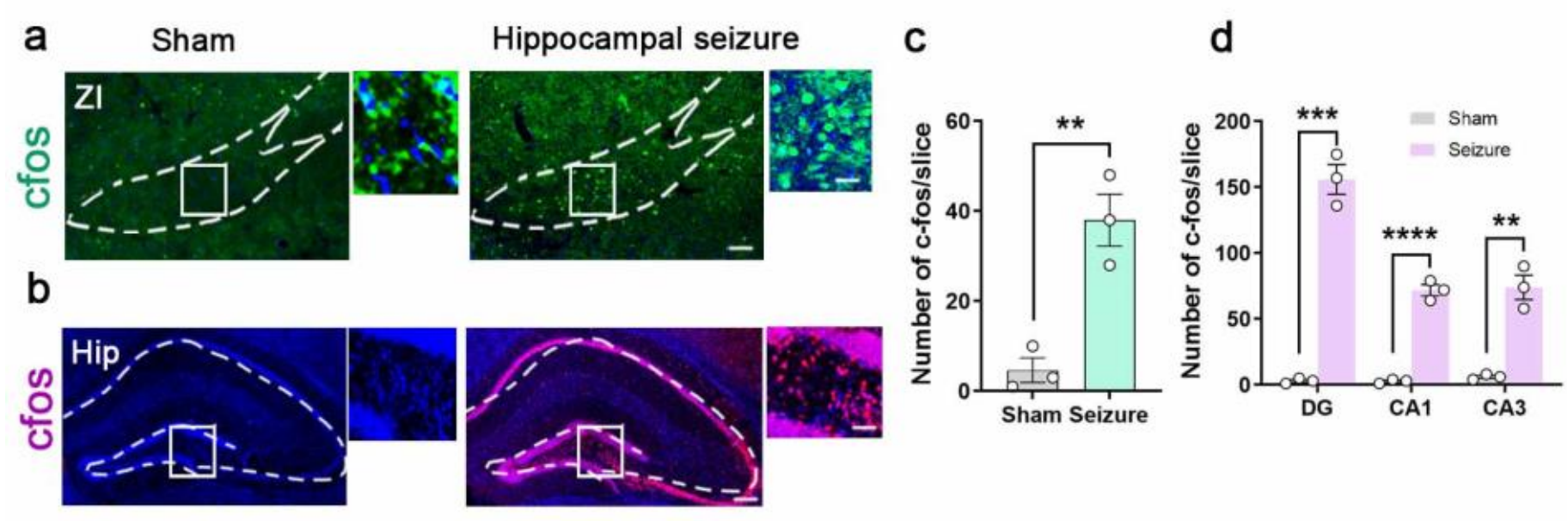

6. To further clarify the role of SST+ neurons within the Zi in regulating epileptic seizures, can additional regulatory methods, beyond optogenetic modulation, be employed to observe the impact of SST+ neuron excitability on seizure activity?

Reply: We appreciate your valuable suggestion. We performed additional chemogenetic activation experiment to further explore how the excitability of ZI SST^+ neurons modulates seizure activity. Consistent with optogenetic activation, chemogenetic activation of ZI SST^+ neurons attenuated seizure progress (**Revised Supplementary Figure 8**).

Revised Supplementary Figure 8:

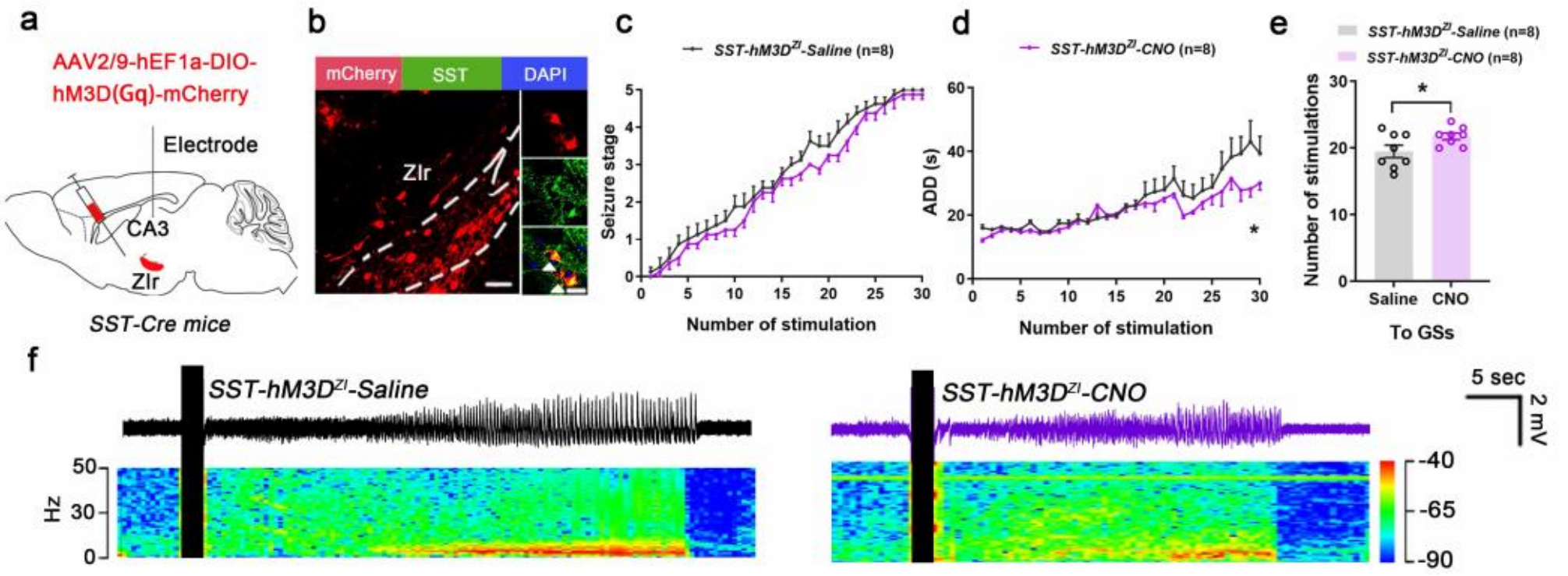
a
AAV2/9-hEF1a-DIO-
hM3D(Gq)-mCherry
Electrode
CA3
ZIr
SST-Cre mice
b
mCherry
SST
DAPI
ZIr
c
SST-hM3D^ZI-Saline (n=8)
Seizure stage
Number of stimulation
d
SST-hM3D^ZI-CNO (n=8)
ADD (s)
Number of stimulation
e
SST-hM3D^ZI-Saline (n=8)
SST-hM3D^ZI-CNO (n=8)
Number of stimulations
Saline
CNO
To GSs
f
SST-hM3D^ZI-Saline
SST-hM3D^ZI-CNO
5 sec
2 mV
Hz

Reviewer #4

While the authors have addressed my concerns, several inaccurate or incorrect statements exist in the revised manuscript which need to be corrected:

Reply: We sincerely appreciate the time and effort you have taken to review our manuscript and for the valuable comments, which have significantly contributed to the revision and improvement of our paper. In this letter, we address each of your comments individually, followed by our corresponding responses. For clarity, we have restated your comments in bold font and provided our responses in blue.

1) Lines 218-219: "suggesting that ZI SST+ GABAergic neurons (not PV+ GABAergic neurons) are required for seizure progression." This is incorrect since seizure progression is still intact, it is just enhanced. Any claims regarding requirement for seizure progression need to be revised.

Reply: Thanks. We have revised the sentence as following:

"suggesting that ZI SST^+ GABAergic neurons (not PV^+ GABAergic neurons) exert an inhibitory constraint on seizure progression, and loss of ZI SST^+ neuronal function exacerbates seizure progression."

2) Lines 232-233: "SST^+ neurons had a significantly greater impact on seizure delay or acceleration, especially during the progression phase (Supplementary Fig. 8)." This is incorrect since none of the comparisons in Supplementary Fig. 8 are significantly different based on the p values noted in the figure legend.

Reply: We sincerely thank you for pointing out this description error. We have revised the sentence as following:

"Quantitative analysis showed the two neuron types differed in their influence on seizure dynamics (Supplementary Fig. 10)."

3) Line 271: As noted in my earlier review, the authors cannot conclude that all the downstream circuits were activated since only VMH during FS and GS and AHN during GS show significant elevated calcium signals. In addition, the comparison noted in the text, about calcium signal being gradually elevated from FS to GS, is not actually shown by the data in Fig. 4 which compares FS and GS separately.

Reply: Thank you very much for these important comments. We softened the original conclusion and explicitly state that the ZI^{SST}-VMH circuit exhibits increased calcium activity during FS and GS, while the ZI^{SST}-AHN circuit shows such changes solely in GS. In addition, we have removed the description of the gradual increase of calcium signals from the FS to the GS. We have revised the sentence as following:

"The results showed that the calcium signal of the ZI^{SST}-VMH circuit was activated during both FS and GS, whereas the ZI^{SST}-AHN circuit was activated during GS (Fig. 4g-m). "

4) Line 314-315: The statement that "ZI SST+ neurons projecting to the VMH, but not to the AHN...contributes to seizure progression" is incorrect since inhibition of the ZI SST+-AHN circuit did lead to changes in seizure progression (just opposite of what was seen with ZI SST+-VMH circuit)

Reply: We sincerely thank you for pointing out this description error. We have revised the statement as following:
"These anatomical differences likely serve as the structural basis for the opposing functional roles of the two circuits".

5) Line 334: VGLUT1 should be VGLUT2.
Reply: Thank you for pointing this careless error. We have revised "VGLUT1" into "VGLUT2" in the revised results section.

6) While some parts are improved, the text is still difficult to read due to grammatical errors throughout the manuscript. Professional copyediting is recommended.
Reply: Thanks for your suggestion. We have fully revised the manuscript, corrected all grammatical errors, and conducted professional copyediting to greatly improve the readability of the text. All the revision are marked in red in the revised manuscript.

Reference

Li, L.Y. et al. Differential Receptive Field Properties of Parvalbumin and Somatostatin Inhibitory Neurons in Mouse Auditory Cortex. *Cereb Cortex* **27**, 1782-1791 (2015)

Heldman, R., Pang, D., Zhao, X., Mensh, B. & Wang, Y. Time or distance encoding by hippocampal neurons with heterogenous ramping rates. *BioRxiv (Preprint)* (2025)

Lee, B., Shin, D., Gross, S.P. & Cho, K. H. Combined Positive and Negative Feedback Allows Modulation of Neuronal Oscillation Frequency during Sensory Processing. *Cell Rep* **25**, 1548-1560 e3 (2018)

Pouchelon, G. et al. The organization and development of cortical interneuron presynaptic circuits are area specific. *Cell Rep* **37**, 109993 (2021)

Miao, Q. et al. Selective Maturation of Temporal Dynamics of Intracortical Excitatory Transmission at the Critical Period Onset. *Cell Rep* **16**, 1677-1689 (2016)

Onorato, I. et al. Distinct roles of PV and Sst interneurons in visually induced gamma oscillations. *Cell Rep* **44**, 115385 (2025)

Reviewer #2

We sincerely appreciate the time and effort you have taken to review our manuscript and for the valuable comments, which have significantly contributed to the revision and improvement of our paper. In this letter, we address each of your comments individually, followed by our corresponding responses. For clarity, we have restated your comments in bold font and provided our responses in blue. All revisions are highlighted in red in the revised version.

1. The manuscript title differs between the main text and the Supplementary Materials. In addition, the main-text title does not clearly state the key finding and should be revised.

Reply: We appreciate your valuable suggestion. We have unified the manuscript title throughout the main text and supplementary materials. Furthermore, we have revised the title to clearly present our key findings and short-term observations, and relevant modifications have been marked in the revised manuscript. The revised manuscript title is presented below:

The main-text title: Zona Incerta somatostatin^{+} neurons modulate seizure progression through hypothalamic circuits

Page 6, lines 151-152: Frequency-dependent anti-seizure effects of ZI PV^{+} and SST^{+} GABAergic neurons

Page 9, lines 248-249: The ZI^{SST}-VMH projections mediate the role of ZI SST^{+} GABAergic neurons in seizure progression

Page 12, lines 327-328: ZI SST^{+} neurons mediate anti-seizure effects through inhibition of downstream VHM glutamatergic populations

Supplementary Figure 1: ZI GABAergic neurons are gradually activated during seizure progression.

Supplementary Figure 6: Optogenetic regulation of ZI PV^{+} and SST^{+} GABAergic neurons alters EEG power spectrum and delta band.

Supplementary Figure 7: Optogenetic activation of ZI PV^{+} or SST^{+} GABAergic neurons exerts divergent frequency-dependent seizure-suppressive effects.

Supplementary Figure 9. Ablation of ZI SST^{+} but not PV^{+} GABAergic neurons amplifies seizure progression.

Supplementary Figure 13: Optogenetic activation of the ZI^{SST}-VMH circuit inhibits seizures in an SSTR-dependent manner.

2. In the revised manuscript, the changes prompted by reviewer comments could be more clearly indicated, and it would help if their locations were noted in the response letter. This is especially important for substantive revisions. For example, the authors wrote "Taken together, we totally agree with your view that the role of the VMH in facilitating seizures may not be solely dependent on the ZI," but it is not entirely clear to me where this discussion was actually added to the manuscript. Similarly, regarding Reviewer 4's concerns about Lines 218 – 219 and Lines 232 – 233, the authors appear to have revised the conclusion statements themselves, though I may have overlooked a corresponding update in the Discussion. I would suggest that for all key revisions, the exact location in the manuscript be stated explicitly in the response letter.

Reply: We appreciate your thoughtful comment and apologize for the unclear marking of revision locations before. All key revisions, including newly-added discussion and rewritten conclusions,

are now annotated with accurate page and line numbers in our reply letter for straightforward verification.

Page 20, lines 559-563: Notably, we speculate that the VMH may not be exclusively under inhibitory control of ZI SST$^+$ neurons; it may also receive strong direct excitatory innervation from the hippocampal CA3 and other epileptogenic regions. This could explain the nearly synchronous activation of ZI, VMH, SC and CA3 in kindling models (Supplementary Fig. 19).

Page 9, lines 226-228: suggesting that ZI SST$^+$ GABAergic neurons (not PV$^+$ GABAergic neurons) exert an inhibitory constraint on seizure progression, and loss of ZI SST$^+$ neuronal function exacerbates seizure progression.

Page 9, lines 241-243: Quantitative analysis showed the two neuron types differed in their influence on seizure dynamics (Supplementary Fig. 10).

3. Supplementary Figure 1d shows data for DG, CA1, and CA3, but the magnified insets currently only display DG. It would be helpful to also include representative images for CA1 and CA3.

Reply: We appreciate your valuable comment. We have supplemented magnified representative immunohistochemical images of CA1 and CA3 in revised Supplementary Figure 1b.

Revised Supplementary Figure 1:

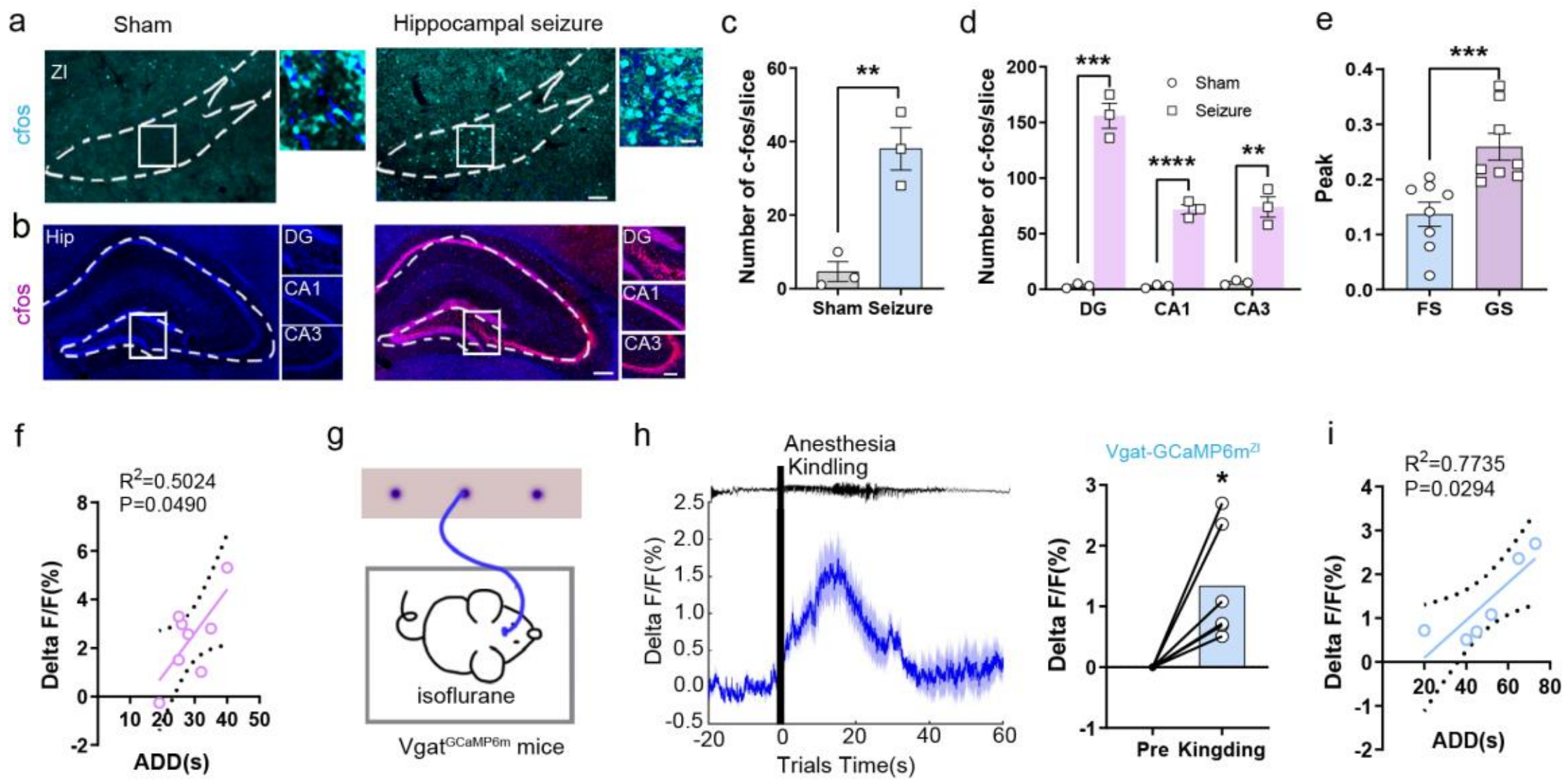

4. I am curious about one point: optogenetic manipulation of ZI PV+ or SST+ neurons mainly shifts the lower-frequency EEG components (delta band) without affecting gamma oscillations. Gamma activity is classically tied to PV+ interneuron function, so this dissociation is unexpected and worth noting. If the authors feel it is appropriate, a brief discussion of this point in the Discussion would be helpful.

Reply: We appreciate your valuable suggestion. Indeed, GABAergic neurons are classically tied to gamma activity in many other regions (Sohal et al., 2009; Onorato et al., 2025), ZI GABAergic neurons are tightly coupled to circuits mediating low-frequency delta oscillations. Consistent with this notion, our previous work demonstrated that low-frequency electrical stimulation (1 Hz and 5 Hz) of the ZI alleviates epileptic seizures by activating local GABAergic neurons (Zhang et al.,

2024). In addition, activation of ventral ZI Lhx6$^+$ GABAergic neurons-which largely overlap with the SST$^+$ subtype-promotes NREM slow-wave sleep, a physiological state primarily characterized by delta-band oscillations (0.5-4 Hz) (Veit et al., 2017). GABAergic activation modulates the overall excitability of slow-rhythm circuits, thereby directly suppressing delta oscillatory activity. Therefore, optogenetic activation of ZI PV$^+$ and SST$^+$ neurons preferentially alters delta-band EEG signals by exerting robust inhibitory control over downstream slow-rhythm networks, including thalamocortical circuits that generate slow-wave delta oscillations (Veit et al., 2017). These findings suggest that GABAergic neuron-dependent rhythmic regulation exhibits brain-region specificity. Thus, we have the discussion section in revised manuscript and marked them in red.

Page 17, lines 472-486: Of note, the frequency-dependent anti-seizure profiles of ZI PV$^+$ and SST$^+$ neurons can be partially interpreted from their inherent coupling to low-frequency oscillatory networks. Our previous work demonstrated that low-frequency electrical stimulation (1 Hz and 5 Hz) of the ZI alleviates seizures by activating local GABAergic neurons[23]. In addition, activation of ventral ZI Lhx6$^+$ GABAergic neurons-which largely overlap with the SST+ subtype-promotes NREM slow-wave sleep, a physiological state primarily characterized by delta-band oscillations (0.5-4 Hz)[43]. GABAergic activation modulates the overall excitability of slow-rhythm circuits, thereby directly suppressing delta oscillatory activity. Therefore, optogenetic activation of ZI PV$^+$ and SST$^+$ neurons preferentially alter delta-band EEG signals by exerting robust inhibitory control over downstream slow-rhythm networks, including thalamocortical circuits that generate slow-wave delta oscillations[43]. Although GABAergic neurons are classically tied to gamma activity in many other regions[44,45], our findings suggest that GABAergic neuron-dependent rhythmic regulation exhibits brain-region specificity.

Reference

Sohal, V.S. et al. Parvalbumin neurons and gamma rhythms enhance cortical circuit performance. *Nature* **459**, 698–702 (2009)

Onorato, I. et al. Distinct roles of PV and Sst interneurons in visually induced gamma oscillations. *Cell Rep* **44**, 115385 (2025)

Distinct roles of PV and Sst interneurons in visually induced gamma oscillatio

Veit, J., et al. Cortical gamma band synchronization through somatostatin interneurons. *Nat Neurosci* **20**, 951959 (2017)

Zhang, M. et al. Low-frequency stimulation in the zona incerta attenuates seizure via driving gabaergic neuronal activity. *Neurobiol Dis* **192**, 106424 (2024)
