# Supplementary material for: Zona incerta somatostatin+ neurons modulate seizure progression through hypothalamic circuits
Source: Nat Commun. 2026 Sep 29;17:11234. doi: 10.1038/s41467-026-78174-9 (PMC13720055; doi:10.1038/s41467-026-78174-9)
Supplement: Supplementary file 5 [file 41467_2026_78174_MOESM3_ESM.docx]

**Supplementary Data 1: The exact statistic parameter for all statistically significant comparisons.**

| **Figure & Penal** | **Statistical test** | **Groups** | **Statistic parameter** |
| --- | --- | --- | --- |
| Figure 1c | Two-tailed paired student's t test | Pre vs FS | t(7)=4.053, P=0.0049, R^2^=0.7012 , 95% CI = [-3.798, 0.9993] |
| Figure 1d | Two-tailed paired student's t test | Pre vs GS | t(7)=13.71, P<0.0001, R^2^=0.9641, 95% CI = [-9.482, -6.693] |
| Figure 1h | Two-way ANOVA for repeated measures | Yellow vs Blue | F(29,406)=1.622, P=0.0237, ƞ2 p=0.104, 95% CI = [-0.1904, -0.8596] |
| Figure 1i | Two-way ANOVA for repeated measures | Yellow vs Blue | F(29,406)=1.681, P=0.0163, ƞ2 p=0.107, 95% CI = [-0.3281, 10.01] |
| Figure 1j | Two-way ANOVA with post-hoc Bonferroni test | Yellow vs Blue | Stage 0-2: P=0.1999;  Stage 3-4: P=0.0448; |
| Figure 1k | Two-tailed Unpaired student's t test | Yellow vs Blue | t(14)=2.820, P=0.0136, R^2^=0.3622 , 95% CI = [-8.583, -1.167] |
| Figure 1o | Two-way ANOVA for repeated measures | Blue vs Yellow | F(29,522)=1.794, P=0.0073, ƞ2 p=0.091, 95% CI = [-0.8752, -0.2248] |
| Figure 1p | Two-way ANOVA for repeated measures | Blue vs Yellow | F(29,522)=1.507, P=0.0451, ƞ2 p=0.077, 95% CI = [-16.69, -0.1549] |
| Figure 1q | Two-way ANOVA with post-hoc Bonferroni test | Blue vs Yellow | Stage 0-2: P=0.4837;  Stage 3-4: P=0.0183; |
| Figure 1r | Two-tailed Unpaired student's t test | Blue vs Yellow | t(18)=3.825, P=0.0012, R^2^=0.4484, 95% CI = [2,749, 9.451] |
| Figure 2c | Two-tailed paired student's t test | Pre vs FS | t(7)=2.930, P=0.022, R^2^=0.5509, 95% CI = [-1.454, -0.1553] |
| Figure 2d | Two-tailed paired student's t test | Pre vs GS | t(7)=3.891, P=0.0060, R^2^=0.6838 , 95% CI = [-4.021, -0.9809] |
| Figure 2g | Two-way ANOVA for repeated measures | Yellow vs Blue | F(29,406)=2.791, P<0.0001, ƞ2 p=0.166, 95% CI = [0.4288, 1.230] |
| Figure 2h | Two-way ANOVA for repeated measures | Yellow vs Blue | F(29,406)=2.461, P<0.0001, ƞ2 p=0.150, 95% CI = [3.549,19.01] |
| Figure 2i | Two-way ANOVA with post-hoc Bonferroni test | Yellow vs Blue | Stage 0-2: P=0.0348, 95% CI = [-8.716,-0.2841]  Stage 3-4: P=0.0409, 95% CI = [-8.591,-0.1591] |
| Figure 2j | Two-tailed Mann-Whitney U test | Yellow vs Blue | U=7, P=0.0053 |
| Figure 2m | Two-tailed paired student's t test | Pre vs FS | t(7)=5.690, P=0.0007, R^2^=0.8222, 95% CI = [-0.4216, -0.1741] |
| Figure 2n | Two-tailed paired student's t test | Pre vs GS | t(7)=7.699, P=0.0001, R^2^=0.6838, 95% CI = [-4.495, -2.383] |
| Figure 2q | Two-way ANOVA for repeated measures | Yellow vs Blue | F(29,464)=1.759, P=0.0095, ƞ2 p=0.099, 95% CI = [0.1501, 1.020] |
| Figure 2r | Two-way ANOVA for repeated measures | Yellow vs Blue | F(29,464)=1.535, P=0.0389, ƞ2 p=0.088, 95% CI = [0.4578, 11.19] |
| Figure 2s | Two-way ANOVA with post-hoc Bonferroni test | Yellow vs Blue | Stage 0-2: P=0.6632, 95% CI = [-6.395, 2.617]  Stage 3-4: P=0.0471, 95% CI = [-9.062, -0.04929] |
| Figure 2t | Two-tailed Mann-Whitney U test | Yellow vs Blue | U=17, P=0.0367 |
| Figure 3c | Two-way ANOVA for repeated measures | Blue vs Yellow | F(29,464)=0.6877, P=0.8901, ƞ2 p=0.041, 95% CI = [-0.4807, 0.4288] |
| Figure 3d | Two-way ANOVA for repeated measures | Blue vs Yellow | F(29,464)=0.8158, P=0.7417, ƞ2 p=0.049, 95% CI = [-9.921, 3.132] |
| Figure 3e | Two-way ANOVA with post-hoc Bonferroni test | Blue vs Yellow | Stage 0-2: P>0.9999, 95% CI = [-3.800, 3.133]  Stage 3-4: P=0.7449,95% CI = [-2.133, 4.800] |
| Figure 3f | Two-tailed unpaired student's t test | Blue vs Yellow | t(16)=0.1204, P=0.9057, R^2^=0.000905, 95% CI = [-6.203, 5.536] |
| Figure 3j | Two-way ANOVA for repeated measures | Blue vs Yellow | F(29,522)=1.720, P=0.0119, ƞ2 p=0.087, 95% CI = [-0.9548, 0.1082] |
| Figure 3k | Two-way ANOVA for repeated measures | Blue vs Yellow | F(29,522)=1.781, P=0.0080, ƞ2 p=0.090, 95% CI = [-14.48, -0.9438] |
| Figure 3l | Two-way ANOVA with post-hoc Bonferroni test | Blue vs Yellow | Stage 0-2: P=0.6365  Stage 3-4: P=0.0453 |
| Figure 3m | Two-tailed unpaired student's t test | Blue vs Yellow | t(18)=2.121, P=0.0480, R^2^=0.2000, 95% CI = [0.05385, 11.15] |
| Figure 4d | Two-way ANOVA with post-hoc Bonferroni test | Yellow vs Blue | Hip: P=0.0480; AV: P>0.9999; VL: P>0.9999; Rt: P>0.9999; AHN: P=0.0002; VMH: P<0.0001; LD: P>0.9999; Po: P>0.9999; LHb: P=0.2801; PC: P>0.9999; CL: P>0.9999; PF: P>0.9999; MD: P>0.9999; PH: P>0.9999; LH: P=0.3458; PAG: P>0.9999; VM: P>0.9999 |
| Figure 4g | Two-tailed paired student's t test | Pre vs FS | t(7)=3.953, P=0.0055, R^2^=0.6907, 95% CI = [-0.3134, -0.07880] |
| Figure 4h | Two-tailed paired student's t test | Pre vs GS | t(7)=4.705, P=0.0022, R^2^=0.7598, 95% CI = [-0.9781, -0.3238] |
| Figure 4i | Two-tailed paired student's t test | Pre vs FS | t(7)=3.953, P=0.1099, R^2^=0.1471, 95% CI = [-1.289, 0.4712] |
| Figure 4j | Two-tailed paired student's t test | Pre vs GS | t(7)=3.101, P=0.0173, R^2^=0.5788, 95% CI = [-4.806, -0.6478] |
| Figure 4k | Two-tailed paired student's t test | Pre vs FS | t(7)=3.953, P=1.918, R^2^=0.3445, 95% CI = [-2.350, 0.2450] |
| Figure 4l | Two-tailed paired student's t test | Pre vs GS | t(7)=1.538, P=0.1679, R^2^=0.2526, 95% CI = [-10.65, 2.255] |
| Figure 5b | Two-way ANOVA for repeated measures | Blue vs Yellow | F(29,522)=1.903, P=0.0034, ƞ2 p=0.096, 95% CI = [-0.8548, -0.07182] |
| Figure 5c | Two-way ANOVA for repeated measures | Blue vs Yellow | F(29,522)=1.501, P=0.0467, ƞ2 p=0.077, 95% CI = [-13.03, 1.627] |
| Figure 5d | Two-way ANOVA with post-hoc Bonferroni test | Blue vs Yellow | Stage 0-2: P=0.3670, 95% CI = [-1.450, 5.450]  Stage 3-4: P=0.0462, 95% CI = [0.05038, 6.950] |
| Figure 5e | Two-tailed Mann-Whitney U test | Blue vs Yellow | U=17, P=0.0102 |
| Figure 5h | Two-way ANOVA for repeated measures | Blue vs Yellow | F(29,464)=1.518, P=0.0430, ƞ2 p=0.091, 95% CI = [-0.07270, 0.9838] |
| Figure 5i | Two-way ANOVA for repeated measures | Blue vs Yellow | F(29,464)=1.600, P=0.0263, ƞ2 p=0.091, 95% CI = [0.09224, 13.56] |
| Figure 5j | Two-way ANOVA with post-hoc Bonferroni test | Blue vs Yellow | Stage 0-2: P=0.6366;  Stage 3-4: P=0.2519 |
| Figure 5k | Two-tailed Mann-Whitney U test | Blue vs Yellow | U=17, P=0.0371 |
| Figure 5n | Two-way ANOVA for repeated measures | Blue vs Yellow | F(29,406)=0.6824, P=0.8942, ƞ2 p=0.046, 95% CI = [-0.5611, 0.4194] |
| Figure 5o | Two-way ANOVA for repeated measures | Blue vs Yellow | F(29,406)=0.8013, P=0.7607, ƞ2 p=0.054, 95% CI = [-8.381, 5.856] |
| Figure 5p | Two-way ANOVA with post-hoc Bonferroni test | Blue vs Yellow | Stage 0-2: P=0.7308, 95% CI = [-2.164, 4.914]  Stage 3-4: P=0.3831, 95% CI = [-5.539, 1.539] |
| Figure 5q | Two-tailed unpaired student's t test | Blue vs Yellow | t(14)=0.7179, P=0.4846, R^2^=0.03551, 95% CI = [-6.978, 3.478] |
| Figure 6d | Two-tailed paired student's t test | Pre vs Activation | t(5)=5.461, P=0.0028, R^2^=0.8564, 95% CI = [0.7789, 2.164] |
| Figure 6g | Two-way ANOVA with post-hoc Tukey's test | mCherry-CNO vs hM3D-saline | P=0.4012, 95% CI = [-0.4231, 0.1231] |
|  |  | mCherry-CNO vs hM3D-CNO | P<0.0001, 95% CI = [-0.9483, -0.3850] |
|  |  | hM3D-saline vs hM3D-CNO | P<0.0001, 95% CI = [-0.7840, -0.2494] |
| Figure 6h | Two-way ANOVA with post-hoc Tukey's test | mCherry-CNO vs hM3D-saline | P=0.429, 95% CI = [-3.452, 1.065] |
|  |  | mCherry-CNO vs hM3D-CNO | P<0.0001, 95% CI = [-7.349, -2.531] |
|  |  | hM3D-saline vs hM3D-CNO | P=0.0002, 95% CI = [-5.953, -1.540] |
| Figure 6i | Two-way ANOVA with post-hoc Tukey's test | mCherry-CNO vs hM3D-saline | P=0.8501, 95% CI = [-0.1972, 0.3157] |
|  |  | mCherry-CNO vs hM3D-CNO | P=0.0091, 95% CI = [-0.6382, -0.07290] |
|  |  | hM3D-saline vs hM3D-CNO | P=0.0007, 95% CI = [-0.6796, -0.1500] |
| Figure 6j | Two-way ANOVA with post-hoc Tukey's test | mCherry-CNO vs hM3D-saline | P=0.2629, 95% CI = [-3.812, 0.7675] |
|  |  | mCherry-CNO vs hM3D-CNO | P<0.0001, 95% CI = [-8.255, -2.871] |
|  |  | hM3D-saline vs hM3D-CNO | P=0.0012, 95% CI = [-6.708, -1.374] |
| Supplementary Figure 1c | Two-tailed unpaired student's t test | Sham vs Seizure | t(4)=5.220, P=0.0064, R^2^=0.8720, 95% CI = [-51.06, -15.60] |
| Supplementary Figure 1d | Two-tailed unpaired student's t test | Sham vs Seizure | DG: t(4)=13.51, P=0.0002, R^2^=0.9785, 95% CI = [-184.5, -121.5];  CA1: t(4)=15.6, P<0.0001, R^2^=0.9838, 95% CI = [-81.28, -56.72];  CA3: t(4)=7.304, P=0.0019, R^2^=0.9303, 95% CI = [-93.85, -42.15] |
| Supplementary Figure 1e | Two-tailed Mann-Whitney U test | FS vs GS | U=1, P=0.0003 |
| Supplementary Figure 1f | Pearson test |  | P=0.0490, R^2^=0.5024, 95% CI = [0.001042, 0.3570] |
| Supplementary Figure 1h | Two-tailed paired student's t test | Pre vs Kindling | t(6)=3.487, P=0.0175, R^2^=0.7086, 95% CI = [-2.331, -0.3526] |
| Supplementary Figure 1i | Pearson test |  | P=0.0294, R^2^=0.7335, 95% CI = [0.006983, 0.07863] |
| Supplementary Figure 2b | Two-way ANOVA with Tukey's multiple comparison test | Yellow vs Blue (n=5) | P<0.0001, 95% CI = [0.3235, 0.9582] |
|  |  | Yellow vs Blue (n=3) | P=0.1863, 95% CI = [-0.1141, 0.7779] |
| Supplementary Figure 2c | Two-way ANOVA with Tukey's multiple comparison test | Yellow vs Blue (n=5) | P<0.0001, 95% CI = [3.666, 8.372] |
|  |  | Yellow vs Blue (n=3) | P=0.1049, 95% CI = [-0.4510, 6.209] |
| Supplementary Figure 3b | Two-tailed unpaired student's t test or Two-tailed Mann-Whitney U test | Sham vs Kindling | ZIr: t(10)=6.192, P=0.0001, R^2^=0.7931, 95% CI = [-11.33, -5.335];  ZIv: U=0, P=0.0022 |
| Supplementary Figure 3e | Two-tailed Mann-Whitney U test | Sham vs Kindling | ZIr: U=0, P=0.0022;  ZIv: U=0.500, P=0.0043; |
| Supplementary Figure 3h | Two-tailed Mann-Whitney U test | Sham vs Kindling | ZIr: U=7.5, P=0.1061;  ZIv: U=15, P>0.9999 |
| Supplementary Figure 3k | Two-tailed Mann-Whitney U test | Sham vs Kindling | ZIr: U=1, P=0.0043;  ZIv: U=11.5, P=0.4242 |
| Supplementary Figure 4a | Two-tailed unpaired student's t test | PV vs SST | t(14)=1.734, P=0.1049, R^2^=0.1768, 95% CI = [-14.82, 1.569] |
| Supplementary Figure 4b | Two-tailed Mann-Whitney U test | PV vs SST | U=18, P=0.1605 |
| Supplementary Figure 4c | Pearson test |  | P<0.0001, R^2^=0.7606, 95% CI = [0.6631, 0.9550] |
| Supplementary Figure 4d | Pearson test |  | P<0.0001, R^2^=0.8284, 95% CI = [0.08451, 0.1442] |
| Supplementary Figure 4h | Ordinary one-way ANOVA with Tukey's multiple comparison test | 100 µA vs 200 µA | P=0.6756, 95% CI = [-8.702, 4.391] |
|  |  | 100 µA vs 400 µA | P<0.0001, 95% CI = [-24.73, -11.64] |
|  |  | 200 µA vs 400 µA | P<0.0001, 95% CI = [-22.58, -9.487] |
| Supplementary Figure 6a | Two-way ANOVA for repeated measures | Yellow vs Blue | F(29,406)=1.559, P=0.0346, ƞ2 p=0.091, 95% CI = [-1.075e-008, 4.276e-008] |
| Supplementary Figure 6b | Two-way ANOVA for repeated measures | Yellow vs Blue | F(29,464)=1.523, P=0.0417, ƞ2 p=0.087, 95% CI = [-7.947e-009, 2.962e-008] |
| Supplementary Figure 6c | Two-way ANOVA for repeated measures | Blue vs Yellow | F(29,464)=0.4581, P=0.9937, ƞ2 p=0.032, 95% CI = [-3.621e-008, 2.735e-008] |
| Supplementary Figure 6d | Two-way ANOVA for repeated measures | Blue vs Yellow | F(29,522)=1.531, P=0.0393, ƞ2 p=0.078, 95% CI = [-4.836e-008, 6.516e-009] |
| Supplementary Figure 6e | Two-tailed unpaired student's t test | Yellow vs Blue | delta: t(14)=1.851, P=0.0854, R^2^=0.1966, 95% CI = [-0.04712, 0.6402];  theta: t(14)=0.3679, P=0.7185, R^2^=0.009573, 95% CI = [-0.05677, 0.08027];  alpha: t(14)=1.957, P=0.0707, R^2^=0.2147, 95% CI = [-0.007538, 0.1642];  beta: t(14)=0.2774, P=0.7855, R^2^=0.005465, 95% CI = [-0.06574, 0.08527];  gamma: t(14)=1.005, P=0.3319, R^2^=0.06731, 95% CI = [-0.08560, 0.03097] |
| Supplementary Figure 6f | Two-tailed unpaired student's t test | Yellow vs Blue | delta: t(16)=0.4927, P=0.6289, R^2^=0.01494, 95% CI = [-0.4996, 0.3112];  theta: t(16)=1.810, P=0.0891, R^2^=0.1700, 95% CI = [-0.01154, 0.1464];  alpha: t(16)=0.9968, P=0.3337, R^2^=0.05847, 95% CI = [-0.04404, 0.1222];  beta: t(16)=0.4620, P=0.6503, R^2^=0.01316, 95% CI = [-0.1073, 0.06892];  gamma: t(16)=2.011, P=0.0614, R^2^=0.2018, 95% CI = [-0.001536, 0.05850] |
| Supplementary Figure 6g | Two-tailed unpaired student's t test | Blue vs Yellow | delta: t(16)=2.052, P=0.0569, R^2^=0.02083, 95% CI = [-0.01020, 0.6246];  theta: t(16)=1.730, P=0.1029, R^2^=0.1576, 95% CI = [-0.01371, 0.1354];  alpha: t(16)=1.259, P=0.09014, R^2^=0.05847, 95% CI = [-0.02455, 0.09635];  beta: t(16)=0.7283, P=0.4770, R^2^ = 0.03208, 95% CI = [-0.07419, 0.03625];  gamma: t(16)=0.03202, P=0.9749, R^2^ = 6.406e-005, 95% CI = [-0.04145, 0.04272] |
| Supplementary Figure 6h | Two-tailed unpaired student's t test or Mann Whitney U test | Blue vs Yellow | delta: Mann-Whitney U=21, P=0.0288;  theta: t(18)=0.02612, P=0.9794, R^2^=3.791e-005, 95% CI = [-0.05685, 0.05828];  alpha: t(18)=1.525, P=0.1445, R^2^=0.1145, 95% CI = [-0.09902, 0.01571];  beta: t(18)=0.1116, P=0.9124, R^2^=0.0006912, 95% CI = [-0.04211, 0.04683];  gamma: Mann-Whitney U=28, P=0.1051 |
| Supplementary Figure 6i | Two-tailed unpaired student's t test or Mann Whitney U test | Yellow vs Blue | delta: t(14)=4.015, P=0.0013; R^2^=0.5352, 95% CI = [0.3556, 1.171]  theta: Mann-Whitney U=14, P=0.650;  alpha: t(14)=0.1430, P=0.8883, R^2^=0.001459, 95% CI = [-0.08901, 0.07789];  beta: t(14)=0.08376, P=0.9344, R^2^=0.0005008, 95% CI = [-0.07662, 0.08284];  gamma: t(14)=0.6433, P=0.5304, R^2^=0.02871, 95% CI = [-0.07383, 0.03976] |
| Supplementary Figure 6j | Two-tailed unpaired student's t test | Yellow vs Blue | delta: t(16)=2.173, P=0.0452; R^2^=0.2278, 95% CI = [0.01453, 1.178];  theta: t(16)=2.018, P=0.0606, R^2^=0.2029, 95% CI = [-0.1229, 0.003016];  alpha: t(16)=1.371, P=0.1893, R^2^=0.1051, 95% CI = [-0.02664, 0.1242];  beta: t(16)=1.563, P=0.1377, R^2^=0.1324, 95% CI = [-0.08047, 0.01218];  gamma: t(16)=1.795, P=0.0915, R^2^=0.1676, 95% CI = [-0.006022, 0.07259] |
| Supplementary Figure 6k | Two-tailed Unpaired student's t test or Mann Whitney U test | Blue vs Yellow | delta: t(16)=0.9155, P=0.3756; R^2^=0.04936, 95% CI = [-0.7002, 0.2791];  theta: Mann-Whitney U=25, P=0.1903;  alpha: t(16)=0.3239, P=0.7502, R^2^=0.006514, 95% CI = [-0.09190, 0.1250];  beta: t(16)=0.7170, P= 0.4837, R^2^=0.03113, 95% CI = [-0.08186, 0.04048];  gamma: Mann-Whitney U=40, P>0.9999 |
| Supplementary Figure 6l | Two-tailed Unpaired student's t test or Mann Whitney U test | Blue vs Yellow | delta: t(18)=2.2140, P=0.0400; R^2^=0.2140, 95% CI = [0.01860, 0.7106];  theta: t(18)=1.221, P=0.2379, R^2^=0.07648, 95% CI = [-0.05759, 0.01526];  alpha: t(18)=0.08584, P=0.9325, R^2^=0.0004101, 95% CI = [-0.1231, 0.1134];  beta: Mann-Whitney U=43, P=0.6350;  gamma: Mann-Whitney U=41, P=0.5288 |
| Supplementary Figure 7b | Two-way ANOVA with Dunnett's multiple comparisons test | Yellow vs Blue 10Hz | P=0.0307, 95% CI = [0.02331, 0.6137] |
|  |  | Yellow vs Blue 20Hz | P=0.0001, 95% CI = [0.2195, 0.7879] |
|  |  | Yellow vs Blue 40Hz | P=0.0060, 95% CI = [0.09152, 0.6789] |
| Supplementary Figure 7c | Two-way ANOVA with Dunnett's multiple comparisons test | Yellow vs Blue 10Hz | P=0.0337, 95% CI = [0.1486, 4.785] |
|  |  | Yellow vs Blue 20Hz | P=0.0009, 95% CI = [1.335, 6.228] |
|  |  | Yellow vs Blue 40Hz | P<0.0001, 95% CI = [2.843, 7.357] |
| Supplementary Figure 7e | Two-way ANOVA with Dunnett's multiple comparisons test | Yellow vs Blue 10Hz | P=0.0005, 95% CI = [0.1840, 0.7860] |
|  |  | Yellow vs Blue 20Hz | P=0.0003, 95% CI = [0.2059, 0.8071] |
|  |  | Yellow vs Blue 40Hz | P>0.9999, 95% CI = [-0.3338, 0.3457] |
| Supplementary Figure 7f | Two-way ANOVA with Dunnett's multiple comparisons test | Yellow vs Blue 10Hz | P<0.0001, 95% CI = [3.519, 7.569] |
|  |  | Yellow vs Blue 20Hz | P<0.0001, 95% CI = [4.737, 8.586] |
|  |  | Yellow vs Blue 40Hz | P=0.0737, 95% CI = [-0.1409, 4.049] |
| Supplementary Figure 8c | Two-way ANOVA | Saline vs CNO | F(29,406)=0.6232, P=0.9387, ƞ2 p=0.043, 95% CI = [-0.1197, 0.6447] |
| Supplementary Figure 8d | Two-way ANOVA | Saline vs CNO | F(29,406)=1.500, P=0.0487, ƞ2 p=0.097, 95% CI = [-1.215, 7.173] |
| Supplementary Figure 8e | Two-tailed unpaired student's t test | Saline vs CNO | t(14)=2.147, P=0.0498, R^2^=0.2477, 95% CI = [0.002355, 4.498] |
| Supplementary Figure 9c | Two-tailed unpaired student's t test | EYFP vs taCasp3 | t(4)=9.448, P=0.0007, R^2^=0.9571, 95% CI = [42.37, 77.63] |
| Supplementary Figure 9d | Two-way ANOVA for repeated measures | EYFP vs taCasp3 | F(29,464)=1.208, P=0.2133, ƞ2 p=0.070, 95% CI = [-0.2779, 0.5816] |
| Supplementary Figure 9e | Two-way ANOVA for repeated measures | EYFP vs taCasp3 | F(29,464)=0.6742, P=0.9019, ƞ2 p=0.040, 95% CI = [-2.576, 7.080] |
| Supplementary Figure 9f | Two-way ANOVA with post-hoc Bonferroni test | EYFP vs taCasp3 | Stage 0-2: P=0.8333, 95% CI = [-6.858, 3.303];  Stage 3-4: P>0.9999, 95% CI = [-4.081, 6.081] |
| Supplementary Figure 9g | Two-tailed unpaired student's t test | EYFP vs taCasp3 | t(16)=1.145, P=0.2691, R^2^=0.07571, 95% CI = [-9.506, 2.839] |
| Supplementary Figure 9k | Two-tailed unpaired student's t test | EYFP vs taCasp3 | t(4)=10.12, P=0.0005, R^2^=0.9624, 95% CI = [15.48, 27.19] |
| Supplementary Figure 9l | Two-way ANOVA for repeated measures | EYFP vs taCasp3 | F(29,522)=2.181, P=0.0004, ƞ2 p0.078, 95% CI = [-1.192, -0.3880] |
| Supplementary Figure 9m | Two-way ANOVA for repeated measures | EYFP vs taCasp3 | F(29,522)=1.491, P=0.0495, ƞ2 p=0.053, 95% CI = [-12.82, -1.157] |
| Supplementary Figure 9n | Two-way ANOVA with post-hoc Bonferroni test | EYFP vs taCasp3 | Stage 0-2: P=0.0053;  Stage 3-4: P=0.0471 |
| Supplementary Figure 9o | Two-tailed unpaired student's t test | EYFP vs taCasp3 | t(18)=3.109, P=0.0061, R^2^=0.3494, 95% CI = [2.075, 10.72] |
| Supplementary Figure 10a | Two-way ANOVA for repeated measures | PV vs SST | F(29,435)=1.133, P=0.2927, ƞ2 p=0.070, 95% CI = [-0.3702, 0.8581] |
| Supplementary Figure 10b | Two-way ANOVA for repeated measures | PV vs SST | F(29,435)=0.8144, P=0.7434, ƞ2 p=0.051, 95% CI = [-1.829, 12.74] |
| Supplementary Figure 10c | Two-way ANOVA for repeated measures | PV vs SST | F(29,493)=0.8144, P=0.2504, ƞ2 p=0.064, 95% CI = [-1.080, 0.2853] |
| Supplementary Figure 10d | Two-way ANOVA for repeated measures | PV vs SST | F(29,464)=1.463, P=0.0589, ƞ2 p=0.047, 95% CI = [-0.1172, 0.8061] |
| Supplementary Figure 12b | Two-way ANOVA for repeated measures | Yellow vs Blue | F(29,464)=0.8442, P=0.7018, ƞ2 p=0.084, 95% CI = [-12.23, 3.596] |
| Supplementary Figure 12c | Two-way ANOVA for repeated measures | Yellow vs Blue | F(29,464)=1.499, P=0.082, ƞ2 p=0.086, 95% CI = [0.05822, 14.31] |
| Supplementary Figure 12d | Two-tailed unpaired student's t test | Yellow vs Blue | t(16)=2.289, P=0.0360, R^2^=0.2466, 95% CI = [-8.561, -0.3275] |
| Supplementary Figure 12e | Two-way ANOVA with post-hoc Bonferroni test | Yellow vs Blue | Stage 0-2: P>0.9999, 95% CI = [-4.621, 3.510];  Stage 3-4: P=0.0257, 95% CI = [-8.621, -0.4904] |
| Supplementary Figure 13b | Two-way ANOVA with Tukey's multiple comparison test | Yellow+Saline vs Blue+Saline | P=0.0475, 95% CI = [0.002994, 0.8081] |
|  |  | Yellow+Saline vs Blue+Bicuculline | P=0.9496, 95% CI = [-0.3190, 0.4872] |
|  |  | Yellow+Saline vs Blue+CYN-154806 | P=0.9984, 95% CI = [-0.4206, 0.3698] |
|  |  | Blue+Saline vs Blue+Bicuculline | P=0.1791, 95% CI = [-0.7302, 0.08732] |
|  |  | Blue+Saline vs Blue+CYN-154806 | P=0.0296, 95% CI = [-0.8319, -0.02998] |
| Supplementary Figure 13c | Two-way ANOVA with Tukey's multiple comparison test | Yellow+Saline vs Blue+Saline | P=0.0020, 95% CI = [1.122, 6.745] |
|  |  | Yellow+Saline vs Blue+Bicuculline | P=0.653, 95% CI = [-0.1171, 5.677] |
|  |  | Yellow+Saline vs Blue+CYN-154806 | P=0.9252, 95% CI = [-3.953, 2.419] |
|  |  | Blue+Saline vs Blue+Bicuculline | P=0.6231, 95% CI = [-3.620, 1.313] |
|  |  | Blue+Saline vs Blue+CYN-154806 | P=0.0001, 95% CI = [-7.502, -1.899] |
| Supplementary Figure 13d | Ordinary one-way ANOVA with Tukey's multiple comparison test | Yellow+Saline vs Blue+Saline | Stage 0-2: P=0.0005, 95% CI = [-5.818, -1.515]  Stage 3-4: P=0.0005, 95% CI = [-5.818, -1.515] |
|  |  | Yellow+Saline vs Blue+Bicuculline | Stage 0-2: P=0.0047, 95% CI = [-4.930, -0.7840]  Stage 3-4: P=0.6339, 95% CI = [-1.974, 4.974] |
|  |  | Yellow+Saline vs Blue+CYN-154806 | Stage 0-2: P=0.3366, 95% CI = [-3.359, 0.7875]  Stage 3-4: P=0.0018, 95% CI = [1.747, 8.443] |
|  |  | Blue+Saline vs Blue+Bicuculline | Stage 0-2: P=0.7025, 95% CI = [-1.264, 2.883]  Stage 3-4: P=0.0168, 95% CI = [0.6046, 7.300] |
|  |  | Blue+Saline vs Blue+CYN-154806 | Stage 0-2: P=0.0323, 95% CI = [0.2475, 6.943]  Stage 3-4: P=0.2062, 95% CI = [-0.8954, 5.800] |
| Supplementary Figure 14c | Two-tailed unpaired student's t test | VMH projection vs AHN projection | ZIv: t(4)=8.582, P=0.0010, R^2^=0.9485, 95% CI = [-28.62, -14.63];  ZIr: t(4)=3.976, P=0.0165, R^2^=0.7981, 95% CI = [-13.95, -2.478] |
|  |  | ZIv vs ZIr | VMH projection: t(4)=19.56, P<0.0001, R^2^=0.9897, 95% CI = [-53.81, -40.43];  AHN projection: t(4)=15.37, P=0.0001, R^2^=0.9833, 95% CI = [-39.80, -27.62] |
| Supplementary Figure 16c | Two-way ANOVA with Tukey's multiple comparison test | EYFP-CNO vs taCasp3-Saline | P=0.9776, 95% CI = [-0.2469, 0.2936] |
|  |  | EYFP-CNO vs taCasp3-CNO | P=0.0010, 95% CI = [0.1445, 0.6911] |
|  |  | taCasp3-Saline vs taCasp3-CNO | P=0.0020, 95% CI = [0.1230, 0.6659] |
| Supplementary Figure 16d | Two-way ANOVA with Tukey's multiple comparison test | EYFP-CNO vs taCasp3-Saline | P=0.4427, 95% CI = [-3.202, 1.015] |
|  |  | EYFP-CNO vs taCasp3-CNO | P=0.0304, 95% CI = [0.1479, 3.796] |
|  |  | taCasp3-Saline vs taCasp3-CNO | P=0.0018, 95% CI = [0.9719, 5.158] |
| Supplementary Figure 17d | Mann-Whitney U test | Saline vs CNO | Mann-Whitney U=4, P=0.0260 |
| Supplementary Figure 18c | Two-way ANOVA with post-hoc Bonferroni test | Yellow vs Blue | Pir: P=0.0286, 95% CI = [44.94, 335.7];  DM: P=0.0419, 95% CI = [2.756, 107.2];  CeA: P>0.9999, 95% CI = [-60.90, 58.24];  PVN: P=0.7776, 95% CI = [-72.90, 34.24] |
| Supplementary Figure 19i | Two-tailed unpaired student's t test | EYFP vs taCasp3 | t(6)=10.59, P<0.0001, R^2^=0.9492, 95% CI = [-164.3, -102.7] |
